# Supplementary material for: Termination of STING responses is mediated via ESCRT‐dependent degradation
Source: EMBO J. 2023 May 4;42(12):e112712. doi: 10.15252/embj.2022112712 (PMC10267698; doi:10.15252/embj.2022112712)
Supplement: Supplementary file 1 — Appendix [file EMBJ-42-e112712-s003.pdf]

Appendix

Supplementary figures

Page

**Appendix Figure S1: Phosphoproteomic analysis of primary BMDMs following STING activation**	**2**
**Appendix Figure S2: STING ER-Golgi and vesicular trafficking**	**3**
**Appendix Figure S3: STING interacts with HRS over time in a ubiquitin-dependent fashion**	**4**
**Appendix Figure S4: STING colocalises with HRS and ubiquitin**	**5**

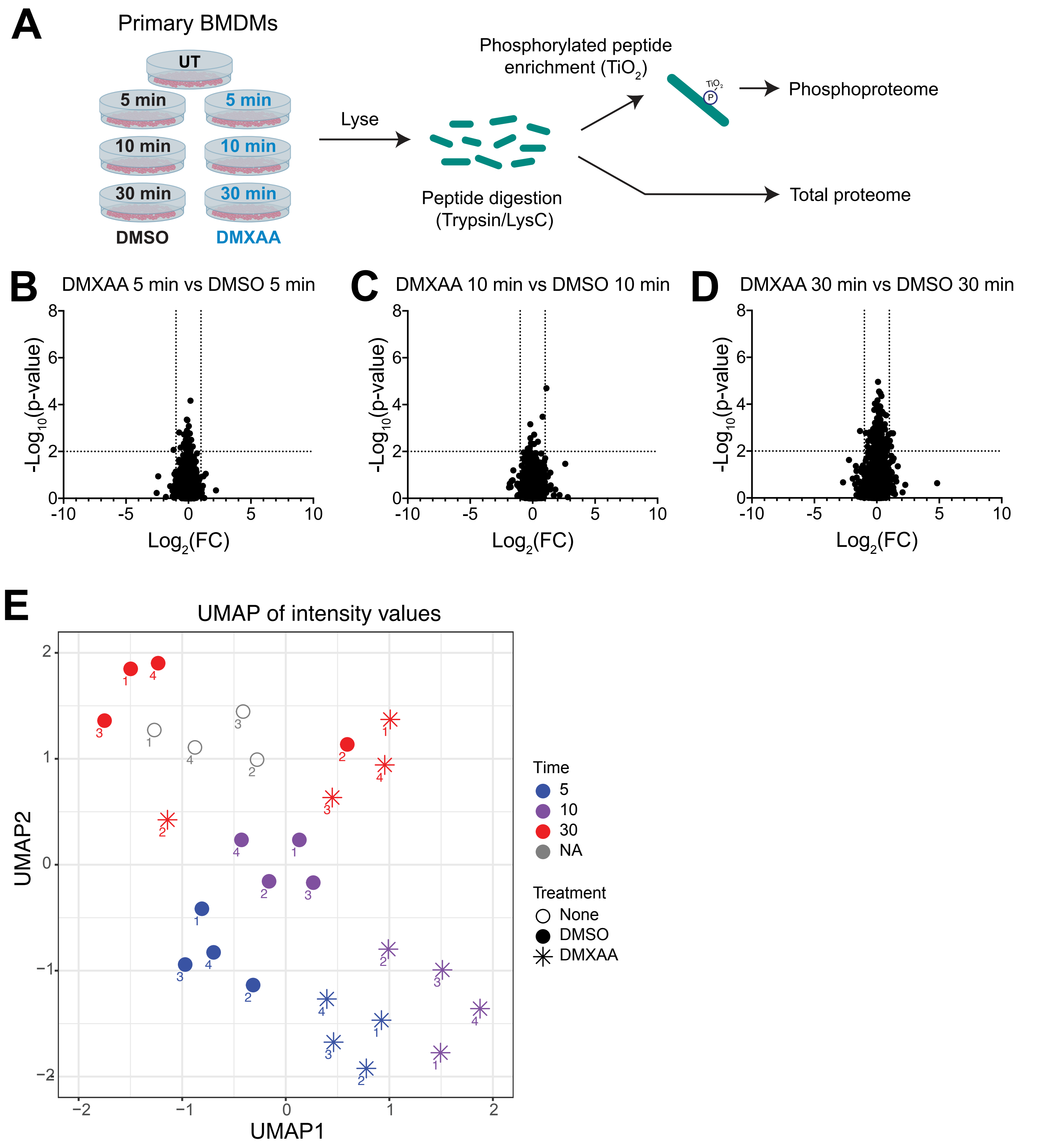

Appendix Figure S1: Phosphoproteomic analysis of primary BMDMs following STING activation

A Schematic showing phosphoproteomic experimental design by primary BMDMs were left untreated (UT) or treated with DMSO (vehicle control) or 50 µg/mL DMXAA for 5, 10 or 30 min. A portion of digest peptides were used for total proteome analysis, while the rest underwent enrichment with titanium oxide beads for phosphorylated peptides, and analysis of the phosphoproteome.

B-D Volcano plots showing changes in total proteome levels for DMXAA vs DMSO groups for 5 min (**B**), 10 min (**C**) and 30 min (**D**) timepoints. -Log10(p-value) is plotted on the y-axis vs Log2(Fold change[FC]) on the x-axis.

E Phosphoproteomic samples were visualised via uniform manifold approximation and projection (UMAP).

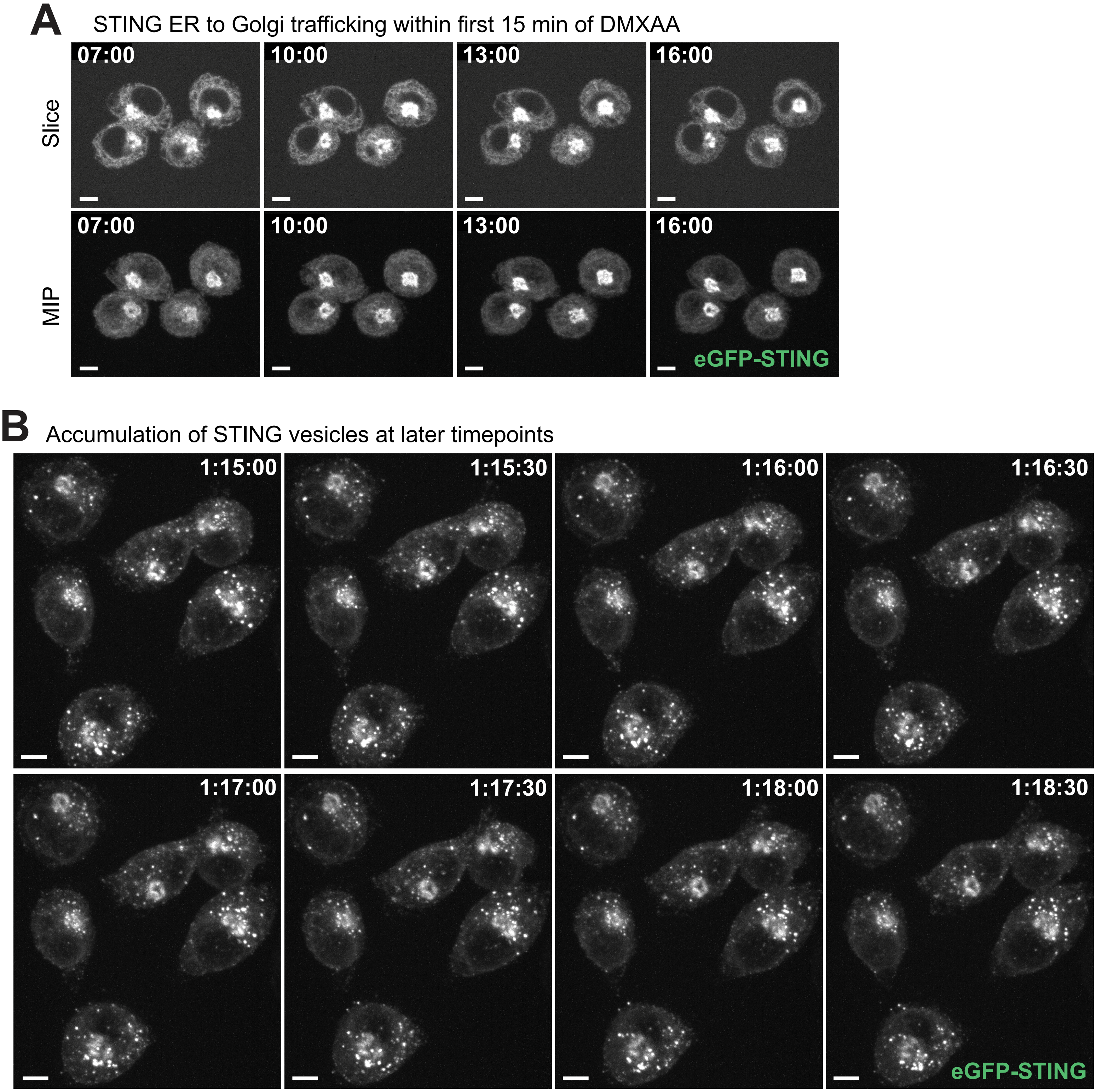

Appendix Figure S2: STING ER-Golgi and vesicular trafficking
A *Sting*$^{-/-}$ iBMDMs expressing eGFP-STING were imaged live on the spinning disk microscope. Images display a time series as indicated, showing STING translocation from ER to Golg after 50 µg/mL DMXAA treatment. Data is shown as a maximum intensity projection (MIP) of Z stack images. Scale bar = 5 µm. Data shown is representative of 3 independent experiments.
B *Sting*$^{-/-}$ iBMDMs expressing eGFP-STING were imaged live on the spinning disk microscope. Images display a time series as indicated, showing transport of STING vesicles for a recording starting 1 h 15 min after 50 µg/mL DMXAA treatment. Data is shown as a maximum intensity projection (MIP) of Z stack images. Scale bar = 5 µm. Data shown is representative of 3 independent experiments.

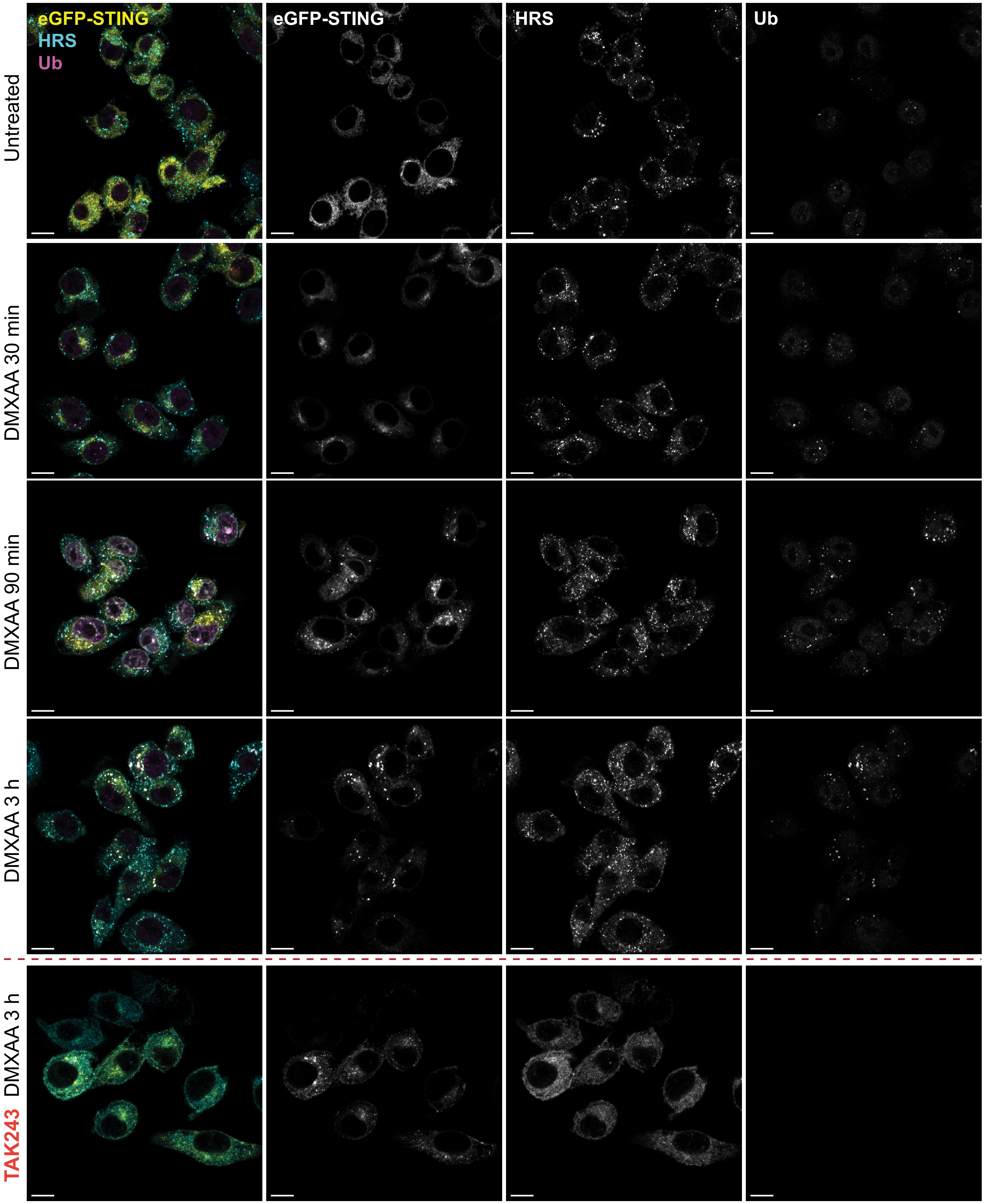

Appendix Figure S3: STING interacts with HRS over time in a ubiquitin-dependent fashion
Sting$^{-/-}$ iBMDMs expressing eGFP-STING (yellow) were left untreated or treated with 50 µg/mL DMXAA for 30 min, 90 min or 3 h. Cells were additionally treated with 2 µM TAK243 (as indicated in red) for 30 min before 50 µg/mL DMXAA for 3 h. Cells were then fixed and underwent immunofluorescence staining for conjugated ubiquitin (Ub; magenta) and HRS (cyan). Z stack images were acquired on the LSM980 confocal microscope. Images are displayed as merged and single colour images for a single Z slice. Scale bar = 10 µm. Data shown is representative of 3 independent experiments. The untreated merged image is also shown in Figure 5C.

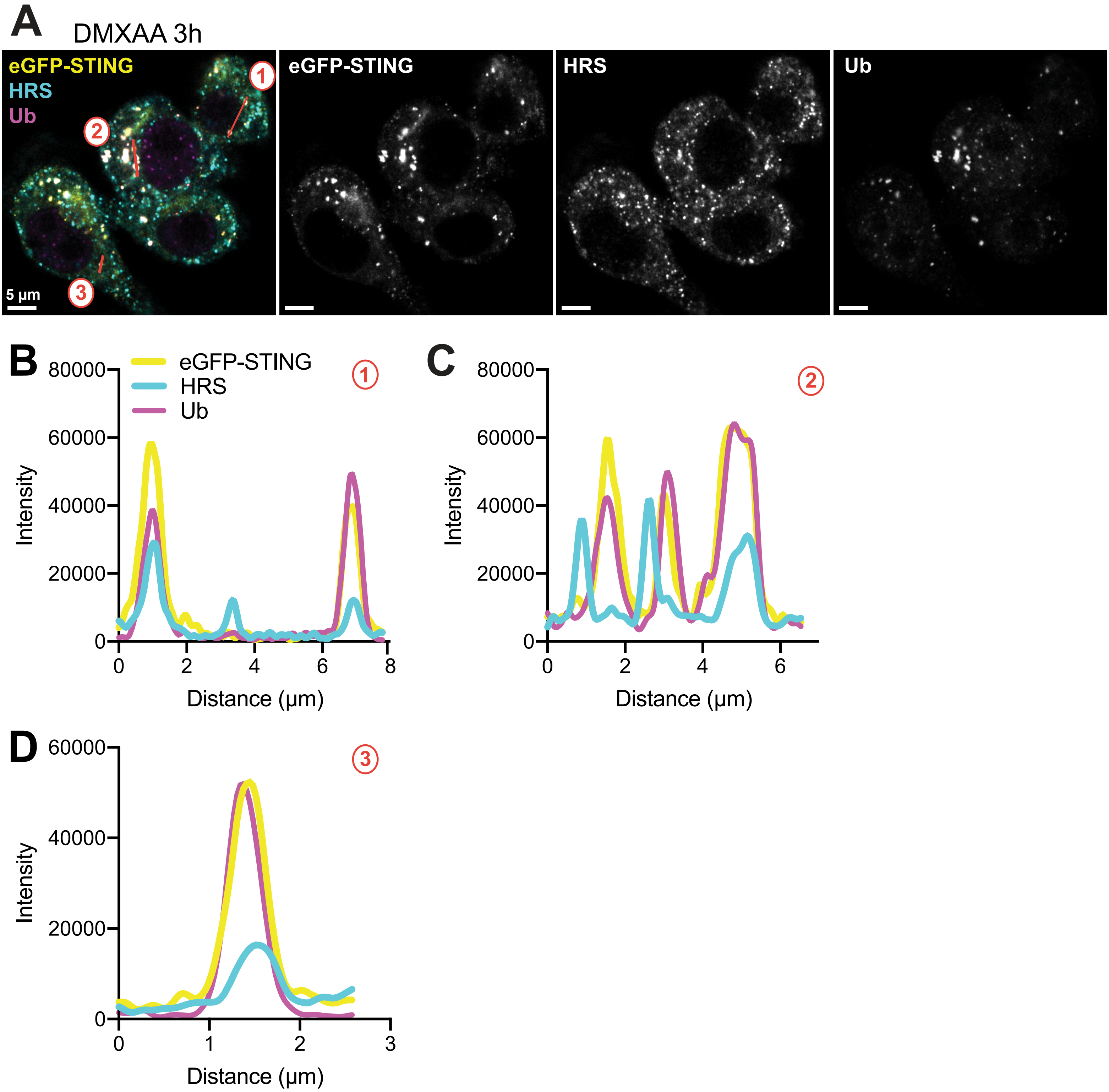

Appendix Figure S4: STING colocalises with HRS and ubiquitin

A *Sting*$^{-/-}$ iBMDMs expressing eGFP-STING (yellow) were left untreated or treated with 50 µg/mL DMXAA for 3 h. Cells were fixed and underwent immunofluorescence staining for conjugated ubiquitin (Ub; magenta) and HRS (cyan). Z stack images were acquired on the LSM980 confocal microscope. Images are displayed as merged images for a single Z slice. Scale bar = 5 µm. Data shown is a zoomed in region from **Appendix Figure S3**, displayed as both merged and single colour images.

B-D The intensity profiles were plotted for each channel for three-line regions as indicated in **A**. This provides an additional example to the data shown in **Figure 5D-F**
