# Supplementary material for: Termination of STING responses is mediated via ESCRT‐dependent degradation
Source: EMBO J. 2023 May 4;42(12):e112712. doi: 10.15252/embj.2022112712 (PMC10267698; doi:10.15252/embj.2022112712)
Supplement: Supplementary file 2 — Expanded View Figures PDF [file EMBJ-42-e112712-s011.pdf]

Expanded View Figures

Figure EV1. Phosphorylation of STING and STING signalling molecules.

A Schematic of the C-terminal tail (CTT) of STING where multiple phosphorylation sites were identified.

B–L The measured intensity values from the phosphoproteomics screen for phosphorylated peptides corresponding to STING (B–E), IKKβ (F), CYLD (G–H), OPTN (I), iRHOM2 (J–L), were plotted for UT, DMSO and DMXAA-treated conditions. The phosphorylated residue is indicated within the graph title. Data shown as mean ± SEM for four replicates. Statistical analysis was performed using unpaired Student's *t*-test. *$P < 0.05$, **$P < 0.01$, ***$P < 0.001$, ****$P < 0.0001$.

© 2023 The Authors

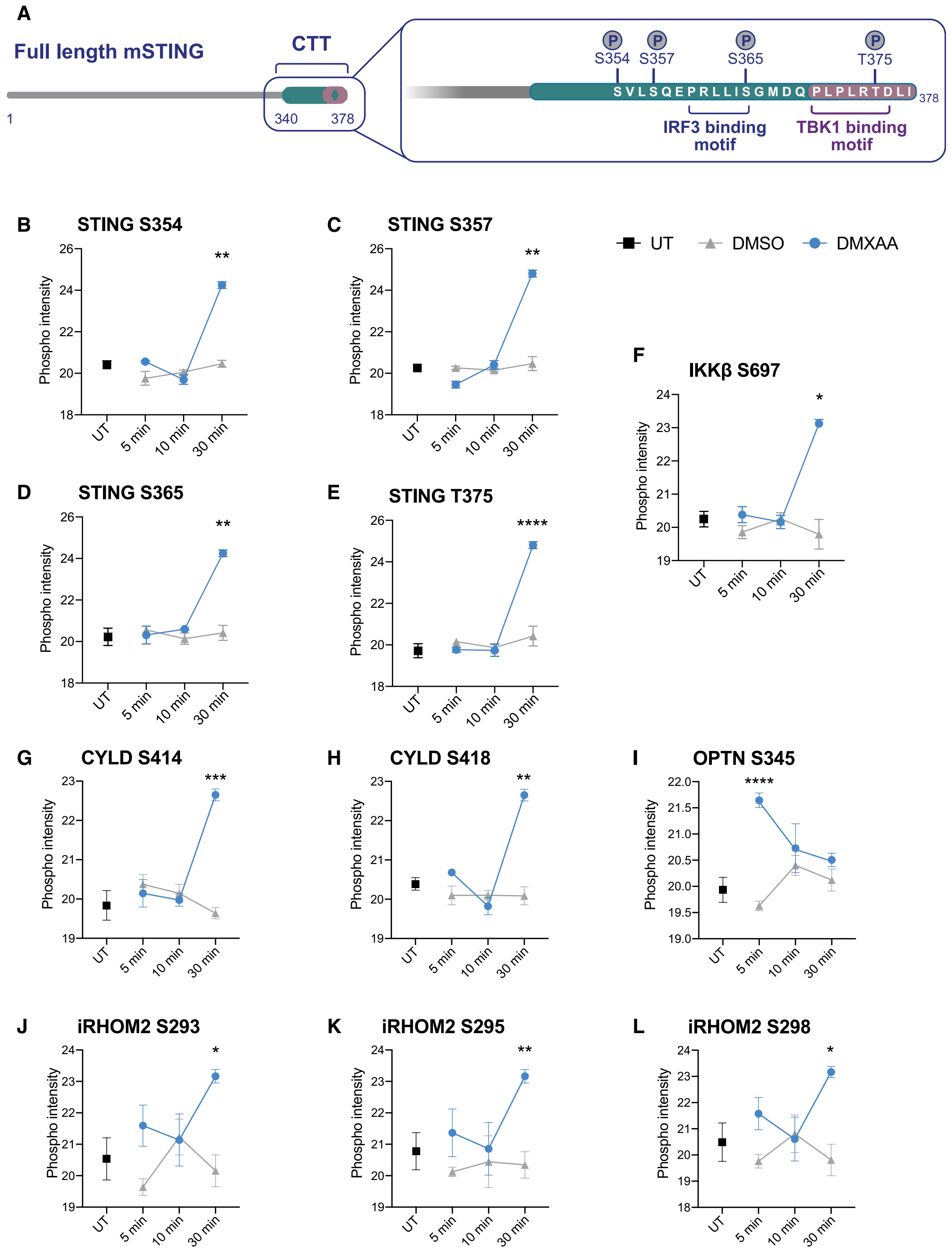
A
Full length mSTING
CTT
1
340
378
S354
S357
S365
T375
S V L S Q E P R L L I S G M D Q P L P L R T D L I
378
IRF3 binding motif
TBK1 binding motif
B STING S354
C STING S357
D STING S365
E STING T375
F IKKβ S697
G CYLD S414
H CYLD S418
I OPTN S345
J iRHOM2 S293
K iRHOM2 S295
L iRHOM2 S298
UT
DMSO
DMXAA
Phospho intensity
5 min
10 min
30 min

Figure EV1.

Figure EV2. Phosphorylation of trafficking proteins in response to STING activation.

A–F The measured intensity values from the phosphoproteomics screen for phosphorylated peptides corresponding to Rab7a (A), CHMP4B (B), LYST (C), TOM1 (D), ATP6V1G3 (E) and CD-MPR (F) were plotted for UT, DMSO and DMXAA-treated conditions. The phosphorylated residue is indicated within the graph title. Data shown as mean ± SEM for four replicates. Statistical analysis was performed using unpaired Student's *t*-test. *$P < 0.05$, **$P < 0.01$, ****$P < 0.0001$.

G Gene ontology (GO) analysis was performed for all proteins found with phosphorylation changes across all three timepoints. Proteins characterised within the organelles STING traffics through were identified using "Cellular components". Proteins localised to the Golgi, endosomes, lysosomes and clathrin-coated vesicles are depicted on a schematic of STING trafficking.

 © 2023 The Authors

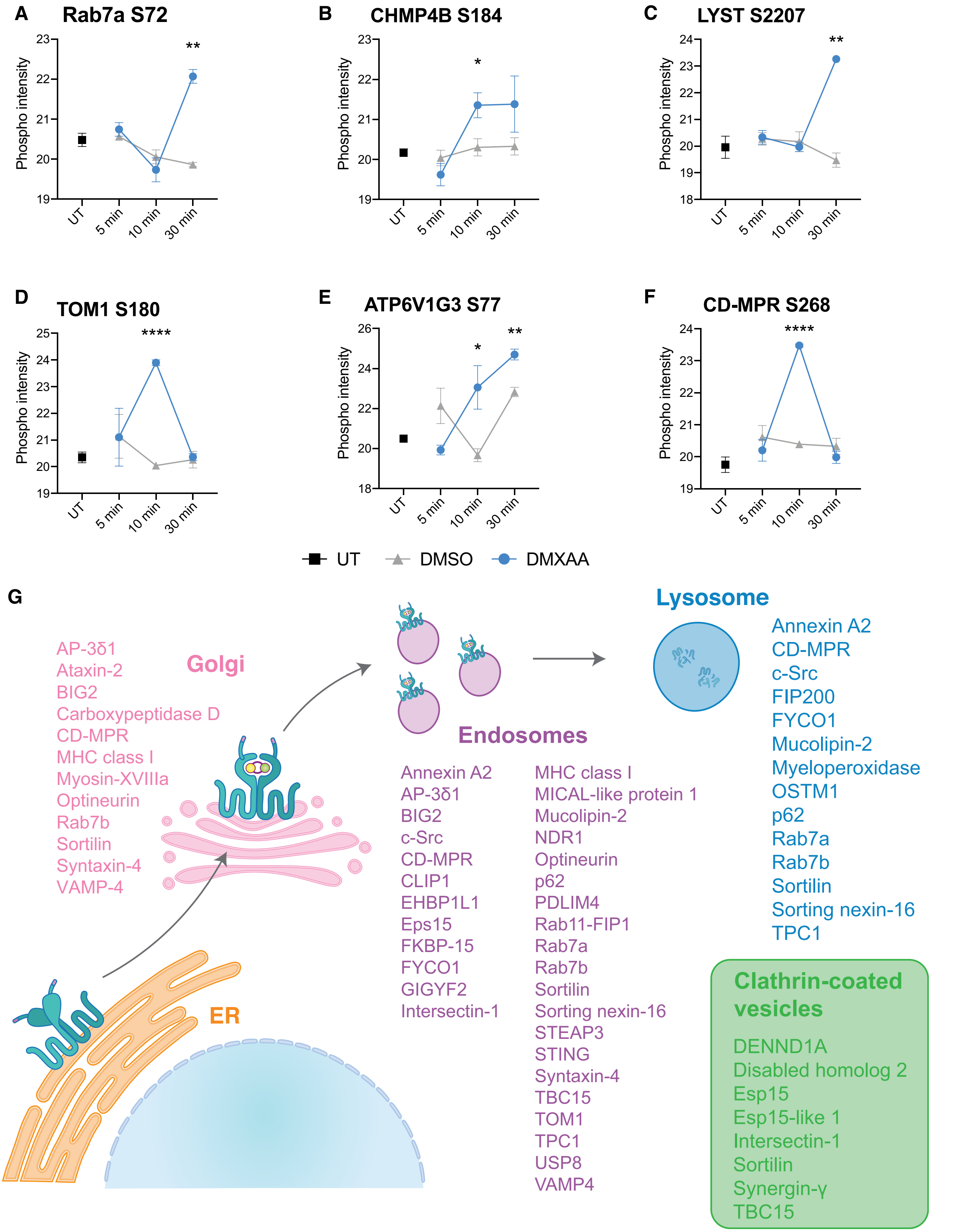
A
Rab7a S72
B
CHMP4B S184
C
LYST S2207
D
TOM1 S180
E
ATP6V1G3 S77
F
CD-MPR S268
Phospho intensity
UT
5 min
10 min
30 min
UT DMSO DMXAA
G
Golgi
AP-3δ1
Ataxin-2
BIG2
Carboxypeptidase D
CD-MPR
MHC class I
Myosin-XVIIIa
Optineurin
Rab7b
Sortilin
Syntaxin-4
VAMP-4
ER
Endosomes
Annexin A2
AP-3δ1
BIG2
c-Src
CD-MPR
CLIP1
EHBP1L1
Eps15
FKBP-15
FYCO1
GIGYF2
Intersectin-1
MHC class I
MICAL-like protein 1
Mucolipin-2
NDR1
Optineurin
p62
PDLIM4
Rab11-FIP1
Rab7a
Rab7b
Sortilin
Sorting nexin-16
STEAP3
STING
Syntaxin-4
TBC15
TOM1
TPC1
USP8
VAMP4
Lysosome
Annexin A2
CD-MPR
c-Src
FIP200
FYCO1
Mucolipin-2
Myeloperoxidase
OSTM1
p62
Rab7a
Rab7b
Sortilin
Sorting nexin-16
TPC1
Clathrin-coated vesicles
DENND1A
Disabled homolog 2
Esp15
Esp15-like 1
Intersectin-1
Sortilin
Synergin-γ
TBC15

Figure EV2.

Figure EV3. STING rapidly traffics from the ER to the Golgi.

A *Sting*$^{-/-}$ iBMDMs alone or *Sting*$^{-/-}$ iBMDMs reconstituted with either eGFP- or mRuby3-tagged STING were treated with 50 μg/ml BMDMs for 2 h. Cells were lysed for immunoblot with the indicated antibodies. Data shown are representative of three independent experiments.

B *Sting*$^{-/-}$ iBMDMs expressing eGFP-STING were imaged using spinning disk microscopy in resting conditions. Data are shown as both a single Z slice and maximum intensity projection (MIP) of Z stack images. Data shown are representative of three independent experiments. Scale bar = 5 μm.

C *Sting*$^{-/-}$ iBMDMs expressing eGFP-STING were imaged live on the spinning disk microscope. Images display a time series as indicated, showing STING translocation from ER to Golgi after 50 μg/ml DMXAA treatment. Data are shown as both a single Z slice and MIP of Z stack images. Data shown are representative of three independent experiments. Scale bar = 5 μm. Corresponds to Movie EV2.

D *Sting*$^{-/-}$ iBMDMs expressing eGFP-STING were imaged live on the spinning disk microscope. Images display a time series as indicated, showing STING vesicles exiting the Golgi for a recording starting 30 min after 50 μg/ml DMXAA treatment. Data are shown as a MIP of Z stack images. Data shown are representative of three independent experiments. Scale bar = 5 μm. Corresponds to Movie EV5.

Source data are available online for this figure.

 © 2023 The Authors

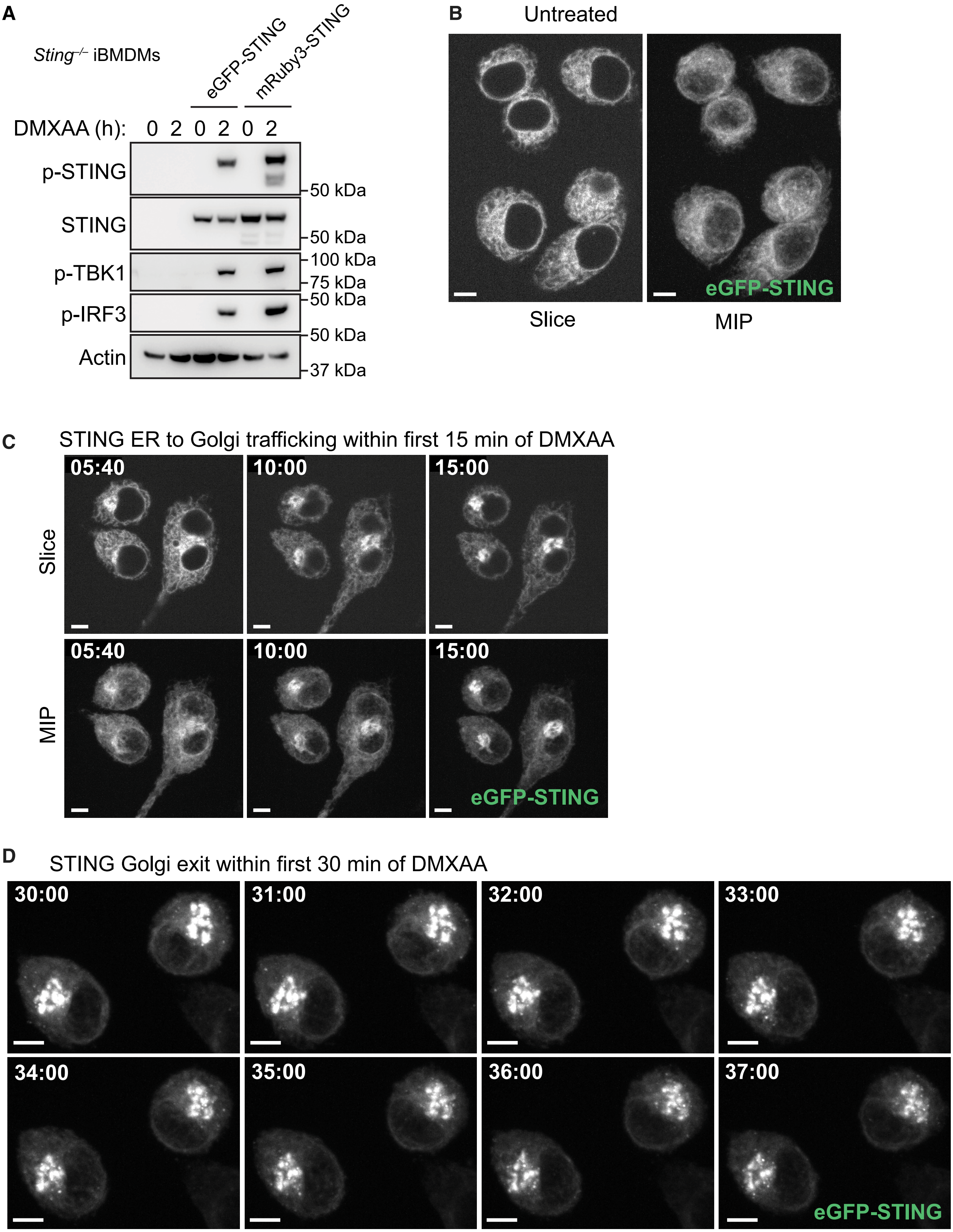
A
Sting−/− iBMDMs
eGFP-STING
mRuby3-STING
DMXAA (h): 0 2 0 2 0 2
p-STING
STING
p-TBK1
p-IRF3
Actin
50 kDa
50 kDa
100 kDa
75 kDa
50 kDa
50 kDa
37 kDa
B
Untreated
eGFP-STING
Slice
MIP
C
STING ER to Golgi trafficking within first 15 min of DMXAA
05:40
10:00
15:00
Slice
MIP
eGFP-STING
D
STING Golgi exit within first 30 min of DMXAA
30:00
31:00
32:00
33:00
34:00
35:00
36:00
37:00
eGFP-STING

Figure EV3.

Figure EV4. STING interacts with HRS.

A WT or TBK1/IKKε^{dKO} iBMDMs were left untreated (i.e. 0 h) or treated with 50 μg/ml DMXAA for 1 or 3 h. Cells were lysed and a portion of lysate underwent immunoblot with the indicated antibodies (i.e. lysate). The remaining lysate was incubated with TUBE beads to isolate ubiquitinated proteins (i.e. TUBE). TUBE samples underwent immunoblot with the indicated antibodies. Data representative of three independent experiments.

B *Sting*$^{-/-}$ iBMDMs or *Sting*$^{-/-}$ iBMDMs expressing HA-STING were left untreated or treated with 50 μg/ml DMXAA for 3 h. Cells were lysed and a portion of lysate underwent immunoblot with the indicated antibodies (i.e. lysate). The remaining lysate underwent immunoprecipitation with an anti-HA antibody (i.e. HA pull down). Samples then underwent immunoblot with the indicated antibodies. Data representative of three independent experiments.

C Primary BMDMs were left untreated or treated with 50 μg/ml DMXAA for 3 h. Cells were lysed and a portion of lysate underwent immunoblot with the indicated antibodies (i.e. lysate). The remaining lysate underwent immunoprecipitation with an anti-STING antibody (i.e. STING pull down). Samples then underwent immunoblot with the indicated antibodies. Data representative of three independent experiments.

D *Sting*$^{-/-}$ iBMDMs expressing eGFP-STING (yellow) were treated with 50 μg/ml DMXAA for 3 h. Cells were fixed and underwent immunofluorescence staining for conjugated ubiquitin (Ub; magenta) and HRS (cyan). Data show two channel merged images relating to those found in Fig 5D. Scale bar = 5 μm.

Source data are available online for this figure.

 © 2023 The Authors

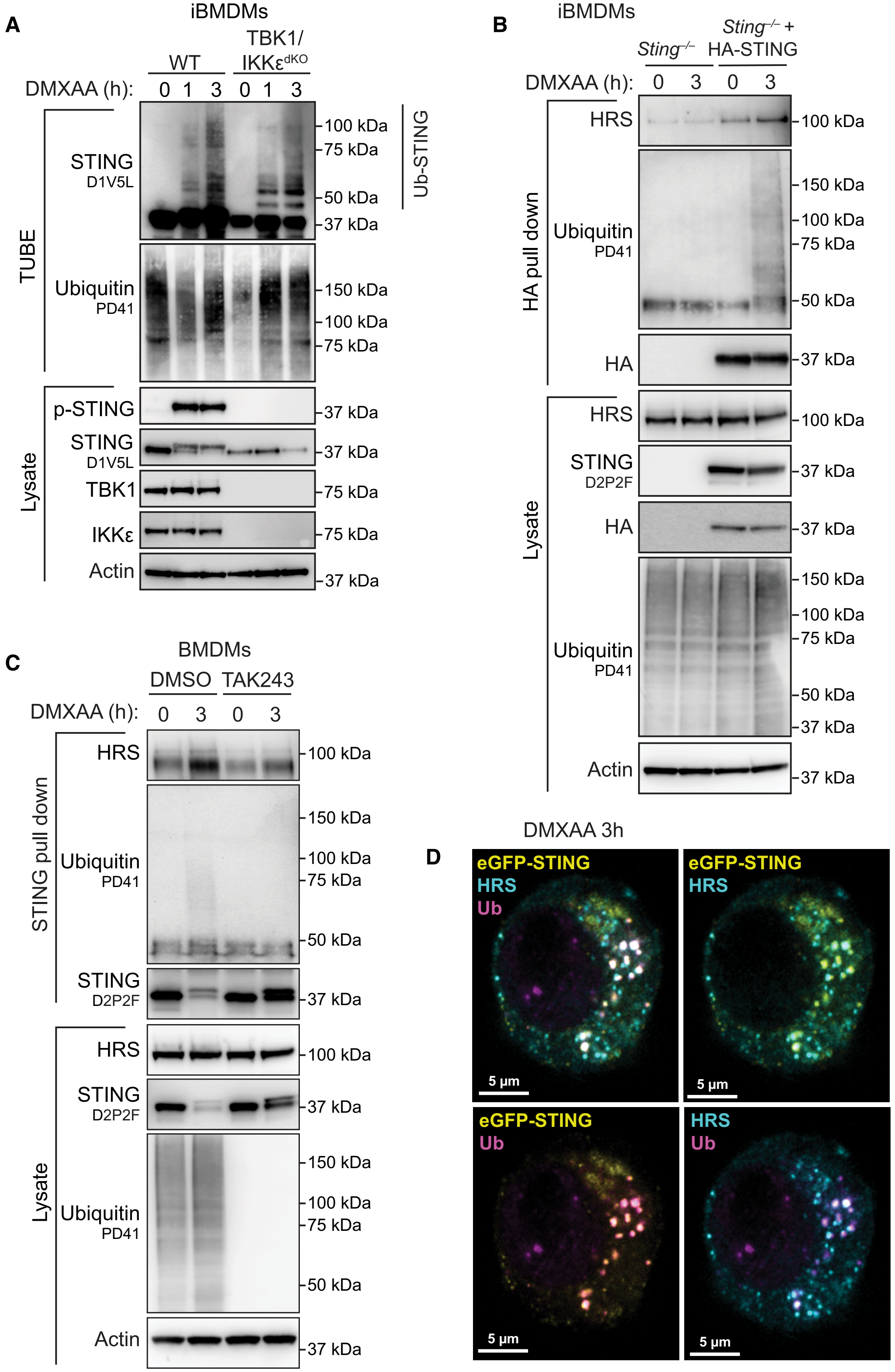
A
iBMDMs
WT
TBK1/ IKKε^{dKO}
DMXAA (h): 0 1 3 0 1 3
TUBE
STING D1V5L
Ubiquitin PD41
Lysate
p-STING
STING D1V5L
TBK1
IKKε
Actin
100 kDa
75 kDa
50 kDa
37 kDa
150 kDa
Ub-STING
B
iBMDMs
Sting−/−
Sting−/− + HA-STING
DMXAA (h): 0 3 0 3
HA pull down
HRS
Ubiquitin PD41
HA
Lysate
STING D2P2F
C
BMDMs
DMSO TAK243
STING pull down
D
DMXAA 3h
eGFP-STING
HRS
Ub
5 µm

Figure EV4.

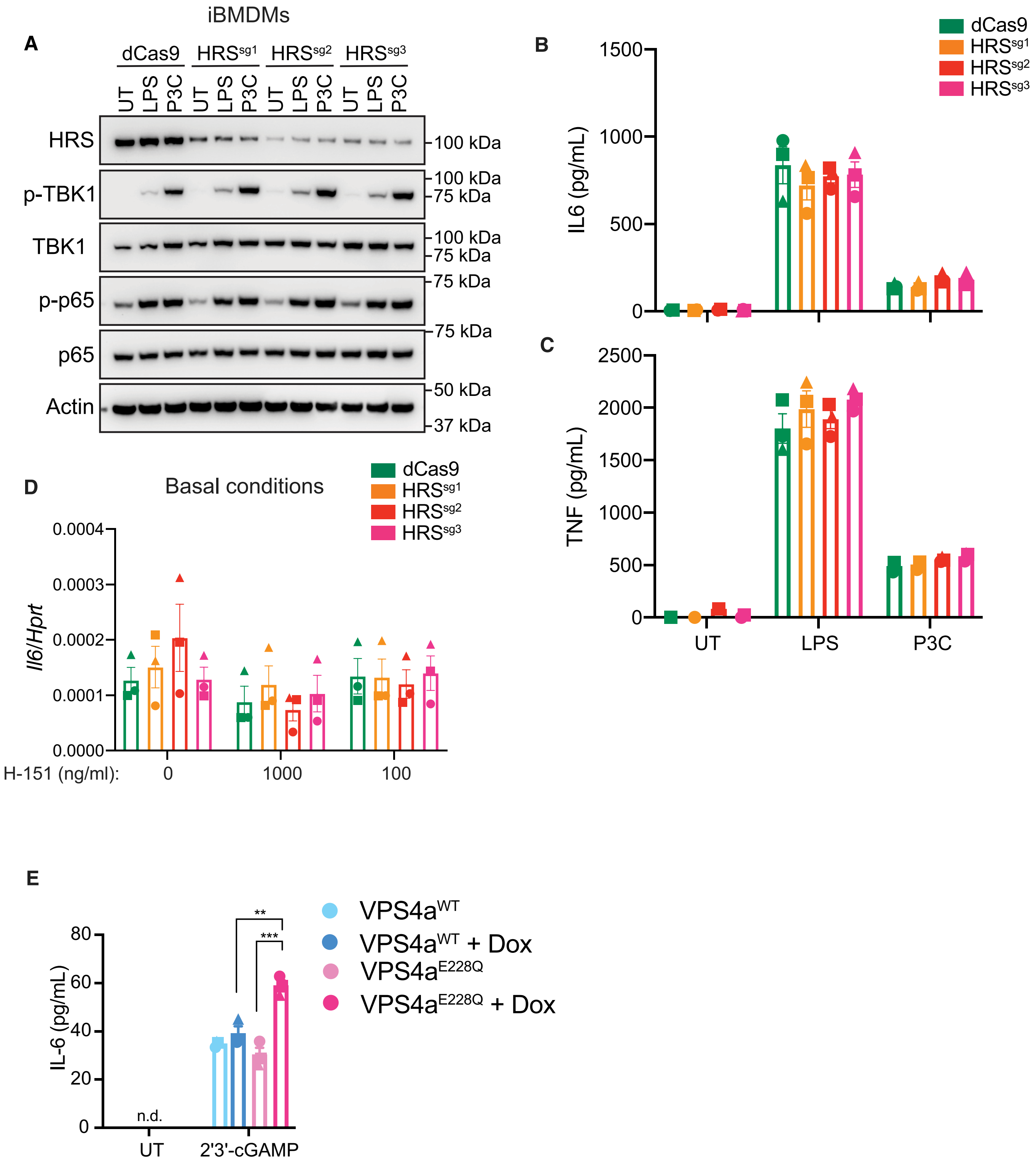

Figure EV5.

© 2023 The Authors

Figure EV5. HRS-depleted iBMDMs maintain normal TLR responses.

A–C dCas9-KRAB expressing iBMDMs, without (i.e. dCas9 alone) or with HRS-targeting sgRNAs (i.e. sg1, sg2 or sg3) were treated for 48 h with 1 μg/ml doxycycline (Dox) to induce sgRNA expression. Cells were then left untreated (UT), or treated with 500 ng/ml LPS or 200 ng/ml Pam3CSK4 (P3C) for 6 h. A Cells were lysed for immunoblot with the indicated antibodies. Data shown are representative of three independent experiments. (B, C) Cell supernatant was collected and assayed for secreted IL-6 (B) or TNF (C) by ELISA. Data are shown as mean ± SEM combined from $N = 3$ independent experiments. Statistical analysis was performed using two-way ANOVA using Bonferroni's multiple comparisons test, where no statically significant differences were observed across cell lines for the same treatment.

D dCas9-KRAB expressing iBMDMs, without (i.e. dCas9 alone) or with HRS-targeting sgRNAs (i.e. sg1, sg2 or sg3) were treated for 24 h with 1 μg/ml Dox to induce sgRNA expression. iBMDMs were further treated with 1 μg/ml (i.e., 1,000 ng/ml) or 100 ng/ml H-151 as indicated and incubated for a further 24 h. Cells were lysed for RNA purification and the expression of *Il6* was analysed by qPCR. Data are shown as mean ± SEM combined from $N = 3$ independent experiments. Statistical analysis was performed using two-way ANOVA using Bonferroni's multiple comparisons test, where no statistically significant changes were observed.

E VPS4a^{WT} or VPS4a^{E228Q} dominant negative BMDMs were left UT or treated with 1 μg/ml Dox for 4 h. iBMDMs were then further left UT or treated with 10 μg/ml 2′3′-cGAM(PS)2 for 4 h. Cell supernatant was collected and secreted IL-6 was measured by ELISA. Data are shown as mean ± SEM combined from $N = 3$ independent experiments. Statistical analysis was performed using one-way ANOVA using Bonferroni's multiple comparisons test, where $^{**}P < 0.01$, $^{***}P < 0.001$. n.d., not detected.

Source data are available online for this figure.

© 2023 The Authors
