# Supplementary material for: Termination of STING responses is mediated via ESCRT‐dependent degradation
Source: EMBO J. 2023 May 4;42(12):e112712. doi: 10.15252/embj.2022112712 (PMC10267698; doi:10.15252/embj.2022112712)
Supplement: Supplementary file 3 — Movie EV1 [file EMBJ-42-e112712-s013.zip › Movie EV1/Movie EV1.rtf]

Movie EV1: STING resides predominantly in the ER at basal conditionsSting–/– iBMDMs expressing eGFP-STING were imaged using spinning disk microscopy in resting conditions. Z stacks were acquired every 5 seconds for 50 frames (i.e., total imaging time ~ 4 min). Movie shown at 5 frames per second (fps). 
