# Supplementary material for: Termination of STING responses is mediated via ESCRT‐dependent degradation
Source: EMBO J. 2023 May 4;42(12):e112712. doi: 10.15252/embj.2022112712 (PMC10267698; doi:10.15252/embj.2022112712)
Supplement: Supplementary file 4 — Movie EV2 [file EMBJ-42-e112712-s015.zip › Movie EV2/Movie EV2.rtf]

Movie EV2: STING rapidly translocates to Golgi regions upon activationSting–/– iBMDMs expressing eGFP-STING were imaged using spinning disk microscopy. Movie starts 5 min 40 sec after addition of 50 g/mL DMXAA. Z stacks were acquired every 5 seconds for 200 frames (i.e., total imaging time ~ 17 min). Movie shown at 5 frames per second (fps). Corresponds to Figure EV3C.
