# Supplementary material for: Termination of STING responses is mediated via ESCRT‐dependent degradation
Source: EMBO J. 2023 May 4;42(12):e112712. doi: 10.15252/embj.2022112712 (PMC10267698; doi:10.15252/embj.2022112712)
Supplement: Supplementary file 5 — Movie EV3 [file EMBJ-42-e112712-s007.zip › Movie EV3/Movie EV3.rtf]

Movie EV3: STING rapidly translocates to Golgi regions upon activation Sting–/– iBMDMs expressing mRuby3-STING were imaged using spinning disk microscopy. Movie starts ~3 min after addition of 50 g/mL DMXAA. Z stacks were acquired every 20 seconds for 30 frames (i.e., total imaging time ~ 10 min). Movie shown at 5 frames per second (fps). 
