# Supplementary material for: Termination of STING responses is mediated via ESCRT‐dependent degradation
Source: EMBO J. 2023 May 4;42(12):e112712. doi: 10.15252/embj.2022112712 (PMC10267698; doi:10.15252/embj.2022112712)
Supplement: Supplementary file 6 — Movie EV4 [file EMBJ-42-e112712-s012.zip › Movie EV4/Movie EV4.rtf]

Movie EV4: STING exists the Golgi on vesicles Sting–/– iBMDMs expressing mRuby3-STING were imaged using spinning disk microscopy. Movie starts 27 min after addition of 50 g/mL DMXAA. Z stacks were acquired every 20 seconds for 120 frames (i.e., total imaging time ~ 40 min). Movie shown at 5 frames per second (fps). Corresponds to Figure 2D.
