# Supplementary material for: Termination of STING responses is mediated via ESCRT‐dependent degradation
Source: EMBO J. 2023 May 4;42(12):e112712. doi: 10.15252/embj.2022112712 (PMC10267698; doi:10.15252/embj.2022112712)
Supplement: Supplementary file 7 — Movie EV5 [file EMBJ-42-e112712-s004.zip › Movie EV5/Movie EV5.rtf]

Movie EV5: STING exists the Golgi on vesicles Sting–/– iBMDMs expressing eGFP-STING were imaged using spinning disk microscopy. Movie starts 29 min after addition of 50 g/mL DMXAA. Z stacks were acquired every 10 seconds for 50 frames (i.e., total imaging time ~ 8 min). Movie shown at 5 frames per second (fps). Corresponds to Figure EV3D.
