# Supplementary material for: Termination of STING responses is mediated via ESCRT‐dependent degradation
Source: EMBO J. 2023 May 4;42(12):e112712. doi: 10.15252/embj.2022112712 (PMC10267698; doi:10.15252/embj.2022112712)
Supplement: Supplementary file 8 — Movie EV6 [file EMBJ-42-e112712-s018.zip › Movie EV6/Movie EV6.rtf]

Movie EV6: Vesicular STING trafficking increases over time Sting–/– iBMDMs expressing eGFP-STING were imaged using spinning disk microscopy. Movie starts 1 h 18 min after addition of 50 g/mL DMXAA. Z stacks were acquired every 5 seconds for 100 frames (i.e., total imaging time ~ 9 min). Movies are shown as 5 fps. Corresponds to Figure 2F.
