# Supplementary material for: Termination of STING responses is mediated via ESCRT‐dependent degradation
Source: EMBO J. 2023 May 4;42(12):e112712. doi: 10.15252/embj.2022112712 (PMC10267698; doi:10.15252/embj.2022112712)
Supplement: Supplementary file 10 — Source Data for Expanded View and Appendix [file EMBJ-42-e112712-s001.zip › EV:S Figures/Figure EV4/Figure EV4A.pdf]

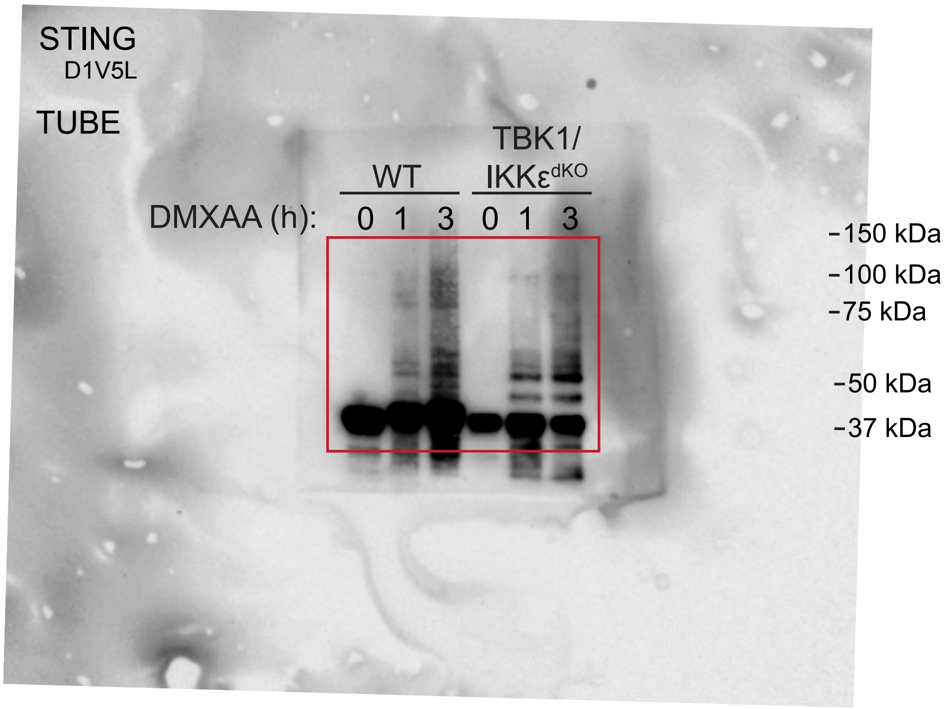
STING
D1V5L
TUBE
TBK1/
WT
IKKε dKO
DMXAA (h): 0 1 3 0 1 3
-150 kDa
-100 kDa
-75 kDa
-50 kDa
-37 kDa

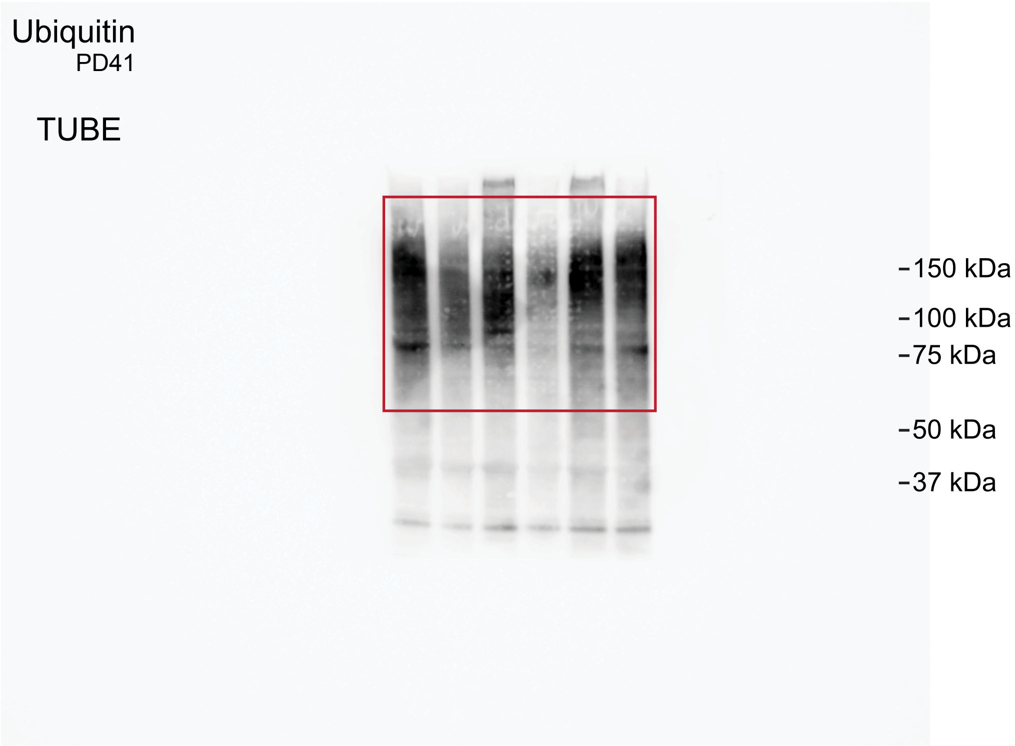
Ubiquitin
PD41
TUBE
-150 kDa
-100 kDa
-75 kDa
-50 kDa
-37 kDa

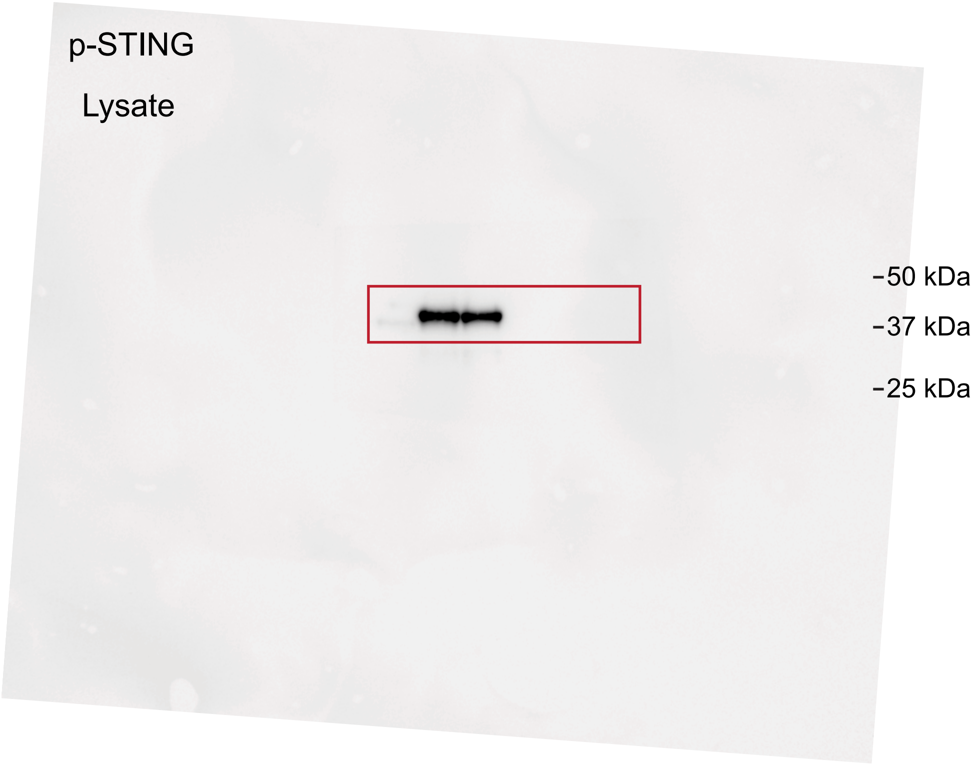
p-STING
Lysate
-50 kDa
-37 kDa
-25 kDa

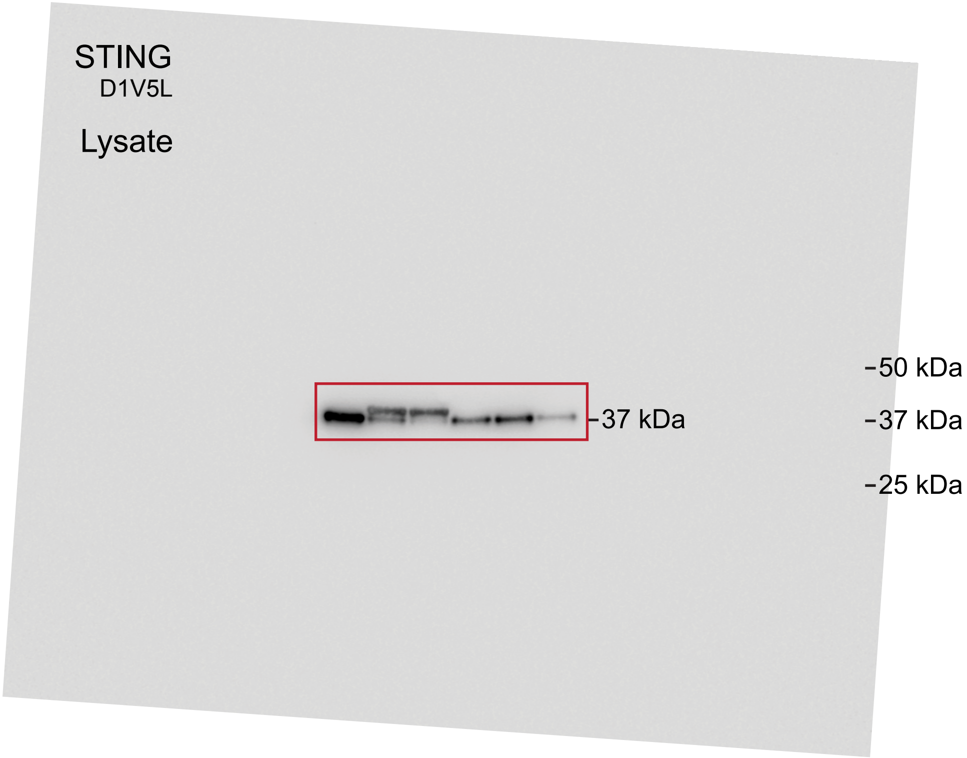
STING
D1V5L
Lysate
-37 kDa
-50 kDa
-37 kDa
-25 kDa

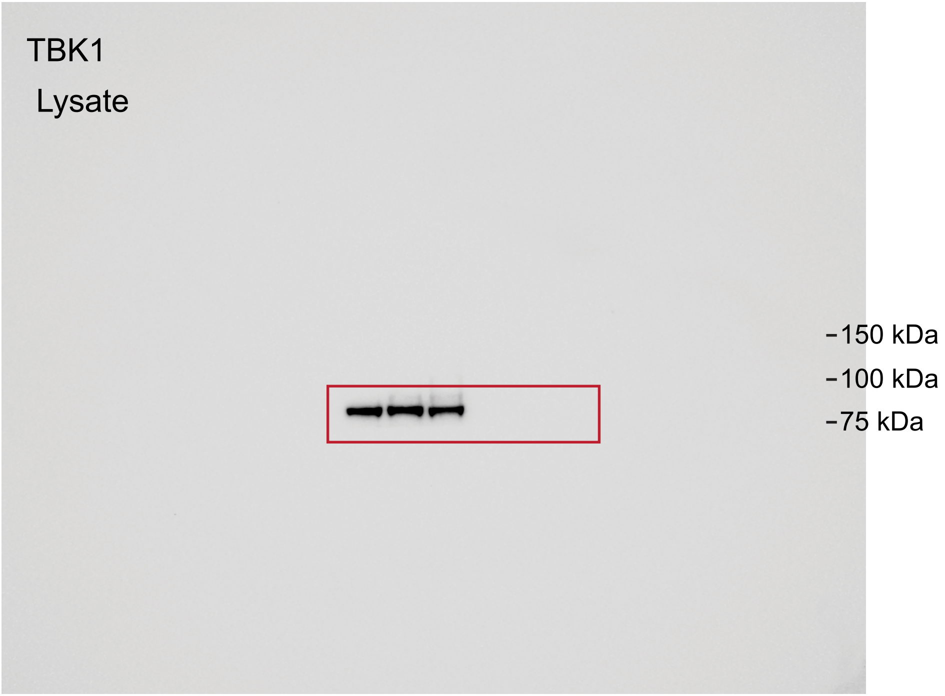
TBK1
Lysate
-150 kDa
-100 kDa
-75 kDa

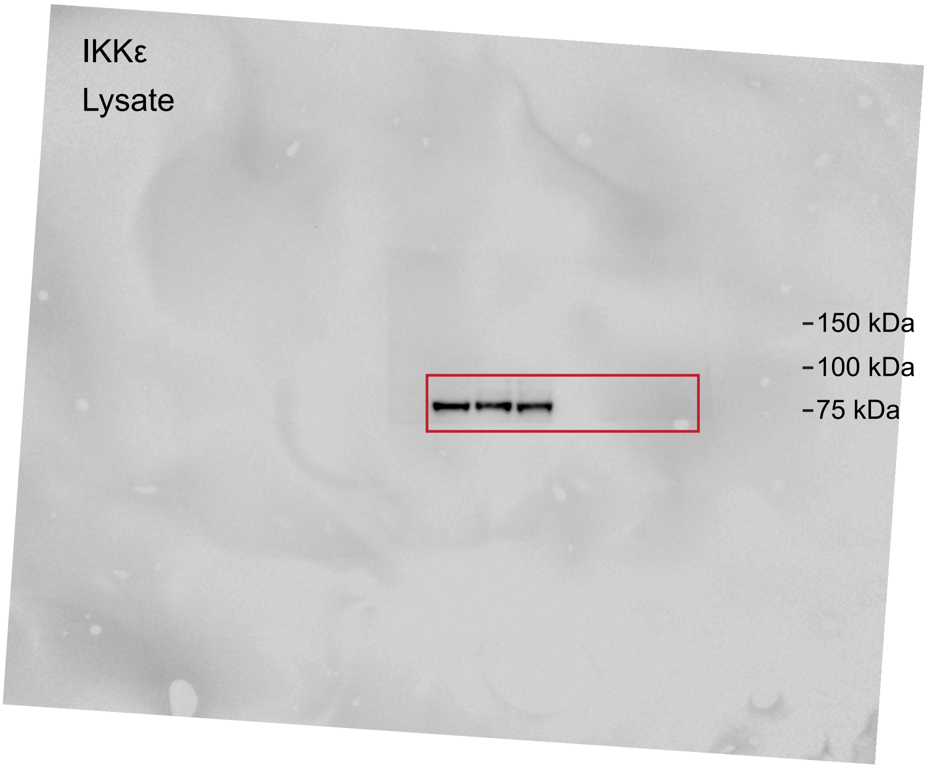
IKKε
Lysate
-150 kDa
-100 kDa
-75 kDa

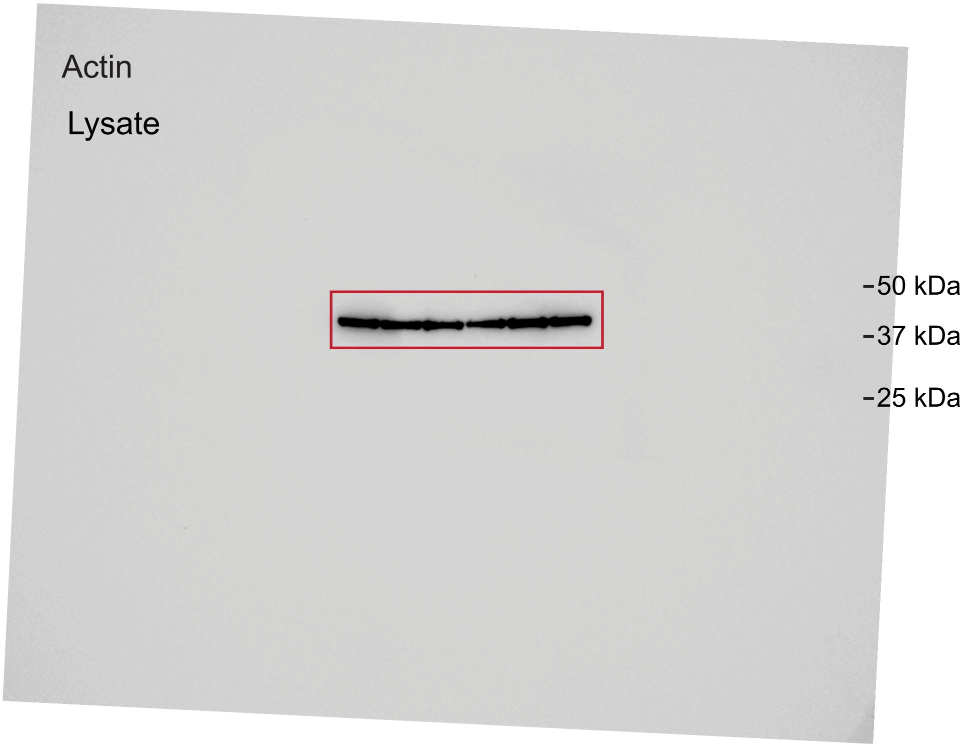
Actin
Lysate
-50 kDa
-37 kDa
-25 kDa
