# Supplementary material for: Termination of STING responses is mediated via ESCRT‐dependent degradation
Source: EMBO J. 2023 May 4;42(12):e112712. doi: 10.15252/embj.2022112712 (PMC10267698; doi:10.15252/embj.2022112712)
Supplement: Supplementary file 10 — Source Data for Expanded View and Appendix [file EMBJ-42-e112712-s001.zip › EV:S Figures/Figure EV4/Figure EV4D.rtf]

Source data for Figure EV4D can be found with the source data for Figure 5D
