# Supplementary material for: Termination of STING responses is mediated via ESCRT‐dependent degradation
Source: EMBO J. 2023 May 4;42(12):e112712. doi: 10.15252/embj.2022112712 (PMC10267698; doi:10.15252/embj.2022112712)
Supplement: Supplementary file 10 — Source Data for Expanded View and Appendix [file EMBJ-42-e112712-s001.zip › EV:S Figures/Figure EV5/Figure EV5A.pdf]

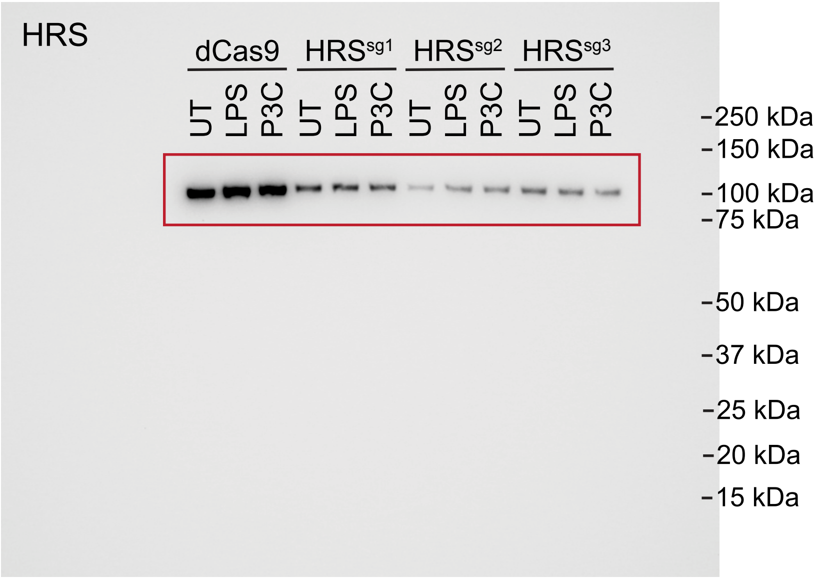
HRS
dCas9
HRSsg1
HRSsg2
HRSsg3
UT
LPS
P3C
UT
LPS
P3C
UT
LPS
P3C
UT
LPS
P3C
-250 kDa
-150 kDa
-100 kDa
-75 kDa
-50 kDa
-37 kDa
-25 kDa
-20 kDa
-15 kDa

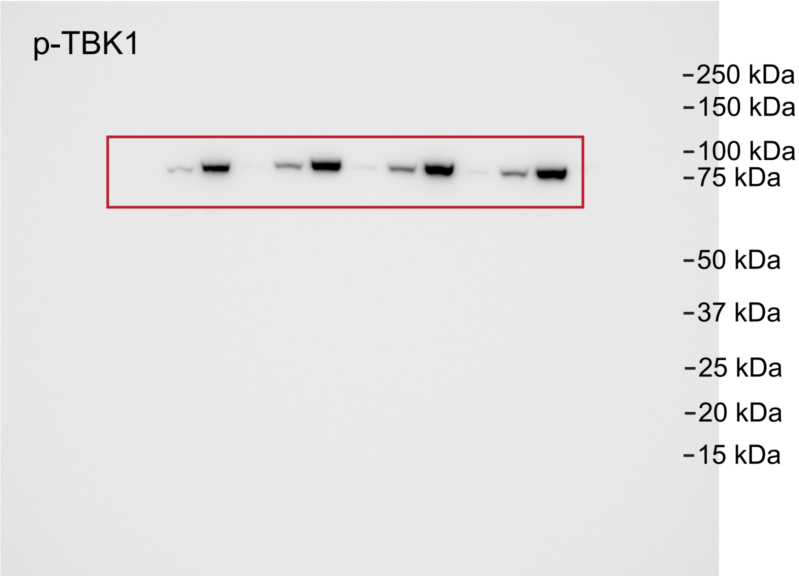
p-TBK1
-250 kDa
-150 kDa
-100 kDa
-75 kDa
-50 kDa
-37 kDa
-25 kDa
-20 kDa
-15 kDa

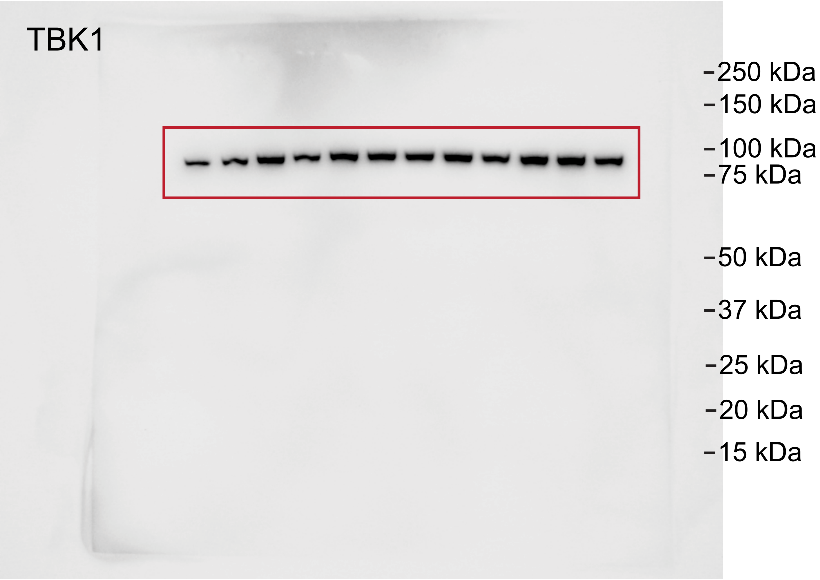
TBK1
-250 kDa
-150 kDa
-100 kDa
-75 kDa
-50 kDa
-37 kDa
-25 kDa
-20 kDa
-15 kDa

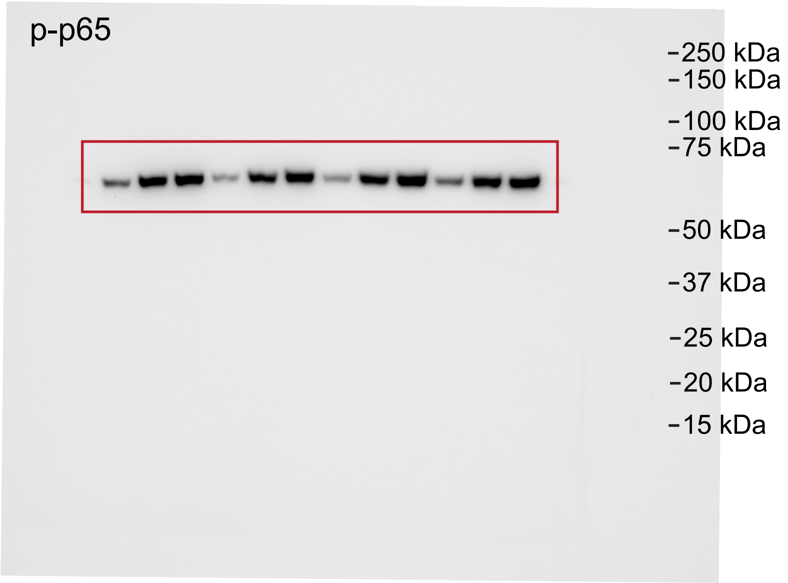
p-p65
-250 kDa
-150 kDa
-100 kDa
-75 kDa
-50 kDa
-37 kDa
-25 kDa
-20 kDa
-15 kDa

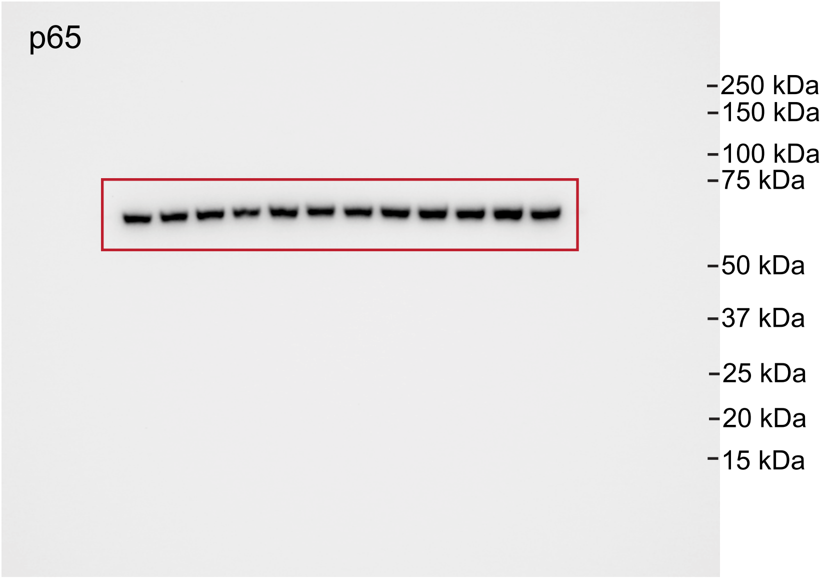
p65
-250 kDa
-150 kDa
-100 kDa
-75 kDa
-50 kDa
-37 kDa
-25 kDa
-20 kDa
-15 kDa

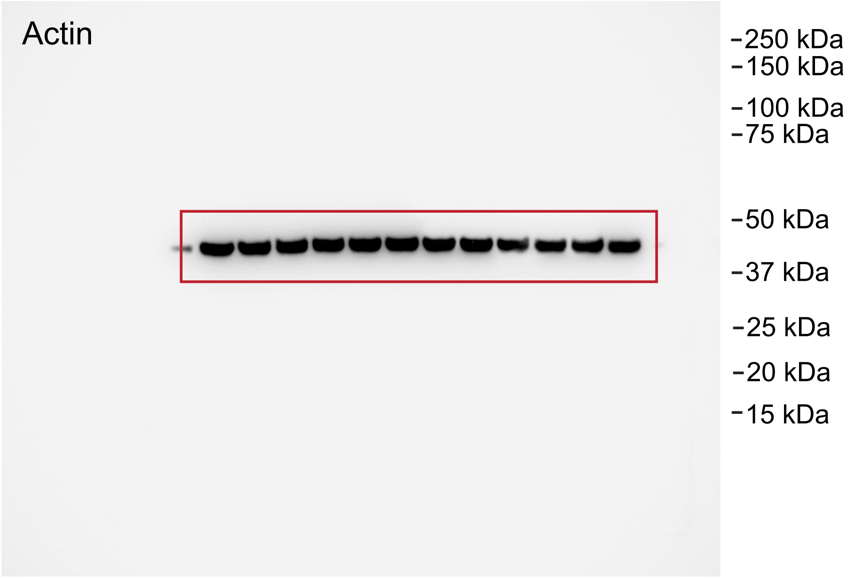
Actin
-250 kDa
-150 kDa
-100 kDa
-75 kDa
-50 kDa
-37 kDa
-25 kDa
-20 kDa
-15 kDa
