# Supplementary figures and images for: Termination of STING responses is mediated via ESCRT‐dependent degradation
Source: EMBO J. 2023 May 4;42(12):e112712. doi: 10.15252/embj.2022112712 (PMC10267698; doi:10.15252/embj.2022112712)

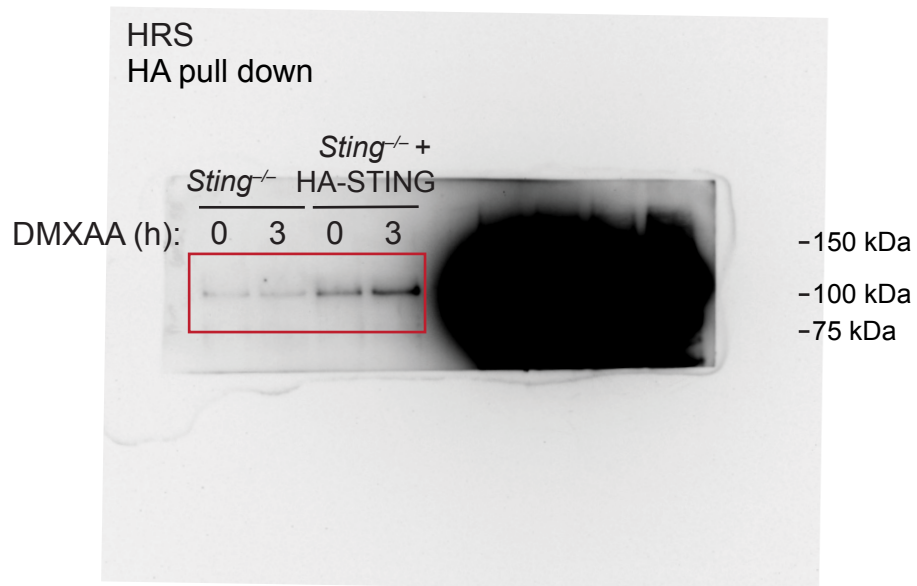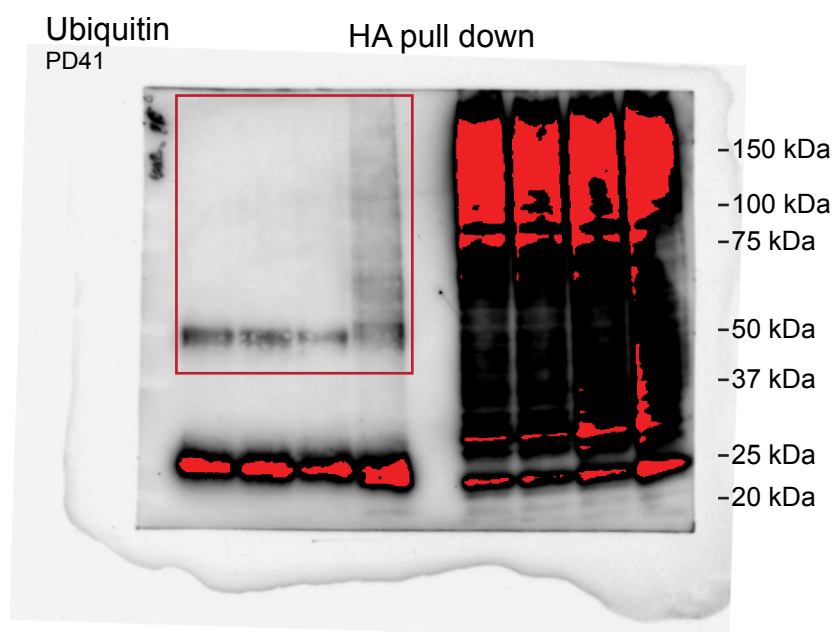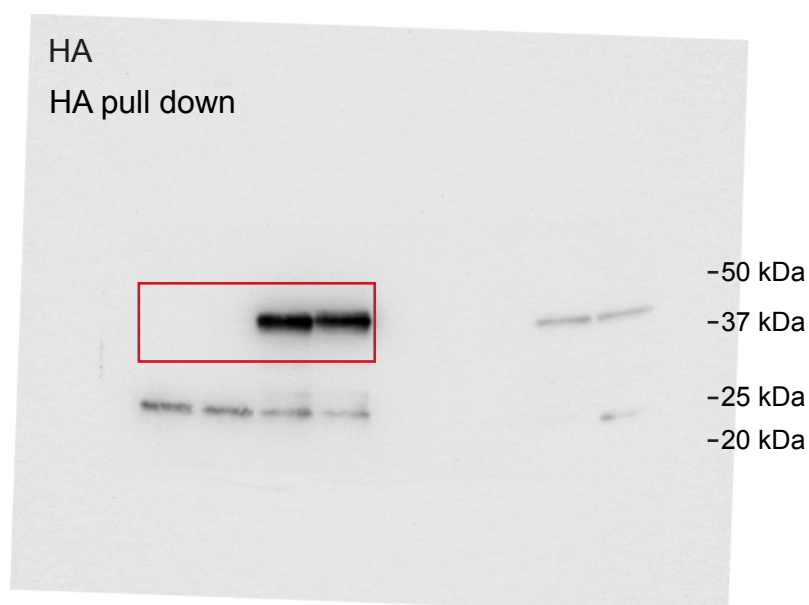

HRS  
Lysate

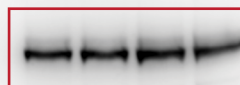

-150 kDa  
-100 kDa  
-75 kDa

STING  
D2P2F  
Lysate

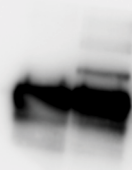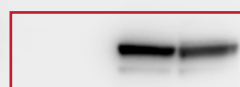

-50 kDa  
-37 kDa  
-25 kDa  
-20 kDa

HA  
Lysate

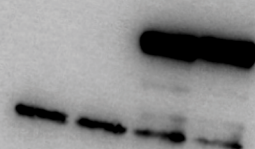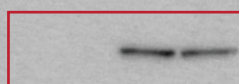

-50 kDa  
-37 kDa  
-25 kDa  
-20 kDa

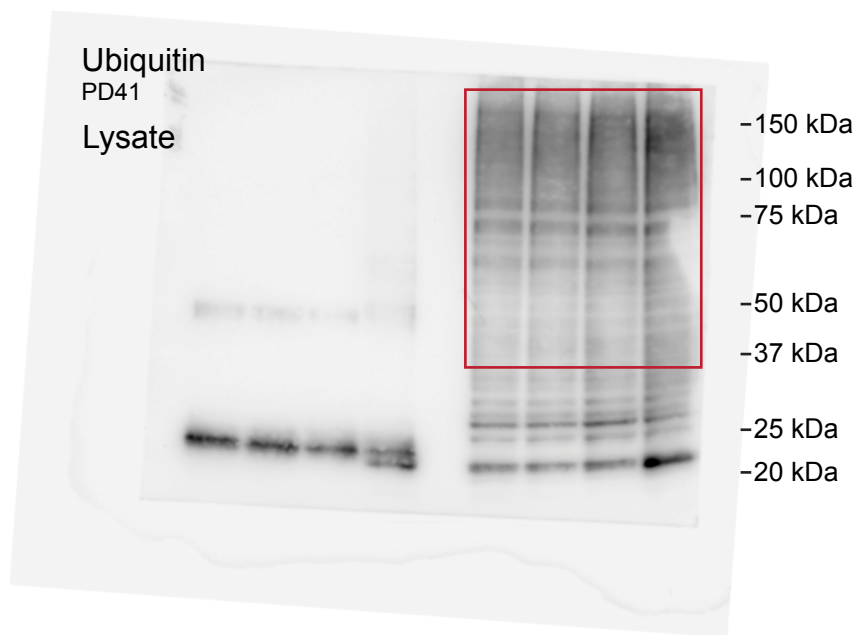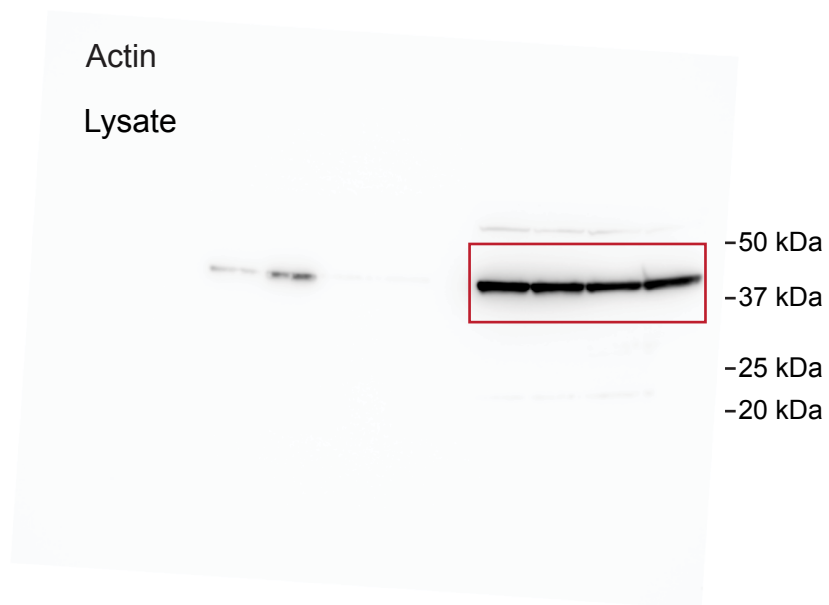

Supplement: Supplementary file 10 — Source Data for Expanded View and Appendix [file EMBJ-42-e112712-s001.zip › EV:S Figures/Figure EV4/Figure EV4B.pdf]

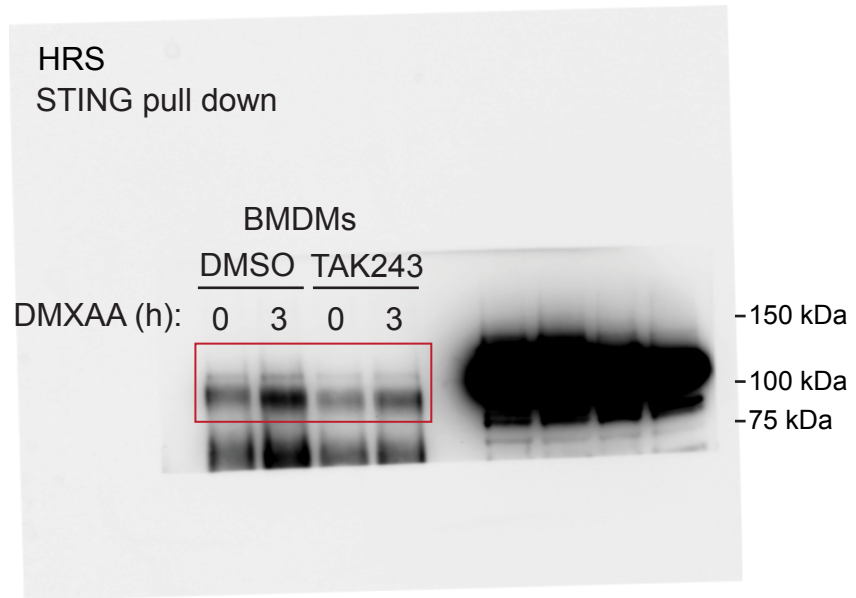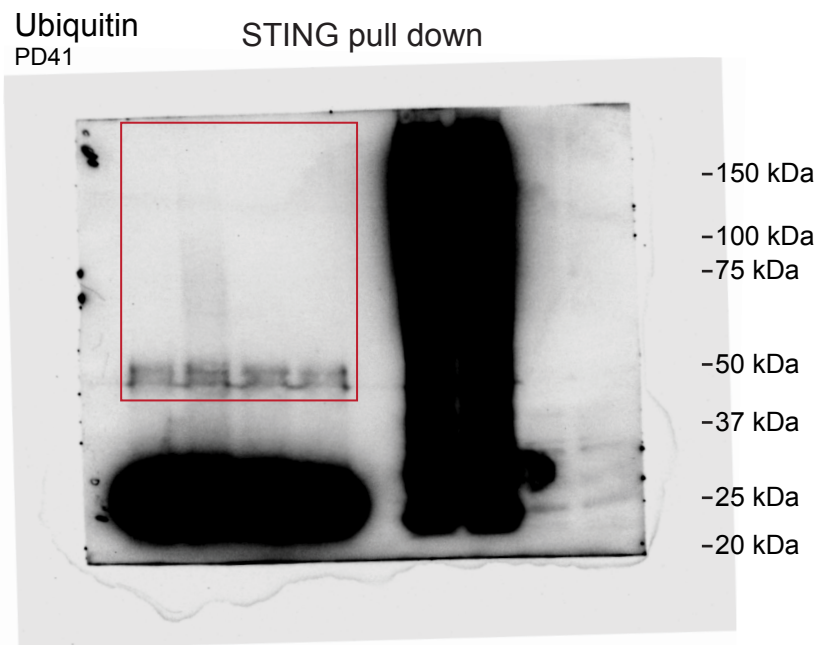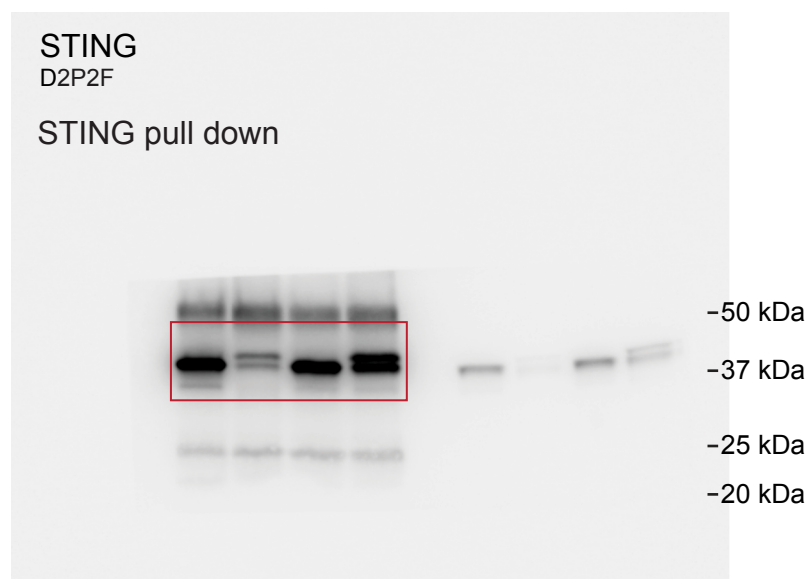

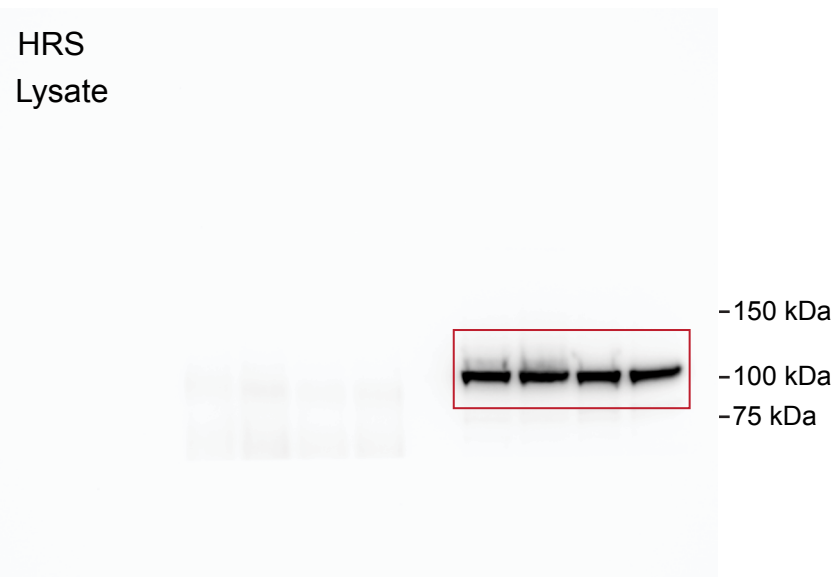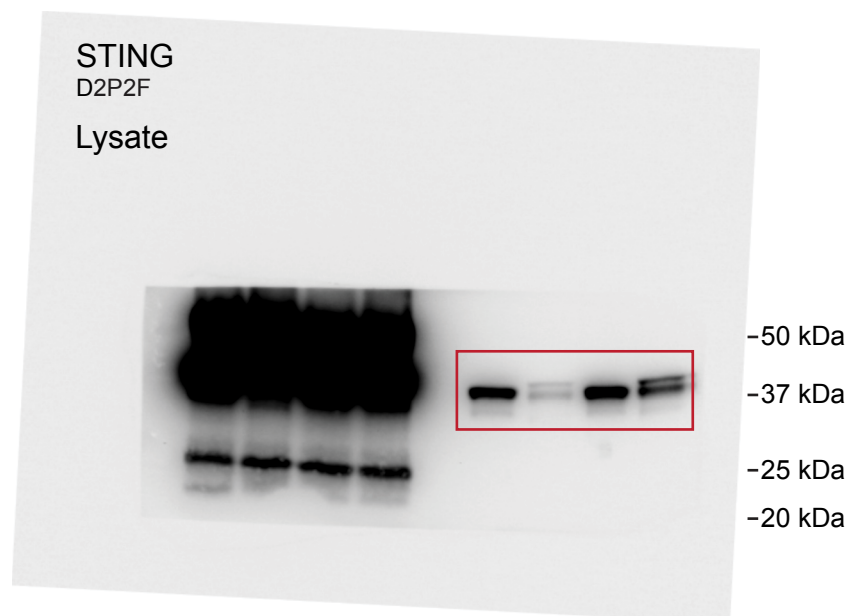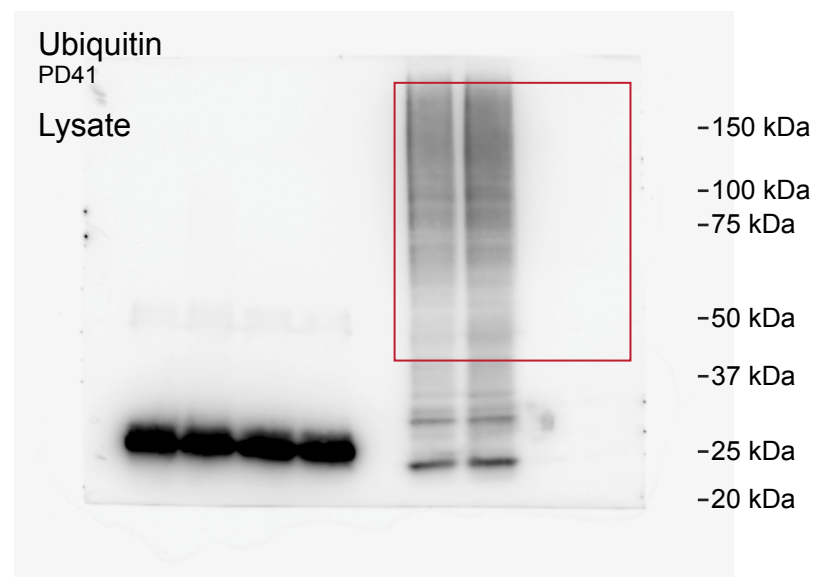

Actin  
Lysate

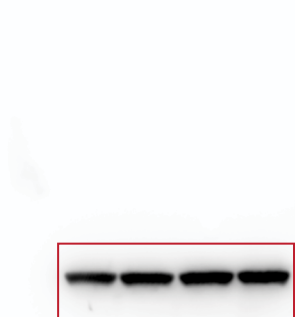

-150 kDa

-100 kDa

-75 kDa

-50 kDa

-37 kDa

-25 kDa

Supplement: Supplementary file 10 — Source Data for Expanded View and Appendix [file EMBJ-42-e112712-s001.zip › EV:S Figures/Figure EV4/Figure EV4C.pdf]

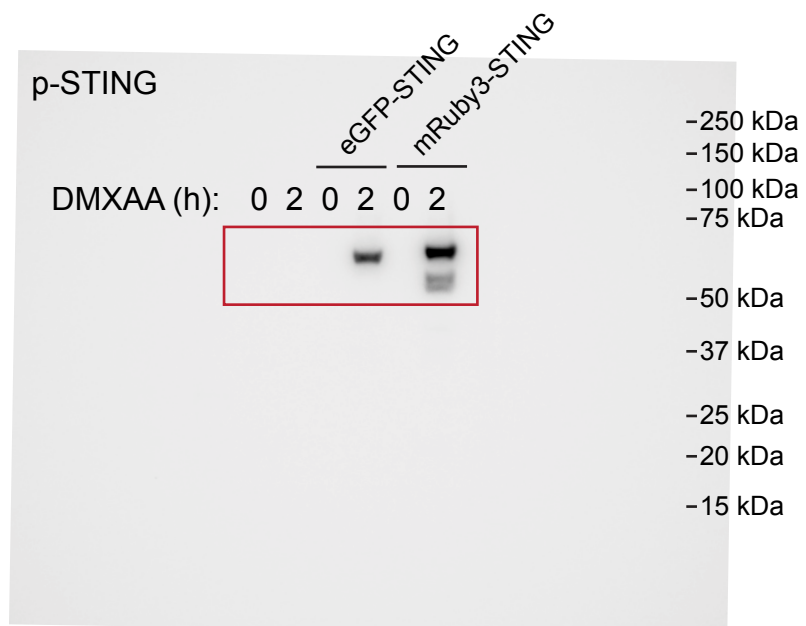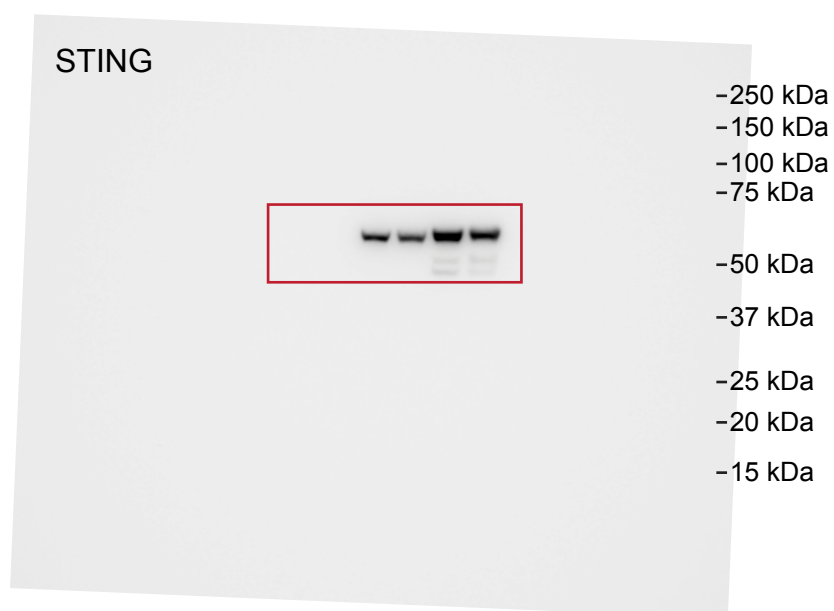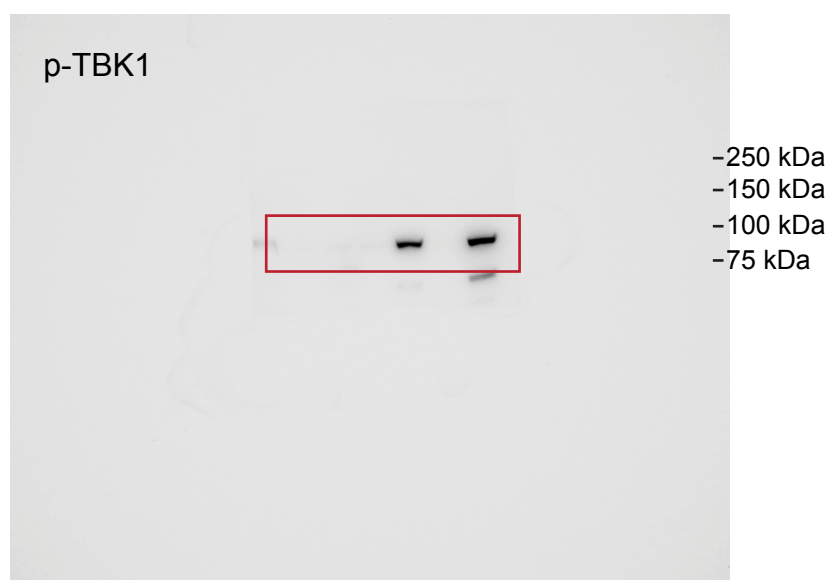

p-IRF3

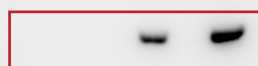

-50 kDa

-37 kDa

-25 kDa

-20 kDa

-15 kDa

Actin

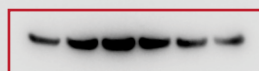

-250 kDa

-150 kDa

-100 kDa

-75 kDa

-50 kDa

-37 kDa

-25 kDa

-20 kDa

-15 kDa

Supplement: Supplementary file 10 — Source Data for Expanded View and Appendix [file EMBJ-42-e112712-s001.zip › EV:S Figures/Figure EV3/Figure EV3A.pdf]

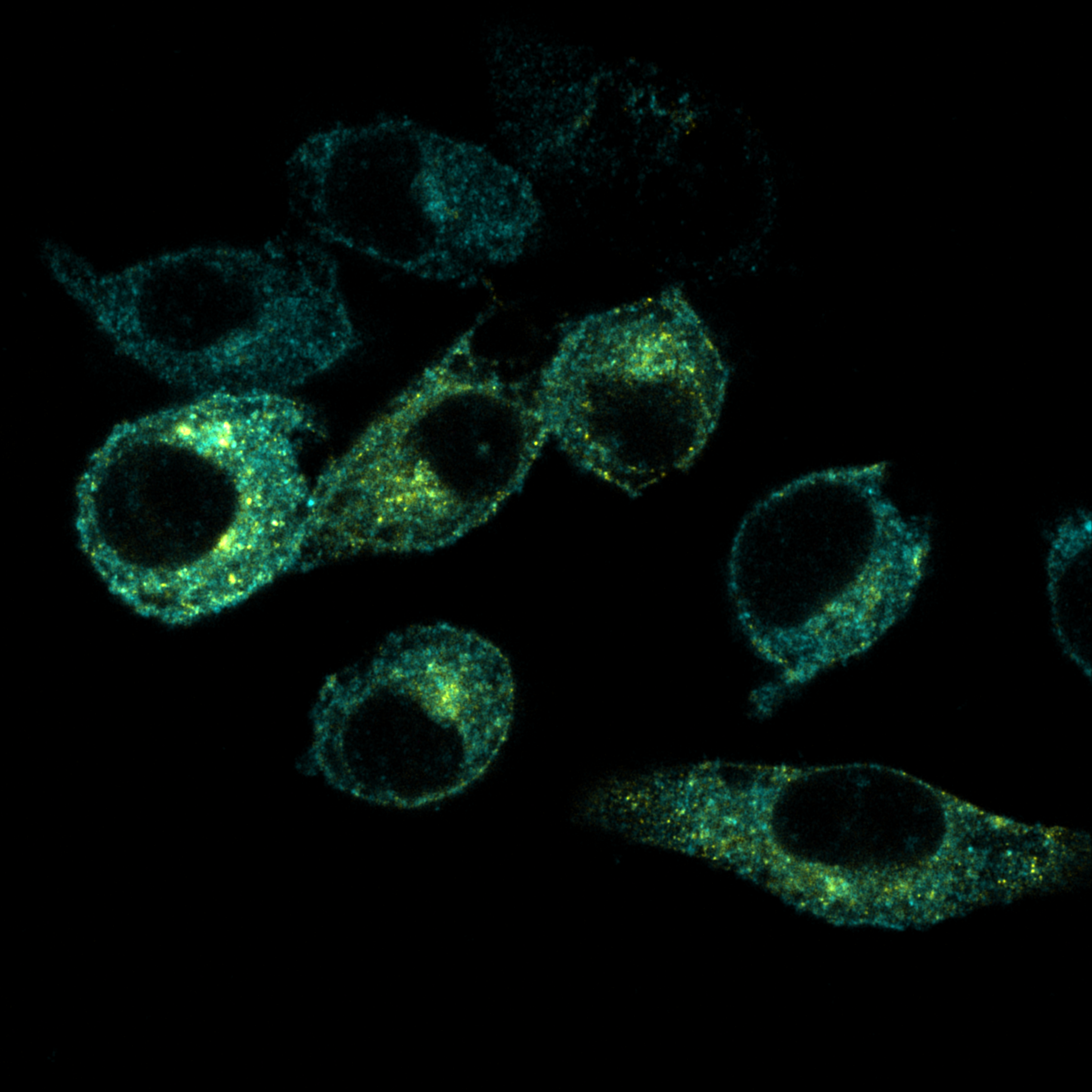

Supplement: Supplementary file 10 — Source Data for Expanded View and Appendix [file EMBJ-42-e112712-s001.zip › EV:S Figures/Appendix Figure S3 and S4/TAK243-DMXAA3h.tif]

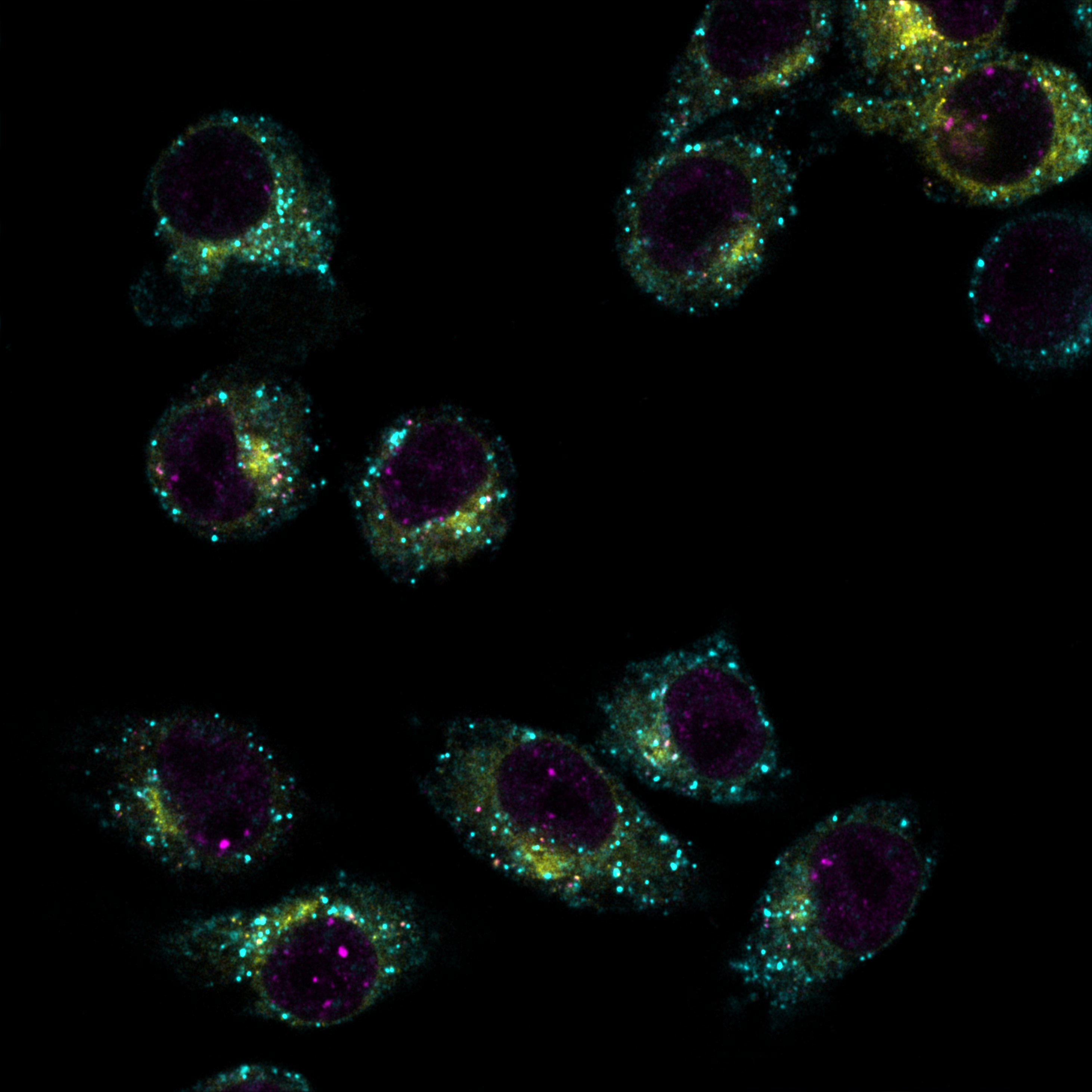

Supplement: Supplementary file 10 — Source Data for Expanded View and Appendix [file EMBJ-42-e112712-s001.zip › EV:S Figures/Appendix Figure S3 and S4/DMXAA30min.tif]

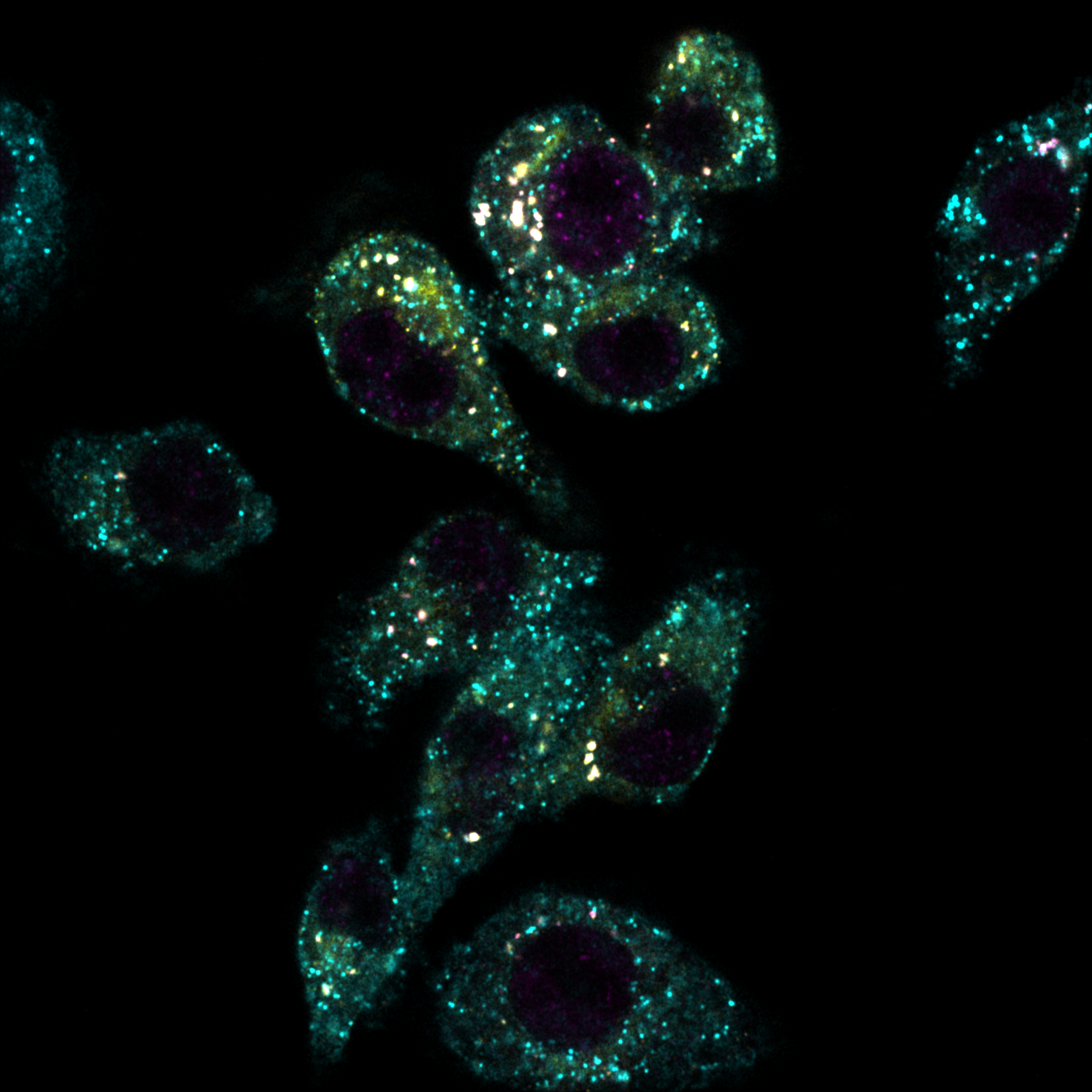

Supplement: Supplementary file 10 — Source Data for Expanded View and Appendix [file EMBJ-42-e112712-s001.zip › EV:S Figures/Appendix Figure S3 and S4/DMXAA3h.tif]

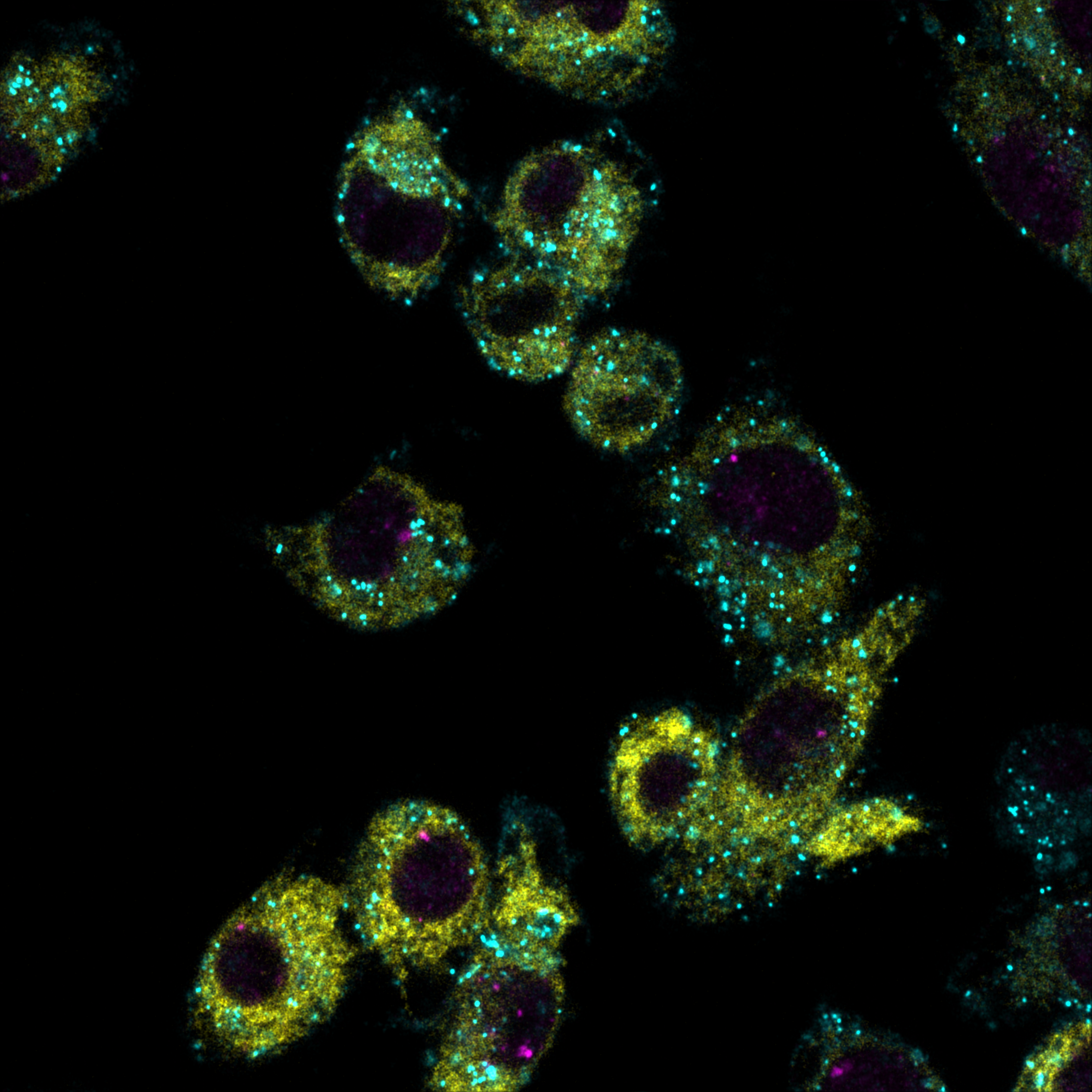

Supplement: Supplementary file 10 — Source Data for Expanded View and Appendix [file EMBJ-42-e112712-s001.zip › EV:S Figures/Appendix Figure S3 and S4/Untreated.tif]

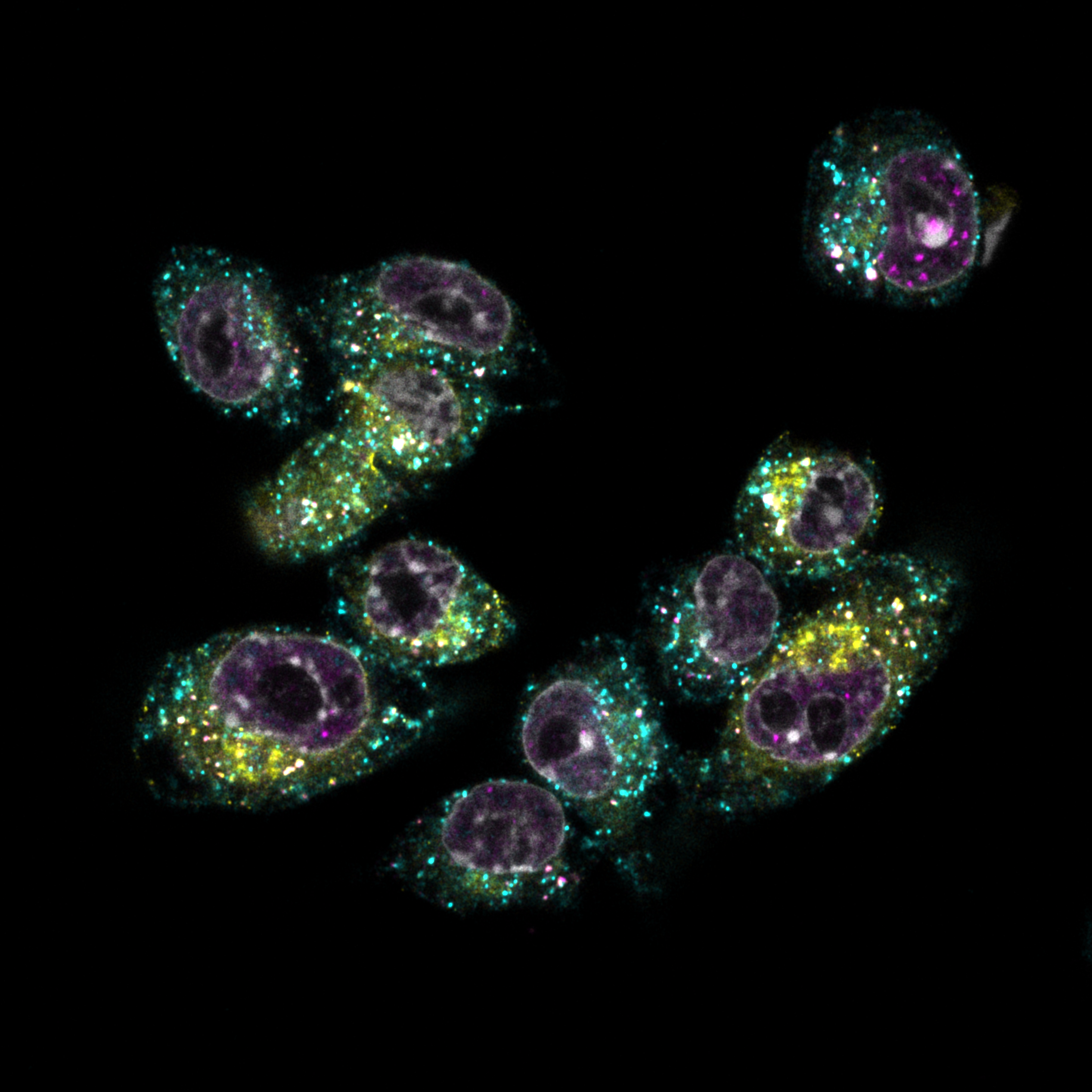

Supplement: Supplementary file 10 — Source Data for Expanded View and Appendix [file EMBJ-42-e112712-s001.zip › EV:S Figures/Appendix Figure S3 and S4/DMXAA90min.tif]

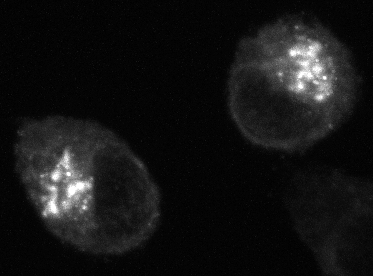

Supplement: Supplementary file 10 — Source Data for Expanded View and Appendix [file EMBJ-42-e112712-s001.zip › EV:S Figures/Figure EV3/Figure EV3D/MIP_FigureEV3D_36min.tif]

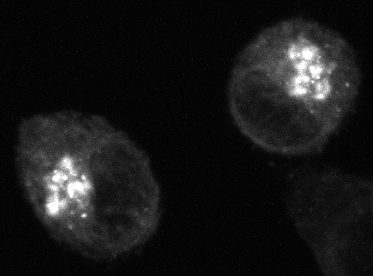

Supplement: Supplementary file 10 — Source Data for Expanded View and Appendix [file EMBJ-42-e112712-s001.zip › EV:S Figures/Figure EV3/Figure EV3D/MIP_FigureEV3D_34min.tif]

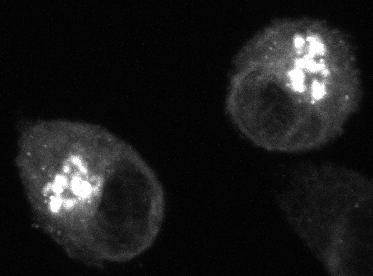

Supplement: Supplementary file 10 — Source Data for Expanded View and Appendix [file EMBJ-42-e112712-s001.zip › EV:S Figures/Figure EV3/Figure EV3D/MIP_FigureEV3D_30min.tif]

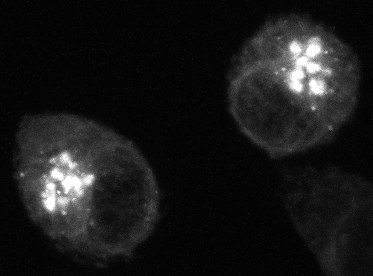

Supplement: Supplementary file 10 — Source Data for Expanded View and Appendix [file EMBJ-42-e112712-s001.zip › EV:S Figures/Figure EV3/Figure EV3D/MIP_FigureEV3D_32min.tif]

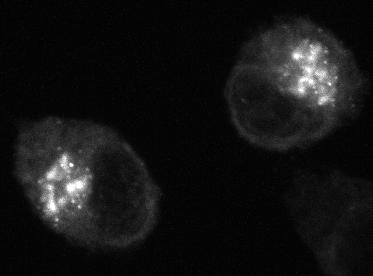

Supplement: Supplementary file 10 — Source Data for Expanded View and Appendix [file EMBJ-42-e112712-s001.zip › EV:S Figures/Figure EV3/Figure EV3D/MIP_FigureEV3D_37min.tif]

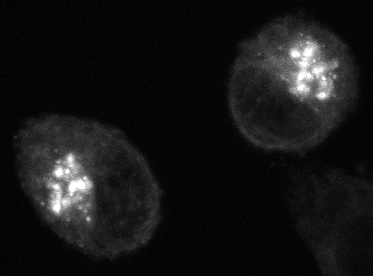

Supplement: Supplementary file 10 — Source Data for Expanded View and Appendix [file EMBJ-42-e112712-s001.zip › EV:S Figures/Figure EV3/Figure EV3D/MIP_FigureEV3D_35min.tif]

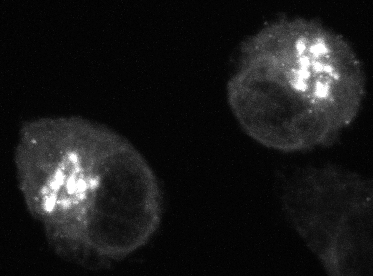

Supplement: Supplementary file 10 — Source Data for Expanded View and Appendix [file EMBJ-42-e112712-s001.zip › EV:S Figures/Figure EV3/Figure EV3D/MIP_FigureEV3D_31min.tif]

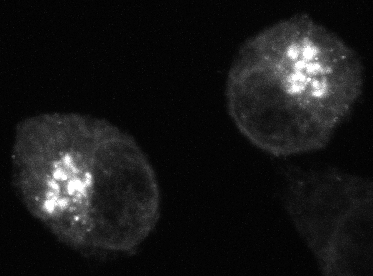

Supplement: Supplementary file 10 — Source Data for Expanded View and Appendix [file EMBJ-42-e112712-s001.zip › EV:S Figures/Figure EV3/Figure EV3D/MIP_FigureEV3D_33min.tif]

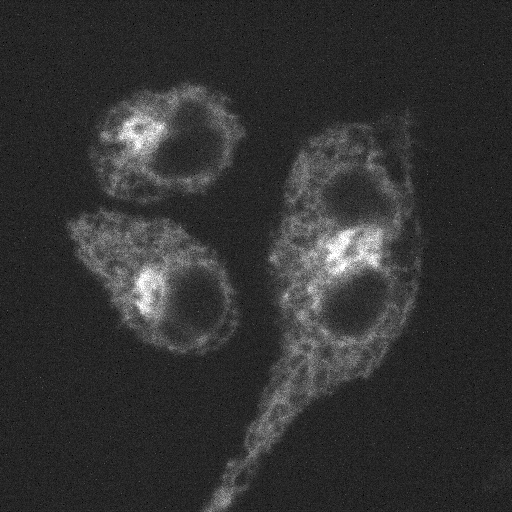

Supplement: Supplementary file 10 — Source Data for Expanded View and Appendix [file EMBJ-42-e112712-s001.zip › EV:S Figures/Figure EV3/Figure EV3C/FigureEV3C_10min.tif]

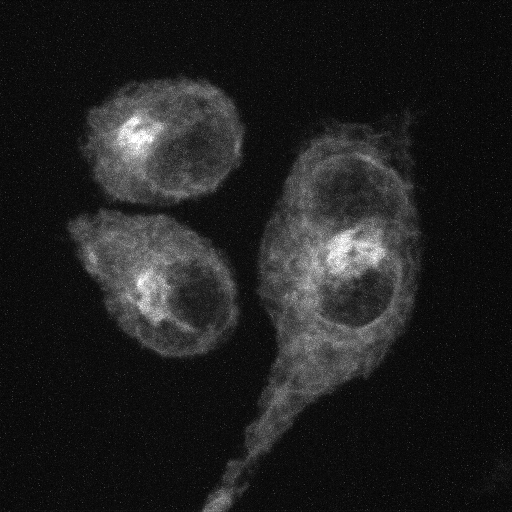

Supplement: Supplementary file 10 — Source Data for Expanded View and Appendix [file EMBJ-42-e112712-s001.zip › EV:S Figures/Figure EV3/Figure EV3C/MIP_FigureEV3C_10min.tif]

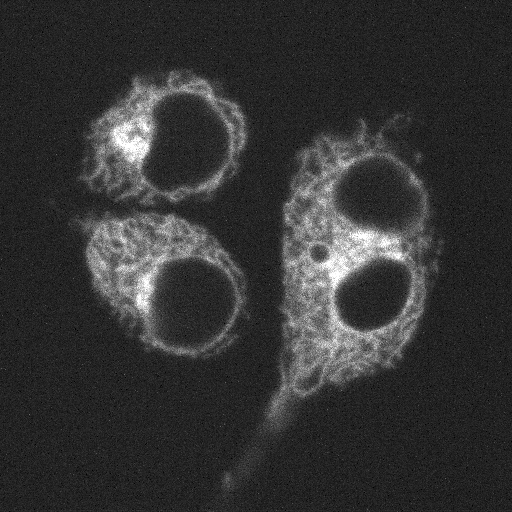

Supplement: Supplementary file 10 — Source Data for Expanded View and Appendix [file EMBJ-42-e112712-s001.zip › EV:S Figures/Figure EV3/Figure EV3C/FigureEV3C_5min40sec.tif]

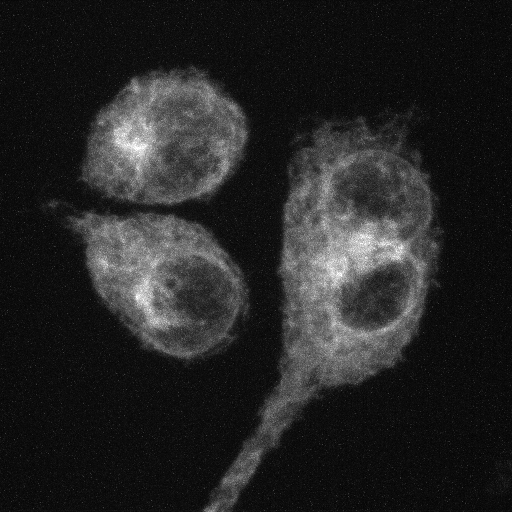

Supplement: Supplementary file 10 — Source Data for Expanded View and Appendix [file EMBJ-42-e112712-s001.zip › EV:S Figures/Figure EV3/Figure EV3C/MIP_FigureEV3C_5min40sec.tif]

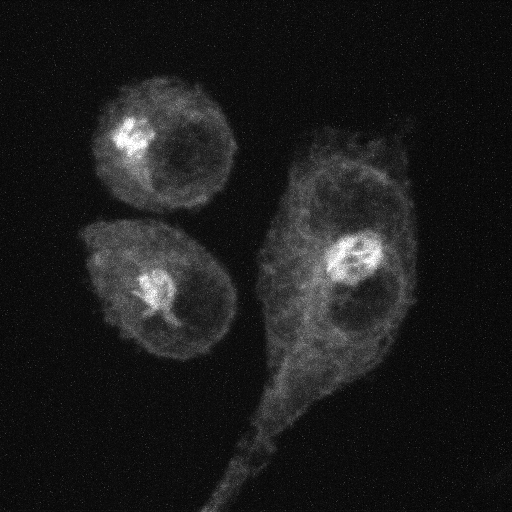

Supplement: Supplementary file 10 — Source Data for Expanded View and Appendix [file EMBJ-42-e112712-s001.zip › EV:S Figures/Figure EV3/Figure EV3C/MIP_FigureEV3C_15min.tif]

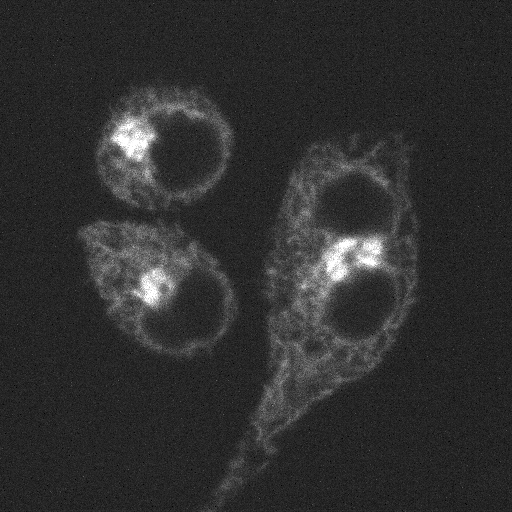

Supplement: Supplementary file 10 — Source Data for Expanded View and Appendix [file EMBJ-42-e112712-s001.zip › EV:S Figures/Figure EV3/Figure EV3C/FigureEV3C_15min.tif]

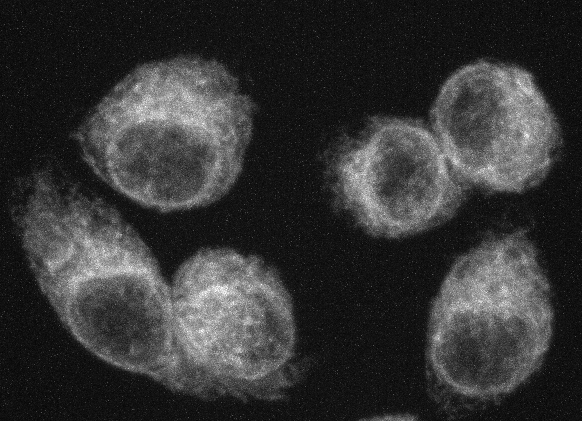

Supplement: Supplementary file 10 — Source Data for Expanded View and Appendix [file EMBJ-42-e112712-s001.zip › EV:S Figures/Figure EV3/Figure EV3B/MIP_FigureEV3B.tif]

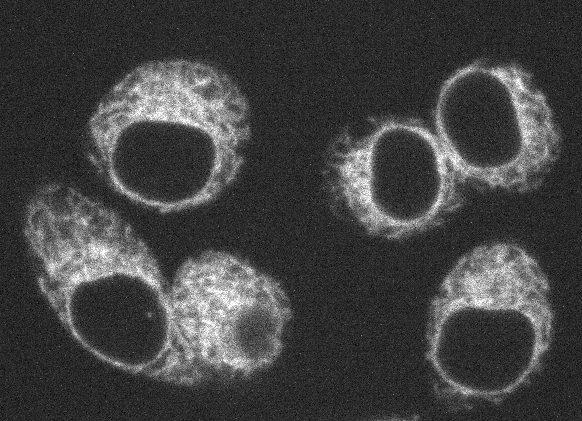

Supplement: Supplementary file 10 — Source Data for Expanded View and Appendix [file EMBJ-42-e112712-s001.zip › EV:S Figures/Figure EV3/Figure EV3B/FigureEV3B.tif]

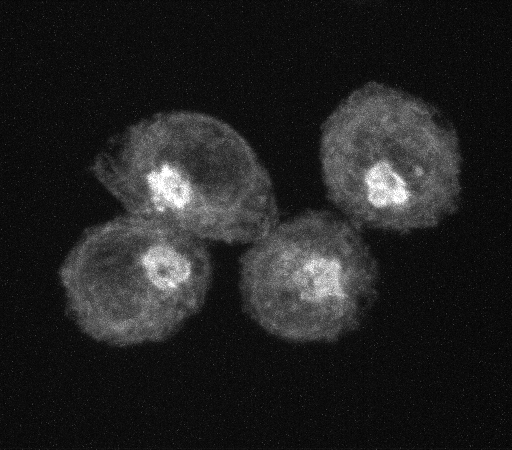

Supplement: Supplementary file 10 — Source Data for Expanded View and Appendix [file EMBJ-42-e112712-s001.zip › EV:S Figures/Appendix Figure S2/Appendix Figure S2A/MIP_FigureS2A_7min.tif]

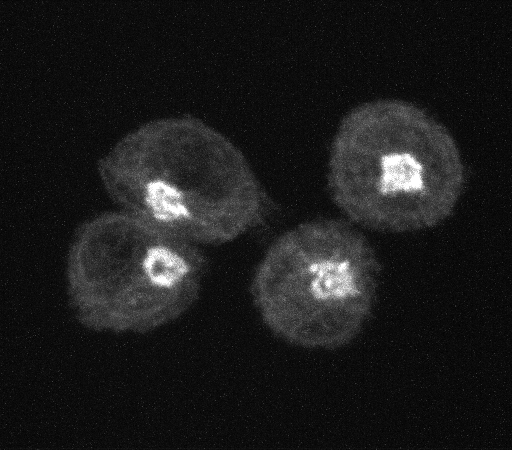

Supplement: Supplementary file 10 — Source Data for Expanded View and Appendix [file EMBJ-42-e112712-s001.zip › EV:S Figures/Appendix Figure S2/Appendix Figure S2A/MIP_FigureS2A_16min.tif]

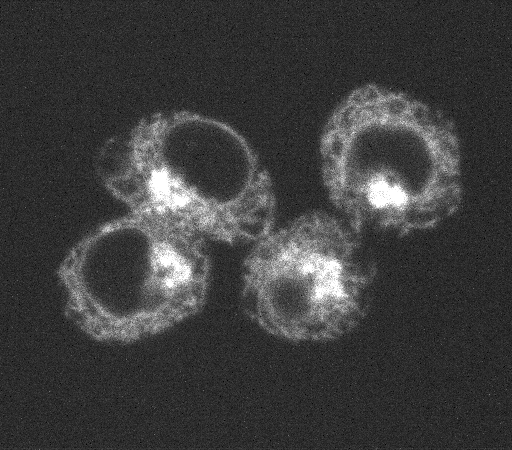

Supplement: Supplementary file 10 — Source Data for Expanded View and Appendix [file EMBJ-42-e112712-s001.zip › EV:S Figures/Appendix Figure S2/Appendix Figure S2A/FigureS2A_7min.tif]

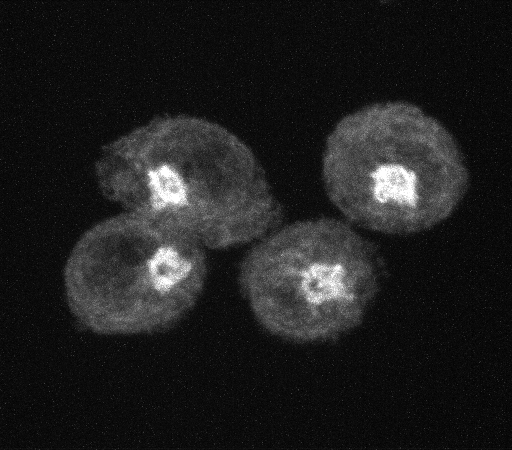

Supplement: Supplementary file 10 — Source Data for Expanded View and Appendix [file EMBJ-42-e112712-s001.zip › EV:S Figures/Appendix Figure S2/Appendix Figure S2A/MIP_FigureS2A_10min.tif]

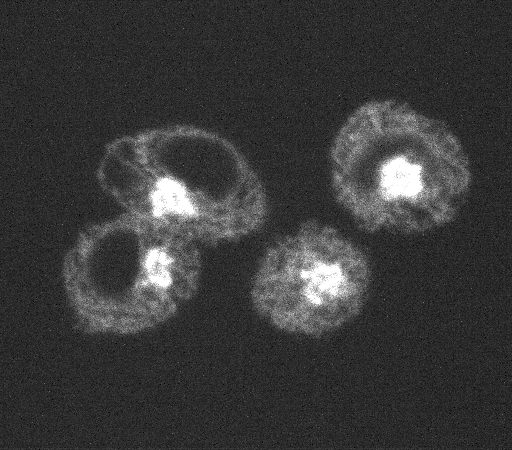

Supplement: Supplementary file 10 — Source Data for Expanded View and Appendix [file EMBJ-42-e112712-s001.zip › EV:S Figures/Appendix Figure S2/Appendix Figure S2A/FigureS2A_13min.tif]

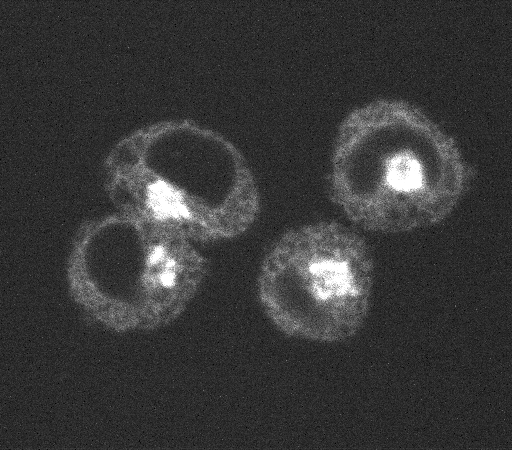

Supplement: Supplementary file 10 — Source Data for Expanded View and Appendix [file EMBJ-42-e112712-s001.zip › EV:S Figures/Appendix Figure S2/Appendix Figure S2A/FigureS2A_16min.tif]

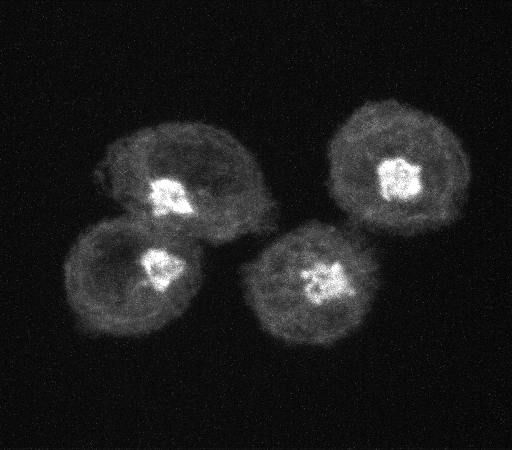

Supplement: Supplementary file 10 — Source Data for Expanded View and Appendix [file EMBJ-42-e112712-s001.zip › EV:S Figures/Appendix Figure S2/Appendix Figure S2A/MIP_FigureS2A_13min.tif]

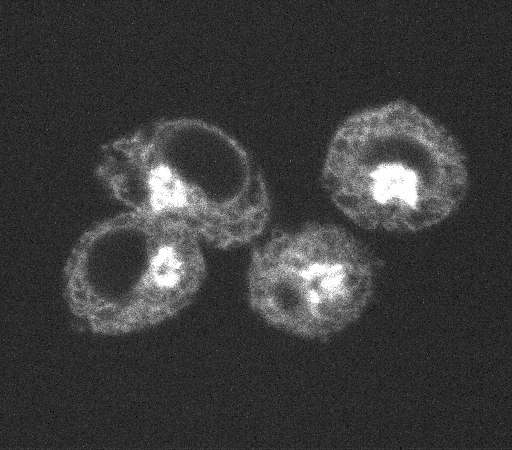

Supplement: Supplementary file 10 — Source Data for Expanded View and Appendix [file EMBJ-42-e112712-s001.zip › EV:S Figures/Appendix Figure S2/Appendix Figure S2A/FigureS2A_10min.tif]

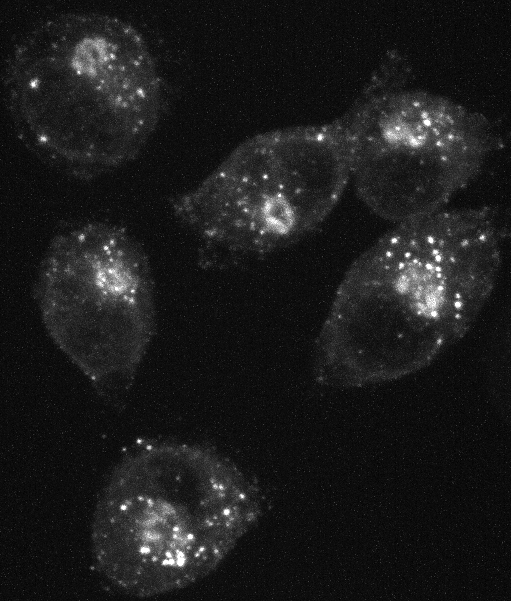

Supplement: Supplementary file 10 — Source Data for Expanded View and Appendix [file EMBJ-42-e112712-s001.zip › EV:S Figures/Appendix Figure S2/Appendix Figure S2B/MIP_FigureS2B_1-18-30.tif]

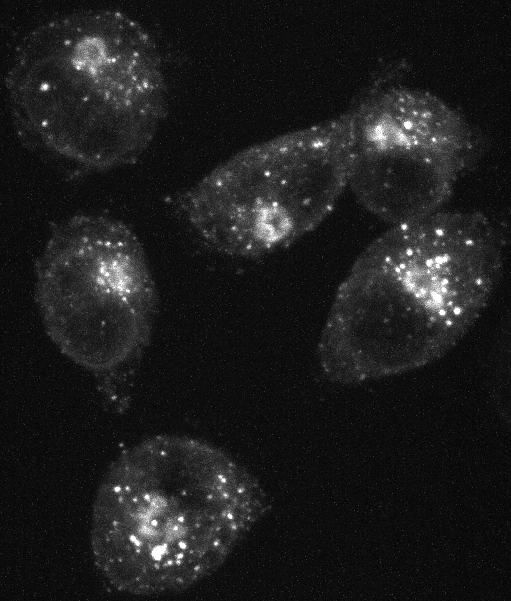

Supplement: Supplementary file 10 — Source Data for Expanded View and Appendix [file EMBJ-42-e112712-s001.zip › EV:S Figures/Appendix Figure S2/Appendix Figure S2B/MIP_FigureS2B_1-16-30.tif]

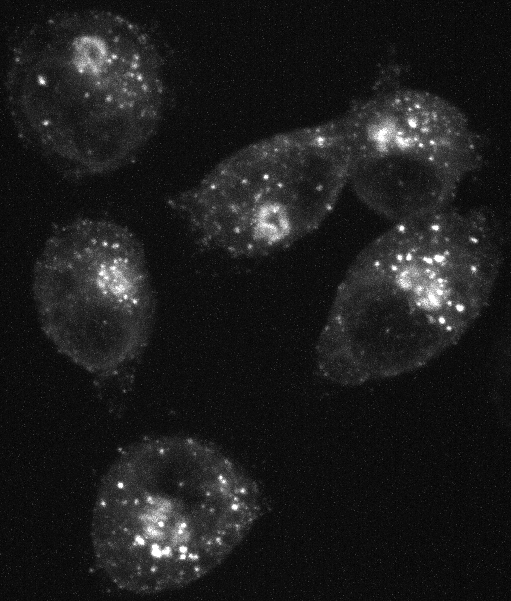

Supplement: Supplementary file 10 — Source Data for Expanded View and Appendix [file EMBJ-42-e112712-s001.zip › EV:S Figures/Appendix Figure S2/Appendix Figure S2B/MIP_FigureS2B_1-17-00.tif]

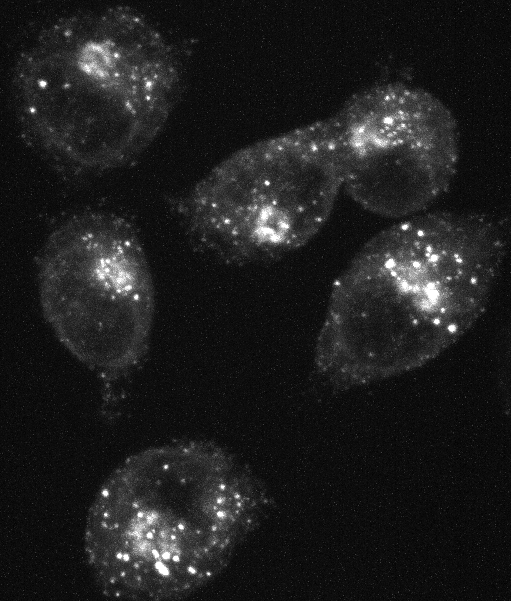

Supplement: Supplementary file 10 — Source Data for Expanded View and Appendix [file EMBJ-42-e112712-s001.zip › EV:S Figures/Appendix Figure S2/Appendix Figure S2B/MIP_FigureS2B_1-15-00.tif]

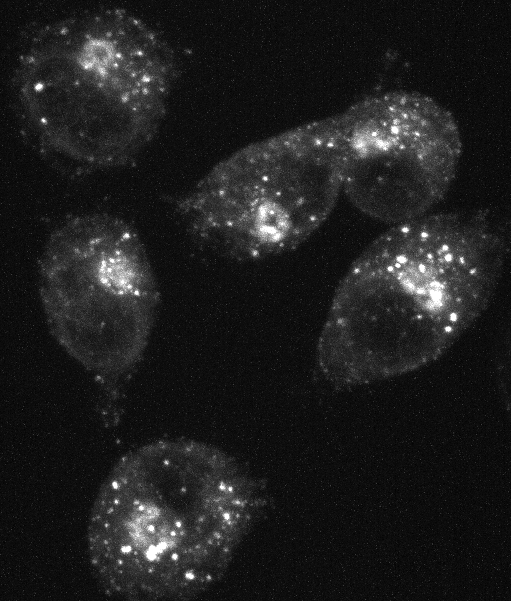

Supplement: Supplementary file 10 — Source Data for Expanded View and Appendix [file EMBJ-42-e112712-s001.zip › EV:S Figures/Appendix Figure S2/Appendix Figure S2B/MIP_FigureS2B_1-15-30.tif]

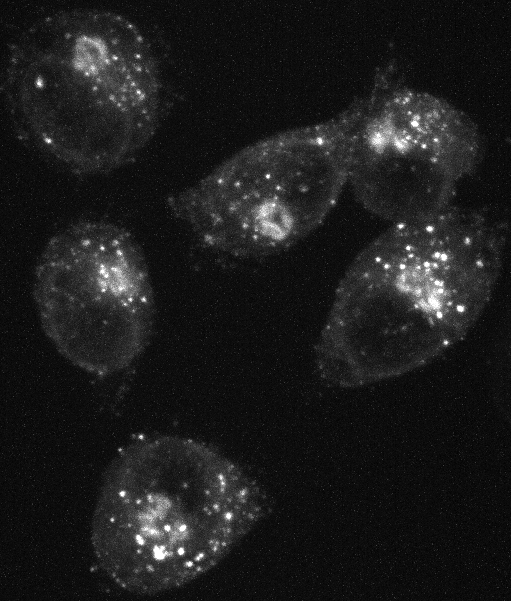

Supplement: Supplementary file 10 — Source Data for Expanded View and Appendix [file EMBJ-42-e112712-s001.zip › EV:S Figures/Appendix Figure S2/Appendix Figure S2B/MIP_FigureS2B_1-17-30.tif]

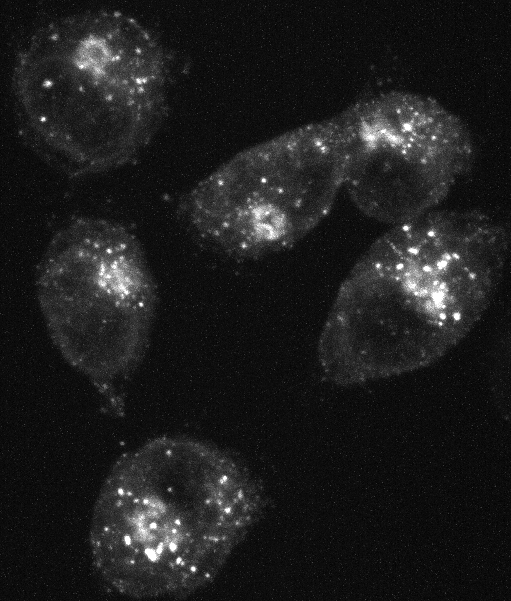

Supplement: Supplementary file 10 — Source Data for Expanded View and Appendix [file EMBJ-42-e112712-s001.zip › EV:S Figures/Appendix Figure S2/Appendix Figure S2B/MIP_FigureS2B_1-16-00.tif]

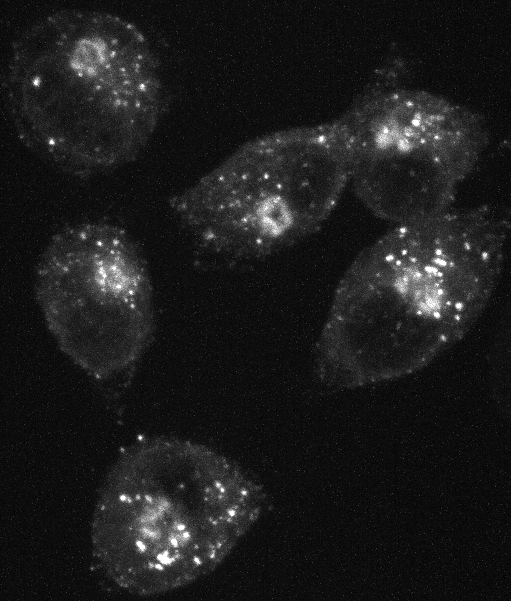

Supplement: Supplementary file 10 — Source Data for Expanded View and Appendix [file EMBJ-42-e112712-s001.zip › EV:S Figures/Appendix Figure S2/Appendix Figure S2B/MIP_FigureS2B_1-18-00.tif]

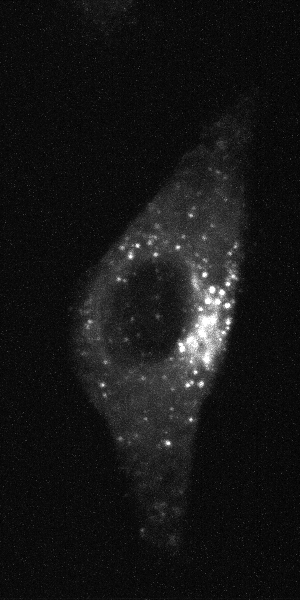

Supplement: Supplementary file 12 — Source Data for Figure 2 [file EMBJ-42-e112712-s008.zip › Figure 2/Figure 2F.tif]

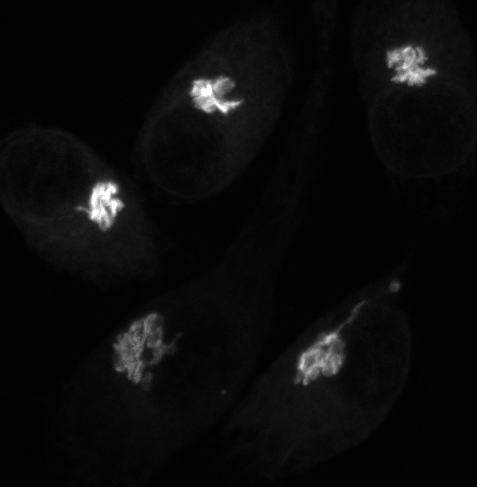

Supplement: Supplementary file 12 — Source Data for Figure 2 [file EMBJ-42-e112712-s008.zip › Figure 2/Figure 2A/UT.tif]

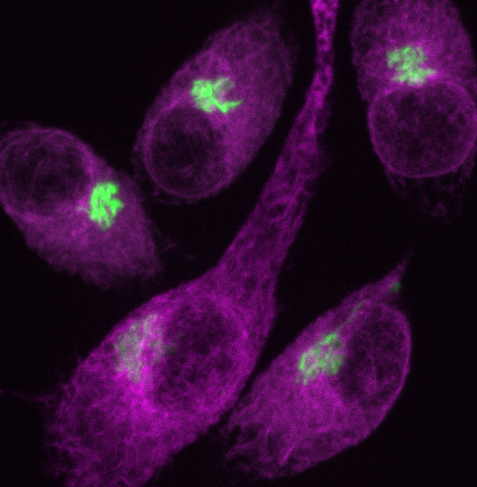

Supplement: Supplementary file 12 — Source Data for Figure 2 [file EMBJ-42-e112712-s008.zip › Figure 2/Figure 2A/UT-composite.tif]

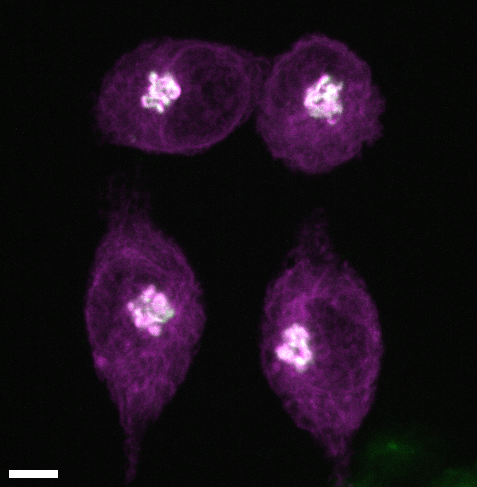

Supplement: Supplementary file 12 — Source Data for Figure 2 [file EMBJ-42-e112712-s008.zip › Figure 2/Figure 2A/DMXAA12min-composite.tif]

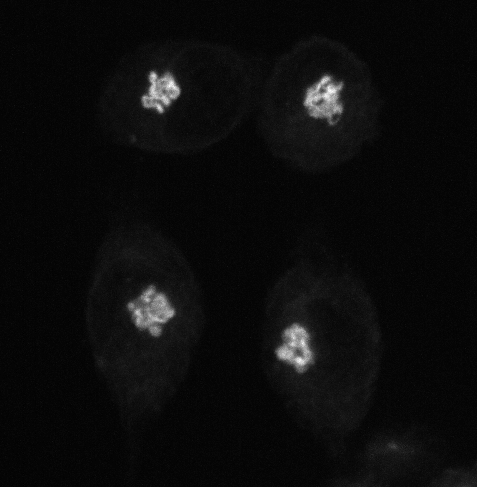

Supplement: Supplementary file 12 — Source Data for Figure 2 [file EMBJ-42-e112712-s008.zip › Figure 2/Figure 2A/DMXAA12min.tif]

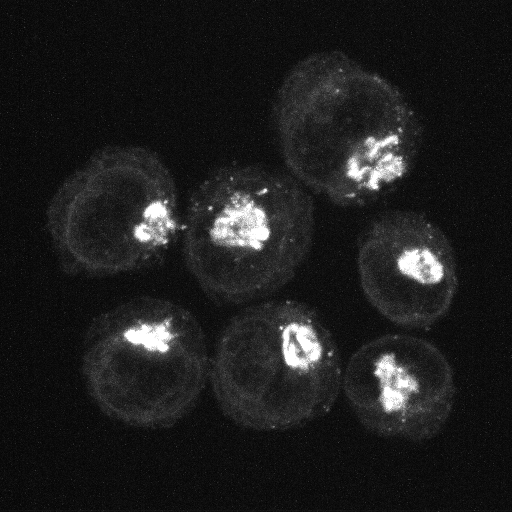

Supplement: Supplementary file 12 — Source Data for Figure 2 [file EMBJ-42-e112712-s008.zip › Figure 2/Figure 2D/MIP_Figure2D_33min.tif]

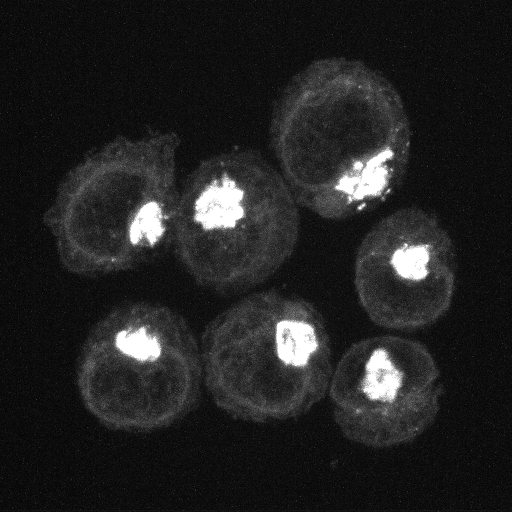

Supplement: Supplementary file 12 — Source Data for Figure 2 [file EMBJ-42-e112712-s008.zip › Figure 2/Figure 2D/MIP_Figure2D_27min.tif]

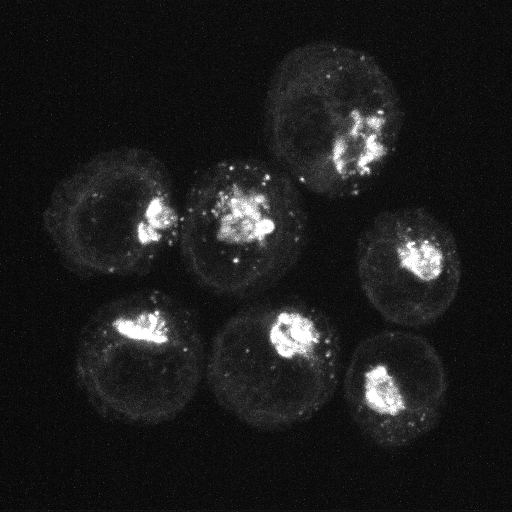

Supplement: Supplementary file 12 — Source Data for Figure 2 [file EMBJ-42-e112712-s008.zip › Figure 2/Figure 2D/MIP_Figure2D_39min.tif]

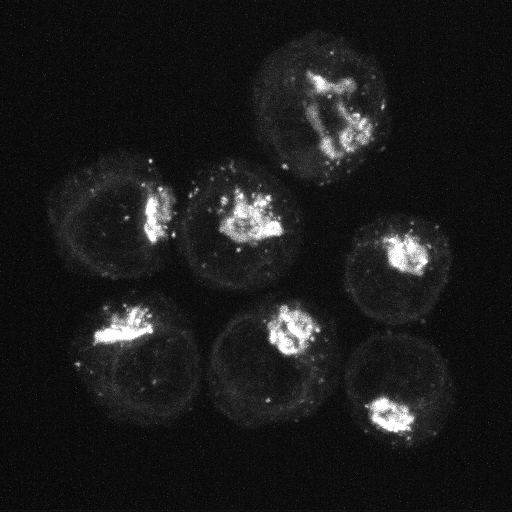

Supplement: Supplementary file 12 — Source Data for Figure 2 [file EMBJ-42-e112712-s008.zip › Figure 2/Figure 2D/MIP_Figure2D_45min.tif]

# LC3B

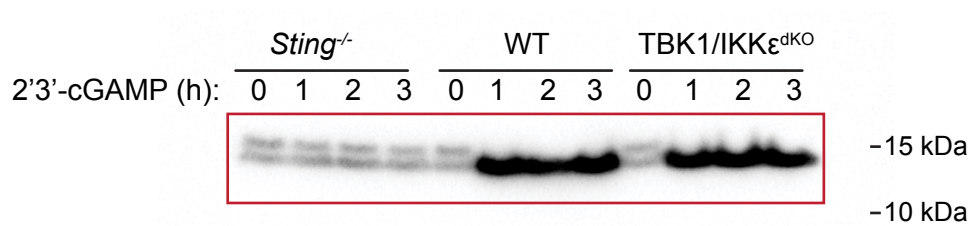

## TBK1

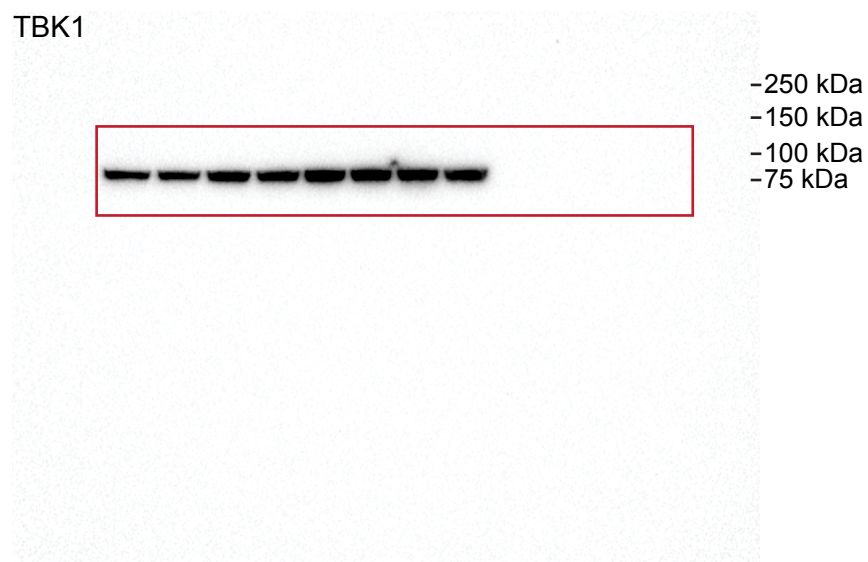

## IKKε

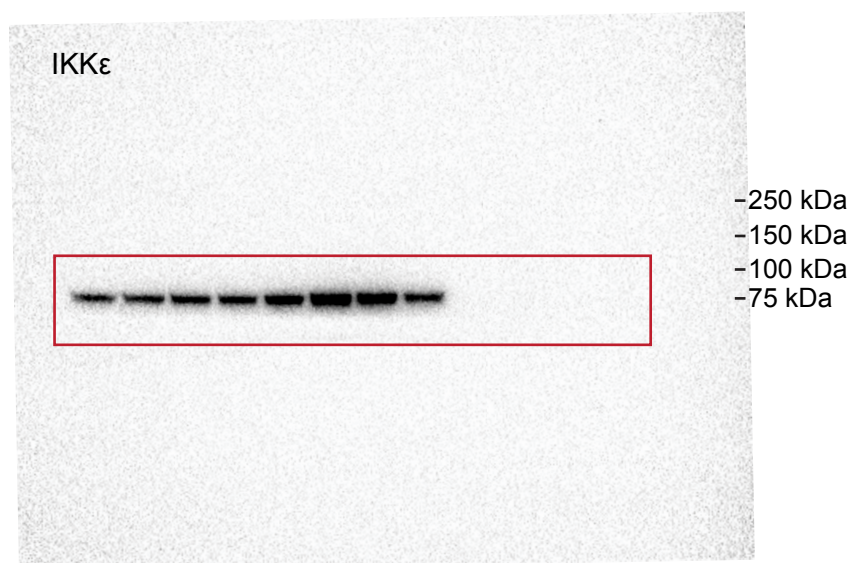

p-STING

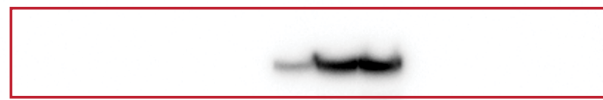

-50 kDa

-37 kDa

-25 kDa

STING

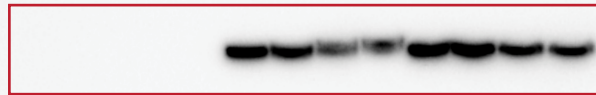

-50 kDa

-37 kDa

-25 kDa

-20 kDa

Actin

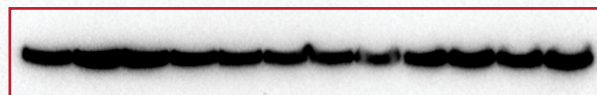

-50 kDa

-37 kDa

-25 kDa

Supplement: Supplementary file 13 — Source Data for Figure 3 [file EMBJ-42-e112712-s005.zip › Figure 3/Figure 3D.pdf]

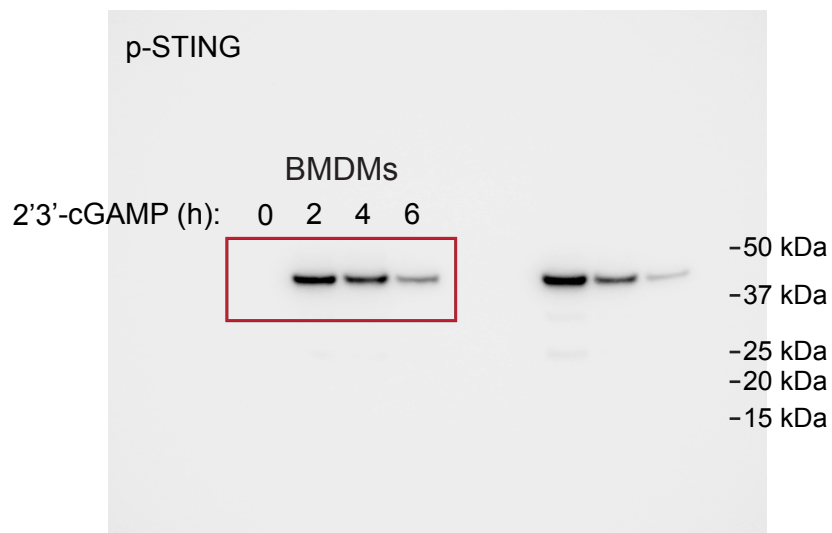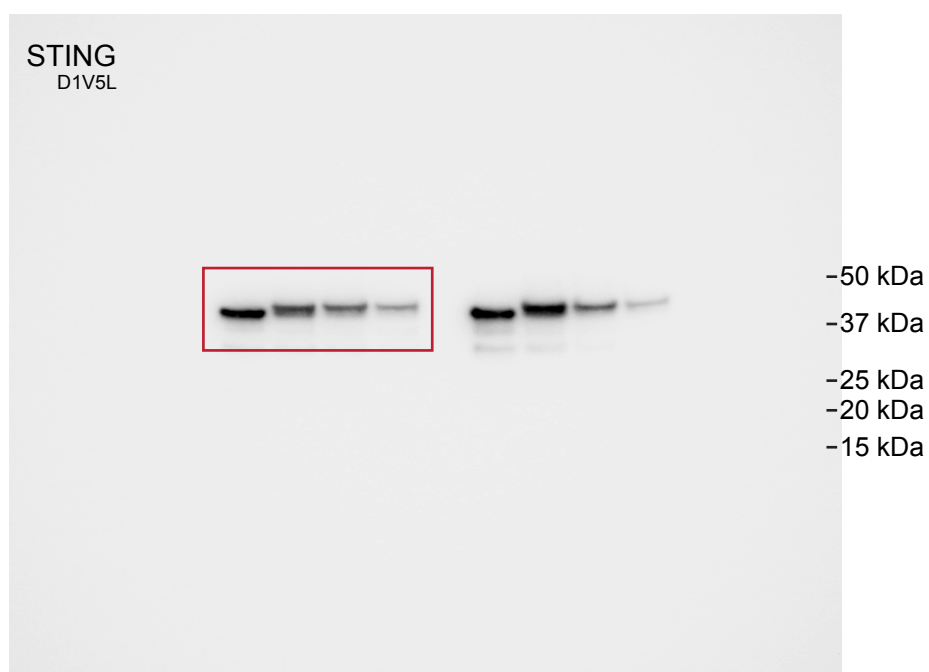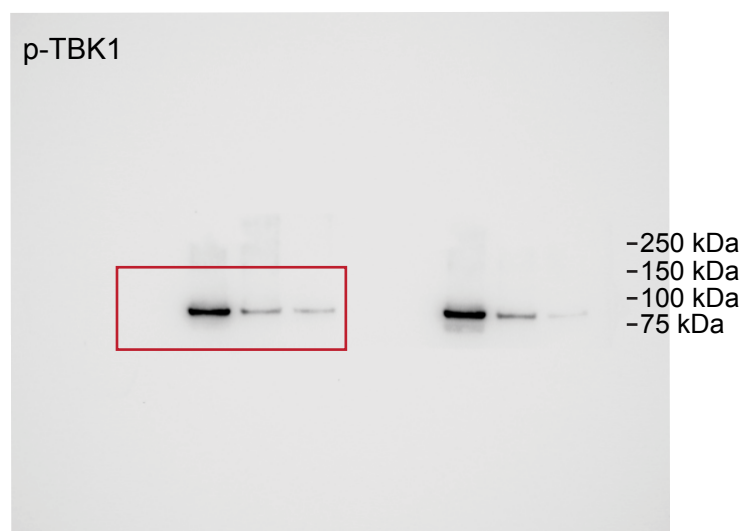

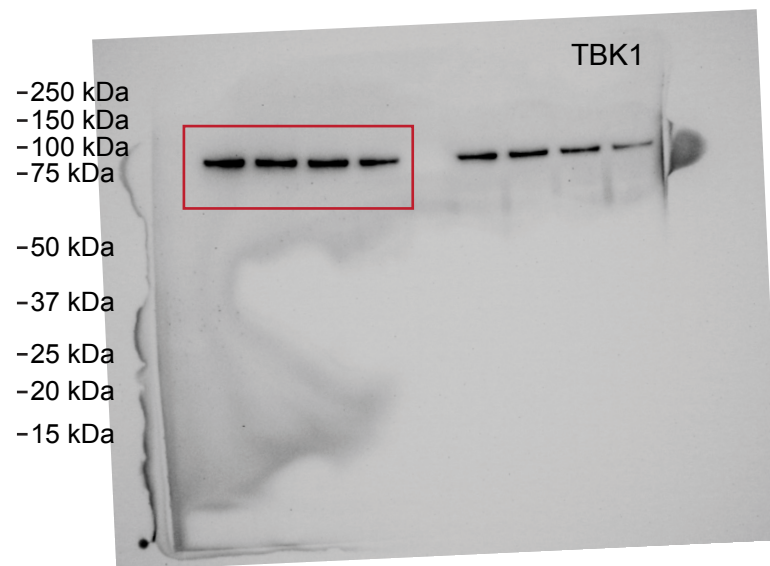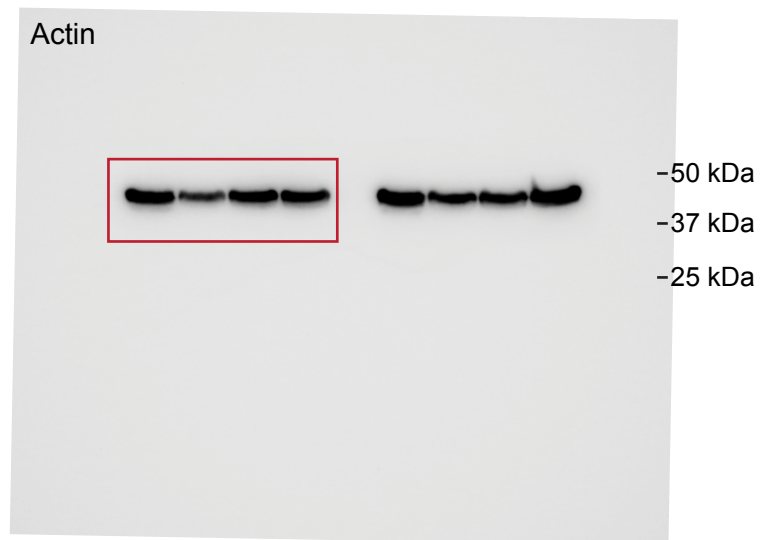

Supplement: Supplementary file 13 — Source Data for Figure 3 [file EMBJ-42-e112712-s005.zip › Figure 3/Figure 3B.pdf]

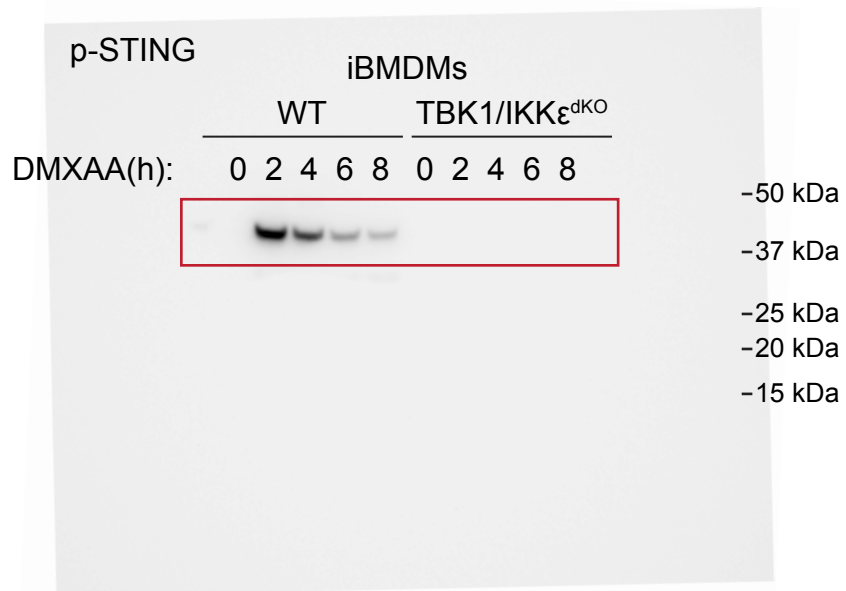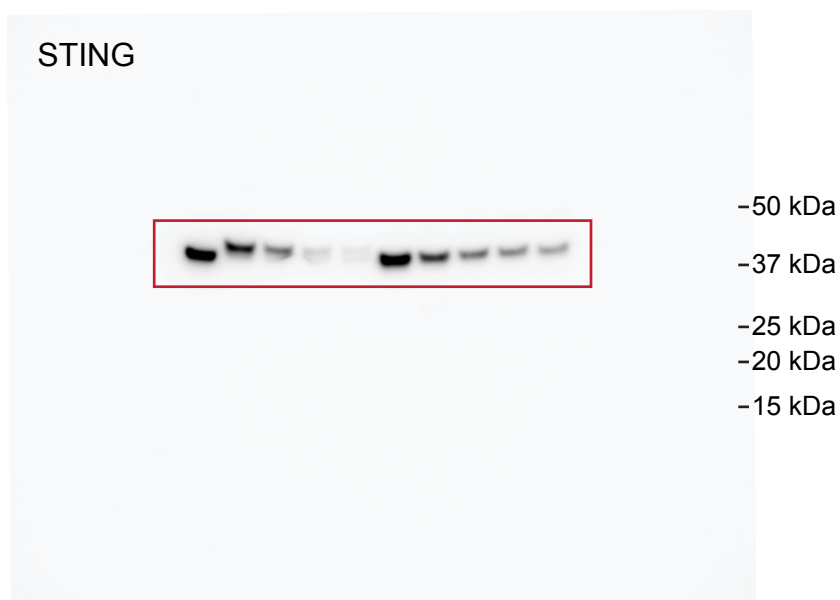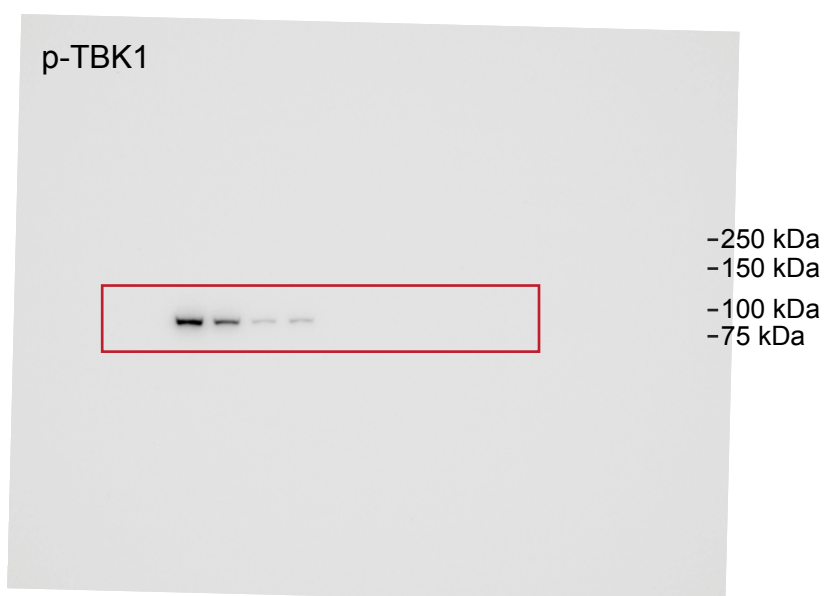

TBK1

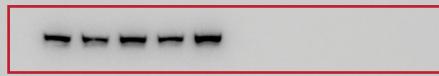

-250 kDa  
-150 kDa  
-100 kDa  
-75 kDa

IKK $\epsilon$

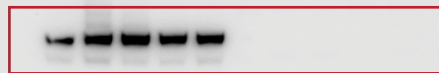

-250 kDa  
-150 kDa  
-100 kDa  
-75 kDa

Actin

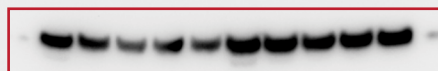

-50 kDa  
-37 kDa  
-25 kDa  
-20 kDa  
-15 kDa

Supplement: Supplementary file 13 — Source Data for Figure 3 [file EMBJ-42-e112712-s005.zip › Figure 3/Figure 3C.pdf]

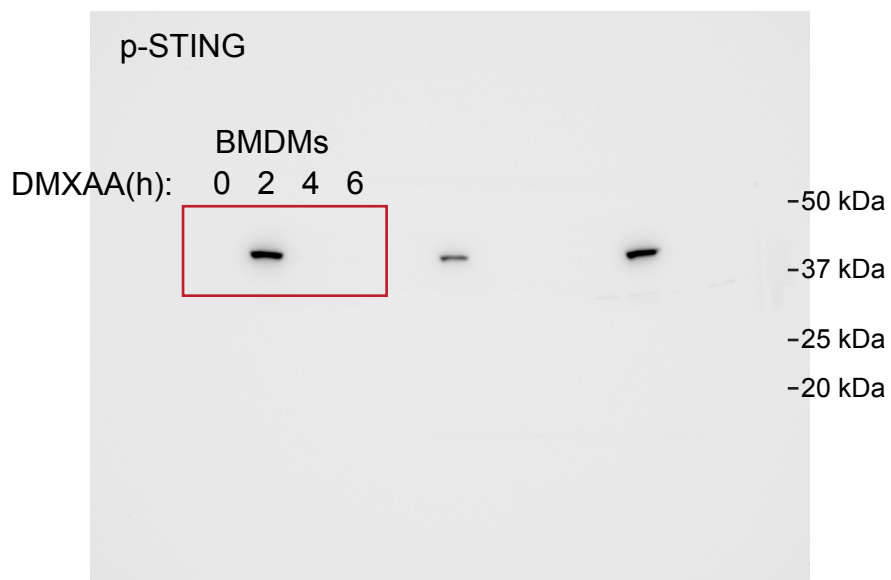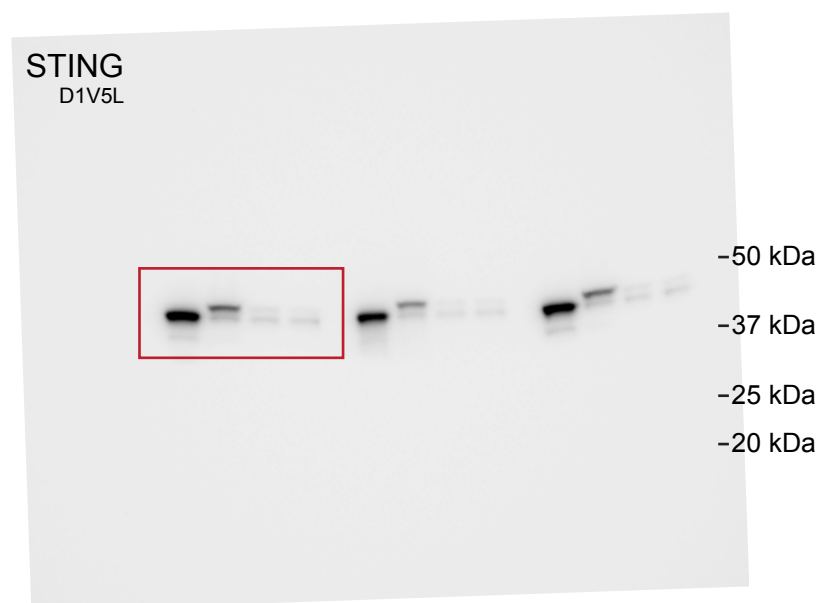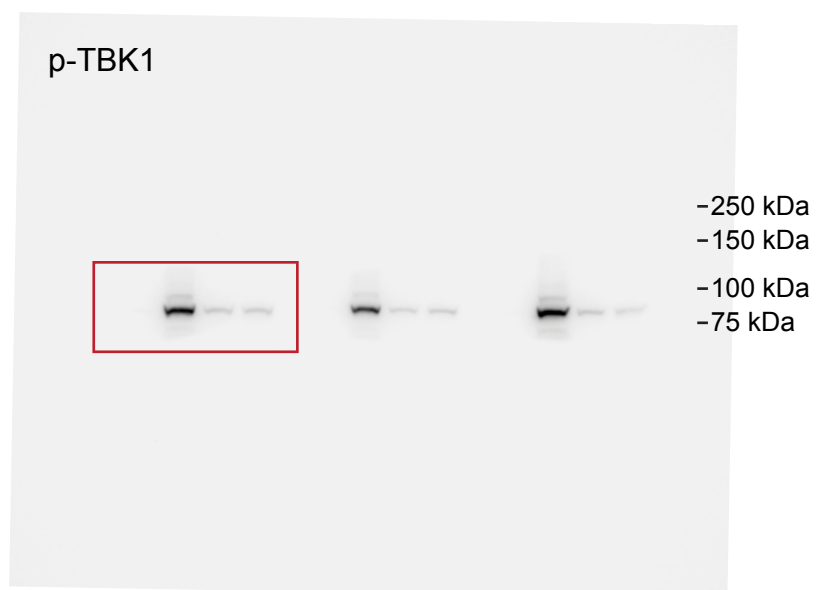

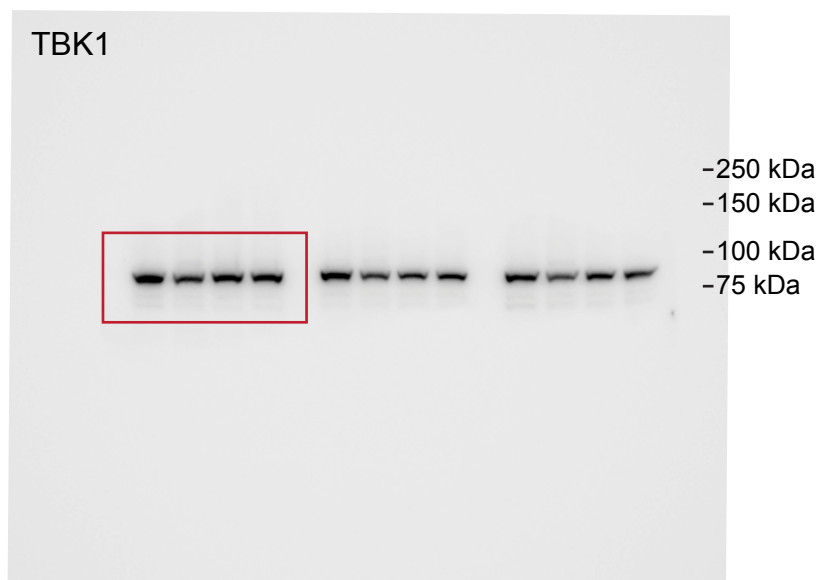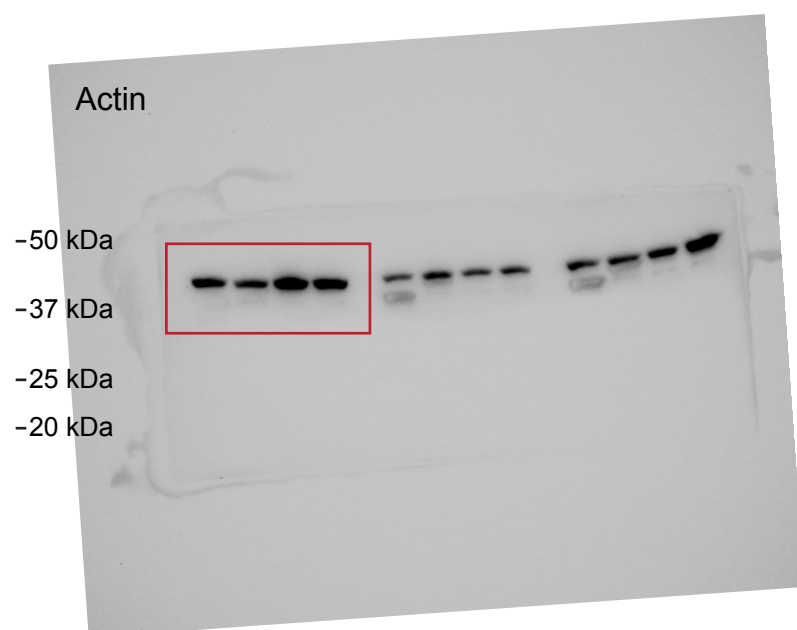

Supplement: Supplementary file 13 — Source Data for Figure 3 [file EMBJ-42-e112712-s005.zip › Figure 3/Figure 3A.pdf]

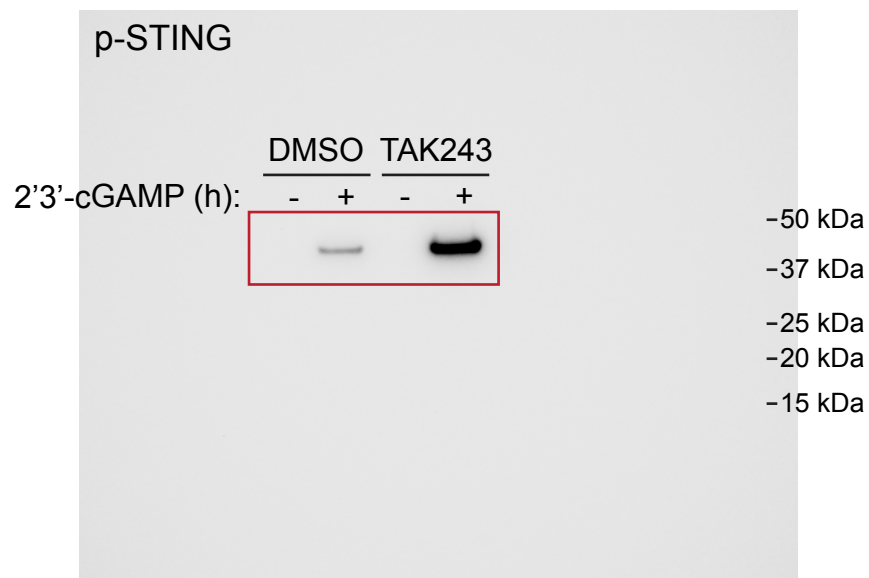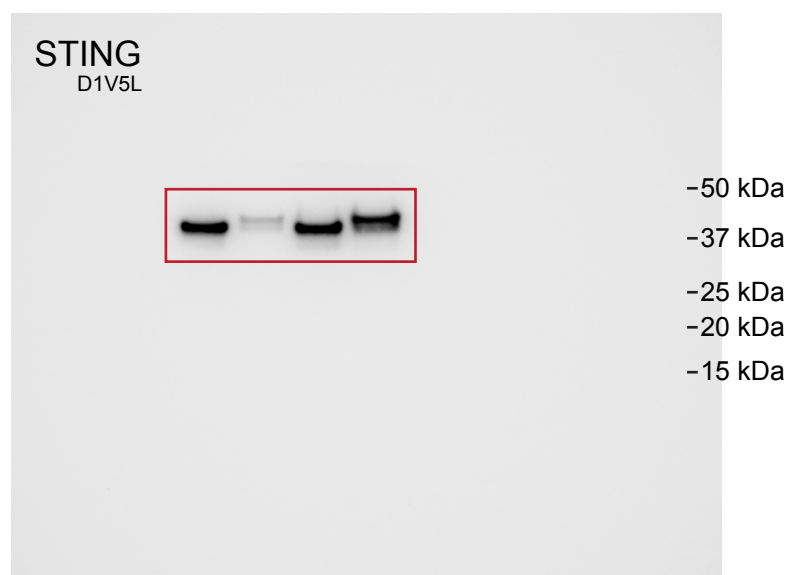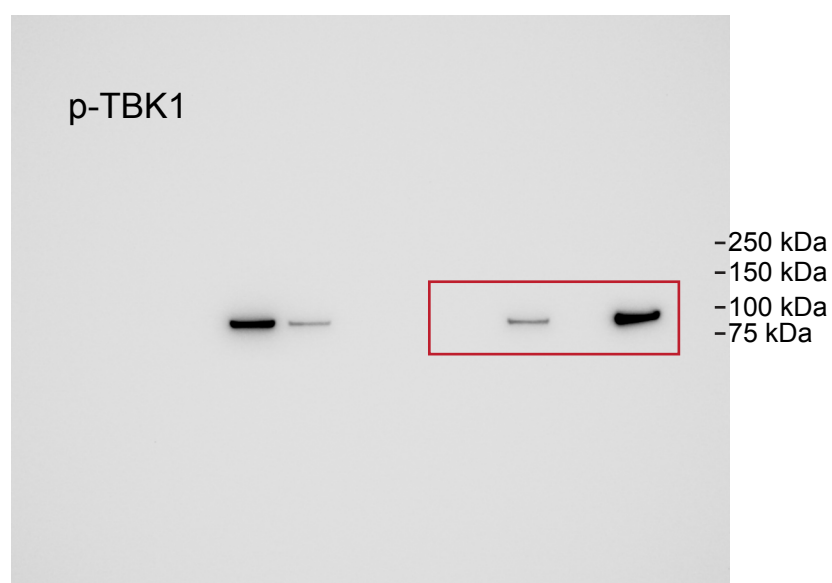

TBK1

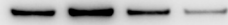

-250 kDa  
-150 kDa  
-100 kDa  
-75 kDa

p-IKK $\epsilon$

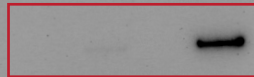

-250 kDa  
-150 kDa  
-100 kDa  
-75 kDa

IKK $\epsilon$

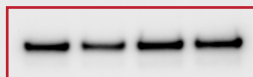

-250 kDa  
-150 kDa  
-100 kDa  
-75 kDa

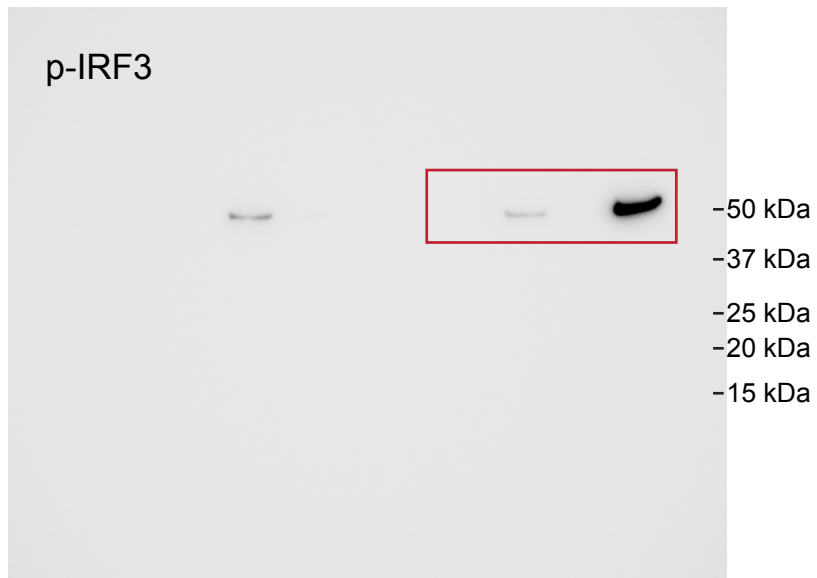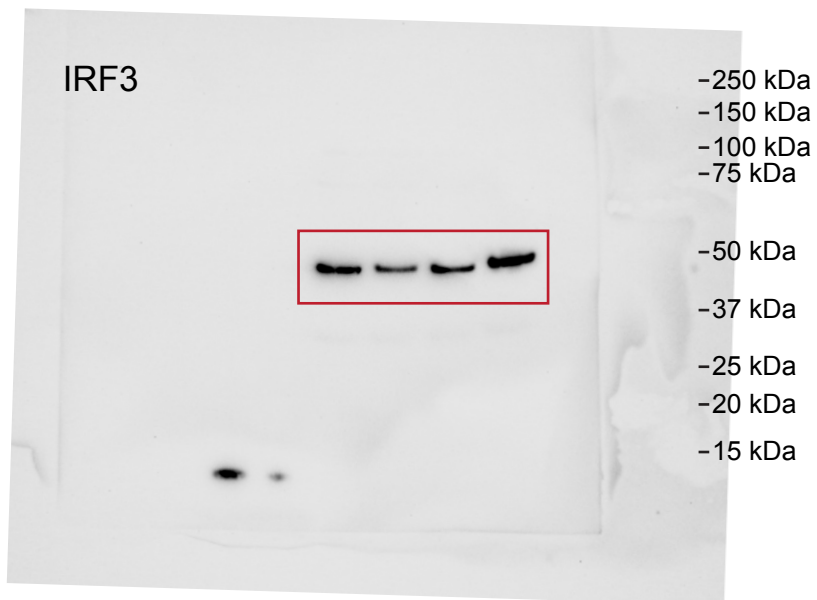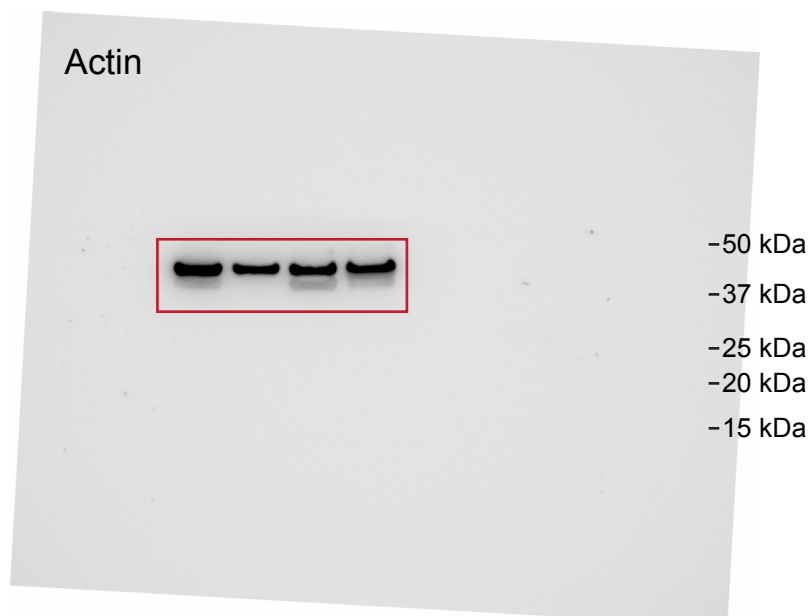

Supplement: Supplementary file 14 — Source Data for Figure 4 [file EMBJ-42-e112712-s006.zip › Figure 4/Figure 4C.pdf]

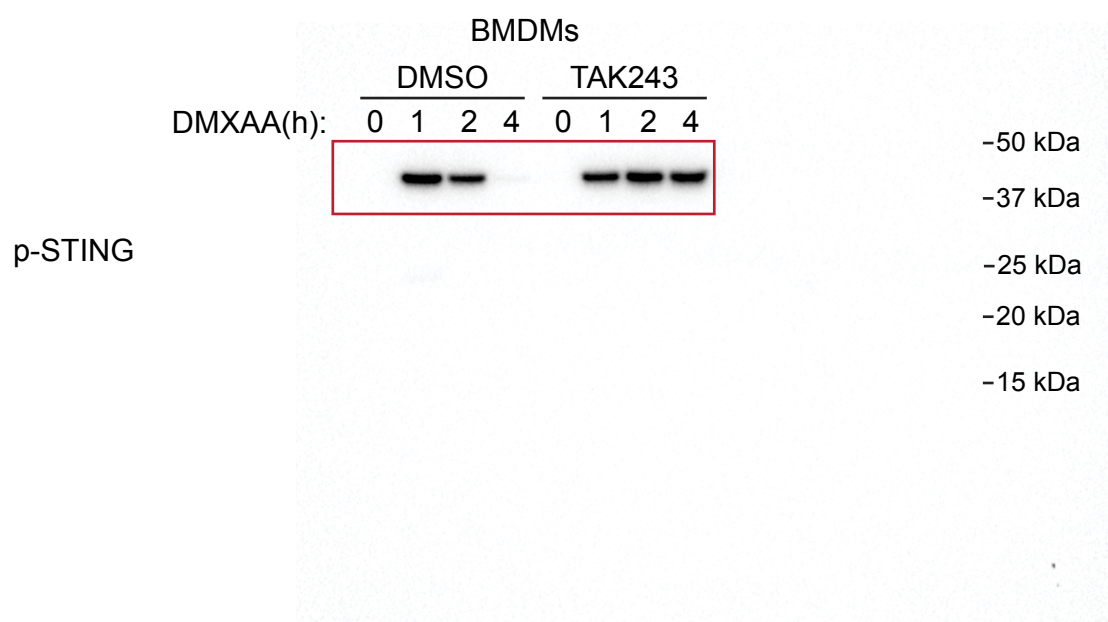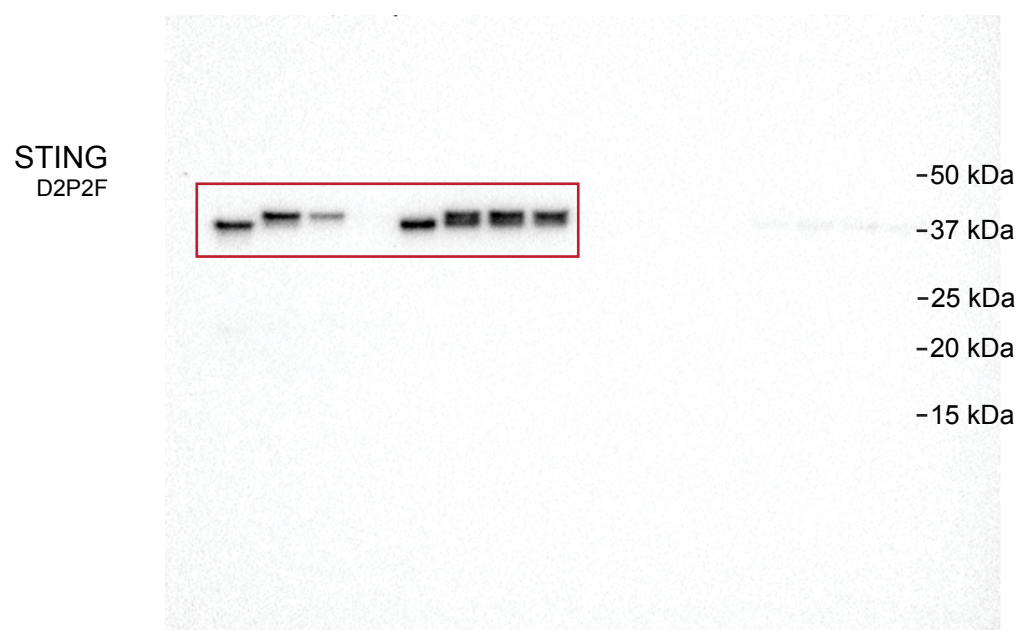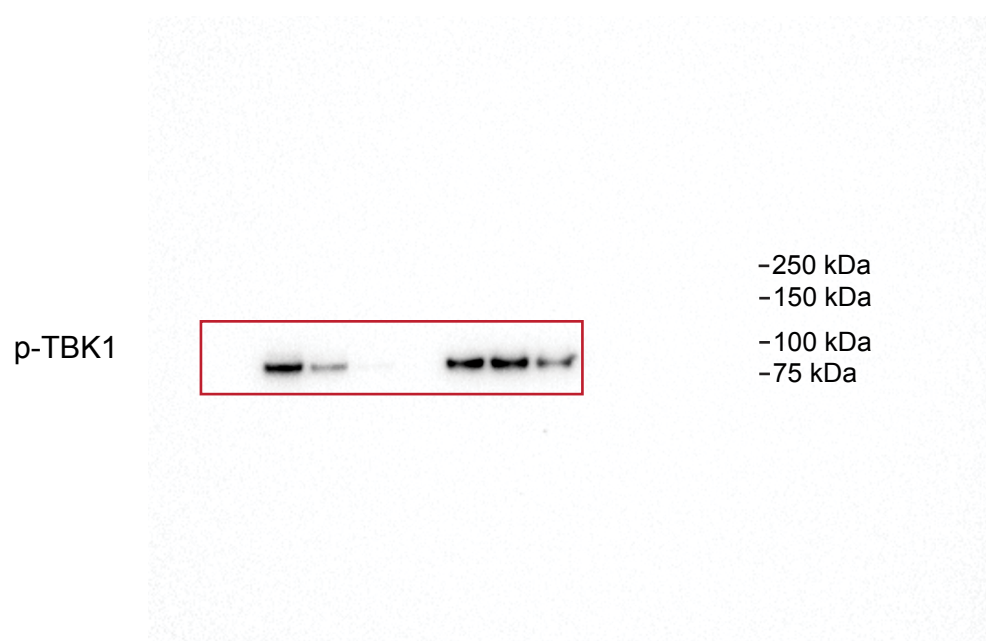

TBK1

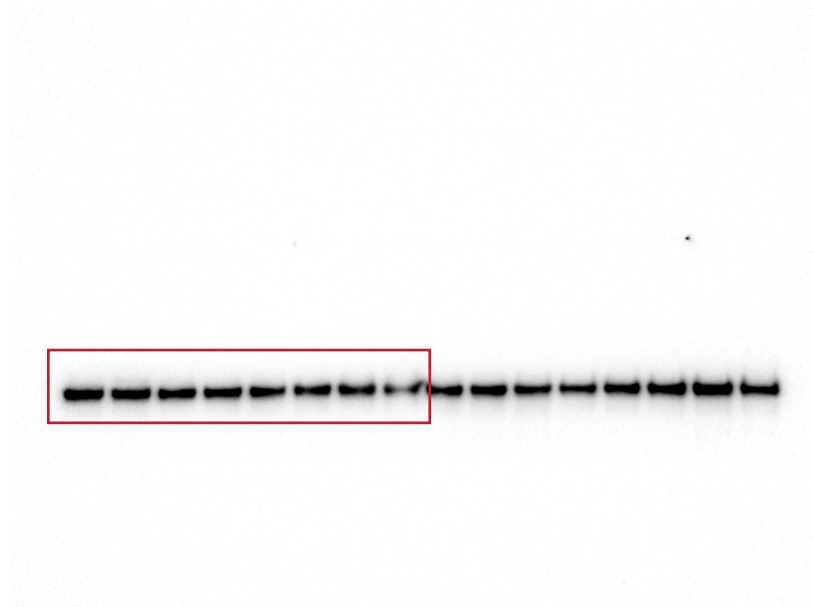

-250 kDa  
-150 kDa  
-100 kDa  
-75 kDa

p-IKK $\epsilon$

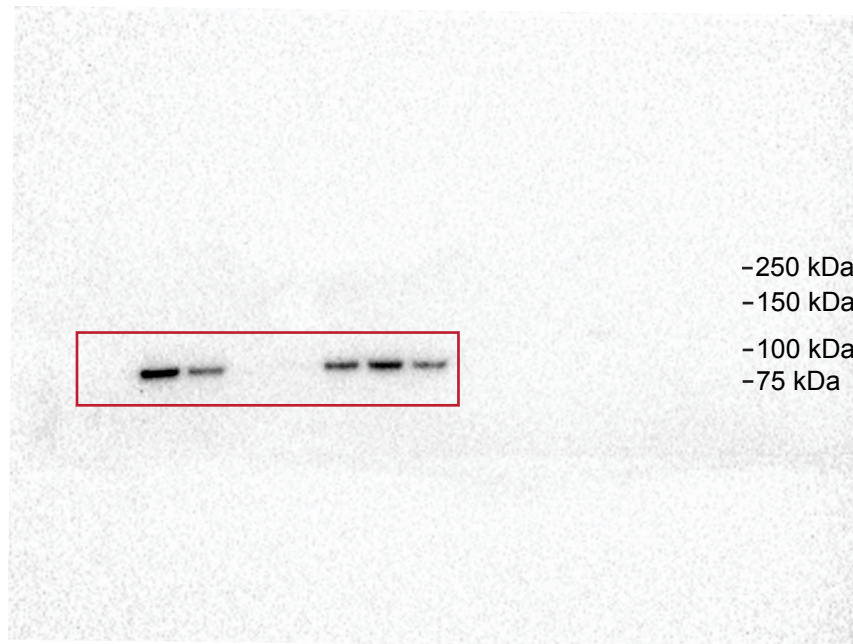

-250 kDa  
-150 kDa  
-100 kDa  
-75 kDa

IKK $\epsilon$

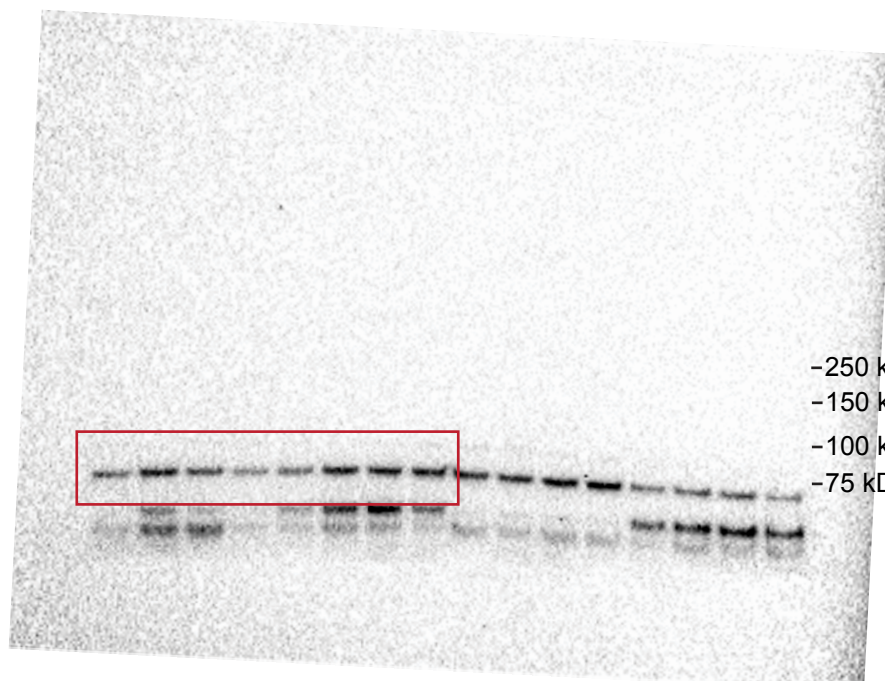

-250 kDa  
-150 kDa  
-100 kDa  
-75 kDa

p-IRF3

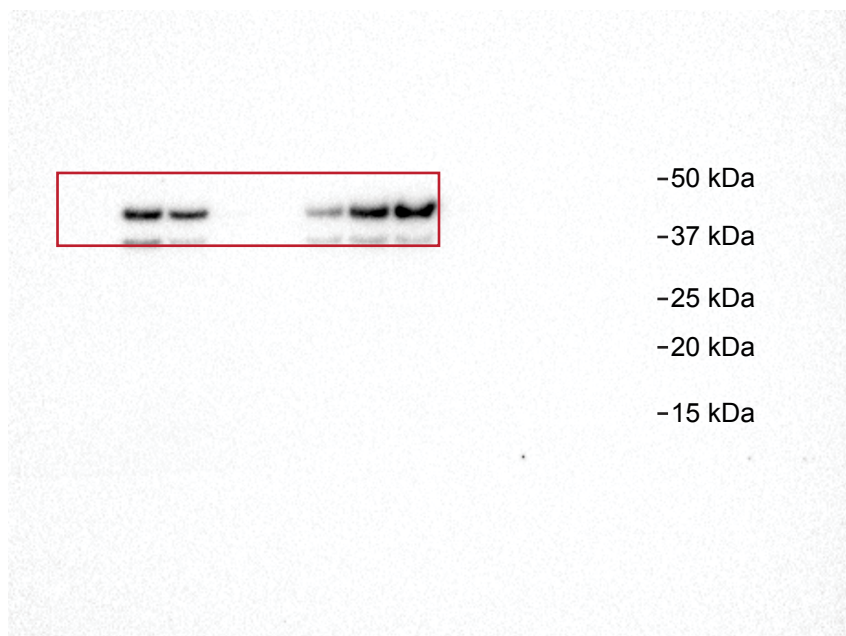

IRF3

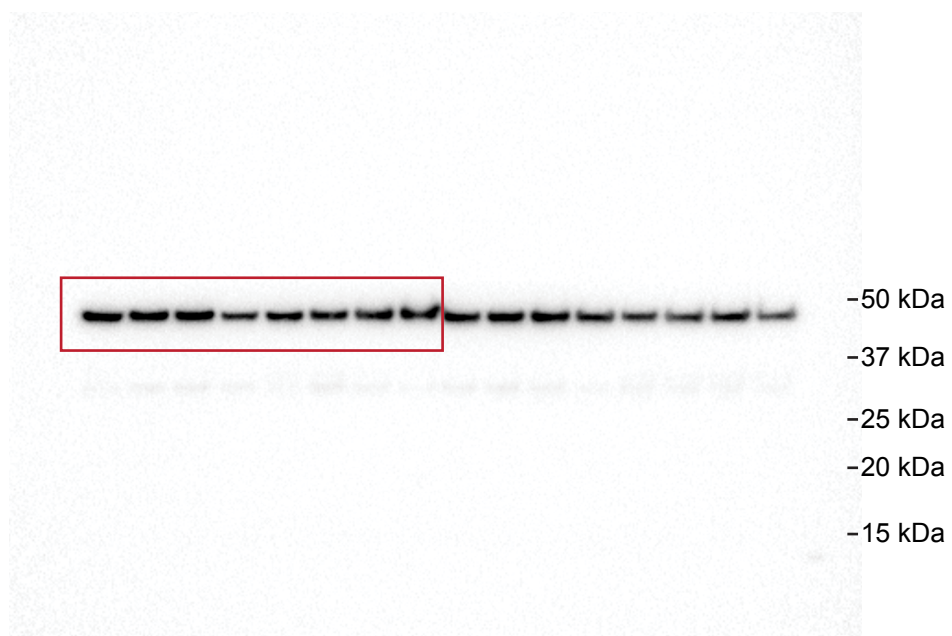

Actin

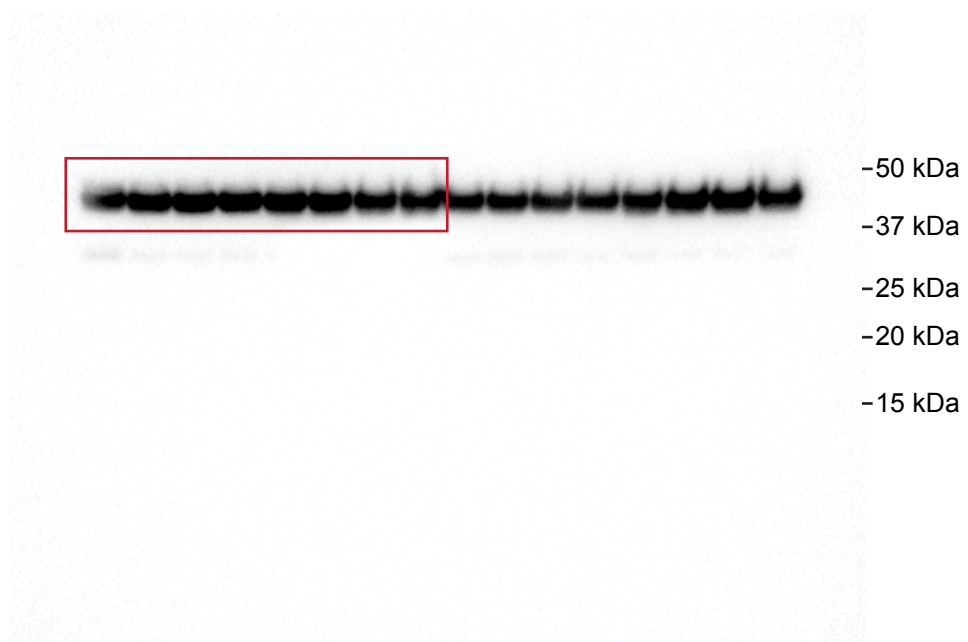

Supplement: Supplementary file 14 — Source Data for Figure 4 [file EMBJ-42-e112712-s006.zip › Figure 4/Figure 4B.pdf]

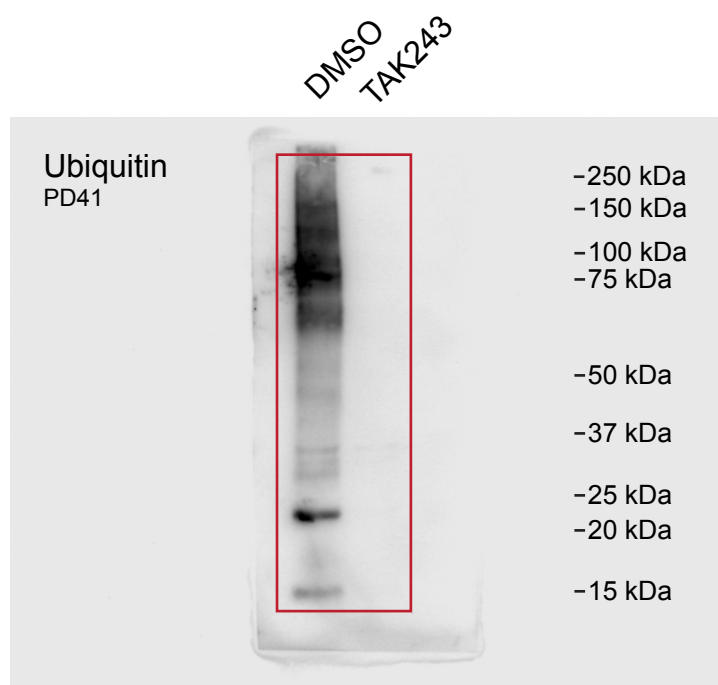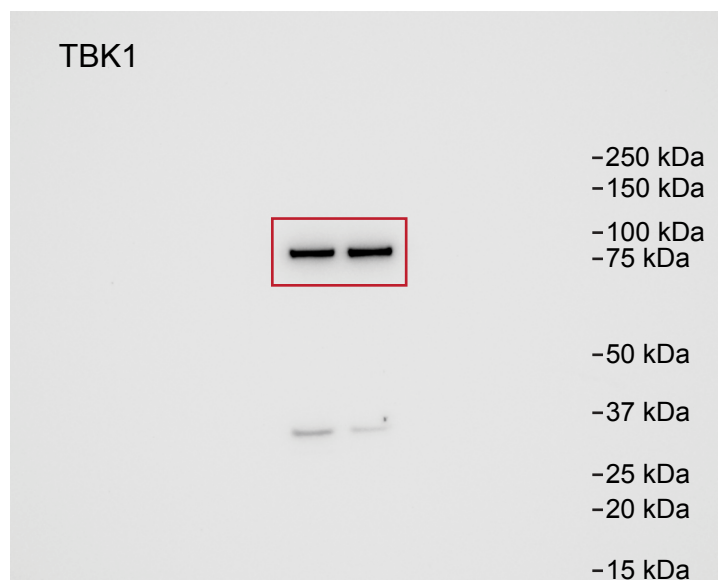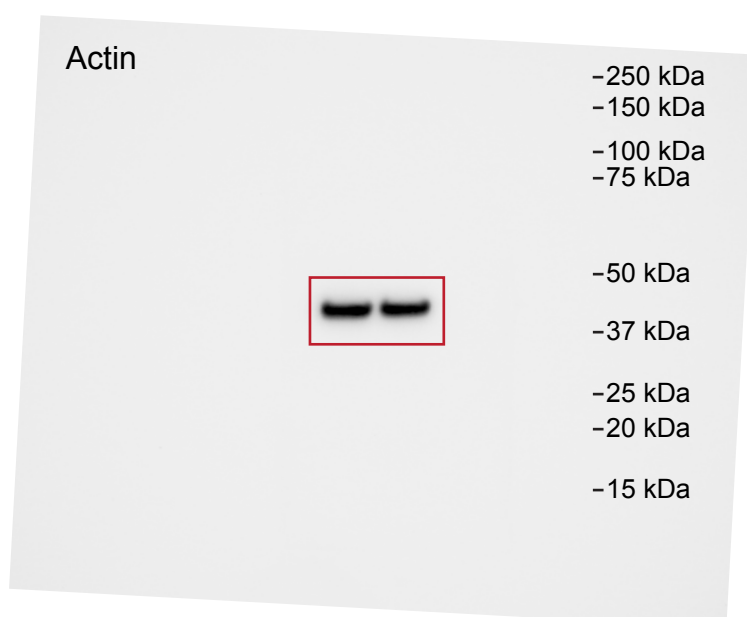

Supplement: Supplementary file 14 — Source Data for Figure 4 [file EMBJ-42-e112712-s006.zip › Figure 4/Figure 4A.pdf]

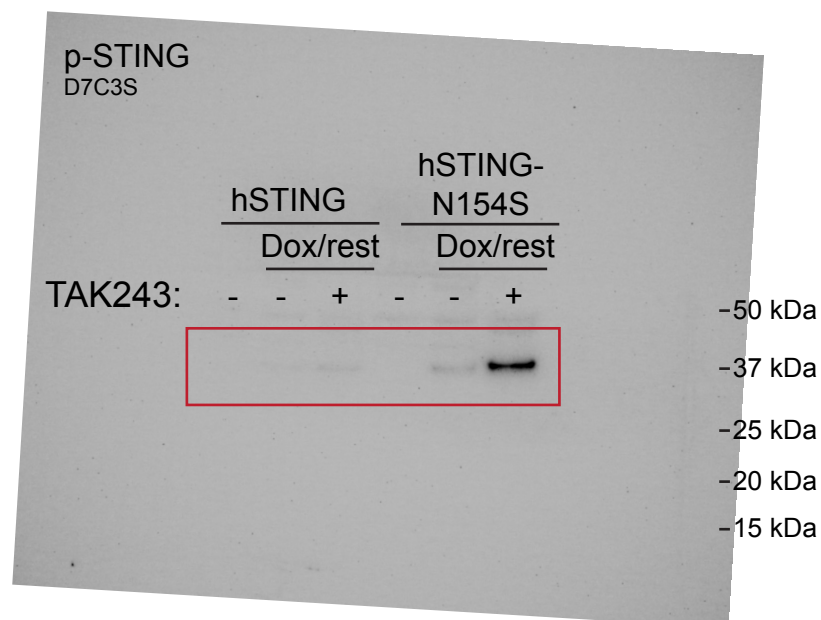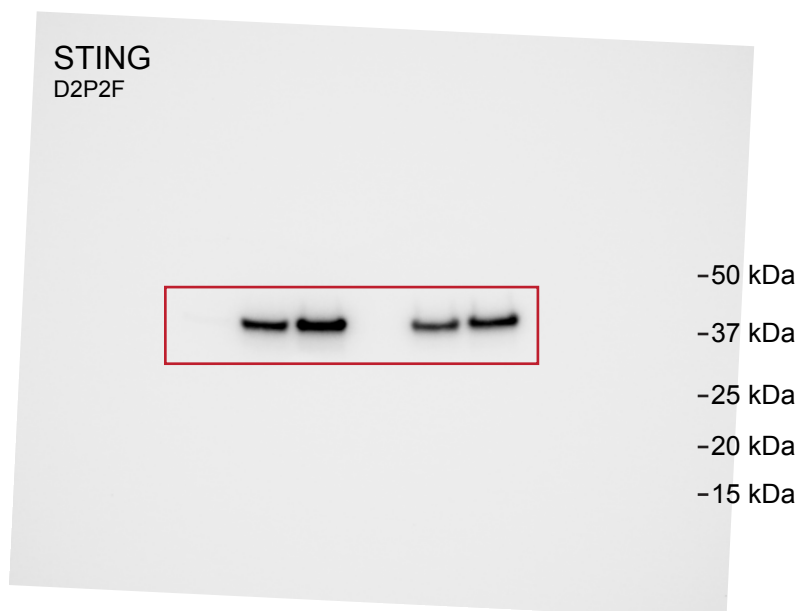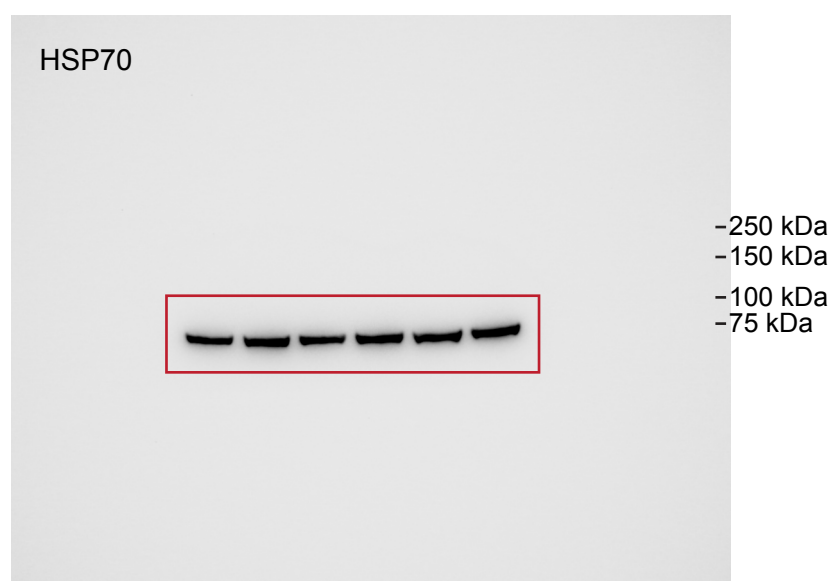

Supplement: Supplementary file 14 — Source Data for Figure 4 [file EMBJ-42-e112712-s006.zip › Figure 4/Figure 4J.pdf]

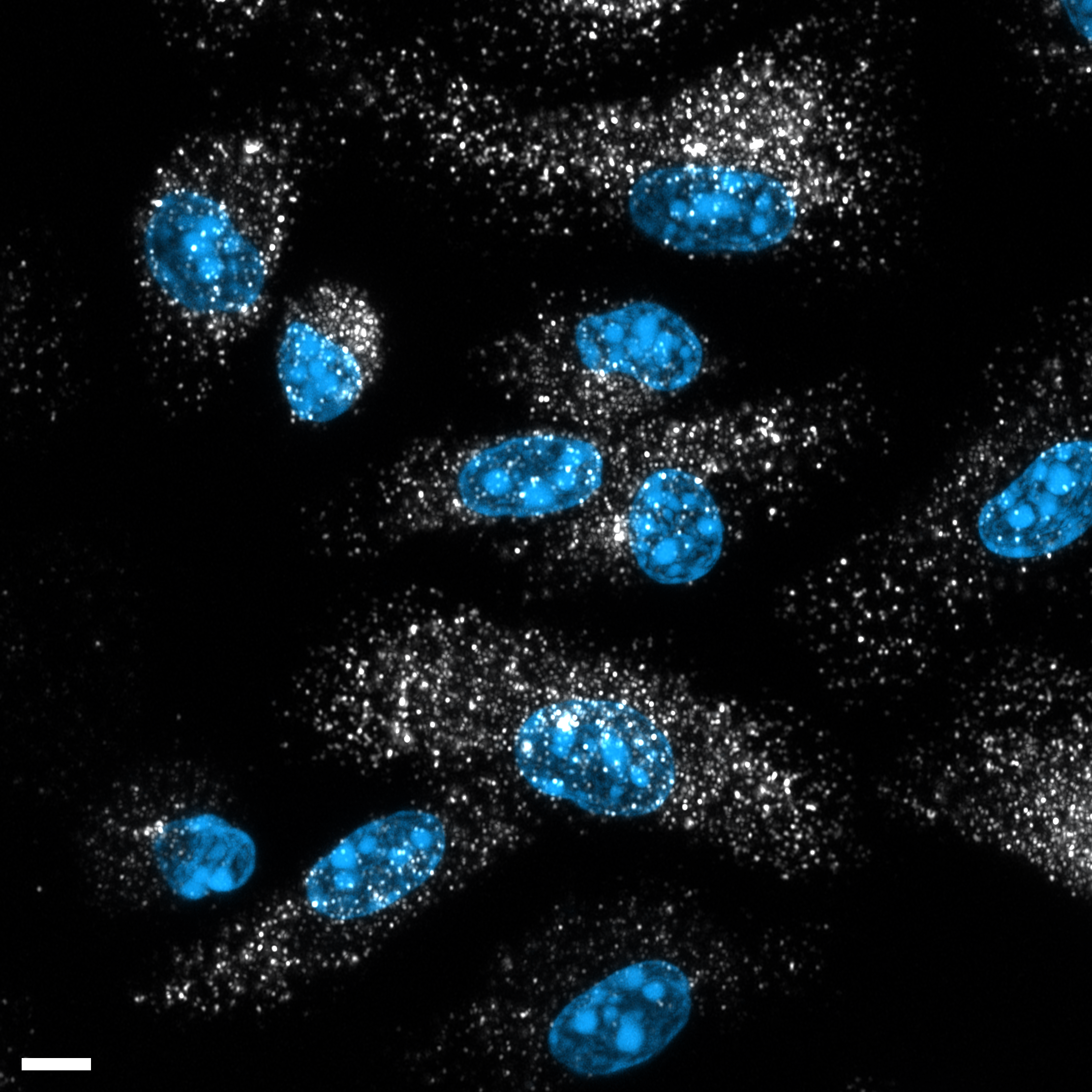

Supplement: Supplementary file 14 — Source Data for Figure 4 [file EMBJ-42-e112712-s006.zip › Figure 4/Figure 4D/TAK243-DMXAA2h.tif]

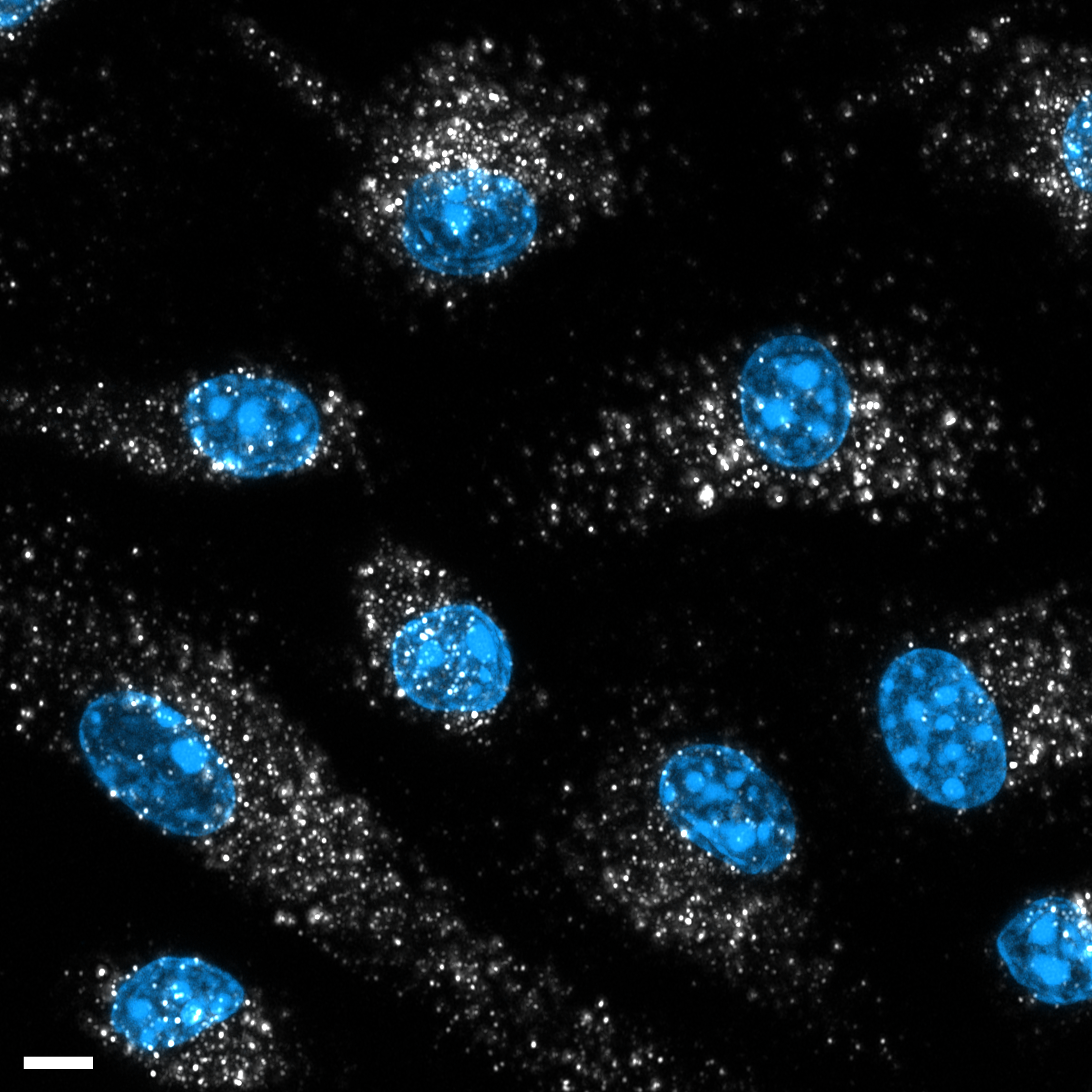

Supplement: Supplementary file 14 — Source Data for Figure 4 [file EMBJ-42-e112712-s006.zip › Figure 4/Figure 4D/UT-DMXAA1h.tif]

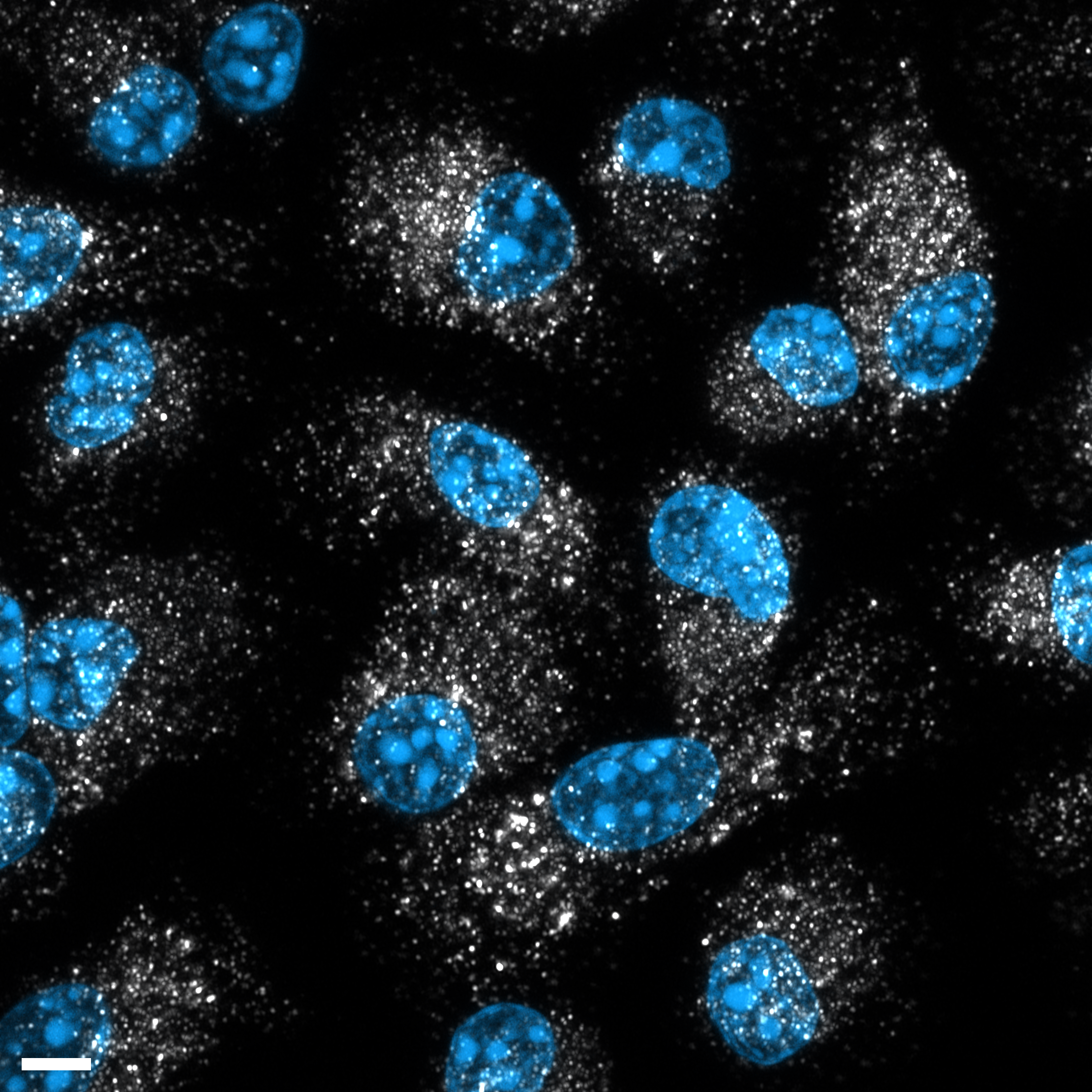

Supplement: Supplementary file 14 — Source Data for Figure 4 [file EMBJ-42-e112712-s006.zip › Figure 4/Figure 4D/TAK243-DMXAA4h.tif]

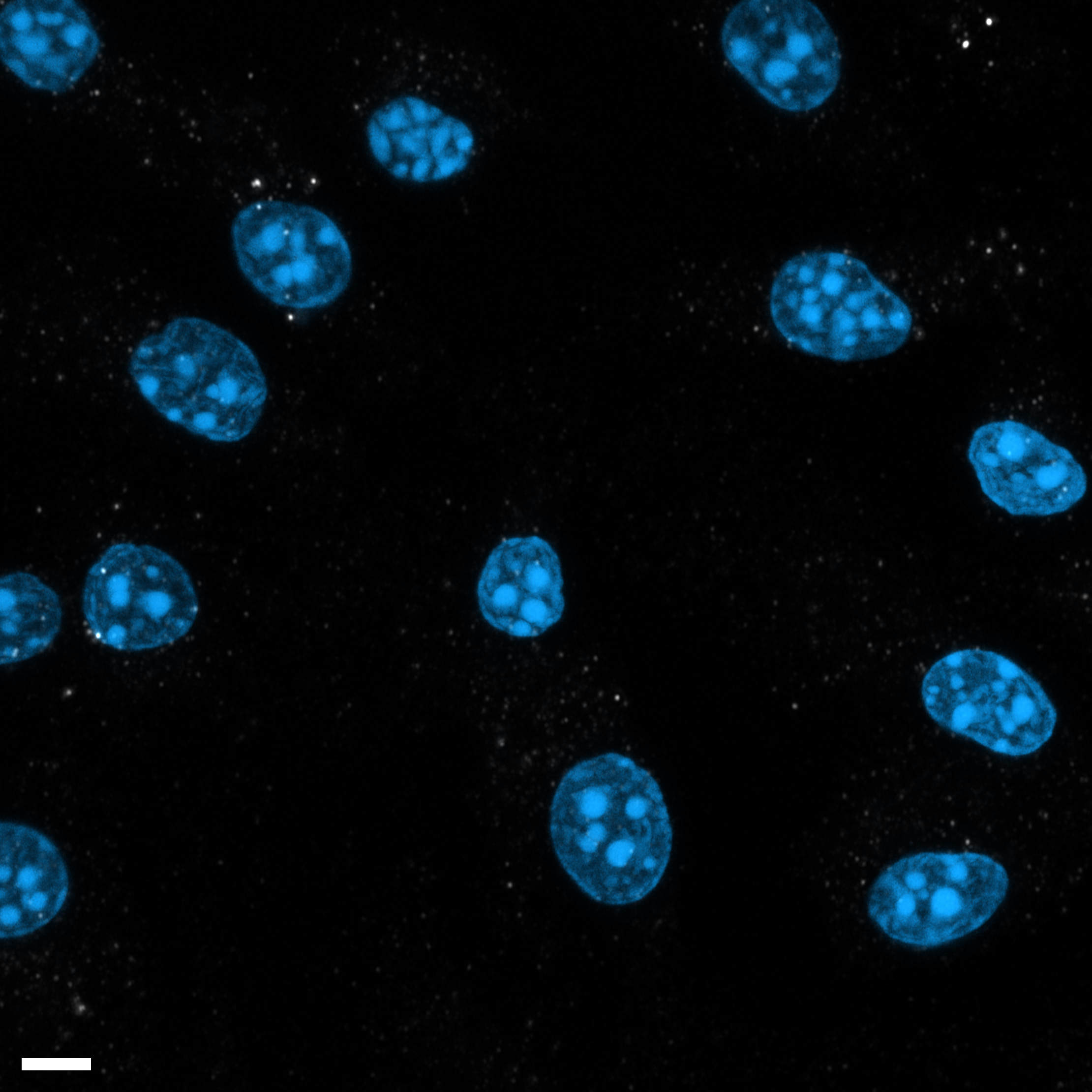

Supplement: Supplementary file 14 — Source Data for Figure 4 [file EMBJ-42-e112712-s006.zip › Figure 4/Figure 4D/UT-DMXAA4h.tif]

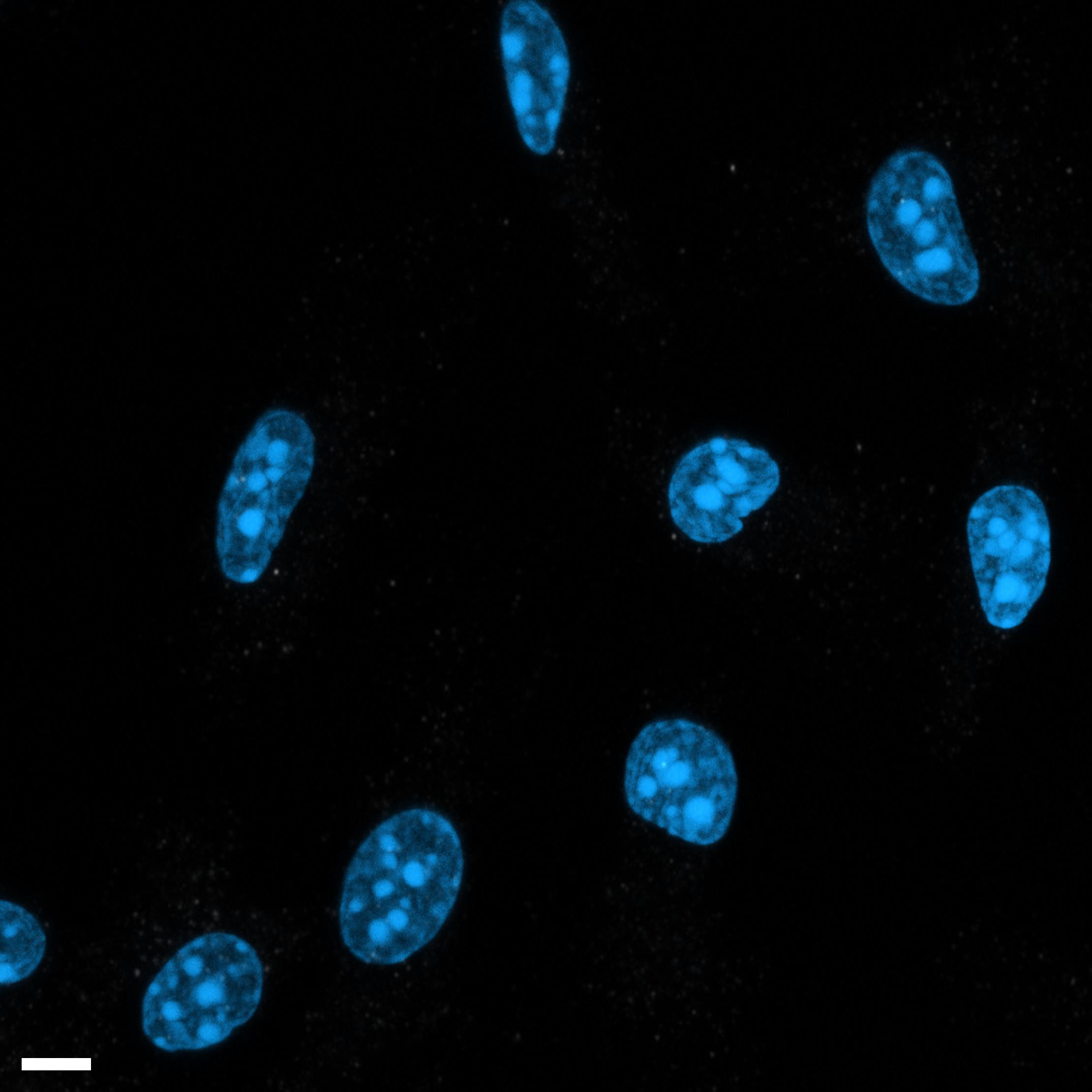

Supplement: Supplementary file 14 — Source Data for Figure 4 [file EMBJ-42-e112712-s006.zip › Figure 4/Figure 4D/UT-UT.tif]

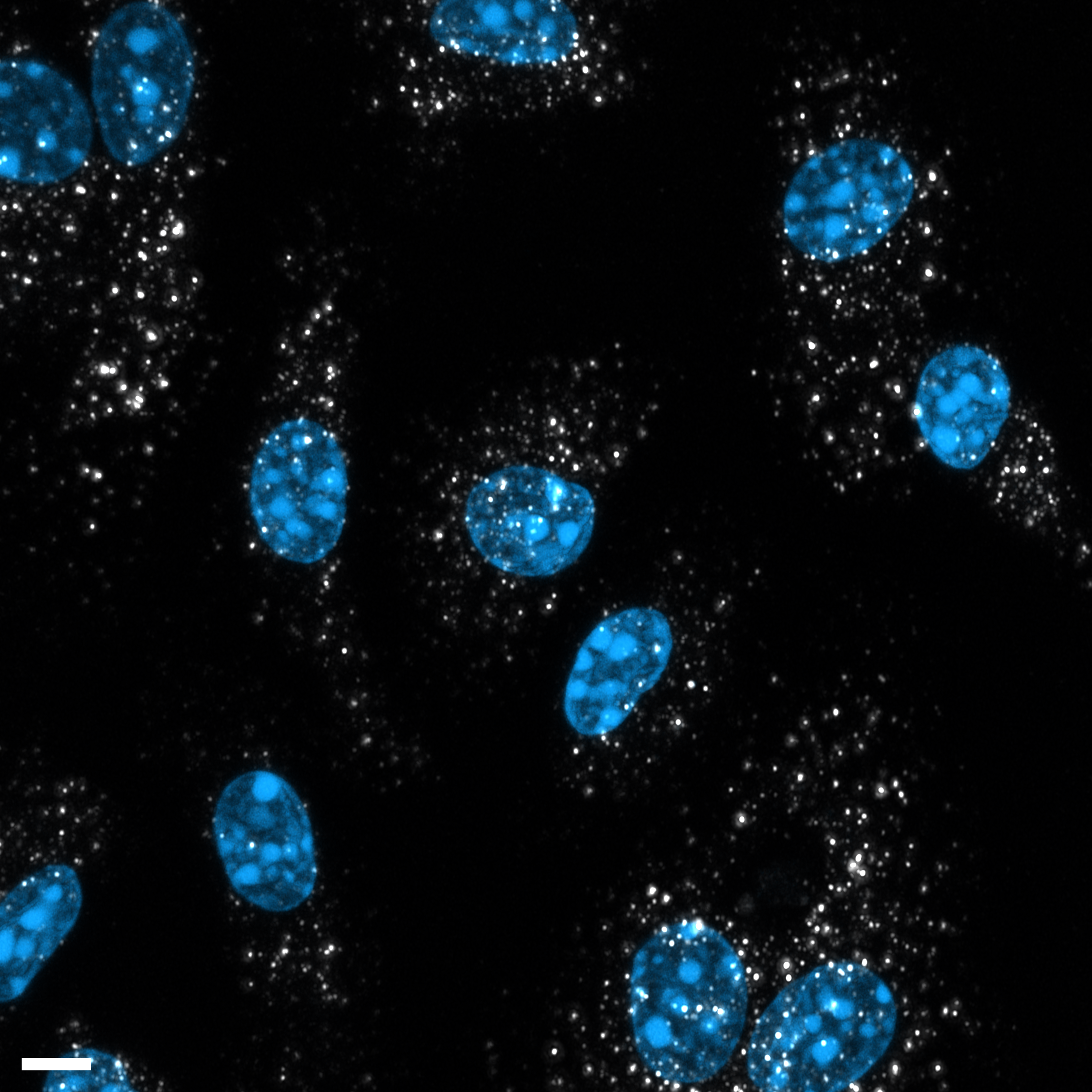

Supplement: Supplementary file 14 — Source Data for Figure 4 [file EMBJ-42-e112712-s006.zip › Figure 4/Figure 4D/UT-DMXAA2h.tif]

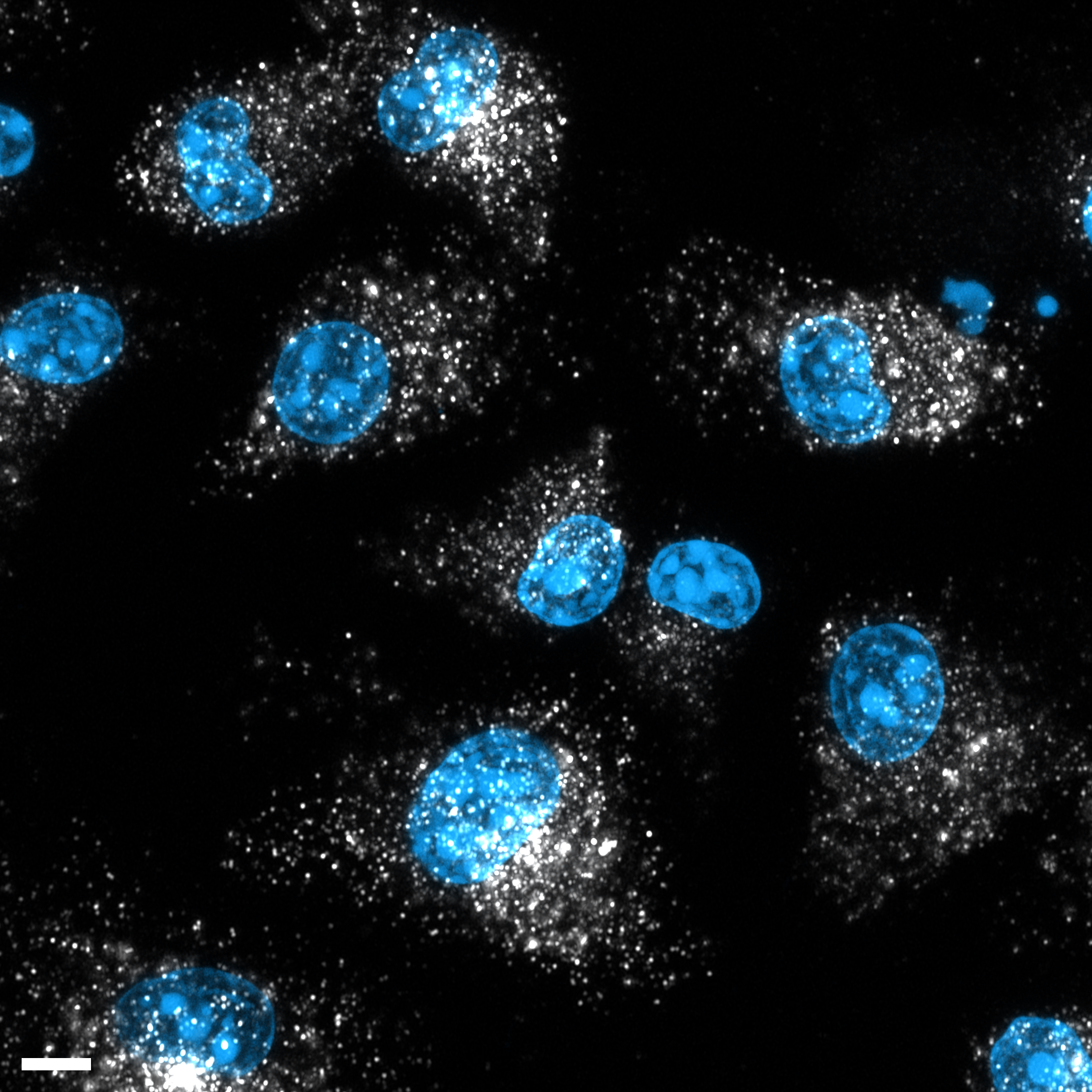

Supplement: Supplementary file 14 — Source Data for Figure 4 [file EMBJ-42-e112712-s006.zip › Figure 4/Figure 4D/TAK243-DMXAA1h.tif]

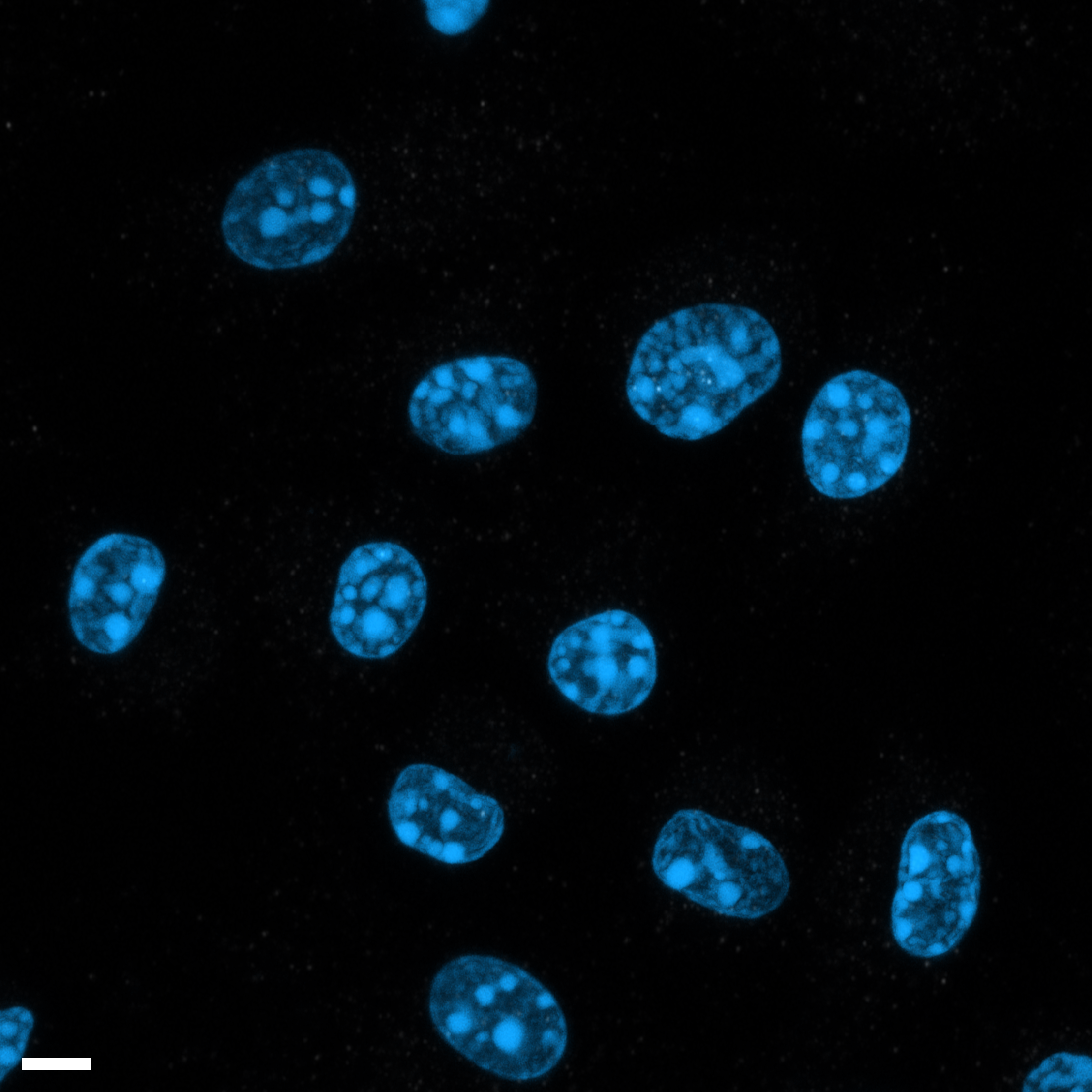

Supplement: Supplementary file 14 — Source Data for Figure 4 [file EMBJ-42-e112712-s006.zip › Figure 4/Figure 4D/TAK243-UT.tif]

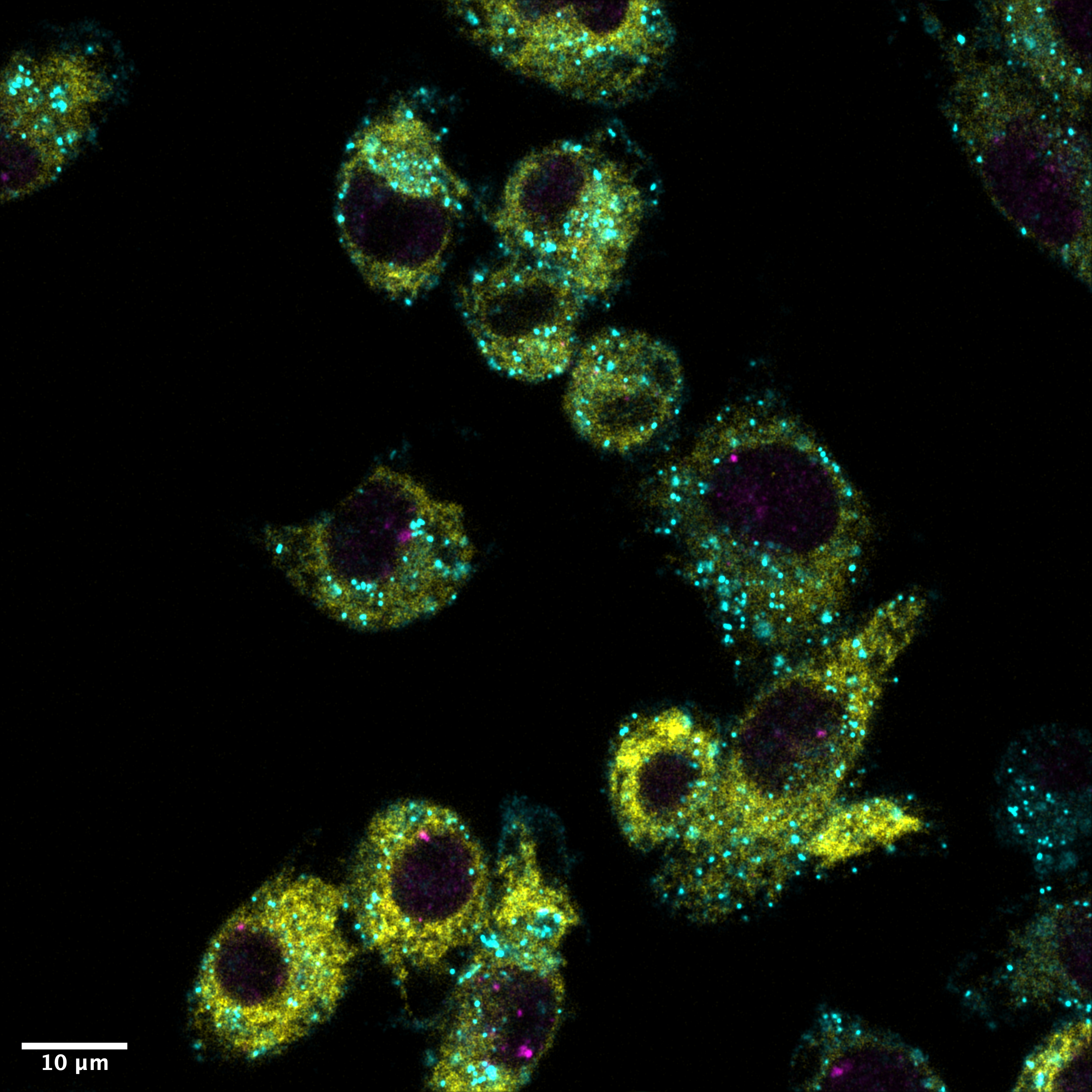

Supplement: Supplementary file 15 — Source Data for Figure 5 [file EMBJ-42-e112712-s014.zip › Figure 5/Figure 5C/UT_composite_RGB.tif]

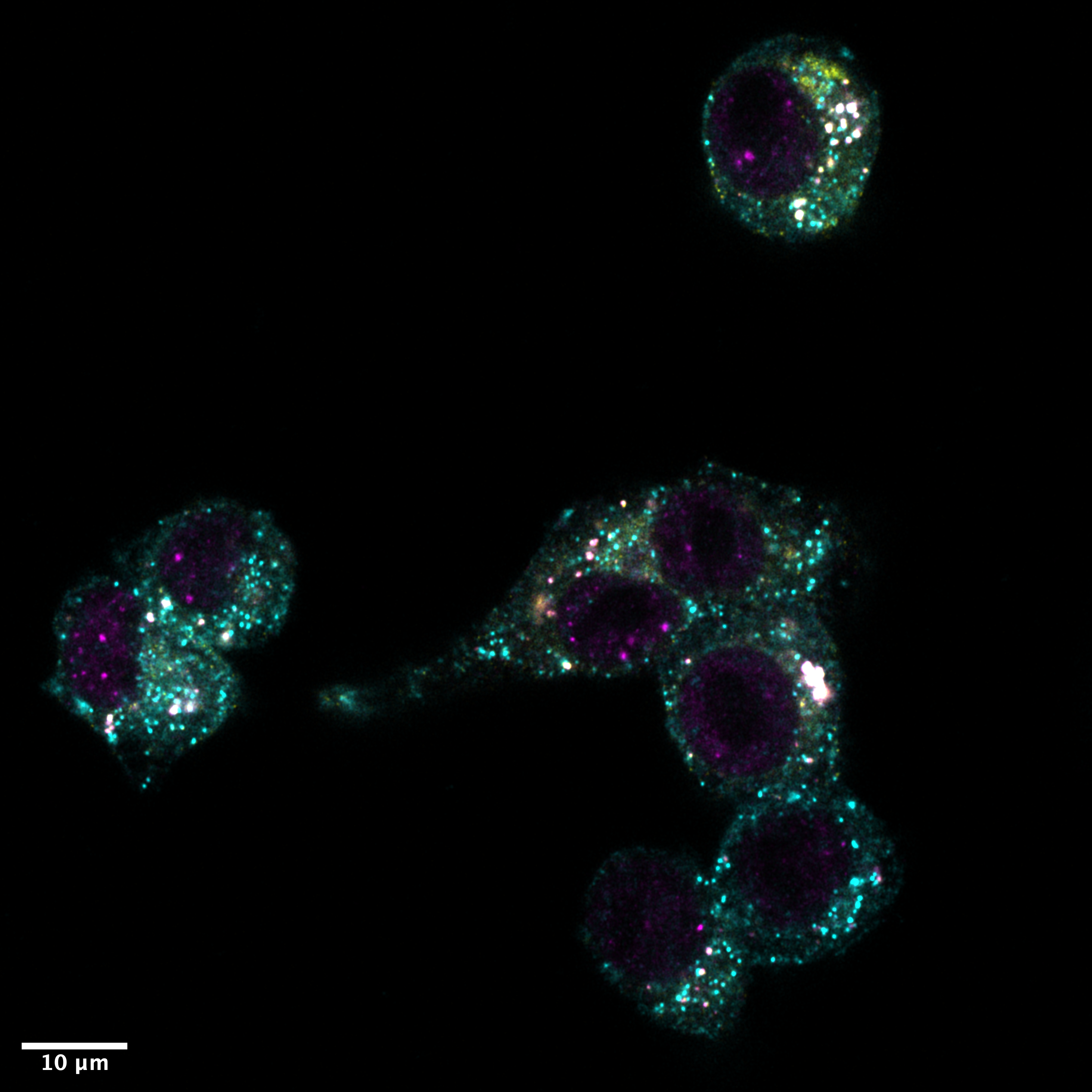

Supplement: Supplementary file 15 — Source Data for Figure 5 [file EMBJ-42-e112712-s014.zip › Figure 5/Figure 5C/DMXAA3h_composite-RGB.tif]

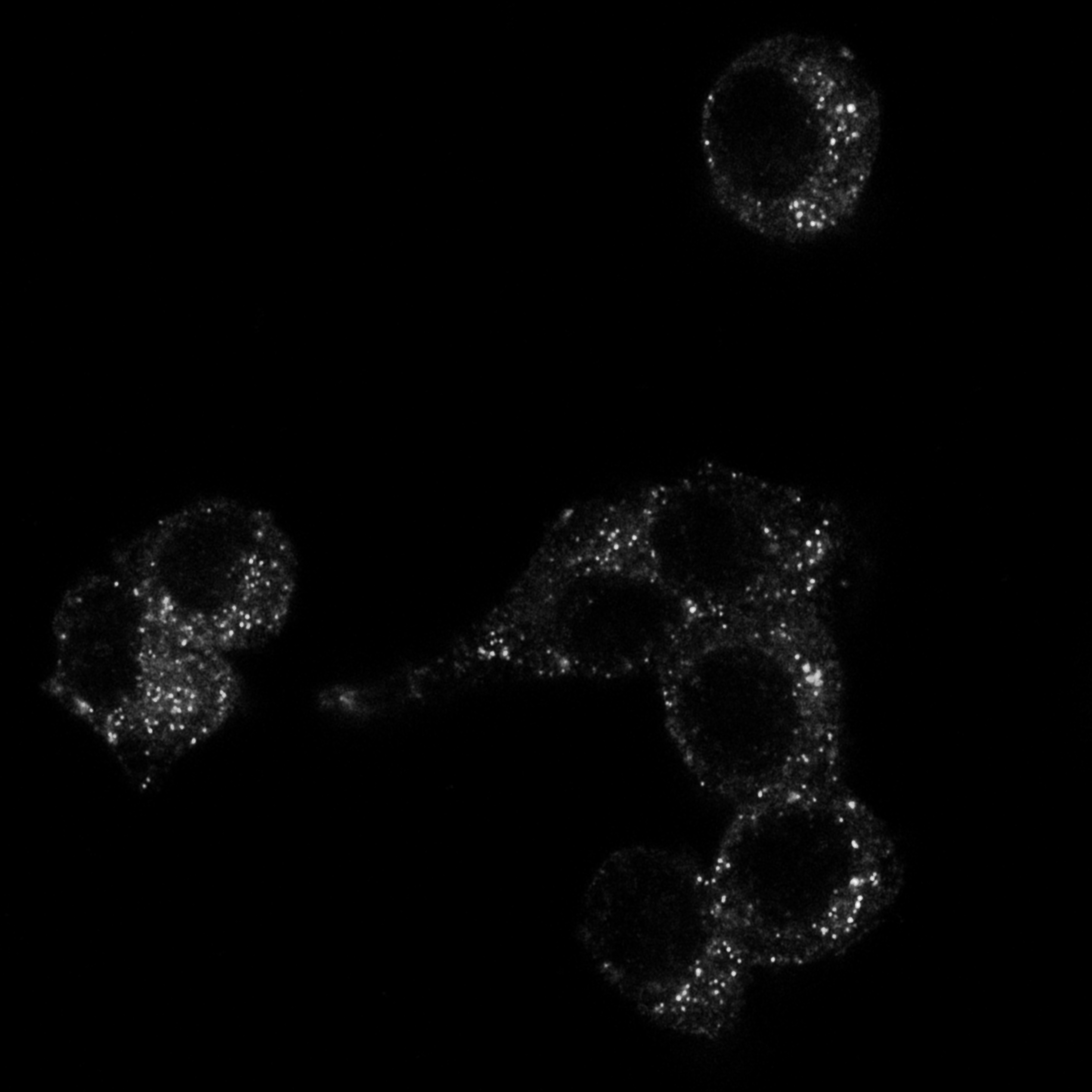

Supplement: Supplementary file 15 — Source Data for Figure 5 [file EMBJ-42-e112712-s014.zip › Figure 5/Figure 5C/DMXAA3h_composite.tif]

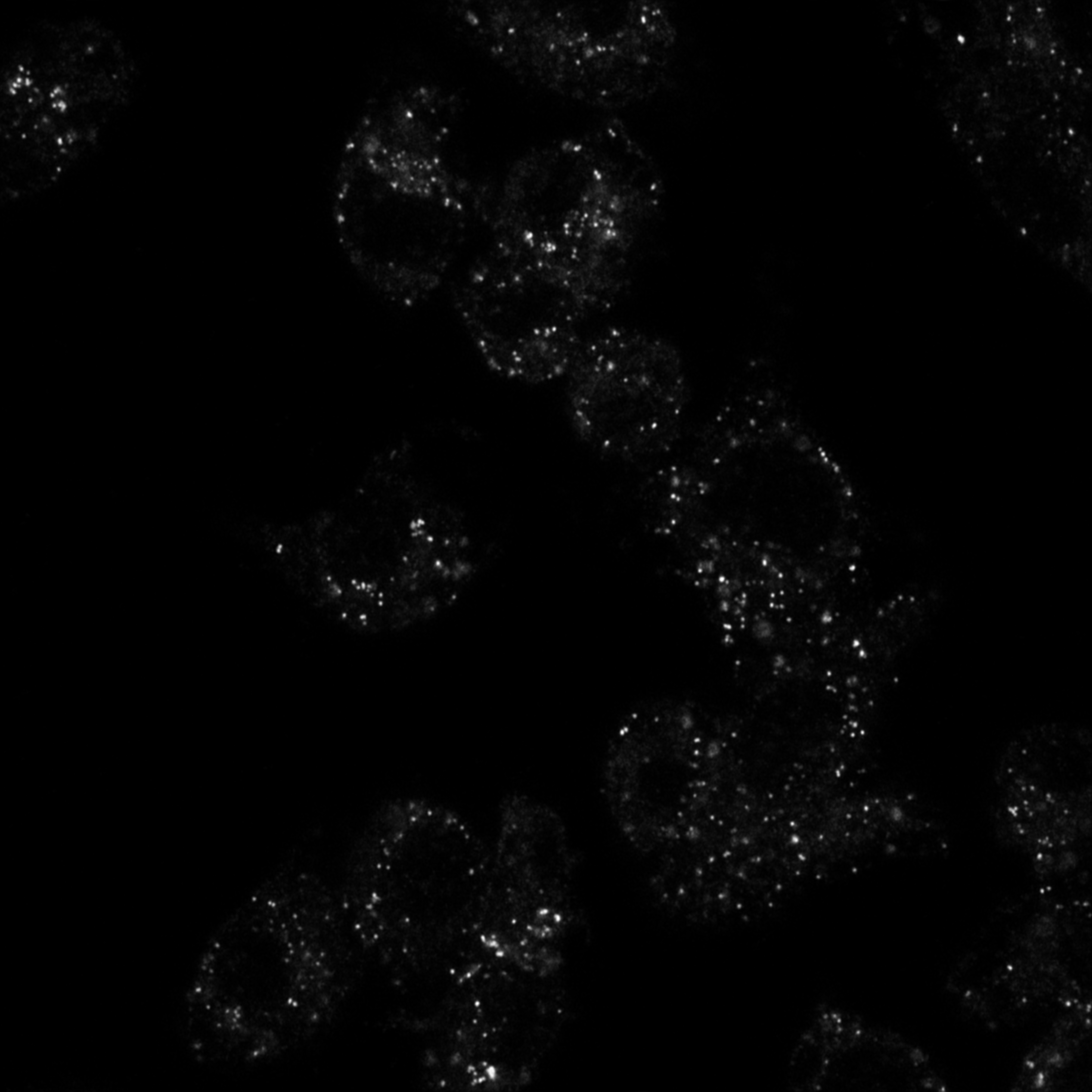

Supplement: Supplementary file 15 — Source Data for Figure 5 [file EMBJ-42-e112712-s014.zip › Figure 5/Figure 5C/UT_composite.tif]

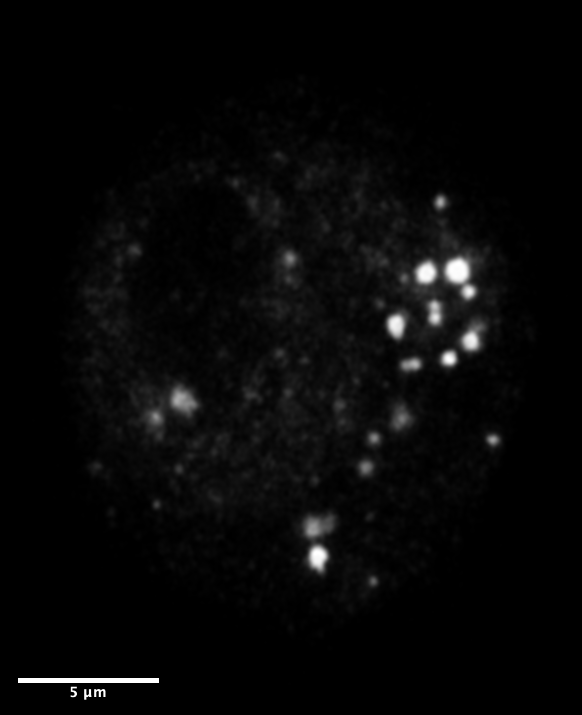

Supplement: Supplementary file 15 — Source Data for Figure 5 [file EMBJ-42-e112712-s014.zip › Figure 5/Figure 5D/DMXAA3h_Ub.tif]

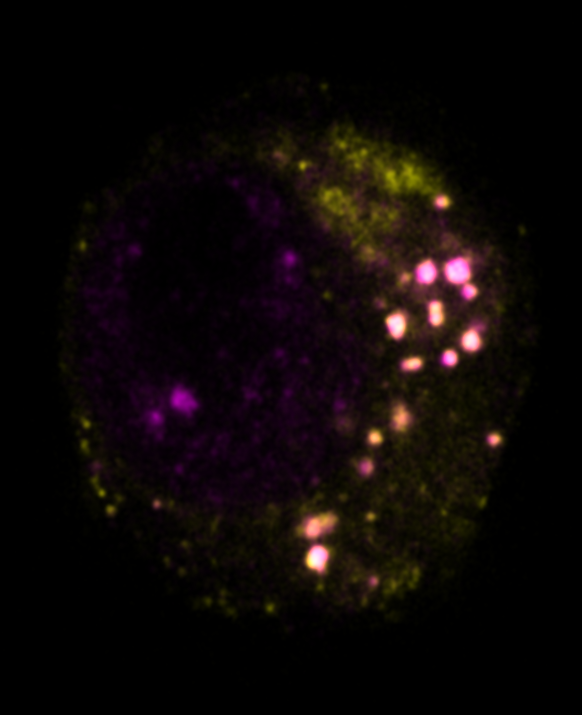

Supplement: Supplementary file 15 — Source Data for Figure 5 [file EMBJ-42-e112712-s014.zip › Figure 5/Figure 5D/DMXAA3h_eGFP-STING:Ub.tif]

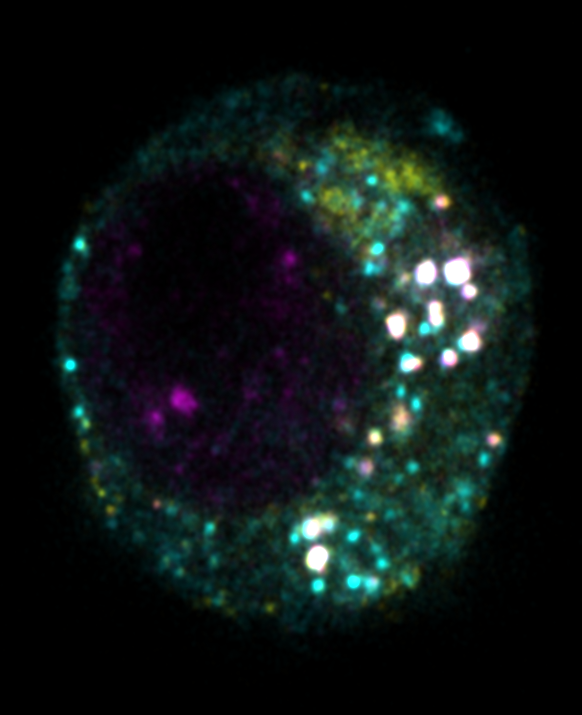

Supplement: Supplementary file 15 — Source Data for Figure 5 [file EMBJ-42-e112712-s014.zip › Figure 5/Figure 5D/DMXAA3h_composite_RGB.tif]

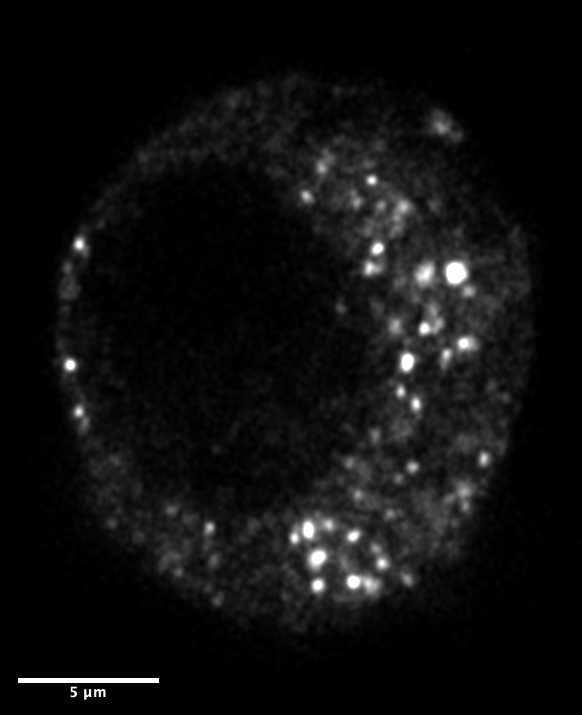

Supplement: Supplementary file 15 — Source Data for Figure 5 [file EMBJ-42-e112712-s014.zip › Figure 5/Figure 5D/DMXAA3h_HRS.tif]

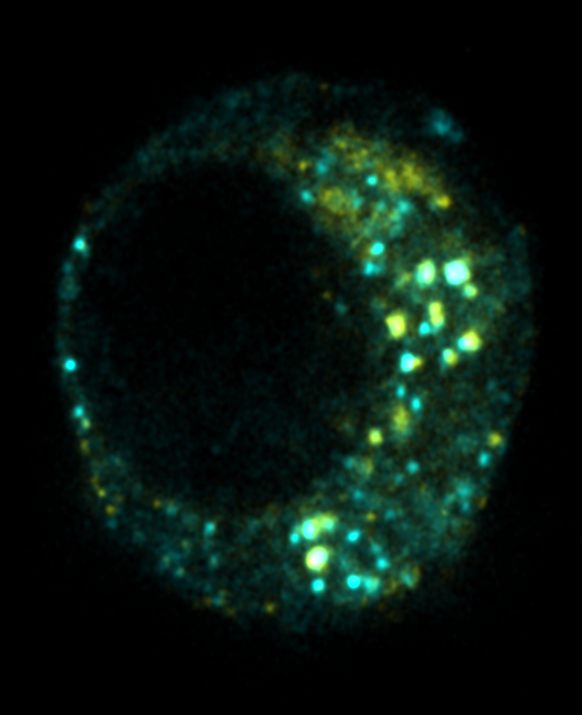

Supplement: Supplementary file 15 — Source Data for Figure 5 [file EMBJ-42-e112712-s014.zip › Figure 5/Figure 5D/DMXAA3h_eGFP-STING:HRS.tif]

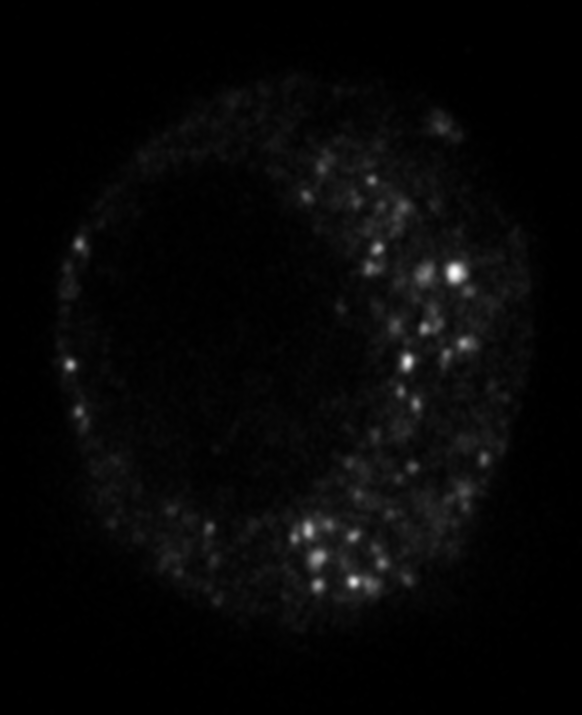

Supplement: Supplementary file 15 — Source Data for Figure 5 [file EMBJ-42-e112712-s014.zip › Figure 5/Figure 5D/DMXAA3h_composite.tif]

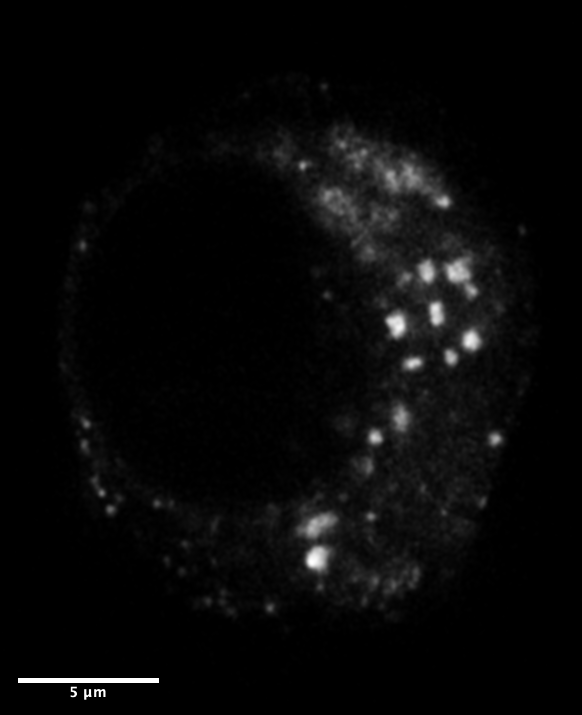

Supplement: Supplementary file 15 — Source Data for Figure 5 [file EMBJ-42-e112712-s014.zip › Figure 5/Figure 5D/DMXAA3h_eGFP-STING.tif]

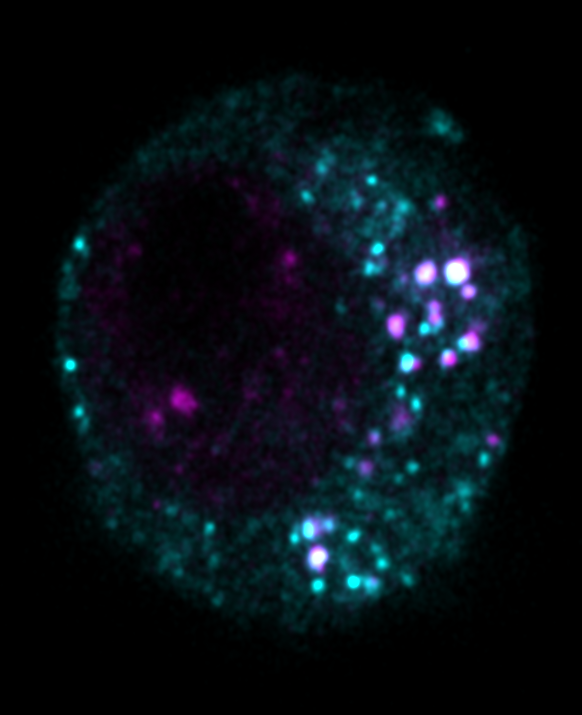

Supplement: Supplementary file 15 — Source Data for Figure 5 [file EMBJ-42-e112712-s014.zip › Figure 5/Figure 5D/DMXAA3h_HRS:Ub.tif]

# p-STING

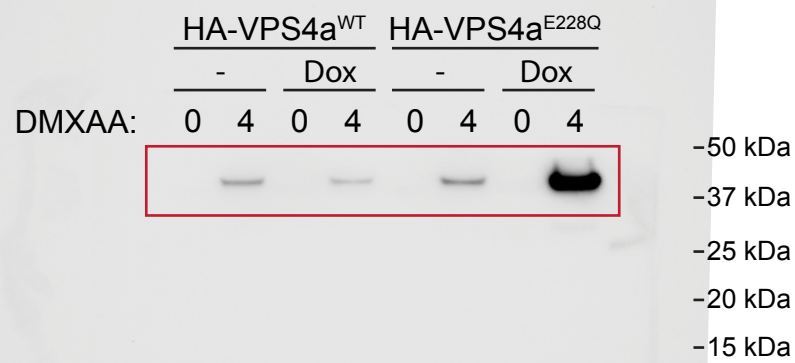

# STING D2P2F

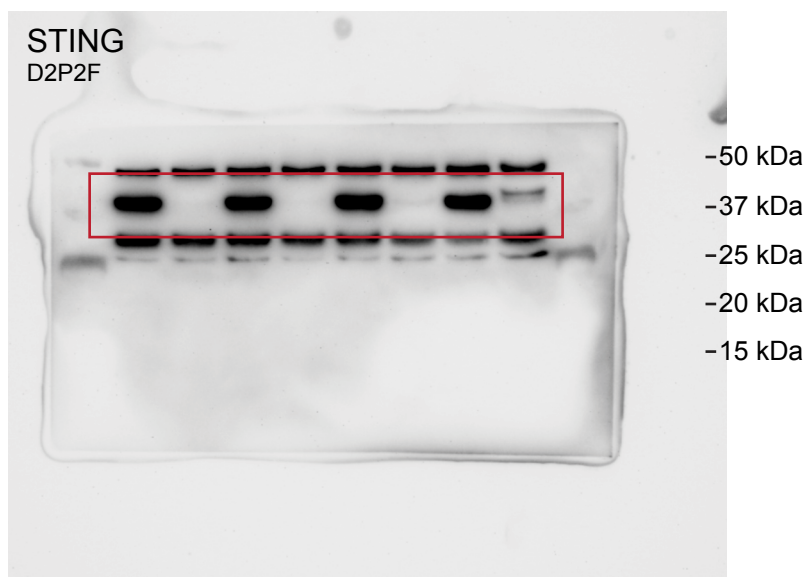

# STING D1V5L

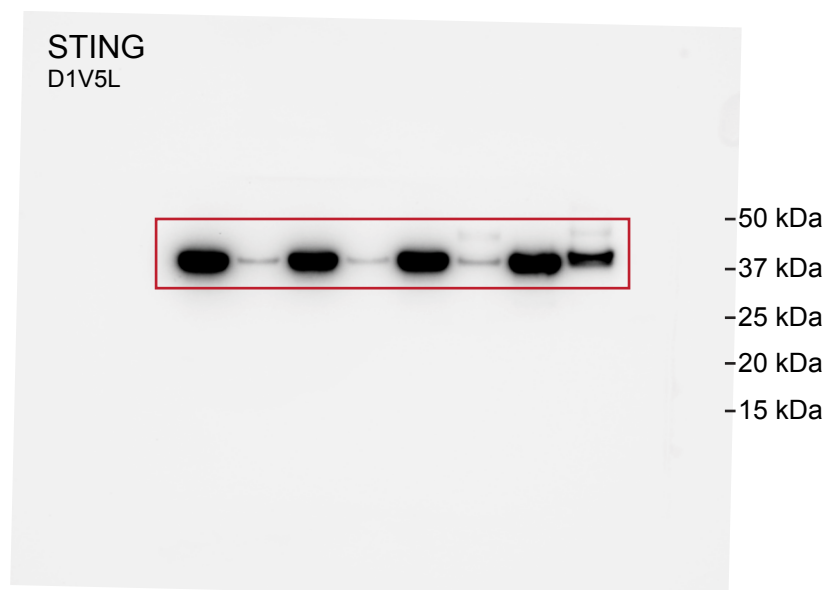

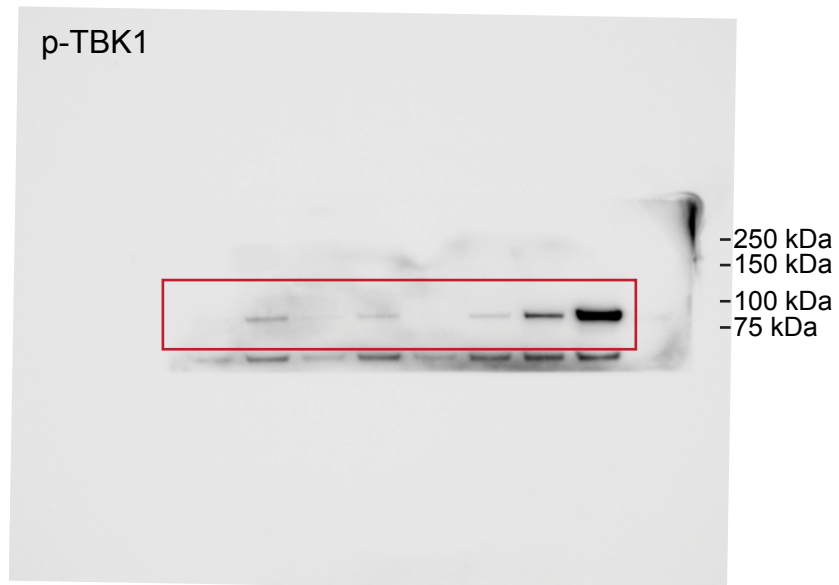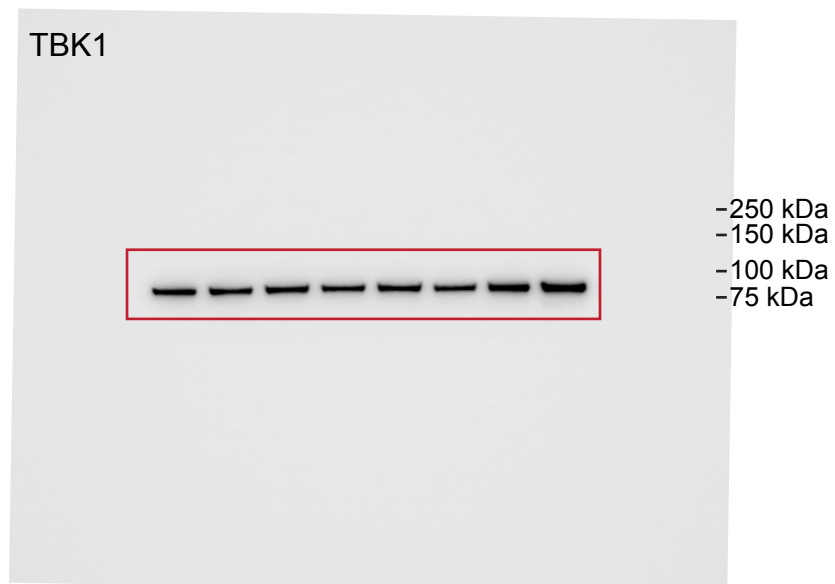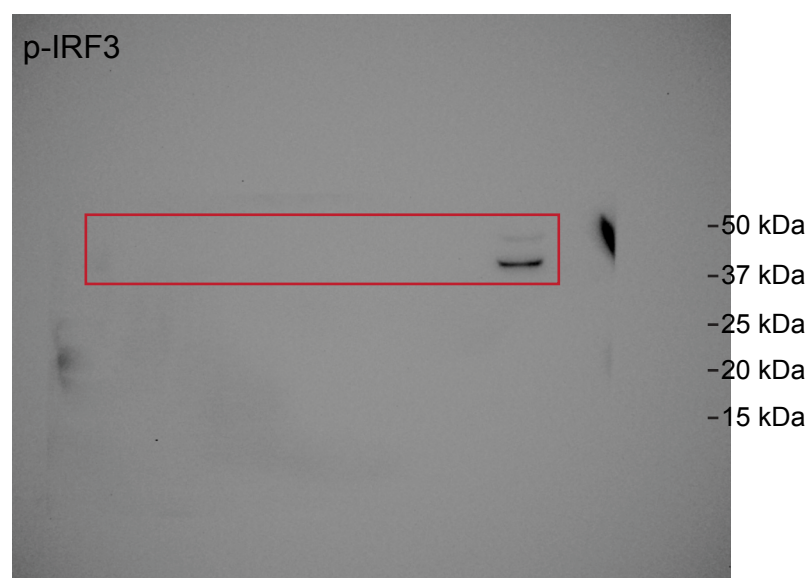

IRF3

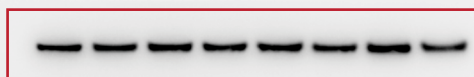

-50 kDa  
-37 kDa  
-25 kDa  
-20 kDa  
-15 kDa

HA

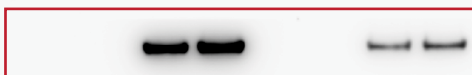

-50 kDa  
-37 kDa  
-25 kDa  
-20 kDa  
-15 kDa

Actin

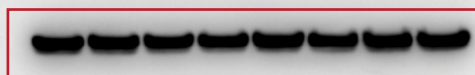

-50 kDa  
-37 kDa  
-25 kDa  
-20 kDa  
-15 kDa

Supplement: Supplementary file 17 — Source Data for Figure 7 [file EMBJ-42-e112712-s017.zip › Figure 7/Figure 7A.pdf]
