# Supplementary material for: Termination of STING responses is mediated via ESCRT‐dependent degradation
Source: EMBO J. 2023 May 4;42(12):e112712. doi: 10.15252/embj.2022112712 (PMC10267698; doi:10.15252/embj.2022112712)
Supplement: Supplementary file 11 — PDF+ [file EMBJ-42-e112712-s009.pdf]

Article

SOURCE DATA | TRANSPARENT PROCESS | OPEN ACCESS

THE EMBO JOURNAL

Termination of STING responses is mediated via ESCRT-dependent degradation

Katherine R Balka[1,*], Rajan Venkatraman[1], Tahnee L Saunders[2,3], Angus Shoppee[1,4], Ee Shan Pang[1,4], Zoe Magill[1,4], Jihane Homman-Ludiye[5], Cheng Huang[1,6], Rachael M Lane[1], Harrison M York[7], Peck Tan[1,4], Ralf B Schittenhelm[1,6], Senthil Arumugam[7,8], Benjamin T Kile[9], Meredith O'Keeffe[1,4] & Dominic De Nardo[1,**]

Abstract

cGAS-STING signalling is induced by detection of foreign or mislocalised host double-stranded (ds)DNA within the cytosol. STING acts as the major signalling hub, where it controls production of type I interferons and inflammatory cytokines. Basally, STING resides on the ER membrane. Following activation STING traffics to the Golgi to initiate downstream signalling and subsequently to endolysosomal compartments for degradation and termination of signalling. While STING is known to be degraded within lysosomes, the mechanisms controlling its delivery remain poorly defined. Here we utilised a proteomics-based approach to assess phosphorylation changes in primary murine macrophages following STING activation. This identified numerous phosphorylation events in proteins involved in intracellular and vesicular transport. We utilised high-temporal microscopy to track STING vesicular transport in live macrophages. We subsequently identified that the endosomal complexes required for transport (ESCRT) pathway detects ubiquitinated STING on vesicles, which facilitates the degradation of STING in murine macrophages. Disruption of ESCRT functionality greatly enhanced STING signalling and cytokine production, thus characterising a mechanism controlling effective termination of STING signalling.

Keywords cGAS-STING; ESCRT; innate immunity; lysosomal degradation; vesicular trafficking

Subject Categories Immunology; Microbiology, Virology & Host Pathogen Interaction

DOI 10.15252/embj.2022112712 | Received 29 September 2022 | Revised 14 April 2023 | Accepted 17 April 2023 | Published online 4 May 2023

The EMBO Journal (2023) 42: e112712

Introduction

Pathogen-derived DNA from either viruses or bacteria are highly immunogenic. Cytosolic double-stranded (ds)DNA is detected by cyclic-GMP-AMP (cGAMP) synthase (cGAS), a nucleotidyltransferase enzyme that once DNA-bound produces a second messenger cyclic dinucleotide (CDN) molecule known as 2′3′-cGAMP. 2′3′-cGAMP then acts as a ligand for Stimulator of Interferon Genes (STING), which forms a platform for innate signalling. While 2′3′-cGAMP is uniquely produced by mammalian cGAS, bacteria can produce and secrete CDN analogues (e.g. 3′3′-cGAMP, c-di-AMP) that also directly bind and activate STING. Following CDN binding, STING induces activation of transcription factors including IRF3 and NF-κB via the signalling kinases TBK1 and IKKε (Balka *et al*, 2020). The activation of such transcription factors culminates in the expression of antiviral type I interferons (IFNs) and a suite of pro-inflammatory cytokines (e.g. tumour necrosis factor [TNF], interleukin-6 [IL-6]), which are important for establishing immune responses and microbial clearance. Recently, a number of studies have highlighted the importance of STING non-IFN responses for host immunity, including during anti-viral immunity against HSV-1 (Wu *et al*, 2020; Yamashiro *et al*, 2020; Yum *et al*, 2021). Examination of STING non-IFN responses was made possible by the generation of a mutant mouse strain lacking the IRF3 binding site within STING (i.e. $\textit{Sting}^{S365A}$), rendering STING unable to induce downstream type I IFN production. These non-IFN responses, which include the production of NF-κB-dependent inflammatory cytokines, as well as induction of autophagy pathways, appear to be more primitive STING responses, conserved across organisms.

The immune functions of STING are highly dependent on its organelle location and intracellular trafficking (Balka & De

1 Department of Biochemistry and Molecular Biology, Immunity Program, Biomedicine Discovery Institute, Monash University, Clayton, Vic., Australia
2 Ubiquitin Signalling Division, Walter and Eliza Hall Institute of Medical Research, Parkville, Vic., Australia
3 Department of Medical Biology, The University of Melbourne, Melbourne, Vic., Australia
4 Department of Biochemistry and Molecular Biology, Cancer Program, Biomedicine Discovery Institute, Monash University, Clayton, Vic., Australia
5 Monash Micro Imaging, Biomedicine Discovery Institute, Monash University, Clayton, Vic., Australia
6 Monash Proteomics and Metabolomics Facility, Monash University, Clayton, Vic., Australia
7 Department of Anatomy and Developmental Biology, Biomedicine Discovery Institute, Monash University, Clayton, Vic., Australia
8 European Molecular Biological Laboratory Australia (EMBL Australia), Monash University, Clayton/Melbourne, Vic., Australia
9 Faculty of Health and Medical Sciences, The University of Adelaide, Adelaide, SA, Australia
*Corresponding author. Tel: +61 3 9905 1125; E-mail: katherine.balka@monash.edu
**Corresponding author. Tel: +61 3 9902 9557; E-mail: dominic.denardo@monash.edu

© 2023 The Authors. Published under the terms of the CC BY 4.0 license.

Nardo, 2021). In resting conditions, STING localises to the endoplasmic reticulum (ER) membrane via four transmembrane (TM) domains, existing as pre-formed dimers. Following CDN binding, STING is transported via coatomer protein complex II (COPII)-coated vesicles that transport cargo from the ER to ER-Golgi intermediate compartment (ERGIC) and Golgi (Ogawa *et al*, 2018; Gui *et al*, 2019; Ran *et al*, 2019). Interestingly, blocking STING ER-Golgi trafficking by disrupting the ADP-ribosylation factor (ARF) GTPases with either the toxin Brefeldin A or a *Shigella* effector protein inhibits STING signalling, completely blocking STING-driven inflammation (Ishikawa *et al*, 2009; Konno *et al*, 2013; Dobbs *et al*, 2015; Ni *et al*, 2017; Ogawa *et al*, 2018). Furthermore, STING must transit to the Golgi for interaction with TBK1 and downstream signalling events. Multiple proteins have now been shown to play roles in mediating STING ER to Golgi trafficking including STIM1, STEEP, iRHOM2, TMED2 and YIP5F (Luo *et al*, 2016; Sun *et al*, 2018; Ran *et al*, 2019; Srikanth *et al*, 2019; Zhang *et al*, 2020; Balka & De Nardo, 2021). At later timepoints, STING has been shown to move into endosomal regions (e.g. positive for Rab7) before it becomes degraded within the acidic environment of the lysosome (Gonugunta *et al*, 2017). Blocking acidification of the lysosome with the V-ATPase inhibitor Bafilomycin A1 or Chloroquine, has been shown to prevent STING degradation and promotes enhanced STING signalling (Gonugunta *et al*, 2017; Gui *et al*, 2019). Interestingly, co-administration of cGAMP and Bafilomycin A1 enhanced STING-induced cytokine production and was found to improve tumour clearance in a B16 melanoma transplant model compared to cGAMP alone (Gonugunta *et al*, 2017).

STING signalling outcomes are fine-tuned by precise movement through multiple organelles. However, the complexity of STING trafficking regulation provides several avenues for dysregulated trafficking to cause aberrant STING activation. Strikingly, in the context of the autoinflammatory disease termed STING-associated vasculopathy with onset in infancy (SAVI), autoactivating STING mutations mimic the structure and location of ligand-activated STING, positioning STING at the Golgi to drive constitutive signalling (Dobbs *et al*, 2015; Ergun *et al*, 2019). Interestingly, preventing recycling of STING back to the ER from the Golgi in the steady state has been shown to drive STING pathology in the context of coatamer subunit α (COPA) deficiency (Deng *et al*, 2020; Lepelley *et al*, 2020; Kato *et al*, 2021; Mukai *et al*, 2021; Steiner *et al*, 2022). Finally, impaired STING degradation has been found in models of neurological diseases including amyotrophic lateral sclerosis (ALS), Parkinson's disease and Niemann-Pick disease type C, driven by deficiency of C9orf72 (McCauley *et al*, 2020), VPS13C (Hancock-Cerutti *et al*, 2022) and Niemann-Pick type C1 (NPC1; Chu *et al*, 2021), respectively. While we now appreciate the importance of understanding STING trafficking at a mechanistic level, several questions still remain, particularly regarding the regulation of post-Golgi vesicular STING trafficking. Moreover, the mechanisms that target STING for degradation within lysosomal compartments remain poorly defined. Here we performed an unbiased mass spectrometry-based proteomics screen in primary murine macrophages to assess global phosphorylation changes following STING activation. In particular, this approach identified several novel trafficking proteins that may be important for regulating distinct STING translocation events. This prompted us to track the dynamics of STING vesicular trafficking in live macrophages using high temporal microscopy. We subsequently identified that the endosomal sorting complexes required for transport (ESCRT) pathway facilitates STING degradation. We found that the ESCRT-0 protein, hepatocyte growth factor-regulated tyrosine kinase substrate (HRS), initially detects ubiquitinated STING on endosomes leading to its degradation. By inhibiting the ESCRT pathway through depletion of HRS or via expression of a dominant negative mutant form of ATPase vacuolar protein sorting 4 homologue A (VPS4a), we observed reduced STING degradation, resulting in an increase in STING-induced signalling and enhanced immune responses. Our discovery that the ESCRT pathway mediates STING degradation sheds new light on how STING signalling is terminated in order to control inflammatory responses.

Results

Global phosphoproteomic screen reveals a prominent role for STING vesicle trafficking

We recently discovered that TBK1 and IKKε redundantly control activation of STING-dependent NF-κB in myeloid cells (Balka *et al*, 2020). In an attempt to discover further novel insights into mechanisms controlling STING signalling responses, we performed a global phosphoproteomic screen of primary mouse bone marrow-derived macrophages (BMDMs) following STING activation. To do this, we performed a time course of STING activation for 5-, 10- and 30-min with the synthetic STING ligand DMXAA (or corresponding DMSO controls). BMDMs were then lysed and peptides digested before phosphorylated peptides were enriched with titanium oxide beads (Appendix Fig S1A). A portion of peptides were also collected to examine the total proteome, where no overall differences were observed in protein levels across the time points (Appendix Fig S1B–D). Importantly, treatment groups clustered together when plotted as a Uniform Manifold Approximation and Projection (UMAP; Appendix Fig S1E). We observed numerous dynamic phosphorylation changes which were either up- or down-regulated following STING activation over time (Fig 1A–C). Of note, we identified strong phosphorylation of several known residues within STING itself following 30 min DMXAA treatment (Fig EV1A; Konno *et al*, 2013; Liu *et al*, 2015; Zhao *et al*, 2019), indicating this approach generated robust, biologically relevant data. These phosphorylation sites in STING (Ser^{354}, Ser^{357}, Ser^{365}, Thr^{375}; Fig EV1B–E) are mostly clustered within the C-terminal tail (CTT), where Ser^{365} facilitates IFN signalling responses by forming a critical residue within the IRF3 binding motif (Tanaka & Chen, 2012; Liu *et al*, 2015). We expected that many of the phosphorylation events following STING activation may comprise signalling molecules involved in the activation of STING-induced transcription factors. Indeed, we detected phosphorylation of molecules previously identified in STING signalling, including IKKβ, CYLD, OPTN and iRHOM2 (Fig EV1F–L).

While we expected the predominant phosphorylation events to be on signalling molecules, to our surprise after performing Reactome pathway analysis of all proteins with differentially regulated phosphorylation changes across all time points, the top ranked pathway identified was "Membrane Trafficking" (Fig 1D). To gain further insights into our dataset, we performed a separate gene ontology (GO) analysis of the differentially regulated phosphorylation changes, detecting terms involved in trafficking

 © 2023 The Authors

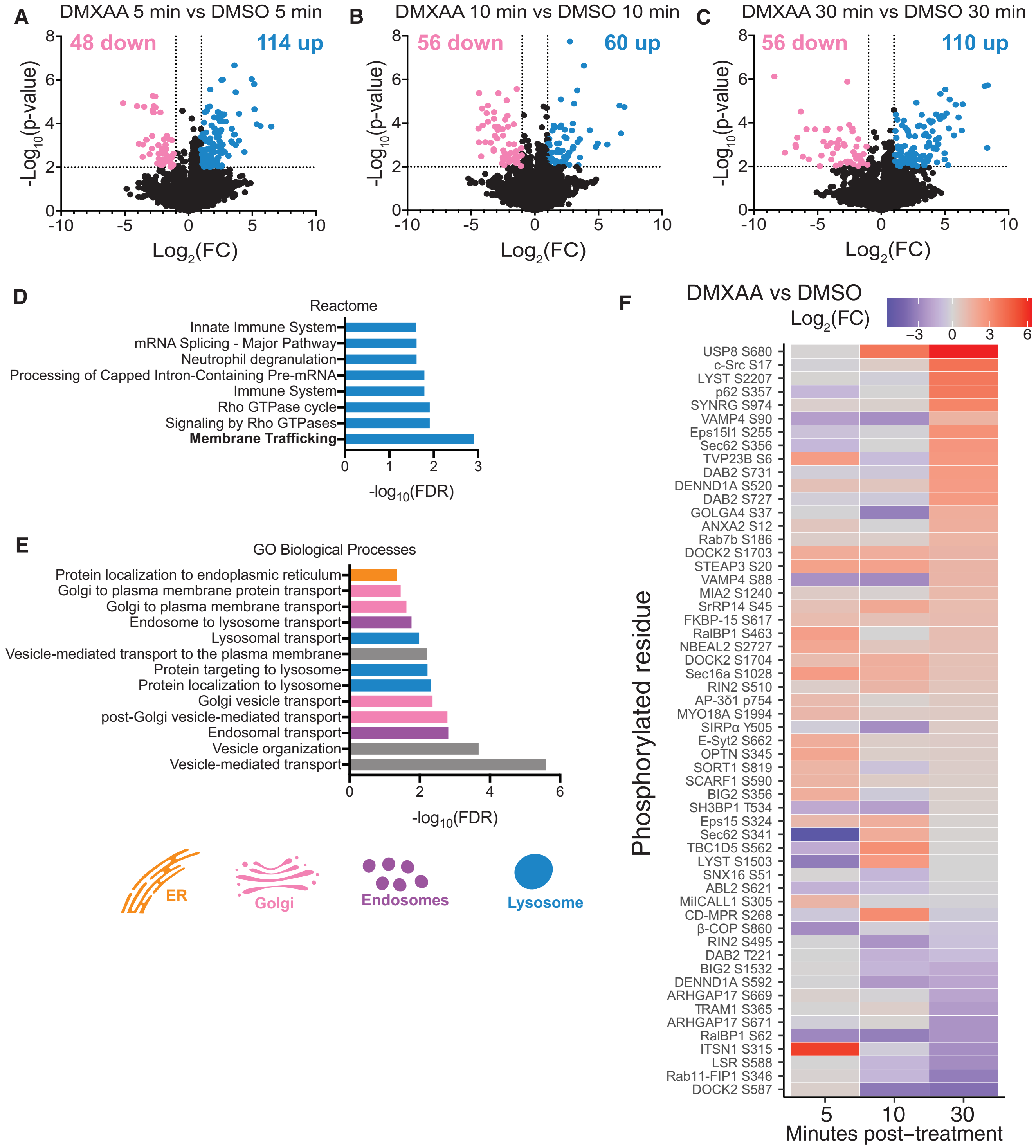
A
DMXAA 5 min vs DMSO 5 min
48 down
114 up
B
DMXAA 10 min vs DMSO 10 min
56 down
60 up
C
DMXAA 30 min vs DMSO 30 min
56 down
110 up
-Log10(p-value)
Log2(FC)
D
Reactome
Innate Immune System
mRNA Splicing - Major Pathway
Neutrophil degranulation
Processing of Capped Intron-Containing Pre-mRNA
Immune System
Rho GTPase cycle
Signaling by Rho GTPases
Membrane Trafficking
-log10(FDR)
E
GO Biological Processes
Protein localization to endoplasmic reticulum
Golgi to plasma membrane protein transport
Golgi to plasma membrane transport
Endosome to lysosome transport
Lysosomal transport
Vesicle-mediated transport to the plasma membrane
Protein targeting to lysosome
Protein localization to lysosome
Golgi vesicle transport
post-Golgi vesicle-mediated transport
Endosomal transport
Vesicle organization
Vesicle-mediated transport
-log10(FDR)
ER
Golgi
Endosomes
Lysosome
F
DMXAA vs DMSO
Log2(FC)
Phosphorylated residue
USP8 S680
c-Src S17
LYST S2207
p62 S357
SYNRG S974
VAMP4 S90
Eps15l1 S255
Sec62 S356
TVP23B S6
DAB2 S731
DENND1A S520
DAB2 S727
GOLGA4 S37
ANXA2 S12
Rab7b S186
DOCK2 S1703
STEAP3 S20
VAMP4 S88
MIA2 S1240
SrRP14 S45
FKBP-15 S617
RalBP1 S463
NBEAL2 S2727
DOCK2 S1704
Sec16a S1028
RIN2 S510
AP-3δ1 p754
MYO18A S1994
SIRPα Y505
E-Syt2 S662
OPTN S345
SORT1 S819
SCARF1 S590
BIG2 S356
SH3BP1 T534
Eps15 S324
Sec62 S341
TBC1D5 S562
LYST S1503
SNX16 S51
ABL2 S621
MICALL1 S305
CD-MPR S268
β-COP S860
RIN2 S495
DAB2 T221
BIG2 S1532
DENND1A S592
ARHGAP17 S669
TRAM1 S365
ARHGAP17 S671
RalBP1 S62
ITSN1 S315
LSR S588
Rab11-FIP1 S346
DOCK2 S587
Minutes post–treatment

Figure 1.

Figure 1. Phosphoproteomic screen uncovers numerous proteins with roles in vesicular trafficking.

A–C Volcano plots depicting significantly up (blue) and down (pink) regulated phosphorylation events for DMXAA vs. DMSO groups for 5 min (A), 10 min (B) and 30 min (C) timepoints. -Log_{10}(*P*-value) is plotted on the *y*-axis vs. Log_2(Fold change [FC]) on the *x*-axis. Significantly regulated events display *P*-value > 0.01 and Log_2(FC) > 1 or < -1.

D Pathway analysis was performed for all proteins with phosphorylation changes found across all three timepoints. Significantly enriched Reactome pathways are plotted with significance value displayed as -Log_{10}(False discovery rate [FDR]).

E Gene ontology (GO) analysis was performed for all proteins with phosphorylation changes found across all three timepoints. Numerous significant Biological Processes terms were identified. Terms related to membrane/vesicular trafficking are plotted with significance value displayed as -Log_{10}(FDR).

F The intensity of phosphorylated peptides that contributed to the GO terms in (E) was plotted in a heatmap over time. Data are shown as Log_2(FC) of DMXAA vs DMSO for each time point.

processes across the ER, Golgi, endosomal and lysosomal compartments, particularly endosome and lysosome vesicular transport (Fig 1E). These findings highlighted trafficking proteins along the known route of STING translocation. We then plotted the phosphorylation levels of proteins involved in the GO terms associated with membrane or vesicular trafficking (in Fig 1E) as a heat map, observing changes in these events in a time-dependent manner (Fig 1F). We observed strong phosphorylation changes in proteins involved in endosome and lysosomal functions, including the late endosomal marker Rab7a (Fig EV2A), which has been shown to colocalise with STING (Gonugunta *et al*, 2017), as well as, proteins involved in transport to the lysosome (CHMP4B, LYST, TOM1 [Fig EV2B–D]) and a subunit of the V-ATPase lysosomal acidifier pump (ATP6V1G3 [Fig EV2E]). We also identified phosphorylation of CD-MPR (Fig EV2F), which traffics lysosomal hydrolase enzymes from the Golgi to the lysosome. The findings from our unbiased phosphoproteomics screen revealed that a large number of proteins involved in trafficking via the Golgi, endosomes and lysosomes are modified upon STING activation (Fig EV2G). In line with recent findings that the clathrin adaptor AP-1 traffics STING from the Golgi (Liu *et al*, 2022), several of the detected proteins were also linked to clathrin-coated vesicles (Fig EV2G). Overall, our screen identified numerous proteins associated with endosome and lysosomal function that are modified upon STING activation.

Live cell imaging reveals the spatiotemporal movement of STING vesicles

STING trafficking in vesicles after exit from the Golgi is poorly understood. After identifying several phosphorylated proteins with roles in vesicular trafficking via our phosphoproteomic screen, we next wanted to examine the temporal dynamics of STING vesicle transport using live cell imaging. Therefore, we generated fluorescent STING reporter systems to perform live cell microscopy experiments enabling us to track the movement of STING in real time. To this end, we reconstituted *Sting*$^{-/-}$ immortalised (i)BMDMs with fluorescently tagged versions of murine STING (reconstituting STING-induced signalling responses, Fig EV3A) and imaged live cells using spinning disk confocal microscopy. To validate our STING reporter systems, we firstly examined the initial event in STING trafficking, which is its redistribution from the ER to the Golgi. In untreated conditions, mRuby3-STING (or eGFP-STING) localised to the ER network (Figs 2A and EV3B and Movie EV1). However, following acute STING activation with DMXAA, we could observe strong colocalisation of mRuby3-STING with the fluorescent Golgi marker, eGFP-Golgin84 (Fig 2A). We then utilised *Sting*$^{-/-}$ iBMDMs expressing eGFP-STING to track the precise kinetics of the STING ER-Golgi transition, observing STING accumulation within only 6 min of DMXAA addition, which increased steadily over time, reaching a 2.5 fold enrichment within 15 min (Figs 2B and EV3C, Appendix Fig S2A and Movies EV2 and EV3). Of note, this occurred in a linear fashion, implying a highly co-ordinated process. These data demonstrate the validity of our reporter systems. We then utilised live cell imaging of our fluorescent-STING macrophage reporters to examine vesicular STING transport post-Golgi. Within 30 min of DMXAA treatment and following enrichment in the Golgi, we started to observe a steady loss of eGFP signal from the Golgi (Fig 2C). Indeed, ~ 30 min after DMXAA stimulation we observed exit of STING from the Golgi into small vesicles (Figs 2D and EV3D and Movies EV4 and EV5). eGFP-STING vesicles emanating out of the Golgi increased rapidly with the total number of vesicles in the whole cell volume, reaching a steady state over time (Fig 2E). This correlated with the post-Golgi vesicle and endosomal transport proteins identified via the phosphoproteomic screen at 30 min (Fig 1). At later time points following STING activation (~ 80 min), we observed further accumulation of eGFP-STING vesicles within the cytosol (Fig 2F, Appendix Fig S2B and Movie EV6 and EV7). Our live cell imaging demonstrates that STING vesicles are visible from the Golgi ~ 30 min following activation and accumulate in the cytosol over time.

STING undergoes degradation following ligand activation

We observed a large number of STING positive vesicles emerging from the Golgi over time. It is known that endosomes containing STING move to the lysosomal system for degradation (Gonugunta *et al*, 2017). Having established that STING exits the Golgi early from around 30 min following DMXAA exposure, we next aimed to assess the degradation kinetics of STING in macrophages. Therefore, we activated STING in primary BMDMs over time for 2, 4 or 6 h with DMXAA (Fig 3A) or a modified version of the endogenous STING ligand (i.e. produced by cGAS) 2′3′-cGAM(PS)2 (referred to hereon as 2′3′-cGAMP; Fig 3B) and assessed STING activation and degradation by Western blot. Stimulation with DMXAA appeared to induce relatively short-lived STING activation with detectable STING phosphorylation (p-STING) at 2 h, that was no longer visible by 4 h (Fig 3A). Activation of TBK1 (i.e. p-TBK1) followed similar kinetics with peak activation at 2 h (Fig 3A). When examining total STING levels, we observed the characteristic increase and shift upward in molecular weight at 2 h, which corresponds to phosphorylated STING. Both bands were greatly depleted over time with minimal STING remaining at both 4 and 6 h post DMXAA treatment (Fig 3A). 2′3′-cGAMP stimulation led to similar, albeit more

 © 2023 The Authors

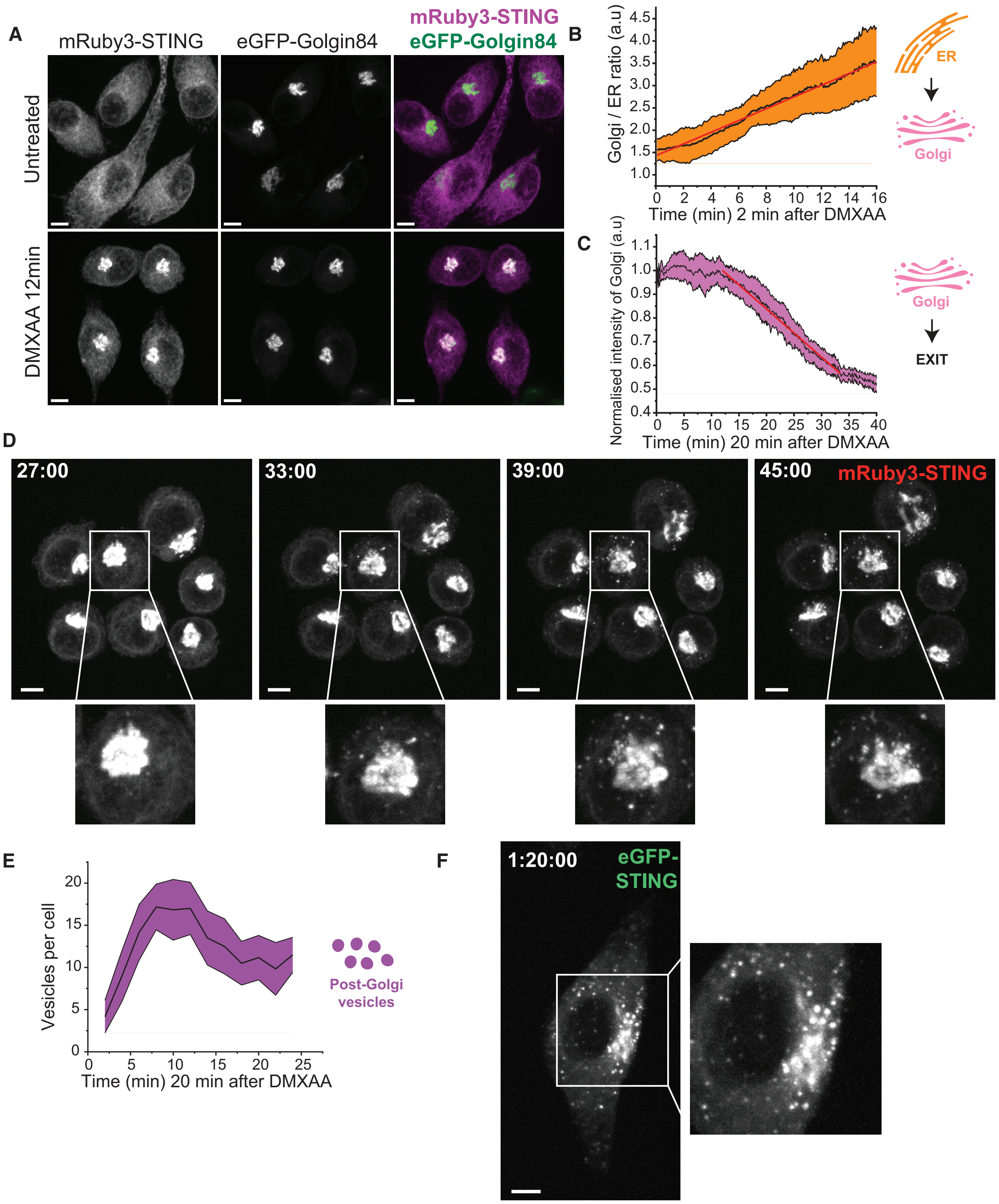
A
mRuby3-STING
eGFP-Golgin84
mRuby3-STING
eGFP-Golgin84
Untreated
DMXAA 12min
B
Golgi / ER ratio (a.u)
Time (min) 2 min after DMXAA
ER
Golgi
C
Normalised intensity of Golgi (a.u)
Time (min) 20 min after DMXAA
Golgi
EXIT
D
27:00
33:00
39:00
45:00
mRuby3-STING
E
Vesicles per cell
Time (min) 20 min after DMXAA
Post-Golgi vesicles
F
1:20:00
eGFP-STING

Figure 2.

Figure 2. STING rapidly traffics to the Golgi and exits via vesicular trafficking.

A *Sting*$^{-/-}$ iBMDMs expressing mRuby3-STING (magenta) and eGFP-Golgin84 (green) were imaged live on the 3i marianas spinning disk microscope. Z stack images were acquired either before (i.e. untreated) or after 50 μg/ml DMXAA treatment (i.e. image captured at 12 min post DMXAA treatment). Data are shown as a maximum intensity projection (MIP) of Z stack images. Scale bar = 5 μm.

B Increase in Golgi accumulation of eGFP signal starting from 2 min after 50 μg/ml DMXAA stimulation of eGFP-STING iBMDMs and presented as Golgi/ER intensity in arbitrary units (a.u.) over time. Line of best fit is shown in red. Data are shown as the mean ± SD ($N = 8$ cells) and representative of three individual experiments.

C eGFP intensity loss from the Golgi starting from 20 min after 50 μg/ml DMXAA stimulation of eGFP-STING iBMDMs and presented as intensity of the Golgi (a.u.) over time. Line of best fit is shown in red. Data are shown as the mean ± SD ($N = 12$ cells) and representative of three individual experiments.

D *Sting*$^{-/-}$ iBMDMs expressing mRuby3-STING were imaged live on the spinning disk microscope. Representative images of timelapse showing STING vesicles exiting the Golgi for a recording starting 27 min after 50 μg/ml DMXAA treatment. Data are shown as a MIP of Z stack images. Square region of interest (ROI) indicates zoomed insert. Scale bar = 5 μm. Corresponds to Movie EV4.

E eGFP-positive small vesicle regions were counted for individual cells over time starting from 20 min after 50 μg/ml DMXAA stimulation of eGFP-STING iBMDMs and presented as the number of endosomes per cell. Data are shown as the mean ± SD ($N = 11$ cells) and representative of three individual experiments.

F *Sting*$^{-/-}$ iBMDMs expressing eGFP-STING were imaged live on the spinning disk microscope. A representative image of timelapse showing transport of STING vesicles for a recording starting 1 h 18 min after 50 μg/ml DMXAA treatment. Data are shown as a MIP of Z stack images. Square ROI indicates zoomed insert. Scale bar = 5 μm. Corresponds to Movie EV6.

Source data are available online for this figure.

prolonged, p-STING and p-TBK1 signal, with slightly more delayed degradation kinetics compared to that in macrophages treated with DMXAA (Fig 3B). Interestingly, STING degradation appeared largely independent of canonical STING signalling events, as iBMDMs deficient for both TBK1 and IKKε (which cannot induce STING IFNβ nor NF-κB responses; Balka *et al*, 2020) exhibited similar levels of STING degradation following activation with DMXAA over time (Fig 3C). Of note, TBK1/IKKε^{dKO} iBMDMs induced comparable STING-dependent LC3B lipidation to WT counterparts (Fig 3D). This is in line with previous studies that have shown STING-induced LC3B lipidation remains active in the absence of STING-induced signalling responses (Gui *et al*, 2019; Liu *et al*, 2019; Wu *et al*, 2020; Yamashiro *et al*, 2020; Yum *et al*, 2021). These data demonstrate that following activation, STING becomes degraded over time in a manner independent of TBK1 and IKKε.

STING degradation is dependent on ubiquitination

While STING is known to be degraded within lysosomes, the mechanisms controlling STING vesicular transport to lysosomal compartments are largely unknown. STING has been shown to orientate on the endosomal membrane with a forward topology, where the C terminus, that facilitates STING signalling via the CTT, faces the cytosol (Gonugunta *et al*, 2017). This suggests that a model in which STING endosomes directly fuse with lysosomes may not be effective at preventing continuing STING responses. Hence, efficient degradation of STING would require its internalisation into larger acidified vacuoles. From our phosphoproteomic screen, we identified phosphorylation of the endosomal sorting complexes required for transport (ESCRT)-III component, CHMP4B (Fig EV2B), which is involved in internalising endosomal membrane proteins into multivesicular bodies (MVB) for subsequent lysosomal degradation (Vietri *et al*, 2020). We posited that STING degradation may therefore require the ESCRT pathway. The generation of MVBs initially involves the detection of ubiquitinated endosomal membrane proteins by the ESCRT-0 complex. Therefore, we hypothesised that STING degradation would be highly dependent on ubiquitination. To examine this, we utilised TAK243, a ubiquitin E1 activating enzyme inhibitor that prevents the conjugation of ubiquitin onto target sites. We found that TAK243 effectively blocked global ubiquitination in BMDMs, as we could no longer detect any ubiquitin smears via Western blot in TAK243-treated cells (Fig 4A). We then pre-incubated BMDMs with TAK243 for 30 min, before stimulating STING with DMXAA for 1, 2 or 4 h. Surprisingly, although previous studies have suggested ubiquitination events may be required for STING trafficking and signalling events (Baker *et al*, 2017; Ni *et al*, 2017), we did not observe a defect in STING signalling responses in the presence of TAK243 (Fig 4B). In fact, treatment with TAK243 led to enhanced STING signalling with increased and prolonged phosphorylation of STING, TBK1, IKKε and IRF3 (Fig 4B). Interestingly, TAK243 treatment also appeared to protect against STING degradation, where although STING levels were nearly undetectable by 4 h in the absence of TAK243, we saw no reduction in total STING protein from 1 to 4 h post DMXAA treatment in the presence of TAK243 (Fig 4B). We obtained similar results following STING activation with 2′3′-cGAMP in the presence of TAK243 (Fig 4C). We then activated STING in the absence or presence of TAK243, before fixing BMDMs and staining for phosphorylated STING. We observed similar kinetics of DMXAA-induced STING phosphorylation as via Western blot with a peak at 1 h, which was then almost undetectable at 4 h post-DMXAA treatment (Fig 4D–left panels, E and F). While the size and intensity of p-STING signal decreased from 1 to 4 h post DMXAA stimulation, in stark contrast TAK243 treated BMDMs maintained the same size and intensity of p-STING over time (Fig 4D–right panels, E and F). We next evaluated the effect of TAK243 treatment on downstream cytokine expression, observing significantly elevated gene expression of *Ifnb1*, *Isg15* and *Il6* in both DMXAA and 2′3′-cGAMP-stimulated primary BMDMs in the presence of TAK243 (Fig 4G–I). Finally, we explored whether TAK243 treatment regulated degradation and/or activation of an autoactivating mutant of STING (STING-N154S), causative for the autoinflammatory disease, STING-associated vasculopathy with onset in infancy (SAVI). We introduced either WT human (h) STING or hSTING-N154S SAVI mutant into *Sting*$^{-/-}$ iBMDMs under the control of a doxycycline (Dox)-inducible promoter. Cells were treated with Dox for 2 h to induce expression of STING before Dox was removed by washing and cells were left for 8 h in the absence or presence of TAK243. We observed that the protein expression of total hSTING-N154S was lower than that of WT-hSTING (Fig 4J), which may be due to increased degradation of the constitutively active mutant. Furthermore, we found that TAK243

 © 2023 The Authors

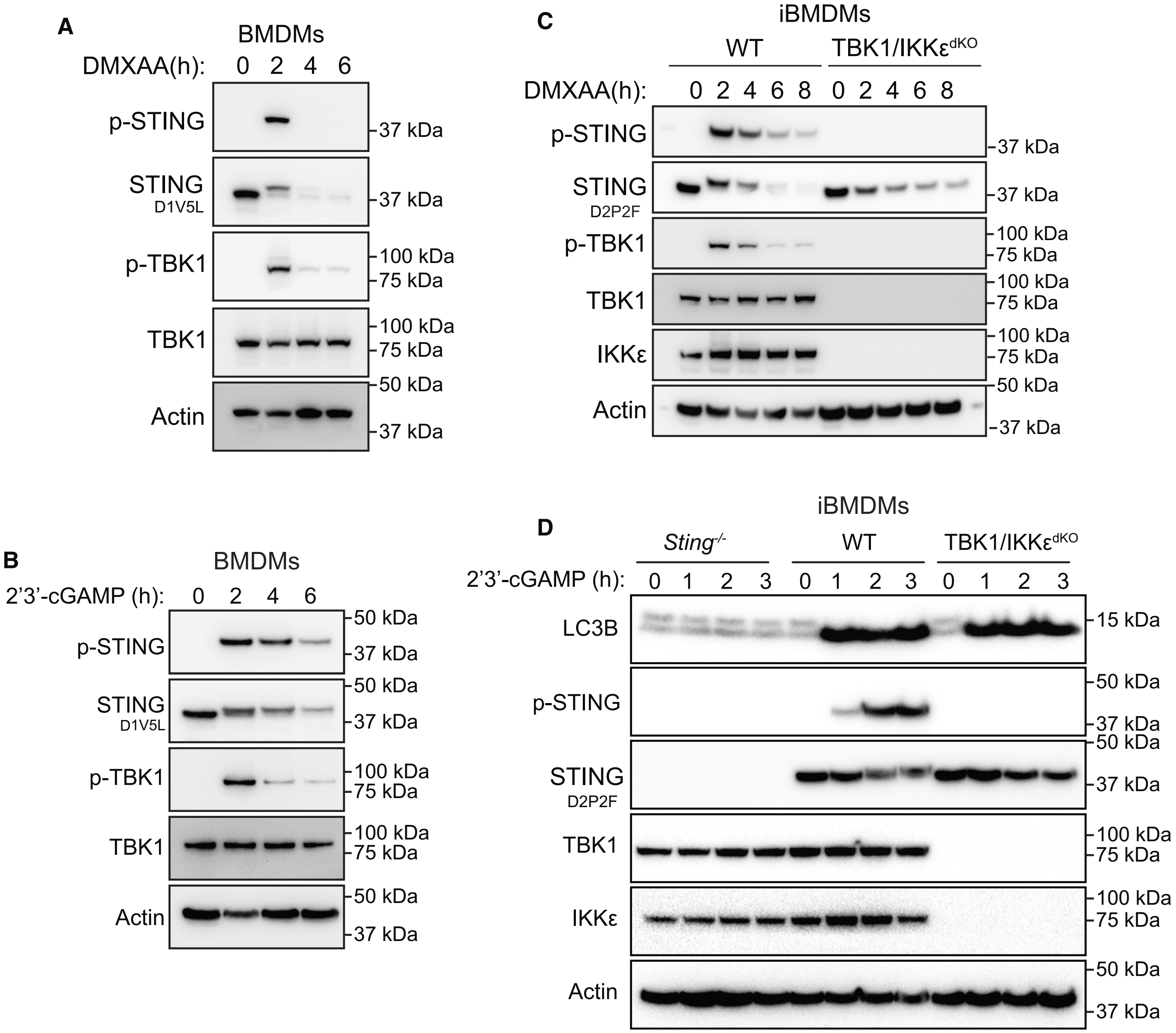

Figure 3. STING degradation is independent of TBK1/IKKε.

A Primary BMDMs were left untreated or treated with 25 μg/ml DMXAA for 2, 4 or 6 h. Cells were lysed for immunoblot with the indicated antibodies. Data shown are representative of three independent experiments.

B Primary BMDMs were left untreated or treated with 10 μg/ml 2′3′-cGAM(PS)2 for 2, 4 or 6 h. Cells were lysed for immunoblot with the indicated antibodies. Data shown are representative of three independent experiments.

C WT or TBK1/IKKε double CRISPR-Cas9 knockout (dKO) iBMDMs were left untreated or treated with 25 μg/ml DMXAA for 2, 4, 6 or 8 h. Cells were lysed for immunoblot with the indicated antibodies. Data shown are representative of three independent experiments.

D *Sting*$^{-/-}$, WT or TBK1/IKKε dKO iBMDMs were left untreated or treated with 10 μg/ml 2′3′-cGAM(PS)2 for 1, 2, or 3 h. Cells were lysed for immunoblot with the indicated antibodies. Data shown are representative of three independent experiments.

Source data are available online for this figure.

treatment appeared to increase the levels of total STING protein for both the WT and N154S forms. Strikingly, TAK243 treatment led to a large increase in phosphorylation of hSTING-N154S, suggesting ubiquitination may also act to degrade SAVI STING (Fig 4J). Therefore, together these data demonstrate that ubiquitination is crucial for STING degradation and the termination of STING signalling responses.

Ubiquitinated STING is detected by the ESCRT pathway

After confirming that ubiquitination is required for STING degradation, we hypothesised that ubiquitination of STING itself leads to detection by the ESCRT pathway. Therefore, we assessed the ubiquitination kinetics of STING following activation. To do this, we activated STING in primary BMDMs for 1.5 or 3 h and then isolated all

© 2023 The Authors

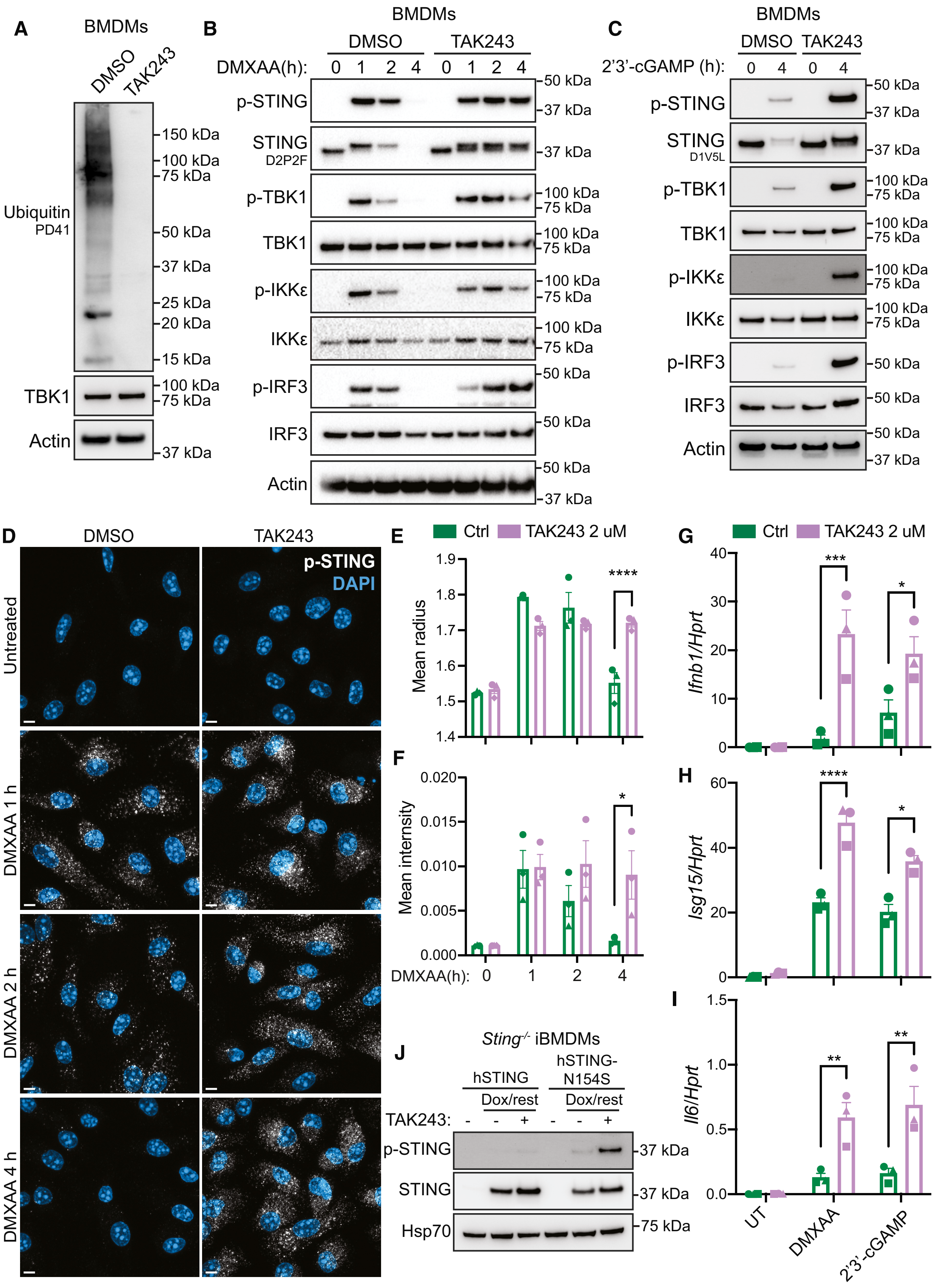

Figure 4.

© 2023 The Authors

Figure 4. STING degradation and termination of signalling is dependent on ubiquitination.

A Primary BMDMs were treated with 2 μM TAK243 or DMSO vehicle control for 4.5 h. Cells were lysed for immunoblot with the indicated antibodies. Data shown are representative of three independent experiments.

B Primary BMDMs were treated with 2 μM TAK243 or DMSO vehicle control for 30 min before being either left untreated or treated with 25 μg/ml DMXAA for 1, 2 or 4 h. Cells were lysed for immunoblot with the indicated antibodies. Data shown are representative of three independent experiments.

C Primary BMDMs were treated with 2 μM TAK243 or DMSO vehicle control for 30 min before being either left untreated or treated with 10 μg/ml 2′3′-cGAM(PS)2 for 4 h. Cells were lysed for immunoblot with the indicated antibodies. Data shown are representative of three independent experiments.

D Primary BMDMs were treated with 2 μM TAK243 or DMSO vehicle control for 30 min before being either left untreated (UT) or treated with 25 μg/ml DMXAA for 1, 2 or 4 h. Cells were fixed and stained for p-STING (white) and the nucleus (DAPI; blue), before Z stack images were acquired on the LSM980 confocal microscope. Images are displayed as a maximum intensity projection (MIP) of Z stack images. Scale bar = 5 μm. Data shown are representative of three independent experiments.

E, F The size (i.e. mean radius) (E) and mean intensity (F) of individual p-STING punctate regions was quantified using CellProfiler. Data are shown as mean ± SEM combined from $N = 3$ independent experiments. Statistical analysis was performed using two-way ANOVA using Bonferroni's multiple comparisons test, where $*P < 0.05$, $****P < 0.0001$. A representative experiment used for quantification is shown in (D).

G–I Primary BMDMs were treated with 2 μM TAK243 or DMSO vehicle control for 30 min before being either left untreated (UT) or treated with 25 μg/ml DMXAA or 10 μg/ml 2′3′-cGAM(PS)2 for 4 h. Cells were lysed for RNA purification and the expression of *Ifnb1* (G), *Isg15* (H) and *Il6* (I) was analysed by qPCR. Data are shown as mean ± SEM combined from $N = 3$ independent experiments. Statistical analysis was performed using two-way ANOVA using Bonferroni's multiple comparisons test, where $*P < 0.05$, $**P < 0.01$, $***P < 0.001$, $****P < 0.0001$.

J *Sting*$^{-/-}$ iBMDMs expressing either WT hSTING or hSTING-N154S were left untreated or treated with 1 μg/ml doxycycline (Dox) for 2 h. Cells were then washed and left to recover for a further 8 h in the absence or presence of 2 μM TAK243. Cells were lysed for immunoblot with indicated antibodies. Data shown are representative of three independent experiments.

Source data are available online for this figure.

poly-ubiquitinated proteins within total cell lysates using the tandem ubiquitin binding entities (TUBE) assay (Hjerpe *et al*, 2009). Using this method, the appearance of a ubiquitin smear upon probing with an antibody to a protein of interest suggests the protein itself is ubiquitinated (i.e. due to covalent modification of differing length ubiquitin chains). Following activation with DMXAA, we observed an enrichment in native STING (i.e. ~ 42 kDa) and the appearance of ubiquitin smears (i.e. Ub-STING), strongly suggesting that STING becomes directly poly-ubiquitinated following activation (Fig 5A). We also observed similar levels of ubiquitinated STING in both WT and TBK1/IKKε^{dKO} iBMDMs, suggesting STING ubiquitination is largely independent of canonical signalling events (Fig EV4A). Next, we wanted to determine whether ubiquitinated STING was detected by the ESCRT-0 complex, in particular focusing on the subunit HRS, that detects ubiquitinated cargo on the endosomal membrane via a ubiquitin interacting motif (UIM; Williams & Urbe, 2007). Therefore, we utilised *Sting*$^{-/-}$ iBMDMs reconstituted with a HA-tagged version of STING and performed HA immunoprecipitation (IP) following DMXAA treatment. We detected the interaction of STING with the ESCRT-0 subunit HRS, which increased over time (Fig 5B), in a STING-dependent manner (Fig EV4B). As expected, we also observed the interaction of TBK1 with STING upon DMXAA treatment (Fig 5B). As HRS interacts with cargo via its UIM, we next performed endogenous STING IP experiments in cell lysates generated from primary BMDMs in the absence or presence of TAK243. Following 3 h DMXAA treatment we observed STING interaction with HRS, which was blunted in the presence of TAK243 (Fig EV4C). To further assess STING ubiquitination and interaction with HRS, we activated eGFP-STING-expressing *Sting*$^{-/-}$ iBMDMs with DMXAA over time and performed co-immunofluorescence staining with antibodies that detect conjugated ubiquitin (Ub, FK2 clone) and endogenous HRS (Fig 5C and Appendix Fig S3). We identified strong colocalization of ubiquitin and STING following DMXAA treatment that increased over time (Figs 5D–F and EV4D and Appendix Fig S4A–D). Many of the eGFP-STING/Ub punctate regions observed were also positive for HRS staining (Figs 5D–F and EV4D and Appendix Fig S4A–D). Strikingly, treatment with TAK243 blocked all conjugated Ub signal and disrupted the localisation of HRS (Appendix Fig S3–bottom panels). Together these data demonstrate that ubiquitinated STING is targeted for recognition by the ESCRT pathway through interaction with ESCRT-0 protein, HRS.

HRS depletion drives enhanced basal and ligand-induced STING responses

After confirming HRS interacts with STING via a ubiquitin-dependent mechanism, we next examined the role of the ESCRT pathway in shutting off STING signalling. To do this we utilised CRISPR interference (CRISPRi) to deplete HRS expression. iBMDMs underwent lentiviral transduction with catalytically dead Cas9 (dCas9) fused to the Krüppel-associated box (KRAB) transcriptional repressor, along with three Dox-inducible single guide (sg) RNAs designed to target the HRS promoter. Following Dox treatment for 48 h to induce sgRNA expression and HRS depletion, iBMDMs were subsequently stimulated with the STING ligands, DMXAA and 2′3′-cGAMP for 6 h before Western blot analysis. Although HRS expression was not fully depleted with any of the three sgRNAs, we observed increased phosphorylation of STING, TBK1 and IRF3 for sg2 and sg3 (Fig 6A). We further assessed STING-induced cytokine production after 6 h DMXAA or 2′3′-cGAMP stimulation across the dCas9-KRAB and each sgRNA iBMDM line. We observed increased IFNβ and/or IL-6 production in HRS sg2 and sg3 compared to control dCas9-KRAB expressing iBMDMs (Fig 6B and C). Importantly, we observed no difference in TLR4 or TLR1/2 signalling nor cytokine production across cell lines following LPS or Pam3CSK4 treatment, respectively (Fig EV5A–C). Furthermore, as low-level STING trafficking is thought to regulate basal STING turnover (Deng *et al*, 2020; Lepelley *et al*, 2020; Chu *et al*, 2021; Kato *et al*, 2021; Mukai *et al*, 2021; Steiner *et al*, 2022; Tu *et al*, 2022), we hypothesised that HRS depletion could lead to accumulation of non-degraded STING and enhance STING activity in the absence of exogenous ligand. To investigate this, we performed qPCR analysis of STING-

© 2023 The Authors

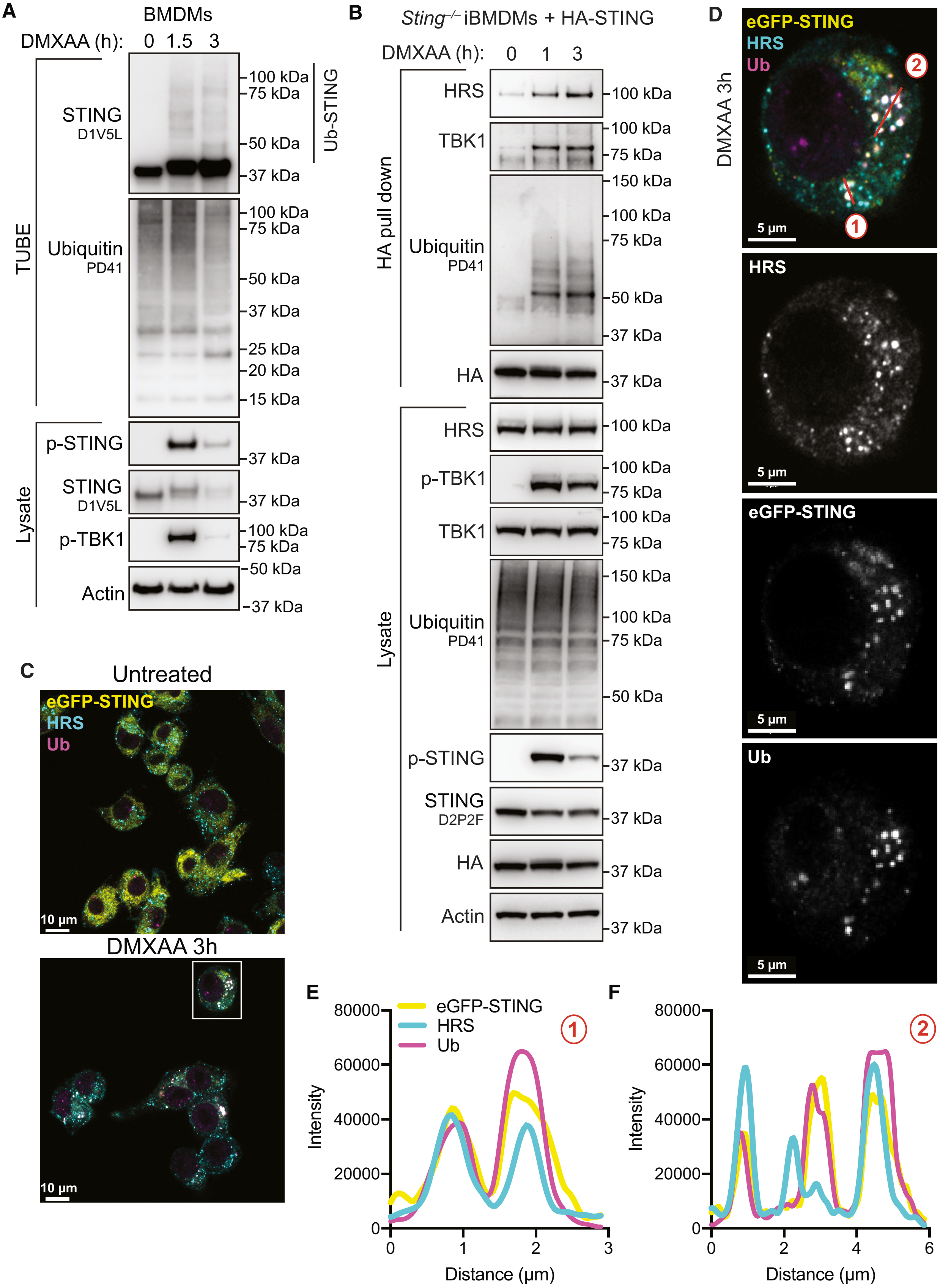

Figure 5.

© 2023 The Authors

Figure 5. ESCRT-0 HRS interacts with ubiquitinated STING.

A Primary BMDMs were left untreated or treated with 50 μg/ml DMXAA for 1.5 or 3 h. Cells were lysed and a portion of lysate underwent immunoblot with the indicated antibodies (i.e. lysate). The remaining lysate was incubated with TUBE beads to isolate ubiquitinated proteins. TUBE samples underwent immunoblot with the indicated antibodies. Data are representative of two independent experiments.

B *Sting*$^{-/-}$ iBMDMs expressing HA-STING were left UT or treated with 50 μg/ml DMXAA for 1 or 3 h. Cells were lysed and a portion of lysate underwent immunoblot with the indicated antibodies (i.e. lysate). The remaining lysate underwent immunoprecipitation with an anti-HA antibody (i.e. HA pull down). Samples then underwent immunoblot with the indicated antibodies. Data representative of three independent experiments.

C *Sting*$^{-/-}$ iBMDMs expressing eGFP-STING (yellow) were left UT or treated with 50 μg/ml DMXAA for 3 h. Cells were fixed and underwent immunofluorescence staining for conjugated ubiquitin (Ub; magenta) and HRS (cyan). Z stack images were acquired on the LSM980 confocal microscope. Images are displayed as merged images for a single Z slice. Scale bar = 10 μm. Data shown are representative of three independent experiments.

D Square ROI indicates zoomed insert from (C), displayed as both merged and single colour images. Scale bar = 5 μm. Two colour merged images can be found in Fig EV4D.

E, F The intensity profiles were plotted for each channel for two-line regions as indicated in (D).

Data information: An additional example of STING/Ub/HRS colocalisation can be found in Appendix Fig S4.
Source data are available online for this figure.

induced cytokines following HRS-depletion alone. We observed that HRS-depleted iBMDMs exhibited increased basal expression of *Ifnb1* and the IFN-stimulated genes (ISGs) *Isg15* and *Irf7* (Fig 6D–F). Furthermore, this tonic IFN activity was blocked by treatment with the STING inhibitor H-151 (Fig 6D–F). Interestingly, we saw no significant basal increase in expression of pro-inflammatory cytokine *Il6* (Fig EV5D). This is in contrast to ligand-activated STING (especially DMXAA), where we found a more pronounced increase in IL-6, compared to IFNβ production, in HRS-depleted cells (Fig 6B and C). Overall, perturbation of the ESCRT pathway leads to enhanced STING responses both basally and following ligand-induced STING activation.

Blocking the ESCRT pathway leads to heightened STING responses

VPS4a is a AAA-type ATPase, that forms a critical component of the ESCRT machinery. There are currently no known pharmacological inhibitors of the ESCRT pathway; however, overexpression of a dominant-negative mutant form of VPS4a (i.e. VPS4a^{E228Q}) is commonly used to interfere with ESCRT-dependent processes (Babst *et al*, 1998). Therefore, we next used a viral transduction system to express Dox-inducible HA-tagged VPS4a^{WT} or dominant-negative VPS4a^{E228Q} in primary BMDMs. Cells were then left untreated or treated with Dox for 4 h to induce expression, followed by 4 h stimulation with DMXAA (Fig 7A) or 2′3′-cGAMP (Fig 7B). We first examined STING protein levels and downstream signalling responses via Western blot, where we clearly observed highly increased phosphorylation of STING, TBK1 and IRF3 in the STING-activated BMDMs expressing VPS4a^{E228Q} compared to VPS4a^{WT} control cells (Fig 7A and B). In addition, we noted a rescue in the amounts of total STING following activation in BMDMs expressing VPS4a^{E228Q}, albeit lower than basal STING expression (Fig 7A and B). The increase in STING levels and downstream signalling responses correlated with a trend in increased IFNβ secretion (Fig 7C) and significantly greater levels of IL-6 (Figs 7D and EV5E) cytokine production upon STING activation in BMDMs expressing VPS4a^{E228Q} compared to VPS4a^{WT} counterparts. Together these data demonstrate that interference with the ESCRT pathway disrupts STING degradation and promotes enhanced immune signalling and cytokine responses.

Discussion

Fine-tuning STING signalling is important for mounting an appropriate immune response to infection. Here we discover a new model for the degradation of STING and the shutdown of STING signalling via the ESCRT pathway (Fig 7E). STING has previously been shown via electron microscopy to localise to endosomal membranes with its C terminus, that facilitates TBK1 and IRF3 recruitment, facing the cytosol (Gonugunta *et al*, 2017). The ESCRT pathway internalises endosomal membrane cargo into MVBs via sequential interactions of ESCRT-0 to -III complexes and VPS4a on the cytosol-facing regions of membrane cargo (Vietri *et al*, 2020). The ESCRT-0 component HRS contains VHS and FYVE domains for interaction with endosomal membranes, as well as a UIM for detecting ubiquitinated cargo. Here we show that HRS binds to STING following activation in a ubiquitin-dependent manner. Following detection, STING would therefore become internalised into intraluminal vesicles (ILVs) within MVBs. Thus, upon fusion of MVBs to lysosomes, STING would be efficiently degraded inside the acidified lysosomal environment. In support of our findings, two independent groups also recently found a role for ESCRT machinery in STING degradation (Gentili *et al*, 2023; Kuchitsu *et al*, 2023). Both studies utilised unique screening approaches, ultimately identifying that ESCRT machinery regulates basal and/or ligand-activated STING degradation in several models utilising human and mouse immune cells and fibroblasts (Gentili *et al*, 2023; Kuchitsu *et al*, 2023), nicely correlating with our findings from murine macrophages. Strikingly, Kuchitsu *et al*, observed clusters of individual STING vesicles within lysosomal membranes via correlative light and electron microscopy (CLEM), distinctive of MVBs (Kuchitsu *et al*, 2023). A limitation of our study is we did not identify the lysine (K) residue(s) required for STING degradation. However, it should be noted that in the study by Kuchitsu *et al* (2023), the authors identified that ubiquitination of K288 drove STING degradation. In contrast, Gentili *et al* (2023), found that mutation of five separate STING lysine residues was required to prevent STING interaction with ESCRT and subsequent degradation. Regardless, together these data demonstrate STING ubiquitination is required for its degradation via ESCRT.

Here we inhibited ESCRT machinery via HRS depletion or expression of a dominant-negative form of VPS4a observing that these

© 2023 The Authors

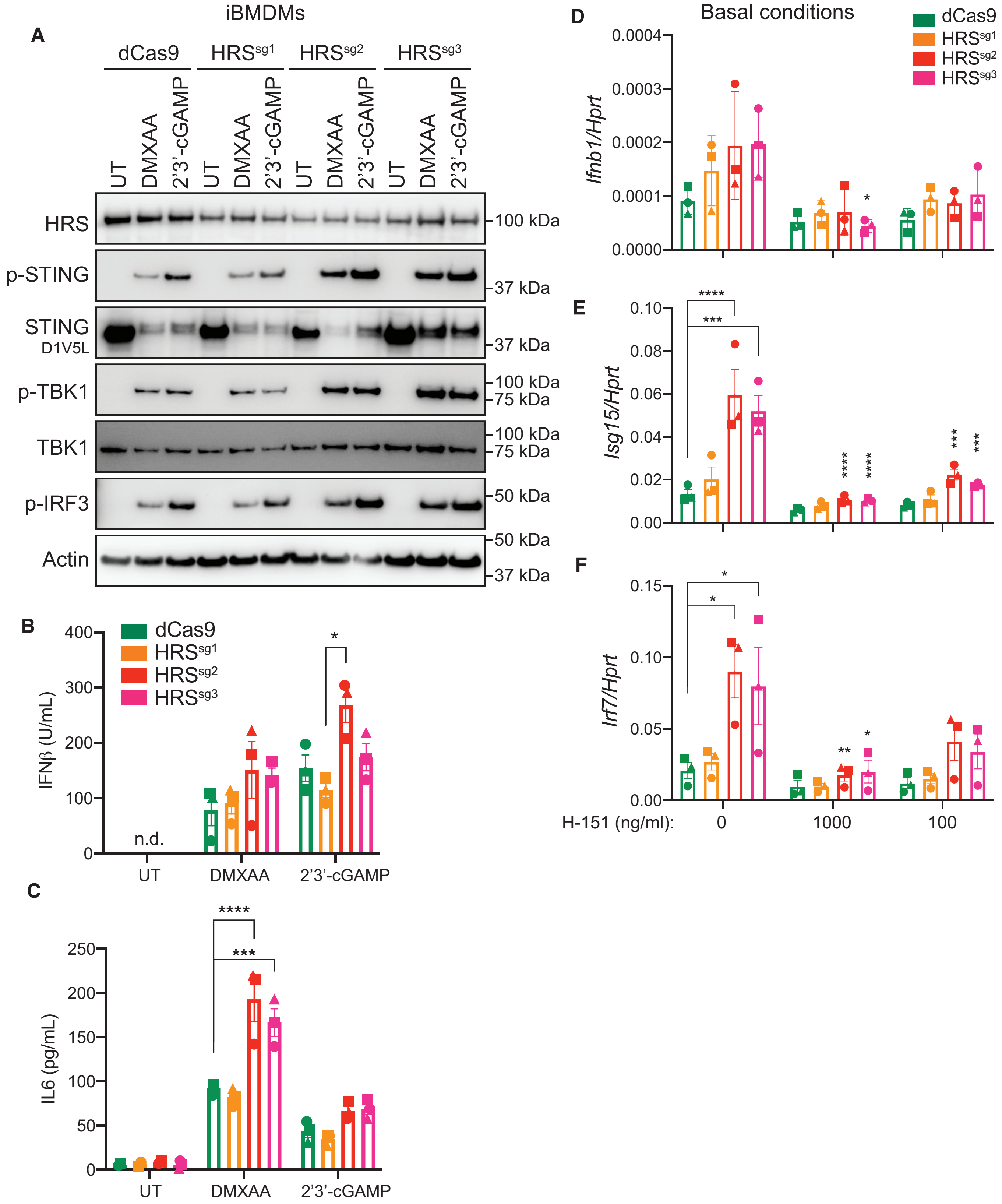

Figure 6.

 © 2023 The Authors

Figure 6. HRS depletion substantiates increased STING responses.

A dCas9-KRAB expressing iBMDMs, without (i.e. dCas9 alone) or with HRS-targeting sgRNAs (i.e., sg1, sg2 or sg3) were treated for 48 h with 1 μg/ml doxycycline (Dox) to induce sgRNA expression. Cells were then left untreated (UT), or treated with 20 μg/ml DMXAA or 10 μg/ml 2′3′-cGAM(PS)2 for 6 h. Cells were lysed for immunoblot with the indicated antibodies. Data shown are representative of three independent experiments.

B, C dCas9-KRAB expressing iBMDMs, without (i.e. dCas9 alone) or with HRS-targeting sgRNAs (i.e. sg1, sg2 or sg3) were treated for 48 h with 1 μg/ml Dox to induce sgRNA expression. Cells were then left UT, or treated with 20 μg/ml DMXAA or 5 μg/ml 2′3′-cGAM(PS)2 for 6 h. Cell supernatant was collected and assayed for secreted IFNβ (B) and IL6 (C) by ELISA. Data are shown as mean ± SEM combined from $N = 3$ independent experiments. Statistical analysis was performed using two-way ANOVA using Bonferroni's multiple comparisons test, where $*P < 0.05$, $***P < 0.001$, $****P < 0.0001$. n.d., not detected.

D–F dCas9-KRAB expressing iBMDMs, without (i.e. dCas9 alone) or with HRS-targeting sgRNAs (i.e. sg1, sg2 or sg3) were treated for 24 h with 1 μg/ml Dox to induce sgRNA expression. iBMDMs were further treated with 1 μg/ml (i.e. 1,000 ng/ml) or 100 ng/ml H-151 as indicated and incubated for a further 24 h. Cells were lysed for RNA purification and the expression of *Ifnb1* (D), *Isg15* (E) and *Irf7* (F) was analysed by qPCR. Data are shown as mean ± SEM combined from $N = 3$ independent experiments. Statistical analysis was performed using two-way ANOVA using Tukey's multiple comparisons test, where $*P < 0.05$, $**P < 0.01$, $***P < 0.001$, $****P < 0.0001$. Bars display significant differences between dCas9 and sgRNA knockdowns in the absence of H-151. Additional asterisks represent significant differences between the absence or presence of H-151 across the same cell line.

Source data are available online for this figure.

approaches prevented STING degradation and enhanced STING signalling. It is worth noting that our knockdown of HRS protein was incomplete and led to a less prominent phenotype compared to expression of dominant-negative VPS4a. This is in line with other recent studies that likewise found knockdown of HRS had less of an effect on STING degradation and immune responses compared to targeting of other ESCRT components (e.g.VSP37, TSG101; Gentili *et al*, 2023; Kuchitsu *et al*, 2023). Of note however, ESCRT perturbation does not appear to provide a complete rescue in STING protein levels. Therefore, multiple mechanisms may exist to tightly regulate STING degradation. For example, Prabakaran *et al* (2018), previously reported p62-mediated autophagy-controlled STING degradation in murine embryonic fibroblasts (MEFs) and THP-1 monocytes, where p62 knockouts similarly exhibited heightened STING responses. Conversely, others have shown STING degradation is independent of several canonical autophagy components (e.g. Atg-3, -5, -7 and -9; Gonugunta *et al*, 2017; Gui *et al*, 2019; Kuchitsu *et al*, 2023). Therefore, further studies are required to define whether it is a balance between autophagy, trafficking cofactors (such as NPC1 – see below) and ESCRT-mediated lysosomal delivery that controls maximal STING degradation. Furthermore, it is also not fully understood when the STING signalling complex (i.e. STING-TBK1/IKKε-IRF3) is dismantled and whether this precedes the movement of STING into larger degradative compartments. Future studies will be important to assess whether this balance may be overturned in distinct cell types, where certain degradative pathways (i.e. MVBs) may be crucially important for STING degradation.

Interestingly, the activation and termination of STING signalling via trafficking-facilitated degradation is controlled via transit through multiple organelles. These complex trafficking events require tight regulation as multiple diseases have now been linked to dysregulation of STING localisation resulting in enhanced immune responses. The assumption remains that STING trafficking is held in an equilibrium, where basal STING turnover is also similarly controlled via trafficking to lysosomes. Disruption or mutations in proteins controlling STING cycling can offset this equilibrium, ultimately leading to the accumulation of STING within active sites at the ERGIC, Golgi and endosomes, which can propagate inflammation. In particular, inactivating mutations in coatomer subunit α (COPA), which mediates retrograde transport from the Golgi to ER, have been shown to drive STING-dependent inflammation in COPA syndrome (Deng *et al*, 2020; Lepelley *et al*, 2020; Kato *et al*, 2021; Mukai *et al*, 2021; Steiner *et al*, 2022). In this context, blocking the recycling of STING back to the ER in steady state conditions leads to accumulation of STING at the Golgi and the propagation of STING signalling events. Interestingly, the inflammation caused by COPA deficiency was rescued by both STING or cGAS depletion, suggesting low level basal cGAS-mediated 2′3′-cGAMP production and STING activation stimulates STING turnover (Lepelley *et al*, 2020; Steiner *et al*, 2022). Additionally, a break on lysosomal degradation of STING can also lead to heightened STING signalling and drive disease. McCauley *et al* (2020), reported that in a murine model of amyotrophic lateral sclerosis (ALS), C9orf72 deficiency led to impaired degradation of STING and elevated STING-dependent inflammation. Furthermore, a lipid trafficking protein VPS13C, which is found to be mutated in early onset Parkinson's disease has also been linked to aberrant STING activity (Hancock-Cerutti *et al*, 2022). VPS13C-depleted HeLa cells display both increased cytosolic mtDNA, as well as a disrupted lysosomal system and impaired STING degradation (Hancock-Cerutti *et al*, 2022). Finally, NPC1 has also been described as a trafficking cofactor of STING that delivers STING to the lysosome (Chu *et al*, 2021). NPC1-deficient mice recapitulate the neurological condition Niemann-Pick disease type C. However, crossing to STING-deficient mice rescues the neuroinflammation and neuropathology observed in NPC1-deficient mice (Chu *et al*, 2021). These studies highlight the importance of controlled STING degradation for prevention of human diseases, particularly in the context of neuroinflammation, where offsetting the balance of STING turnover can develop into STING-driven inflammation and disease.

Here, we show that blocking the ESCRT pathway genetically or via overexpression of dominant-negative VPS4a led to enhanced STING signalling and cytokine output. Ultimately, our data suggest that STING activation could be boosted in the context of an underfunctioning ESCRT pathway (Fig 7E). Firstly, while HRS knockout mice are embryonically lethal (day E11), mice harbouring a mutation in the VHS domain of HRS present with a progressive neurological disease, reduced motor function, reduced size and only survive up to 5 weeks (Komada & Soriano, 1999; Watson *et al*, 2015). Mice deficient for VPS4a are also embryonically lethal, while heterozygous MEFs display disrupted endolysosomal morphology (Drusenheimer *et al*, 2015). Patient mutations have been described in VPS4a, as well as the ESCRT-III proteins, CHMP1A and CHMP4B across varying disease phenotypes (Shiels *et al*, 2007; Mochida

© 2023 The Authors

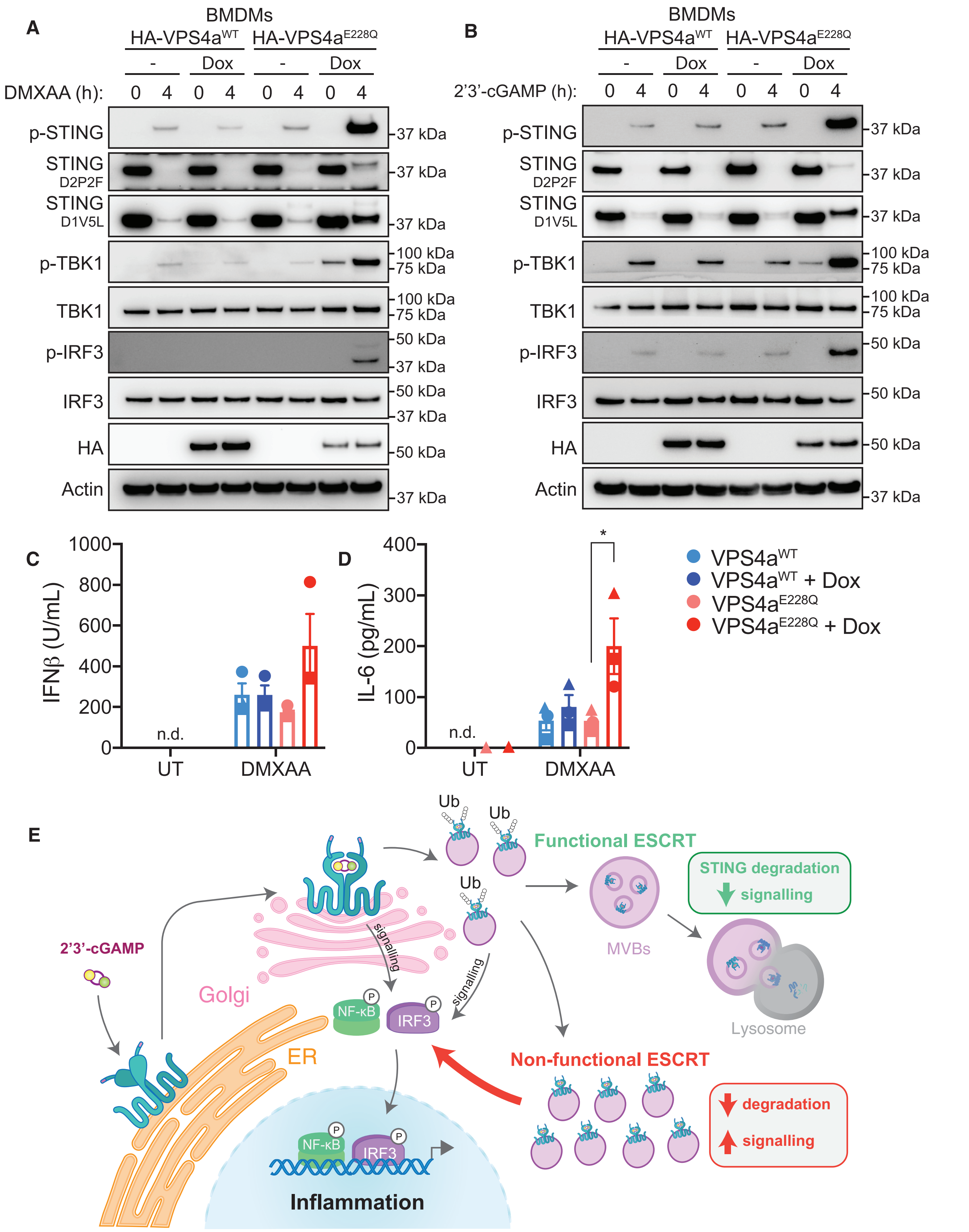

Figure 7.

© 2023 The Authors

Figure 7. The ESCRT pathway is required for effective termination of STING responses.

A, B VPS4a[WT] or VPS4a[E228Q] dominant negative BMDMs were left untreated (UT) or treated with 1 μg/ml doxycycline (Dox) for 4 h. BMDMs were then further left UT or treated with 25 μg/ml DMXAA (A) or 10 μg/ml 2′3′-cGAM(PS)2 (B) for 4 h. Cells were lysed for immunoblot with the indicated antibodies. Data shown are representative of 2 independent experiments for (A) and 3 independent experiments for (B).

C, D VPS4a[WT] or VPS4a[E228Q] dominant negative BMDMs were left UT or treated with 1 μg/ml Dox for 4 h. BMDMs were then further left UT or treated with 25 μg/ml DMXAA for 4 h. Cell supernatant was collected and secreted IFNβ (C) or IL-6 (D) was measured by ELISA. Data are shown as mean ± SEM combined from $N = 3$ independent experiments. Statistical analysis was performed using one-way ANOVA. using Bonferroni's multiple comparions test, where $^{*}P < 0.01$. n.d., not detected.

E A proposed model for ESCRT-dependent degradation and termination of STING-mediated immune responses.

Source data are available online for this figure.

et al, 2012; Rodger et al, 2020). In particular, VPS4a-associated disease, presents as a severe neurodevelopmental disease. Interestingly, Gentili et al (2023), modelled a dominant-negative patient mutation in the ESCRT protein UBAP1 which causes hereditary spastic paraplegia. Expression of the UBAP1 mutant led to disruption of basal STING degradation and increased STING-dependent inflammation. In addition to mediating lysosomal degradation, ESCRT-III and VPS4a play roles in closing the nuclear envelope (NE), as well as spindle disassembly following mitosis (Olmos et al, 2015; Vietri et al, 2015). Rodger et al (2020), also discovered that patient neurons harbouring VPS4a mutations displayed abnormal nuclear morphology, increased γH2AX DNA damage and micronuclei formation. CHMP4B and VPS4b have further been shown to be recruited to sites of NE rupture in migrating cells to reseal the NE (Denais et al, 2016; Raab et al, 2016). Of note, cGAS also rapidly associated with damaged DNA at NE rupture sites (Raab et al, 2016). Therefore, together these observations suggest that VPS4a and/or additional ESCRT mutations may have the potential to provoke both cGAS activation, as well as impede STING degradation, providing an opportunity for strong aberrant STING inflammation. However, further studies are required to assess if cGAS is activated in the context of ESCRT/VPS4a deficiency. Additionally, mutations in Lysosomal trafficking regulator (LYST), which we found to be strongly phosphorylated at 30 min post DMXAA treatment (Fig EV2C) are causative in an immunodeficiency disease known as Chediak-Higashi syndrome (CHS) (Windhorst et al, 1966; Burkhardt et al, 1993; Barbosa et al, 1996; Sepulveda et al, 2015). LYST deficiency in either mice or through mutations seen in patients is characterised by enlarged lysosomes and disruption of endolysosomal trafficking (Windhorst et al, 1966; Burkhardt et al, 1993; Barbosa et al, 1996; Sepulveda et al, 2015), analogous to VPS4a and ESCRT mutants. Interestingly, TRIF-dependent TLR3/4 signalling in endosomes was found to be disrupted in cells derived from CHS LYST-mutant mice, which exhibited inhibited cytokine production (Westphal et al, 2017). Therefore, it would be interesting to further assess STING activation and degradation in the context of ESCRT and lysosomal trafficking defects, where additional disease mechanisms may be identified. Conversely, Gonugunta et al (2017), also provided proof of principle evidence that blocking STING degradation to boost STING signalling can also be exploited for therapeutic benefit, by demonstrating that co-administration of cGAMP and the lysosomal inhibitor, Bafilomycin A1, improved tumour clearance over cGAMP alone in a murine melanoma model. We speculate that blocking the interaction between STING and ESCRT components and/or ubiquitin may provide a more specific avenue for enhancing STING responses for improved therapeutic outcomes. Our phosphoproteomic screen and discovery of ESCRT-mediated STING degradation open up important new questions regarding the regulation of STING trafficking and degradation. Identifying the precise mechanisms that limit STING signalling is becoming increasingly important for understanding a growing number of diseases, where balancing STING degradation may constrain unwarranted STING-dependent inflammation.

Materials and Methods

General reagents

DMSO (Sigma-Aldrich #D2650), DMXAA (SelleckChem #S1537), 2′3′-cGAM(PS)2 (InvivoGen #tlrl-nacga2srs), LPS-B5 Ultrapure (InvivoGen #tlrl-pb5lps), Pam3CSK4 (InvivoGen #tltl-pms), TAK243 (SelleckChem #S8341), H-151 (InvivoGen #inh-h151), doxycycline (Sigma-Aldrich #D9891).

Animals

C57BL/6 *Sting*$^{+/+}$ and *Sting*$^{-/-}$ mice as previously reported (Jin et al, 2011) were housed under specific pathogen-free conditions at Monash University. All procedures involving mice were approved by the Monash University Animal Ethics Committee (#15294, #28073).

Primary murine bone marrow-derived macrophages (BMDMs)

Bone marrow cells were harvested from femurs and tibias of 6- to 8-week-old male or female C57BL/6 mice and cultured with complete Dulbecco's Modified Eagle Medium (DMEM, made in house) supplemented with 20% L929 conditioned media for 6 days at 37°C in humidified atmosphere with 10% CO_2 to generate primary bone marrow-derived macrophages (BMDMs).

Cell lines

Human Embryonic Kidney 293T (HEK293T) cells and immortalised (i)BMDMs were cultured in complete DMEM at 37°C in 10% CO_2 humidified atmosphere and passaged every 2–3 days. Wildtype, *Sting*$^{-/-}$ iBMDMs (De Nardo et al, 2018b) and TBK1/IKKε CRISPR knockout iBMDMs (Balka et al, 2020) were generated as previously described.

CRISPR/Cas9 gene editing

HRS depletion was performed via CRISPR interference (CRISPRi) using CRISPR/Cas9 gene editing as previously described (Baker &

Masters, 2018). Third-generation lentiviral transduction (see below) was used to generate iBMDMs expressing blasticidin-selectable dCas9-KRAB, which were selected with 5 µg/ml blasticidin for ~ 72 h. CRISPRi sgRNA guides were designed using the Broad Institute CRISPick online tool (https://portals.broadinstitute.org/gppx/crispick/public) and cloned into the doxycycline-inducible sgRNA plasmid pFgH1tUT, expressing GFP (see below). Following lentiviral transduction (see below) of sgRNAs into dCas9-KRAB iBMDMs, cells were sorted for GFP-positive cells via flow cytometry using a BD Biosciences Influx™ cell sorter. iBMDMs were treated with 1 µg/ml doxycycline to induce HRS depletion as indicated in the figure legends.

Generation of lentiviral plasmids

All primers were ordered from Integrated DNA Technologies (IDT). The second-generation lentiviral plasmids pLVX-mRuby3-mSTING, pLVX-eGFP-mSTING and pLVX-mSTING were generated by Gibson Assembly utilising the backbone from pLVX-EF1a-EGFP-TA-Golgin84-IRES-Puromycin (Addgene #133028) between cloning sites *XbaI* and *BamHI*. The online tool NEBuilder was used to design all primers for Gibson Assembly cloning. A HA-tag was introduced to the N terminus of STING in pLVX-mSTING via Q5 mutagenesis to generate pLVX-HA-mSTING. pTRIPz-hSTING (ΔAgeI) was used as previously described (Balka *et al*, 2020). pTRIPz-hSTING-N154S was generated using Q5 mutagenesis. NEBasechanger was used to design primers for Q5 mutagenesis. Third-generation sgRNA plasmids for HRS were generated by annealing sgRNA oligo guide sequences (sg1: 5′-tcccTCCGGAGTGGGGTCGCCATG-3′, 5′-aaacCATGGCGACCCCACTCCGGA -3′, sg2: 5′-tcccTGCAGCGTCGG TCCGGAGTG-3′, 5′-aaacCACTCCGGACCGACGCTGCA-3′, sg3: 5′-tcccCTGCAGCGTCGGTCCGGAGT-3′, 5′-aaacACTCCGGACCGACG CTGCAG-3′) before ligation into *BsmBI* sites of pFgH1tUT-GFP. 5-α Competent *E.coli* (high efficiency; New England Biolabs [NEB], C2987U) was used to prepare single colony preparations of plasmids. DNA was extracted and plasmids were subsequently subjected to Sanger sequencing by Monash Micromon Genomics.

Lentiviral transduction

Third-generation lentivirus was generated by transient transfection of HEK293T cells with lentiviral plasmids (dCas9-KRAB, pFgH1tUT), pMDL (packaging), RSV-REV (packaging) and VSVg (envelope) plasmids complexed into liposomes using Lipofectamine 2000 (Thermo Scientific) diluted in OptimMEM (Thermo Scientific). Second-generation lentivirus was generated via transient transfection of HEK293T cells with lentiviral pLVX, pPAX2 (packaging) and pMD2.G (envelope) plasmids complexed into liposomes using Lipofectamine 2000 (Thermo Scientific) diluted in OptimMEM (Thermo Scientific). Retrovirus was generated by transient transfection of HEK293T cells with pRetroX, gag-pol (packaging) and VSVg (envelope) plasmids complexed into liposomes using Lipofectamine 2000 (Thermo Scientific) diluted in OptimMEM (Thermo Scientific). Lentiviruses or retroviruses were harvested 48 h later, filtered through a 0.45 µm filter and used to infect iBMDMs target cell lines. Cells were subsequently enriched for lentiviral plasmid expression via antibiotic selection (i.e., 5 µg/ml blasticIdin, 2.5 µg/ml puromycin) or cell sorting using a BD Biosciences Influx™ sorter.

Expression of VPS4a in primary BMDMs

pRetroX-VPS4a and pRetroX-VPS4a-E228Q plasmids (Ruhl *et al*, 2018) were used to generate retroviruses in HEK293T cells as above, before retroviral transduction of day 2 bone marrow progenitor cells was performed as previously described (Cardona Gloria *et al*, 2018) to generate BMDMs expressing HA-tagged VPS4a^{WT} and VPS4a^{E228Q}.

Preparation of whole-cell lysates (WCLs)

For immunoblot assays, ~ 4×10^5 iBMDMs or 1×10^6 primary BMDMs were seeded in 12-well plates the day prior to cell stimulation. Following appropriate cell treatments, cells were lysed on ice with 150 µl of 1× Radioimmune precipitation assay (RIPA) buffer (20 mM Tris–HCl pH 7.4, 150 mM NaCl, 1 mM EDTA, 1% Triton X-100, 10% glycerol, 0.1% SDS and 0.5% deoxycholate, 5 mM NaF, 10 mM $NaPP_i$, 1 mM Na_3VO_4) supplemented with 1 mM phenylmethylsulfonyl fluoride (PMSF) and 1× cOmplete protease inhibitors (Roche Biochemicals). WCLs were clarified by centrifugation at 17,000 *g* for 1 min through Pierce centrifuge columns (Thermo Scientific) before 60 µl was diluted with 20 µl 4× reducing LDS-sample buffer (Thermo Scientific) supplemented with 5% β-mercaptoethanol (Sigma-Aldrich) (SB) and heated to 95°C for 10 min before SDS-polyacrylamide gel electrophoresis (SDS–PAGE) and immunoblot.

Immunoprecipitation (IP) and tandem ubiquitin binding entities (TUBE) assays

20×10^6 BMDMs or iBMDMs were suspended in 1 ml DMEM in a 1.5 ml Eppendorf tube with or without STING stimulation (e.g. 50 µg/ml DMXAA) as indicated with constant mixing. Cells were pelleted at 400 *g* for 5 min at 4°C. DMEM was removed by aspiration and cells were resuspended in 1 ml 1× NP-40 lysis buffer (40 mM Tris–HCl [pH 7.4], 2 mM EDTA, 2% Nonidet P-40 [IGEPAL], 20% Glycerol), supplemented with 1× cOmplete™ Protease inhibitor cocktail (Roche Biochemicals), 100 µM PMSF, 1 mM NaPPi, 500 µM NaF, 100 µM Na_3VO_4 and allowed to lyse on ice for 30 min. For TUBE experiments, the NP-40 lysis buffer was supplemented with DUB inhibitors 20 µM PR-619 (LifeSensors SI-9619) and 20 µM N-Ethylmaleimide (Sigma-Aldrich E3876). Samples were centrifuged at 17,000 *g* for 10 min at 4°C to clarify lysates. 90 µl of clarified WCL was mixed with 30 µl 4× SB, boiled at 95°C for 10 min. IP experiments were performed as previously described (De Nardo *et al*, 2018a). Here the primary antibody (anti-HA 3F-10, Roche # 11867431001 at 1 µg per IP sample; or anti-STING D1V5L Rabbit mAb (Rodent Preferred), Cell Signalling Technology #50494, used at 1:50) was added to the remaining lysate and incubated on a rotator for 1 h at 4°C. 50 µl of Protein G magnetic Dynabeads™ (Thermo Scientific) was then added to each sample and incubated for a further 1 h on a rotator at 4°C. Beads were washed 4× with 1 ml NP-40 lysis buffer using a magnetic holder. Protein was eluted by incubation with 35 µl 1× SB and boiled at 95°C for 10 min. For TUBE experiments, 50 µl of the slurry of TUBE agarose beads (LifeSensors UM-0401-1000) were added to each sample and incubated for 1 h on a rotator at 4°C. Beads were pulse centrifuged to sediment the beads and the supernatant was removed. Beads were washed 4× with 1 ml

 © 2023 The Authors

NP-40 lysis buffer. Protein was eluted by incubation with 35 µl 2× SB and boiled at 95°C for 10 min. Samples were centrifuged at 17,000 *g* for 1 min and underwent SDS–PAGE and immunoblot.

Immunoblotting

Between 20 and 35 µl of prepared WCL, IP or TUBE samples were run on NuPAGE™ 4–12% Bis-Tris Protein Gels (Thermo Scientific) with MES or MOPS running buffer (Thermo Scientific). Following activation of Immobilon-P polyvinyl difluoride (PVDF) membrane (Millipore Merk) in methanol, proteins were transferred to membranes using the Trans-Blot Turbo System (Bio-Rad). Membranes were then blocked using 5% skim milk powder in TBS + 0.1% Tween 20 (TBST) at room temperature (RT) for 1 h before incubation overnight in primary antibodies at 4°C. The following primary antibodies were purchased from Cell Signalling Technology (CST) and diluted as indicated; p-STING (Ser365) D8F4W Rabbit mAb #72971 1:1,000, p-STING (Ser366) D7C3S Rabbit mAb #19781 1:1,000, STING D2P2F Rabbit mAb #13647 1:1,000, STING D1V5L Rabbit mAb (Rodent Preferred) #50494 1:1–2000, p-TBK1/NAK (Ser172) D52C2 Rabbit mAb #5483 1:1–2,000, TBK1 #3013 1:1,000, p-IKKε (Ser172) D1B7 Rabbit mAb #8766 1:1,000, IKKε D61F9 Rabbit mAb #3416 1:1,000, p-IRF3 (Ser396) 4D4G Rabbit mAb #4947 1:500, IRF3 D83B9 Rabbit mAb #4302 1:1,000, p-NF-κB p65 (Ser536) 93H1 Rabbit mAb #3033 1:1,000, NF-κB p65 C22B4 Rabbit mAb #4764 1:1,000, Ubiquitin P4D1 Mouse mAb #3936 1:1,000, HRS D7T5N Rabbit mAb #15087 1:1,000, HA-Tag 6E2 Mouse mAb #2367 1:1,000, LC3B D11 Rabbit mAb #3868, 1:1,000. Additional antibodies include the following: β-actin Mouse mAb HRP-conjugated AC-15 Abcam ab49900, Hsp70 Mouse mAb 5A5 Abcam ab2787. Membranes were then washed 3× in TBST (10–20 min per wash) and incubated with appropriate HRP-conjugated secondary antibodies (Peroxidase-AffiniPure Goat Anti-Rabbit IgG: Jackson ImmunoResearch Labs #111-035-003, Goat anti-mouse Thermo Scientific #A16078) for 1 h at RT before membranes were again washed 3× in TBST. Chemiluminescence was detected by subjecting membranes to Immobilon Forte Western HRP substrate (Millipore Merk) before imaging using the ChemiDoc Touch or ChemiDoc XRS+ Imaging Systems (Bio-Rad). Images were acquired and converted to tagged image format file (TIFF) using Image Lab software (Bio-Rad). When required, antibodies were removed from membranes using a mild stripping buffer (50 mM glycine +0.4% SDS pH 2.2) before 3× washes in TBST and re-probing with required primary antibodies.

Reverse transcription quantitative polymerase chain reaction (RT–qPCR)

RNA was isolated from BMDMs or iBMDMs using the RNeasy Plus Mini Kit (QIAGEN). Complementary DNA (cDNA) was generated using SuperScript III Reverse Transcriptase (ThermoScientific). qPCR was performed with QuantiNova Probe PCR Master Mix SYBR Green (QIAGEN) using the Bio-Rad CFX384™ Real-Time System Thermal Cycler. Expression levels are displayed as $2^{-\Delta C_t}$, normalised to the housekeeping gene *Hprt*. Primer sequences used were as follows: *Hprt* Forward: 5′-tgaagtactcattatagtcaagggca-3′, *Hprt* Reverse: 5′-ctggtgaaaaggacctctcg-3′, *Ifnb1* Forward: 5′-ccagctccaa gaaaggacga-3′, *Ifnb1* Reverse: 5′-tggatggcaaaggcagtgta-3′, *Irf7* Forwa rd: 5′-aagctggagccatgggtatg-3′, *Irf7* Reverse: 5′-cgatgtcttcgtagaga ctgttgg-3′, *Isg15* Forward: 5′-tgtgagagcaagcagccaga-3′, *Isg15* Reverse: 5′-cccccagcatcttcaccttt-3′, *Il6* Forward: 5′-ccagaaaccgctatgaagttcc-3′, *Il6* Reverse: 5′-cggacttgtgaagtagggaagg-3′.

Enzyme-Linked immunosorbent assay (ELISA)

Cell supernatant was assayed for murine TNF or IL-6 using eBioscence kits (Thermo Scientific) according to the manufacturer's protocol. Murine IFNβ was measured using a custom-made protocol as previously reported (Roberts *et al*, 2007). The monoclonal rat anti-mouse IFNβ (USBiological Life Sciences; 138027) was used as a coating antibody, while the polyclonal rabbit anti-mouse IFNβ (PBL Assay Science; 32400-1) was used for detection. Recombinant mouse IFNβ (carrier-free) (PBL Assay Science; 12401-1) was used to generate a standard curve, peroxidase-conjugated AffiniPure F(ab′)$_2$ fragment donkey anti-rabbit IgG (H + L) (Jackson Immuno Research; 711-036-152) was used for colorimetric detection, and PBS with 1% bovine serum albumin (BSA) was used as the assay diluent.

Immunofluorescence

2.5–5 × 10^4 iBMDMs or 1 × 10^5 primary BMDMs were seeded in 8-well µ-slide ibiTreat chamber slides (iBidi). The next day, following any cell treatments, cells were washed with PBS and fixed with 4% paraformaldehyde (PFA) in PBS at RT for 30 min. Cells were then washed twice with PBS, permeabilised with 0.15% Triton X-100 in PBS for 20 min and then incubated with blocking buffer (PBS + 10% FBS) for 1 h at RT. Primary antibodies (p-STING (Ser365) CST D1C4T Rabbit mAb #62912 1:400, HRS CST D7T5N Rabbit mAb #15087 1:400, Ubiquitin FK2 Mouse mAb Sigma-Aldrich #04-263 1:1,500) were diluted in blocking buffer and samples were incubated at 4°C overnight. The following day, samples were washed 3 times with blocking buffer before incubation with Alexa-conjugated secondary antibodies (Goat anti-rabbit IgG AlexaFlour™ 647: Thermo Scientific #A21245, Goat anti-mouse IgG AlexaFluor™ plus 555: ThermoScientific #A32727) diluted 1:1,000 in blocking buffer. Samples were subsequently washed 3 times with blocking buffer before nuclear staining with 300 nM DAPI (in PBS) for 10 min at RT and then a final wash using PBS. Z-stack images (with a 0.5 µm Z step) were acquired using the Zeiss confocal light scanning microscope (LSM) 980, equipped with Airyscan 2 detector with ZEN software. A 63×/1.40 NA oil objective with Immersol 518F immersion oil (Zeiss) was used. Images were handled in ImageJ to generate single colour grayscale and composite images. The intensity plots were generated using the plot profile tool.

Imaging analysis of p-STING puncta

A FIJI macro and CellProfiler pipeline were used to quantify the number of phosphorylated (p)-STING puncta per cell and analyse their intensity. Firstly, the FIJI macro was used to batch-process all image files: generation of maximum intensity projections (MIP) of Z-stacks; splitting (3) channels; and conversion of images into a binary mask, removing objects touching edges. This mask was subsequently used for cell segmentation in CellProfiler. Secondly, the resulting images files were used as input for the CellProfiler pipeline to detect the number of p-STING puncta per cell. The binary mask

was used to segment cell objects. The p-STING puncta were detected and related as a component (child) of the cell object (parent). A reference overlay outline of the puncta and cell objects was created to calculate different measurements (cell and related cell-puncta size, area occupied and intensity) and exported in a spreadsheet before data were plotted using GraphPad Prism.

Live cell imaging

$2.5–5 \times 10^4$ iBMDMs were seeded in 8-well μ-slide ibiTreat chamber slides (iBidi). The following day cells were imaged live using the 3i marianas spinning disk confocal microscope with CSU-W1T2 Yokogawa spinning disk confocal (50 μm disk) coupled to inverted Zeiss Axio observer 7 stand. iBMDMs were imaged before or after treatment with 50 μg/ml DMXAA. Z stack images were acquired with a 0.5 μm z-step over time. Details for image acquisition can be found in figure legends. Imaris software was used to export 3D movies for visualisation as mp4 files. Single images or image sequences were exported from ImageJ either as maximum intensity projections (MIPs) or a single z-slice.

Live cell imaging analysis

To measure the increase in Golgi accumulation of eGFP-STING, cells treated with 50 μg/ml DMXAA were imaged as a volumetric time-series ~ 2 min post addition. Two ROIs were drawn, one encompassing the perinuclear Golgi and the other including the more peripheral endoplasmic reticulum, identified from the last frames. The mean intensity above background, for both ROIs was measured from maximum intensity projections of the volumes and the Golgi/ER intensity was plotted over time. For obtaining intensity loss at Golgi; Golgi boundary was obtained by watershed-based segmentation and intensity was measured. The individual cells were normalised by maximum value (usually the first time-point) to describe the rate of STING loss independent of initial values and to ascertain variability in the rates. Linear parts of the curve were fit using a linear function and presented as a guide to the eye. To obtain endosomal numbers, the data were first photo-bleach corrected using histogram normalisation (Miura, 2020) to avoid undercounting owing to bleaching, and detected and tracked as previously described (York *et al*, 2021), and counted for individual cells across entire time-lapse movies.

Phosphoproteomic screen

Primary BMDMs were seeded at a density of 10×10^6 in 10 cm petri dishes the day before the experiment. BMDMs were then left untreated or treated with 50 μg/ml DMXAA or the corresponding dilution of DMSO for 5, 10 or 30 min. All treatments were performed in quadruplicate. BMDMs were then washed 3× with TBS and lysed with 1 ml of lysis buffer (4% sodium deoxycholate [SDC], 100 mM HEPES) on ice, before samples were snap-frozen on dry ice. Samples were processed by the Monash Proteomics and Metabolomics Facility (MPMF) similarly as previously described (Huang *et al*, 2020). Briefly, protein lysates were reduced with 10 mM TCEP (ThermoFisher), alkylated with 40 mM CAA (Sigma) and digested with trypsin (Promega). The majority of each sample was subjected to phosphopeptide enrichment, whilst a small aliquot was removed for the analysis of the underlying proteome. Both sample types were analysed by LC–MS/MS on a QExactive HF mass spectrometer (ThermoFisher Scientific) coupled online to a RSLC nano HPLC (Ultimate 3000, UHPLC ThermoFisher Scientific). Samples were loaded onto a 100 μm, 2 cm nanoviper Pepmap100 trap column, eluted and separated on a RSLC nano column 75 μm × 50 cm, Pepmap100 C18 analytical column (ThermoFisher Scientific). The tryptic peptides or phosphopeptides were separated by increasing concentrations of 80% ACN/0.1% formic acid at a flow of 250 nl/min for 158 min. Data-independent acquisition (DIA) mass spectrometry was used to analyse the total proteome. The instrument was set to switch between a full MS scan and 43 subsequent DIA scans. Survey full scan MS spectra (m/z 375–1,575) were acquired with 60,000 resolution (at m/z 200) after accumulating ions to an AGC (Automatic Gain Control) target of 3×10^6 with a maximum injection time of 54 ms. 43 DIA scans were acquired with 15,000 resolution (at m/z 200) covering the m/z range between 375 and 975 and using an isolation window of 14 m/z. The AGC target was set to 2×10^5 with a maximum injection time of 22 ms and the normalised collision energy to 27. Spectronaut v14 (Biognosys) was used to analyse all DIA data. Data-dependent acquisition (DDA) mass spectrometry was used to analyse the phosphoproteome. The instrument was set to toggle between a full MS scan and subsequent MS/MS scans. Survey full scan MS spectra (m/z 375–1,575) were acquired with 60,000 resolution (at m/z 200) after accumulating ions to an AGC \ target of 3×10^6 with a maximum injection time of 54 ms. Dynamic exclusion was set to 30 s. The 30 most intense multiply charged ions ($z \geq 2$) were sequentially isolated and fragmented in the collision cell by higher-energy collisional dissociation (HCD) with a maximum injection time of 54 ms, 30,000 resolution and an AGC target of 1×10^5. MaxQuant (version 1.6.5.0; Cox & Mann, 2008) was used to interrogate all DDA files against a human protein sequence database. Cysteine carbamidomethylation was specified as a fixed modification, and methionine oxidation, N-terminal acetylation and phosphorylation at serine, threonine or tyrosine were set as variable modifications. Only proteins and peptides falling below a false discovery rate (FDR) of 1% were considered for subsequent analyses.

Analysis of phosphoproteomics data

Phosphoproteomics data were further analysed using R language (https://www.R-project.org/). Normalised intensity values were supplied by the Monash Proteomics and Metabolomics Facility (MPMF). Samples were visualised via uniform manifold approximation and projection (UMAP) using the *umap* library. One pair of replicates (DMXAA- and DMSO-treated) at the 30 min time point exhibited abnormalities consistent with a suspected technical error and were excluded from subsequent analysis. Unpaired *t*-tests were performed using the *stats* library. Differentially phosphorylated residues were selected with a threshold of $P \leq 0.01$ and Log2FC ≥ 1. Volcano plots and intensity plots were generated in GraphPad Prism, while heatmaps were produced using the *ggplot2* library. Gene ontology (GO) and Reactome pathway analysis was performed using the STRING online platform (https://string-db.org).

Statistical analysis

Unpaired two-tailed Student's *t*-test, one-way or two-way ANOVA was performed using Prism 7.0 GraphPad Software as indicated in

© 2023 The Authors

figure legends. No calculations were performed to estimate sample size. Samples were not blinded during experimentation.

All reagents are available upon request from the corresponding authors.

Data availability

The mass spectrometry proteomics data have been deposited to the ProteomeXchange Consortium via the PRoteomics IDEntifications (PRIDE) (Perez-Riverol *et al*, 2022) partner repository with the dataset identifier PXD037108.

Expanded View for this article is available online.

Acknowledgements
We thank Professor Petr Broz (University of Lausanne) for kindly providing the pRetroX-VPS4a and pRetroX-VPS4a-E228Q plasmids. We acknowledge Associate Professor Joseph Rosenbluh (Monash University) for the dCas9-KRAB-expressing plasmid. pLVX-EF1a-EGFP-TA-Golgin84-IRES-Puromycin was a gift from David Andrews (Addgene plasmid # 133028; http://n2t.net/addgene:133028; RRID: Addgene_133028). We also acknowledge Monash Micro Imaging (MMI), the Monash Proteomics and Metabolomics Facility (MPMF), Flowcore, Monash Micromon and Monash Animal Research Platform (MARP) facilities for their support and contribution to this work. This study used BPA-enabled (Bioplatforms Australia)/NCRIS-enabled (National Collaborative Research Infrastructure Strategy) infrastructure located at the Monash Proteomics and Metabolomics Facility. K.R.B. is supported by a Monash Silver Jubilee Postgraduate Research scholarship and a Monash Graduate Excellence Scholarship. R.V., A.S. and Z.M. are supported by Australian Government Research Training Program Scholarships. D.D.N. is supported in part by a Monash University FMNHS Senior Postdoctoral Fellowship. This work was supported by an Australian National Health and Medical Research Council (NHMRC) Program Grant (1113577) to B.T.K. and an Australian Research Council (ARC) Discovery Project Grant (DP210103122) to M.O.K. Open access publishing facilitated by Monash University, as part of the Wiley - Monash University agreement via the Council of Australian University Librarians.

Author contributions
Katherine R Balka: Conceptualization; resources; data curation; formal analysis; validation; investigation; methodology; writing – original draft; project administration; writing – review and editing. **Rajan Venkatraman:** Data curation; investigation; writing – review and editing. **Tahnee L Saunders:** Formal analysis; methodology; writing – review and editing. **Angus Shoppee:** Formal analysis; writing – review and editing. **Ee Shan Pang:** Investigation; writing – review and editing. **Zoe Magill:** Investigation; writing – review and editing. **Jihane Homman-Ludiye:** Formal analysis; writing – review and editing. **Cheng Huang:** Data curation; formal analysis; investigation; methodology; writing – review and editing. **Rachael M Lane:** Resources; writing – review and editing. **Harrison M York:** Data curation; formal analysis; writing – review and editing. **Peck Tan:** Investigation; writing – review and editing. **Ralf B Schittenhelm:** Formal analysis; supervision; methodology; writing – review and editing. **Senthil Arumugam:** Formal analysis; supervision; writing – review and editing. **Benjamin T Kile:** Resources; supervision; funding acquisition. **Meredith O'Keeffe:** Conceptualization; formal analysis; supervision; funding acquisition; writing – review and editing. **Dominic De Nardo:** Conceptualization; resources; data curation; formal analysis; supervision; validation; investigation; methodology; writing – original draft; project administration; writing – review and editing.

Disclosure and competing interests statement
The authors declare that they have no conflict of interest.

References

Babst M, Wendland B, Estepa EJ, Emr SD (1998) The Vps4p AAA ATPase regulates membrane association of a Vps protein complex required for normal endosome function. *EMBO J* 17: 2982–2993

Baker PJ, Masters SL (2018) Generation of genetic knockouts in myeloid cell lines using a lentiviral CRISPR/Cas9 system. *Methods Mol Biol* 1714: 41–55

Baker PJ, De Nardo D, Moghaddas F, Tran LS, Bachem A, Nguyen T, Hayman T, Tye H, Vince JE, Bedoui S *et al* (2017) Posttranslational modification as a critical determinant of cytoplasmic innate immune recognition. *Physiol Rev* 97: 1165–1209

Balka KR, De Nardo D (2021) Molecular and spatial mechanisms governing STING signalling. *FEBS J* 288: 5504–5529

Balka KR, Louis C, Saunders TL, Smith AM, Calleja DJ, D'Silva DB, Moghaddas F, Tailler M, Lawlor KE, Zhan Y *et al* (2020) TBK1 and IKKepsilon act redundantly to mediate STING-induced NF-kappaB responses in myeloid cells. *Cell Rep* 31: 107492

Barbosa MD, Nguyen QA, Tchernev VT, Ashley JA, Detter JC, Blaydes SM, Brandt SJ, Chotai D, Hodgman C, Solari RC *et al* (1996) Identification of the homologous beige and Chediak-higashi syndrome genes. *Nature* 382: 262–265

Burkhardt JK, Wiebel FA, Hester S, Argon Y (1993) The giant organelles in beige and Chediak-higashi fibroblasts are derived from late endosomes and mature lysosomes. *J Exp Med* 178: 1845–1856

Cardona Gloria Y, Latz E, De Nardo D (2018) Generation of innate immune reporter cells using retroviral transduction. *Methods Mol Biol* 1714: 97–117

Chu TT, Tu X, Yang K, Wu J, Repa JJ, Yan N (2021) Tonic prime-boost of STING signalling mediates Niemann-pick disease type C. *Nature* 596: 570–575

Cox J, Mann M (2008) MaxQuant enables high peptide identification rates, individualized p.p.b.-range mass accuracies and proteome-wide protein quantification. *Nat Biotechnol* 26: 1367–1372

De Nardo D, Balka KR, Cardona Gloria Y, Rao VR, Latz E, Masters SL (2018a) Interleukin-1 receptor-associated kinase 4 (IRAK4) plays a dual role in myddosome formation and toll-like receptor signaling. *J Biol Chem* 293: 15195–15207

De Nardo D, Kalvakolanu DV, Latz E (2018b) Immortalization of murine bone marrow-derived macrophages. *Methods Mol Biol* 1784: 35–49

Denais CM, Gilbert RM, Isermann P, McGregor AL, te Lindert M, Weigelin B, Davidson PM, Friedl P, Wolf K, Lammerding J (2016) Nuclear envelope rupture and repair during cancer cell migration. *Science* 352: 353–358

Deng Z, Chong Z, Law CS, Mukai K, Ho FO, Martinu T, Backes BJ, Eckalbar WL, Taguchi T, Shum AK (2020) A defect in COPI-mediated transport of STING causes immune dysregulation in COPA syndrome. *J Exp Med* 217: e20201045

Dobbs N, Burnaevskiy N, Chen D, Gonugunta VK, Alto NM, Yan N (2015) STING activation by translocation from the ER is associated with infection and autoinflammatory disease. *Cell Host Microbe* 18: 157–168

Drusenheimer N, Migdal B, Jackel S, Tveriakhina L, Scheider K, Schulz K, Groper J, Kohrer K, Klein T (2015) The mammalian orthologs of drosophila Lgd, CC2D1A and CC2D1B, function in the endocytic pathway, but their individual loss of function does not affect notch Signalling. *PLoS Genet* 11: e1005749

© 2023 The Authors

Ergun SL, Fernandez D, Weiss TM, Li L (2019) STING polymer structure reveals mechanisms for activation, hyperactivation, and inhibition. *Cell* 178: 290–301.e10

Gentili M, Liu B, Papanastasiou M, Dele-Oni D, Schwartz MA, Carlson RJ, Al'Khafaji AM, Krug K, Brown A, Doench JG *et al* (2023) ESCRT-dependent STING degradation inhibits steady-state and cGAMP-induced signalling. *Nat Commun* 14: 611

Gonugunta VK, Sakai T, Pokatayev V, Yang K, Wu J, Dobbs N, Yan N (2017) Trafficking-mediated STING degradation requires sorting to acidified Endolysosomes and can Be targeted to enhance anti-tumor response. *Cell Rep* 21: 3234–3242

Gui X, Yang H, Li T, Tan X, Shi P, Li M, Du F, Chen ZJ (2019) Autophagy induction via STING trafficking is a primordial function of the cGAS pathway. *Nature* 567: 262–266

Hancock-Cerutti W, Wu Z, Xu P, Yadavalli N, Leonzino M, Tharkeshwar AK, Ferguson SM, Shadel GS, De Camilli P (2022) ER-lysosome lipid transfer protein VPS13C/PARK23 prevents aberrant mtDNA-dependent STING signaling. *J Cell Biol* 221: e202106046

Hjerpe R, Aillet F, Lopitz-Otsoa F, Lang V, England P, Rodriguez MS (2009) Efficient protection and isolation of ubiquitylated proteins using tandem ubiquitin-binding entities. *EMBO Rep* 10: 1250–1258

Huang C, Foster SR, Shah AD, Kleifeld O, Canals M, Schittenhelm RB, Stone MJ (2020) Phosphoproteomic characterization of the signaling network resulting from activation of the chemokine receptor CCR2. *J Biol Chem* 295: 6518–6531

Ishikawa H, Ma Z, Barber GN (2009) STING regulates intracellular DNA-mediated, type I interferon-dependent innate immunity. *Nature* 461: 788–792

Jin L, Hill KK, Filak H, Mogan J, Knowles H, Zhang B, Perraud AL, Cambier JC, Lenz LL (2011) MPYS is required for IFN response factor 3 activation and type I IFN production in the response of cultured phagocytes to bacterial second messengers cyclic-di-AMP and cyclic-di-GMP. *J Immunol* 187: 2595–2601

Kato T, Yamamoto M, Honda Y, Orimo T, Sasaki I, Murakami K, Hemmi H, Fukuda-Ohta Y, Isono K, Takayama S *et al* (2021) Augmentation of stimulator of interferon genes-induced type I interferon production in COPA syndrome. *Arthritis Rheumatol* 73: 2105–2115

Komada M, Soriano P (1999) Hrs, a FYVE finger protein localized to early endosomes, is implicated in vesicular traffic and required for ventral folding morphogenesis. *Genes Dev* 13: 1475–1485

Konno H, Konno K, Barber GN (2013) Cyclic dinucleotides trigger ULK1 (ATG1) phosphorylation of STING to prevent sustained innate immune signaling. *Cell* 155: 688–698

Kuchitsu Y, Mukai K, Uematsu R, Takaada Y, Shinojima A, Shindo R, Shoji T, Hamano S, Ogawa E, Sato R *et al* (2023) STING signalling is terminated through ESCRT-dependent microautophagy of vesicles originating from recycling endosomes. *Nat Cell Biol* 25: 453–466

Lepelley A, Martin-Niclos MJ, Le Bihan M, Marsh JA, Uggenti C, Rice GI, Bondet V, Duffy D, Hertzog J, Rehwinkel J *et al* (2020) Mutations in COPA lead to abnormal trafficking of STING to the Golgi and interferon signaling. *J Exp Med* 217: e20200600

Liu S, Cai X, Wu J, Cong Q, Chen X, Li T, Du F, Ren J, Wu YT, Grishin NV *et al* (2015) Phosphorylation of innate immune adaptor proteins MAVS, STING, and TRIF induces IRF3 activation. *Science* 347: aaa2630

Liu D, Wu H, Wang C, Li Y, Tian H, Siraj S, Sehgal SA, Wang X, Wang J, Shang Y *et al* (2019) STING directly activates autophagy to tune the innate immune response. *Cell Death Differ* 26: 1735–1749

Liu Y, Xu P, Rivara S, Liu C, Ricci J, Ren X, Hurley JH, Ablasser A (2022) Clathrin-associated AP-1 controls termination of STING signalling. *Nature* 610: 761–767

Luo WW, Li S, Li C, Lian H, Yang Q, Zhong B, Shu HB (2016) iRhom2 is essential for innate immunity to DNA viruses by mediating trafficking and stability of the adaptor STING. *Nat Immunol* 17: 1057–1066

McCauley ME, O'Rourke JG, Yanez A, Markman JL, Ho R, Wang X, Chen S, Lall D, Jin M, Muhammad A *et al* (2020) C9orf72 in myeloid cells suppresses STING-induced inflammation. *Nature* 585: 96–101

Miura K (2020) Bleach correction ImageJ plugin for compensating the photobleaching of time-lapse sequences. *F1000Res* 9: 1494

Mochida GH, Ganesh VS, de Michelena MI, Dias H, Atabay KD, Kathrein KL, Huang HT, Hill RS, Felie JM, Rakiec D *et al* (2012) CHMP1A encodes an essential regulator of BMI1-INK4A in cerebellar development. *Nat Genet* 44: 1260–1264

Mukai K, Ogawa E, Uematsu R, Kuchitsu Y, Kiku F, Uemura T, Waguri S, Suzuki T, Dohmae N, Arai H *et al* (2021) Homeostatic regulation of STING by retrograde membrane traffic to the ER. *Nat Commun* 12: 61

Ni G, Konno H, Barber GN (2017) Ubiquitination of STING at lysine 224 controls IRF3 activation. *Sci Immunol* 2: eaah7119

Ogawa E, Mukai K, Saito K, Arai H, Taguchi T (2018) The binding of TBK1 to STING requires exocytic membrane traffic from the ER. *Biochem Biophys Res Commun* 503: 138–145

Olmos Y, Hodgson L, Mantell J, Verkade P, Carlton JG (2015) ESCRT-III controls nuclear envelope reformation. *Nature* 522: 236–239

Perez-Riverol Y, Bai J, Bandla C, Garcia-Seisdedos D, Hewapathirana S, Kamatchinathan S, Kundu DJ, Prakash A, Frericks-Zipper A, Eisenacher M *et al* (2022) The PRIDE database resources in 2022: a hub for mass spectrometry-based proteomics evidences. *Nucleic Acids Res* 50: D543–D552

Prabakaran T, Bodda C, Krapp C, Zhang BC, Christensen MH, Sun C, Reinert L, Cai Y, Jensen SB, Skouboe MK *et al* (2018) Attenuation of cGAS-STING signaling is mediated by a p62/SQSTM1-dependent autophagy pathway activated by TBK1. *EMBO J* 37: e97858

Raab M, Gentili M, de Belly H, Thiam HR, Vargas P, Jimenez AJ, Lautenschlaeger F, Voituriez R, Lennon-Dumenil AM, Manel N *et al* (2016) ESCRT III repairs nuclear envelope ruptures during cell migration to limit DNA damage and cell death. *Science* 352: 359–362

Ran Y, Xiong MG, Xu ZS, Luo WW, Wang SY, Wang YY (2019) YIPF5 is essential for innate immunity to DNA virus and facilitates COPII-dependent STING trafficking. *J Immunol* 203: 1560–1570

Roberts ZJ, Goutagny N, Perera PY, Kato H, Kumar H, Kawai T, Akira S, Savan R, van Echo D, Fitzgerald KA *et al* (2007) The chemotherapeutic agent DMXAA potently and specifically activates the TBK1-IRF-3 signaling axis. *J Exp Med* 204: 1559–1569

Rodger C, Flex E, Allison RJ, Sanchis-Juan A, Hasenahuer MA, Cecchetti S, French CE, Edgar JR, Carpentieri G, Ciolfi A *et al* (2020) De novo VPS4A mutations cause multisystem disease with abnormal neurodevelopment. *Am J Hum Genet* 107: 1129–1148

Ruhl S, Shkarina K, Demarco B, Heilig R, Santos JC, Broz P (2018) ESCRT-dependent membrane repair negatively regulates pyroptosis downstream of GSDMD activation. *Science* 362: 956–960

Sepulveda FE, Burgess A, Heiligenstein X, Goudin N, Menager MM, Romao M, Cote M, Mahlaoui N, Fischer A, Raposo G *et al* (2015) LYST controls the biogenesis of the endosomal compartment required for secretory lysosome function. *Traffic* 16: 191–203

Shiels A, Bennett TM, Knopf HL, Yamada K, Yoshiura K, Niikawa N, Shim S, Hanson PI (2007) CHMP4B, a novel gene for autosomal dominant cataracts linked to chromosome 20q. *Am J Hum Genet* 81: 596–606

Srikanth S, Woo JS, Wu B, El-Sherbiny YM, Leung J, Chupradit K, Rice L, Seo GJ, Calmettes G, Ramakrishna C *et al* (2019) The Ca(2+) sensor STIM1

 © 2023 The Authors

regulates the type I interferon response by retaining the signaling adaptor STING at the endoplasmic reticulum. *Nat Immunol* 20: 152–162

Steiner A, Hrovat-Schaale K, Prigione I, Yu CH, Laohamonthonkul P, Harapas CR, Low RRJ, De Nardo D, Dagley LF, Mlodzianoski MJ *et al* (2022) Deficiency in coatomer complex I causes aberrant activation of STING signalling. *Nat Commun* 13: 2321

Sun MS, Zhang J, Jiang LQ, Pan YX, Tan JY, Yu F, Guo L, Yin L, Shen C, Shu HB *et al* (2018) TMED2 potentiates cellular IFN responses to DNA viruses by reinforcing MITA dimerization and facilitating its trafficking. *Cell Rep* 25: 3086–3098.e3

Tanaka Y, Chen ZJ (2012) STING specifies IRF3 phosphorylation by TBK1 in the cytosolic DNA signaling pathway. *Sci Signal* 5: ra20

Tu X, Chu TT, Jeltema D, Abbott K, Yang K, Xing C, Han J, Dobbs N, Yan N (2022) Interruption of post-Golgi STING trafficking activates tonic interferon signaling. *Nat Commun* 13: 6977

Vietri M, Schink KO, Campsteijn C, Wegner CS, Schultz SW, Christ L, Thoresen SB, Brech A, Raiborg C, Stenmark H (2015) Spastin and ESCRT-III coordinate mitotic spindle disassembly and nuclear envelope sealing. *Nature* 522: 231–235

Vietri M, Radulovic M, Stenmark H (2020) The many functions of ESCRTs. *Nat Rev Mol Cell Biol* 21: 25–42

Watson JA, Bhattacharyya BJ, Vaden JH, Wilson JA, Icyuz M, Howard AD, Phillips E, DeSilva TM, Siegal GP, Bean AJ *et al* (2015) Motor and sensory deficits in the teetering mice result from mutation of the ESCRT component HGS. *PLoS Genet* 11: e1005290

Westphal A, Cheng W, Yu J, Grassl G, Krautkramer M, Holst O, Foger N, Lee KH (2017) Lysosomal trafficking regulator Lyst links membrane trafficking to toll-like receptor-mediated inflammatory responses. *J Exp Med* 214: 227–244

Williams RL, Urbe S (2007) The emerging shape of the ESCRT machinery. *Nat Rev Mol Cell Biol* 8: 355–368

Windhorst DB, Zelickson AS, Good RA (1966) Chediak-higashi syndrome: hereditary gigantism of cytoplasmic organelles. *Science* 151: 81–83

Wu J, Dobbs N, Yang K, Yan N (2020) Interferon-independent activities of mammalian STING mediate antiviral response and tumor immune evasion. *Immunity* 53: 115–126.e5

Yamashiro LH, Wilson SC, Morrison HM, Karalis V, Chung JJ, Chen KJ, Bateup HS, Szpara ML, Lee AY, Cox JS *et al* (2020) Interferon-independent STING signaling promotes resistance to HSV-1 in vivo. *Nat Commun* 11: 3382

York HM, Patil A, Moorthi UK, Kaur A, Bhowmik A, Hyde GJ, Gandhi H, Fulcher A, Gaus K, Arumugam S (2021) Rapid whole cell imaging reveals a calcium-APPL1-dynein nexus that regulates cohort trafficking of stimulated EGF receptors. *Commun Biol* 4: 224

Yum S, Li M, Fang Y, Chen ZJ (2021) TBK1 recruitment to STING activates both IRF3 and NF-kappaB that mediate immune defense against tumors and viral infections. *Proc Natl Acad Sci USA* 118: e2100225118

Zhang BC, Nandakumar R, Reinert LS, Huang J, Laustsen A, Gao ZL, Sun CL, Jensen SB, Troldborg A, Assil S *et al* (2020) STEEP mediates STING ER exit and activation of signaling. *Nat Immunol* 21: 868–879

Zhao B, Du F, Xu P, Shu C, Sankaran B, Bell SL, Liu M, Lei Y, Gao X, Fu X *et al* (2019) A conserved PLPLRT/SD motif of STING mediates the recruitment and activation of TBK1. *Nature* 569: 718–722

License: This is an open access article under the terms of the Creative Commons Attribution License, which permits use, distribution and reproduction in any medium, provided the original work is properly cited.

Expanded View Figures

Figure EV1. Phosphorylation of STING and STING signalling molecules.

A Schematic of the C-terminal tail (CTT) of STING where multiple phosphorylation sites were identified.

B–L The measured intensity values from the phosphoproteomics screen for phosphorylated peptides corresponding to STING (B–E), IKKβ (F), CYLD (G–H), OPTN (I), iRHOM2 (J–L), were plotted for UT, DMSO and DMXAA-treated conditions. The phosphorylated residue is indicated within the graph title. Data shown as mean ± SEM for four replicates. Statistical analysis was performed using unpaired Student's *t*-test. *$P < 0.05$, **$P < 0.01$, ***$P < 0.001$, ****$P < 0.0001$.

© 2023 The Authors

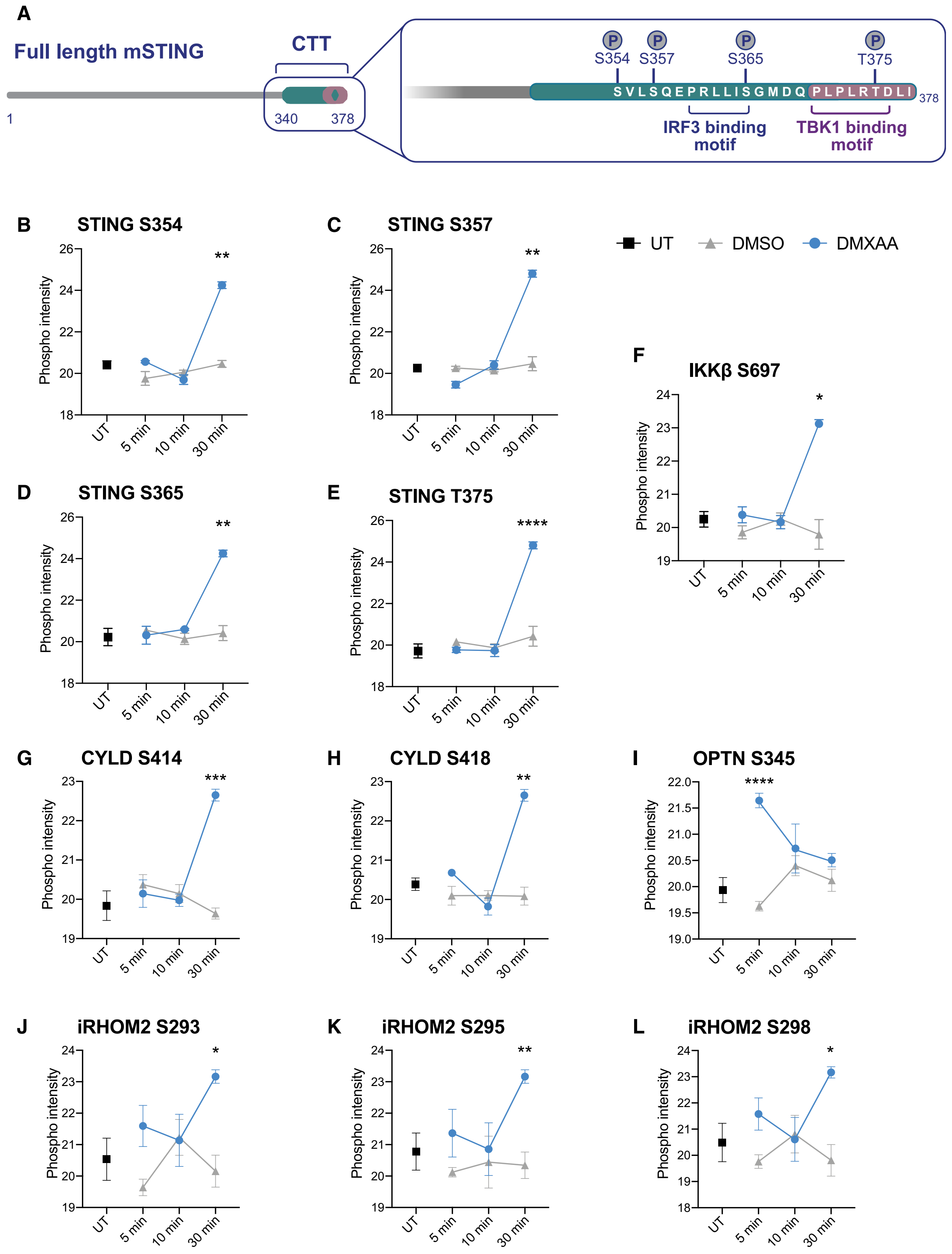
A
Full length mSTING
CTT
1
340
378
S354
S357
S365
T375
SVLSQEPRLLISGMDQ
PLPLRTDLI
378
IRF3 binding motif
TBK1 binding motif
B STING S354
C STING S357
D STING S365
E STING T375
F IKKβ S697
G CYLD S414
H CYLD S418
I OPTN S345
J iRHOM2 S293
K iRHOM2 S295
L iRHOM2 S298
UT
DMSO
DMXAA
Phospho intensity
5 min
10 min
30 min

Figure EV1.

Figure EV2. Phosphorylation of trafficking proteins in response to STING activation.

A–F The measured intensity values from the phosphoproteomics screen for phosphorylated peptides corresponding to Rab7a (A), CHMP4B (B), LYST (C), TOM1 (D), ATP6V1G3 (E) and CD-MPR (F) were plotted for UT, DMSO and DMXAA-treated conditions. The phosphorylated residue is indicated within the graph title. Data shown as mean ± SEM for four replicates. Statistical analysis was performed using unpaired Student's *t*-test. *$P < 0.05$, **$P < 0.01$, ****$P < 0.0001$.

G Gene ontology (GO) analysis was performed for all proteins found with phosphorylation changes across all three timepoints. Proteins characterised within the organelles STING traffics through were identified using "Cellular components". Proteins localised to the Golgi, endosomes, lysosomes and clathrin-coated vesicles are depicted on a schematic of STING trafficking.

 © 2023 The Authors

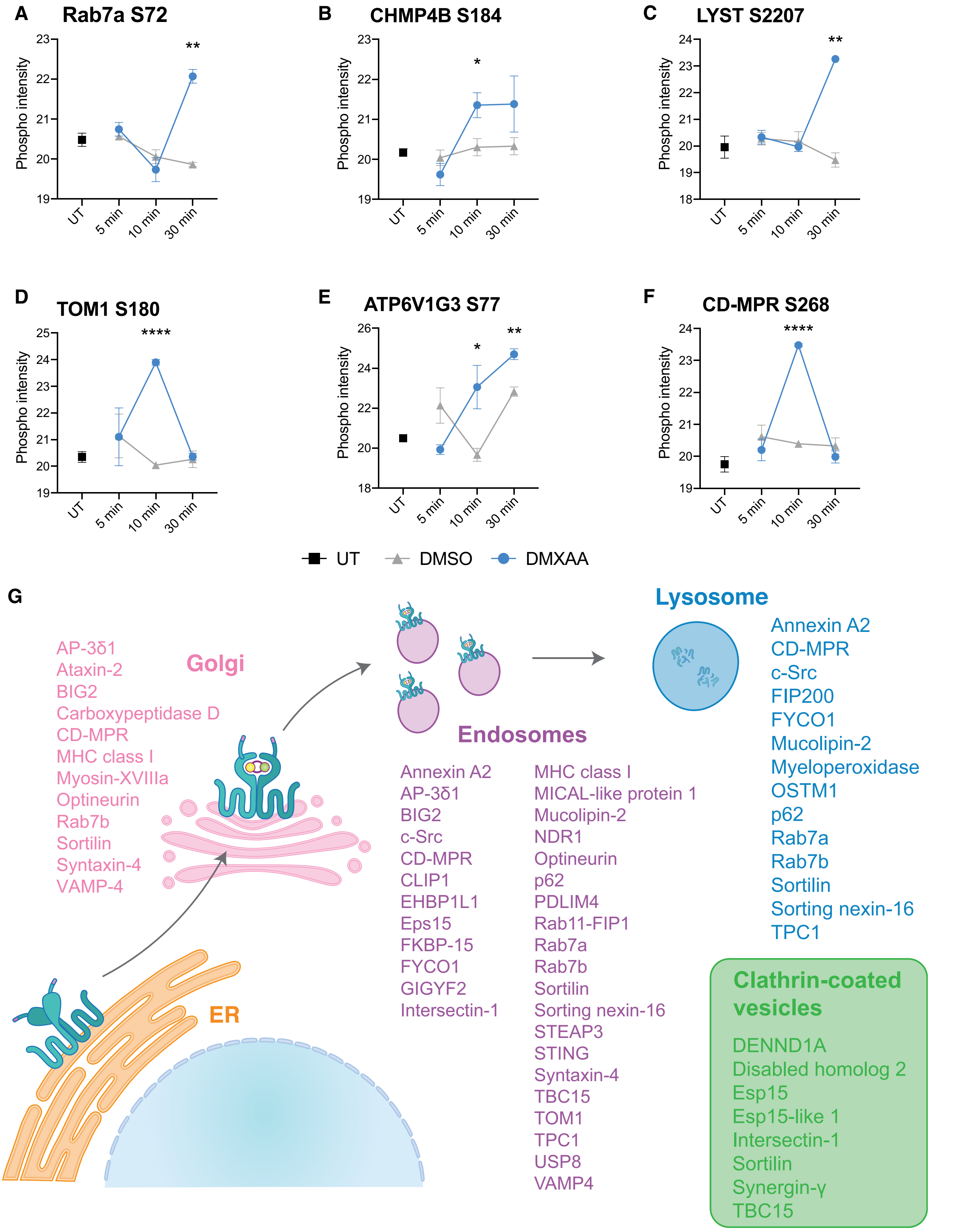

A
Rab7a S72
B
CHMP4B S184
C
LYST S2207
D
TOM1 S180
E
ATP6V1G3 S77
F
CD-MPR S268
Phospho intensity
UT
5 min
10 min
30 min
UT DMSO DMXAA
G
Golgi
AP-3δ1
Ataxin-2
BIG2
Carboxypeptidase D
CD-MPR
MHC class I
Myosin-XVIIIa
Optineurin
Rab7b
Sortilin
Syntaxin-4
VAMP-4
ER
Endosomes
Annexin A2
AP-3δ1
BIG2
c-Src
CD-MPR
CLIP1
EHBP1L1
Eps15
FKBP-15
FYCO1
GIGYF2
Intersectin-1
MHC class I
MICAL-like protein 1
Mucolipin-2
NDR1
Optineurin
p62
PDLIM4
Rab11-FIP1
Rab7a
Rab7b
Sortilin
Sorting nexin-16
STEAP3
STING
Syntaxin-4
TBC15
TOM1
TPC1
USP8
VAMP4
Lysosome
Annexin A2
CD-MPR
c-Src
FIP200
FYCO1
Mucolipin-2
Myeloperoxidase
OSTM1
p62
Rab7a
Rab7b
Sortilin
Sorting nexin-16
TPC1
Clathrin-coated vesicles
DENND1A
Disabled homolog 2
Esp15
Esp15-like 1
Intersectin-1
Sortilin
Synergin-γ
TBC15

Figure EV2.

Figure EV3. STING rapidly traffics from the ER to the Golgi.

A *Sting*$^{-/-}$ iBMDMs alone or *Sting*$^{-/-}$ iBMDMs reconstituted with either eGFP- or mRuby3-tagged STING were treated with 50 μg/ml BMDMs for 2 h. Cells were lysed for immunoblot with the indicated antibodies. Data shown are representative of three independent experiments.

B *Sting*$^{-/-}$ iBMDMs expressing eGFP-STING were imaged using spinning disk microscopy in resting conditions. Data are shown as both a single Z slice and maximum intensity projection (MIP) of Z stack images. Data shown are representative of three independent experiments. Scale bar = 5 μm.

C *Sting*$^{-/-}$ iBMDMs expressing eGFP-STING were imaged live on the spinning disk microscope. Images display a time series as indicated, showing STING translocation from ER to Golgi after 50 μg/ml DMXAA treatment. Data are shown as both a single Z slice and MIP of Z stack images. Data shown are representative of three independent experiments. Scale bar = 5 μm. Corresponds to Movie EV2.

D *Sting*$^{-/-}$ iBMDMs expressing eGFP-STING were imaged live on the spinning disk microscope. Images display a time series as indicated, showing STING vesicles exiting the Golgi for a recording starting 30 min after 50 μg/ml DMXAA treatment. Data are shown as a MIP of Z stack images. Data shown are representative of three independent experiments. Scale bar = 5 μm. Corresponds to Movie EV5.

Source data are available online for this figure.

 © 2023 The Authors

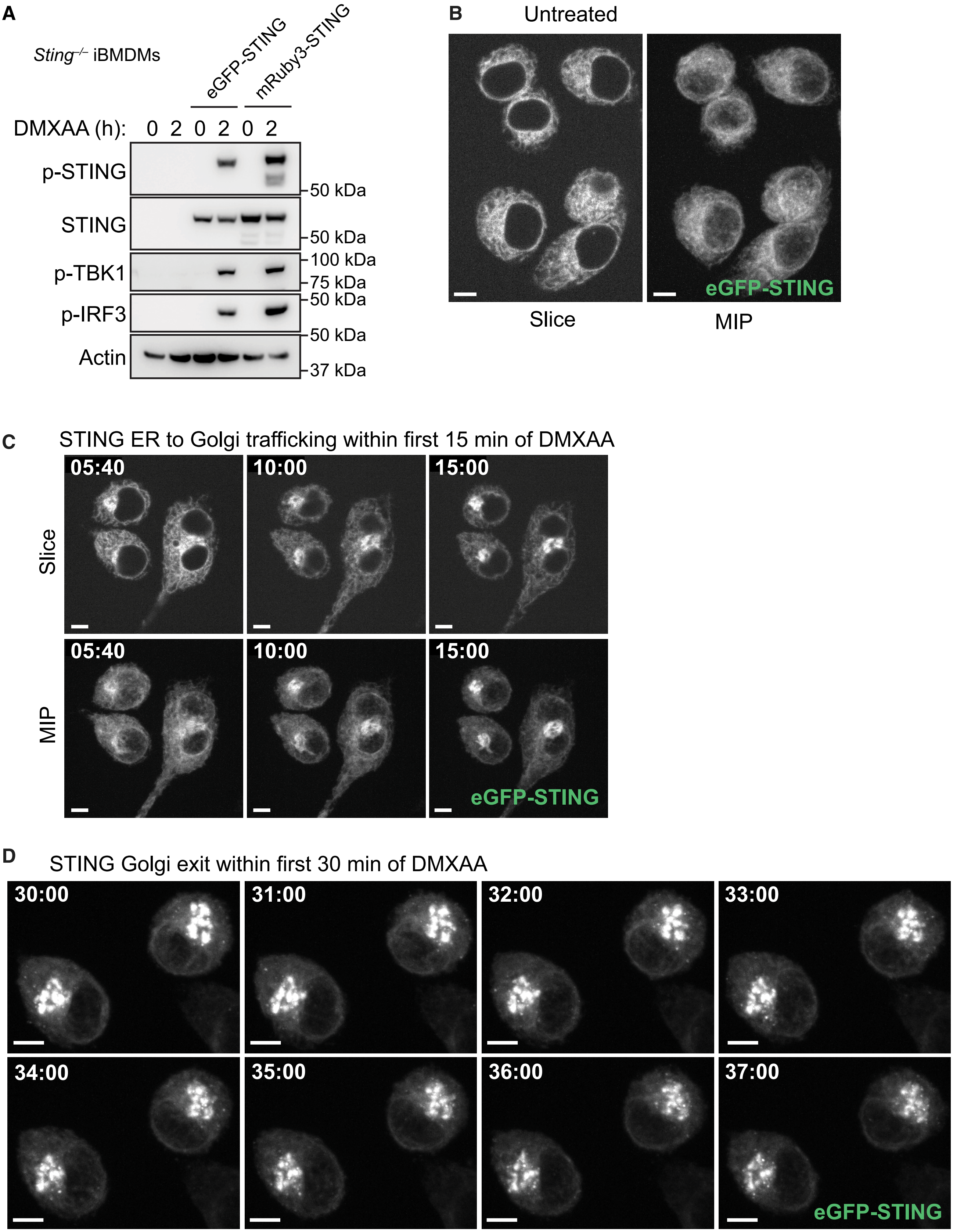
A
Sting−/− iBMDMs
eGFP-STING
mRuby3-STING
DMXAA (h): 0 2 0 2 0 2
p-STING
STING
p-TBK1
p-IRF3
Actin
50 kDa
50 kDa
100 kDa
75 kDa
50 kDa
50 kDa
37 kDa
B
Untreated
Slice
MIP
eGFP-STING
C
STING ER to Golgi trafficking within first 15 min of DMXAA
Slice
MIP
05:40
10:00
15:00
D
STING Golgi exit within first 30 min of DMXAA
30:00
31:00
32:00
33:00
34:00
35:00
36:00
37:00

Figure EV3.

Figure EV4. STING interacts with HRS.

A WT or TBK1/IKKε^{dKO} iBMDMs were left untreated (i.e. 0 h) or treated with 50 μg/ml DMXAA for 1 or 3 h. Cells were lysed and a portion of lysate underwent immunoblot with the indicated antibodies (i.e. lysate). The remaining lysate was incubated with TUBE beads to isolate ubiquitinated proteins (i.e. TUBE). TUBE samples underwent immunoblot with the indicated antibodies. Data representative of three independent experiments.

B *Sting*$^{-/-}$ iBMDMs or *Sting*$^{-/-}$ iBMDMs expressing HA-STING were left untreated or treated with 50 μg/ml DMXAA for 3 h. Cells were lysed and a portion of lysate underwent immunoblot with the indicated antibodies (i.e. lysate). The remaining lysate underwent immunoprecipitation with an anti-HA antibody (i.e. HA pull down). Samples then underwent immunoblot with the indicated antibodies. Data representative of three independent experiments.

C Primary BMDMs were left untreated or treated with 50 μg/ml DMXAA for 3 h. Cells were lysed and a portion of lysate underwent immunoblot with the indicated antibodies (i.e. lysate). The remaining lysate underwent immunoprecipitation with an anti-STING antibody (i.e. STING pull down). Samples then underwent immunoblot with the indicated antibodies. Data representative of three independent experiments.

D *Sting*$^{-/-}$ iBMDMs expressing eGFP-STING (yellow) were treated with 50 μg/ml DMXAA for 3 h. Cells were fixed and underwent immunofluorescence staining for conjugated ubiquitin (Ub; magenta) and HRS (cyan). Data show two channel merged images relating to those found in Fig 5D. Scale bar = 5 μm.

Source data are available online for this figure.

 © 2023 The Authors

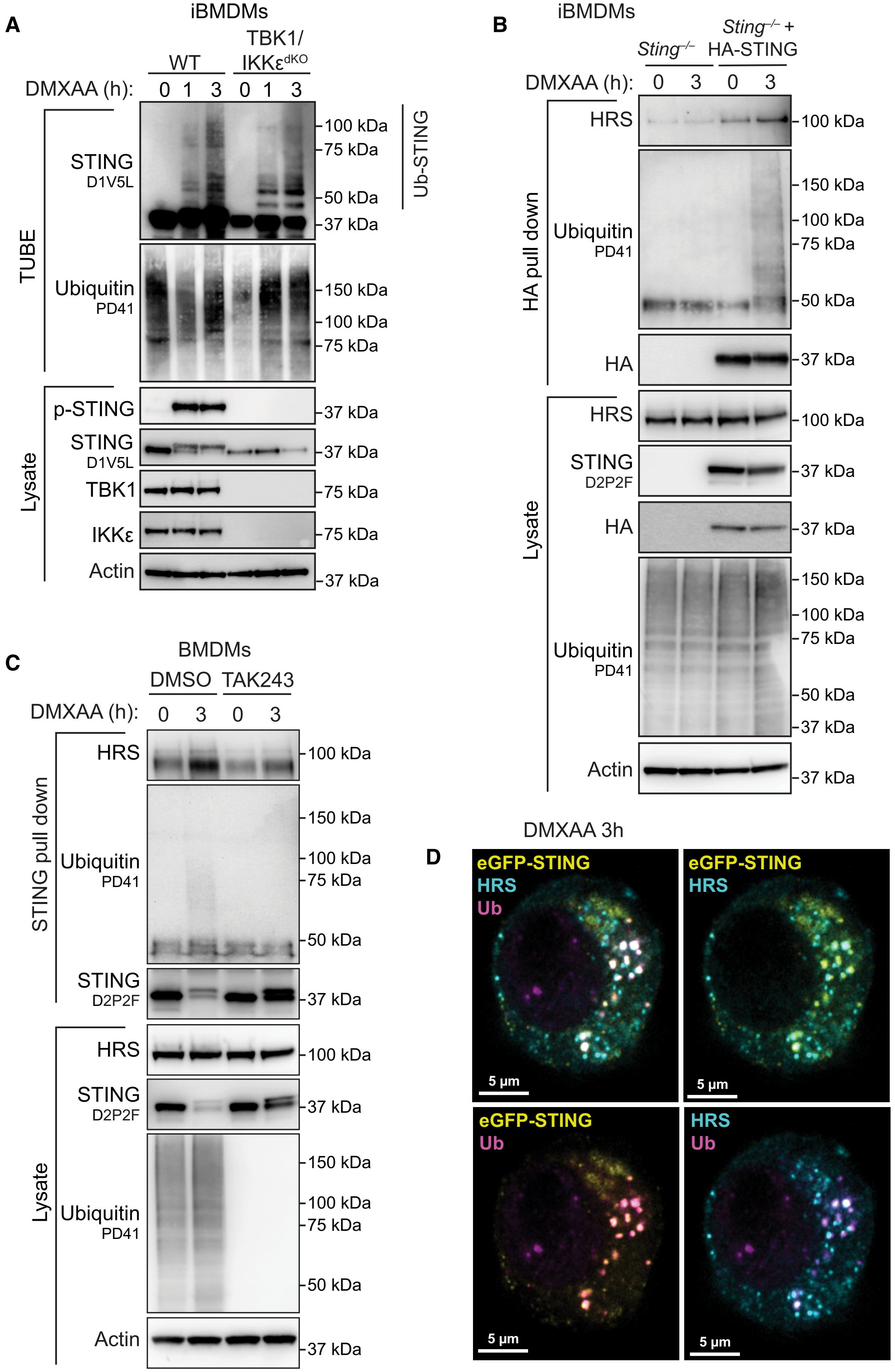
A
iBMDMs
WT
TBK1/ IKKε^{dKO}
DMXAA (h):
0 1 3 0 1 3
TUBE
STING D1V5L
Ubiquitin PD41
Ub-STING
Lysate
p-STING
STING D1V5L
TBK1
IKKε
Actin
100 kDa
75 kDa
50 kDa
37 kDa
150 kDa
B
iBMDMs
Sting$^{-/-}$
Sting$^{-/-}$ + HA-STING
DMXAA (h):
0 3 0 3
HA pull down
HRS
Ubiquitin PD41
HA
Lysate
STING D2P2F
Actin
C
BMDMs
DMSO
TAK243
STING pull down
D
DMXAA 3h
eGFP-STING
HRS
Ub
5 µm

Figure EV4.

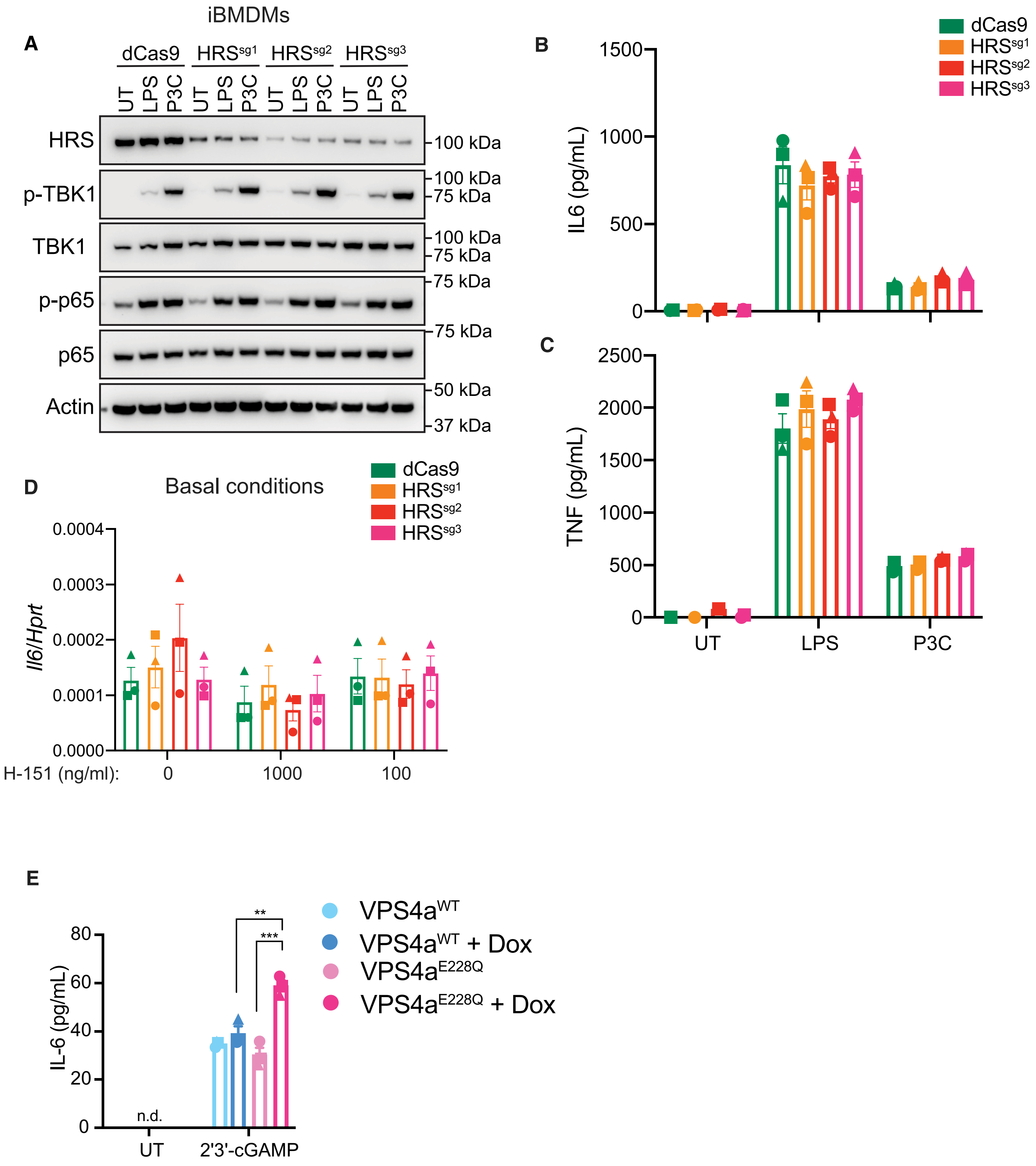

Figure EV5.

 © 2023 The Authors

Figure EV5. HRS-depleted iBMDMs maintain normal TLR responses.

A–C dCas9-KRAB expressing iBMDMs, without (i.e. dCas9 alone) or with HRS-targeting sgRNAs (i.e. sg1, sg2 or sg3) were treated for 48 h with 1 μg/ml doxycycline (Dox) to induce sgRNA expression. Cells were then left untreated (UT), or treated with 500 ng/ml LPS or 200 ng/ml Pam3CSK4 (P3C) for 6 h. A Cells were lysed for immunoblot with the indicated antibodies. Data shown are representative of three independent experiments. (B, C) Cell supernatant was collected and assayed for secreted IL-6 (B) or TNF (C) by ELISA. Data are shown as mean ± SEM combined from $N = 3$ independent experiments. Statistical analysis was performed using two-way ANOVA using Bonferroni's multiple comparisons test, where no statically significant differences were observed across cell lines for the same treatment.

D dCas9-KRAB expressing iBMDMs, without (i.e. dCas9 alone) or with HRS-targeting sgRNAs (i.e. sg1, sg2 or sg3) were treated for 24 h with 1 μg/ml Dox to induce sgRNA expression. iBMDMs were further treated with 1 μg/ml (i.e., 1,000 ng/ml) or 100 ng/ml H-151 as indicated and incubated for a further 24 h. Cells were lysed for RNA purification and the expression of *Il6* was analysed by qPCR. Data are shown as mean ± SEM combined from $N = 3$ independent experiments. Statistical analysis was performed using two-way ANOVA using Bonferroni's multiple comparisons test, where no statistically significant changes were observed.

E VPS4a^{WT} or VPS4a^{E228Q} dominant negative BMDMs were left UT or treated with 1 μg/ml Dox for 4 h. iBMDMs were then further left UT or treated with 10 μg/ml 2′3′-cGAM(PS)2 for 4 h. Cell supernatant was collected and secreted IL-6 was measured by ELISA. Data are shown as mean ± SEM combined from $N = 3$ independent experiments. Statistical analysis was performed using one-way ANOVA using Bonferroni's multiple comparisons test, where $^{**}P < 0.01$, $^{***}P < 0.001$. n.d., not detected.

Source data are available online for this figure.

© 2023 The Authors

Appendix

Supplementary figures

Page

**Appendix Figure S1: Phosphoproteomic analysis of primary BMDMs following STING activation**	**2**
**Appendix Figure S2: STING ER-Golgi and vesicular trafficking**	**3**
**Appendix Figure S3: STING interacts with HRS over time in a ubiquitin-dependent fashion**	**4**
**Appendix Figure S4: STING colocalises with HRS and ubiquitin**	**5**

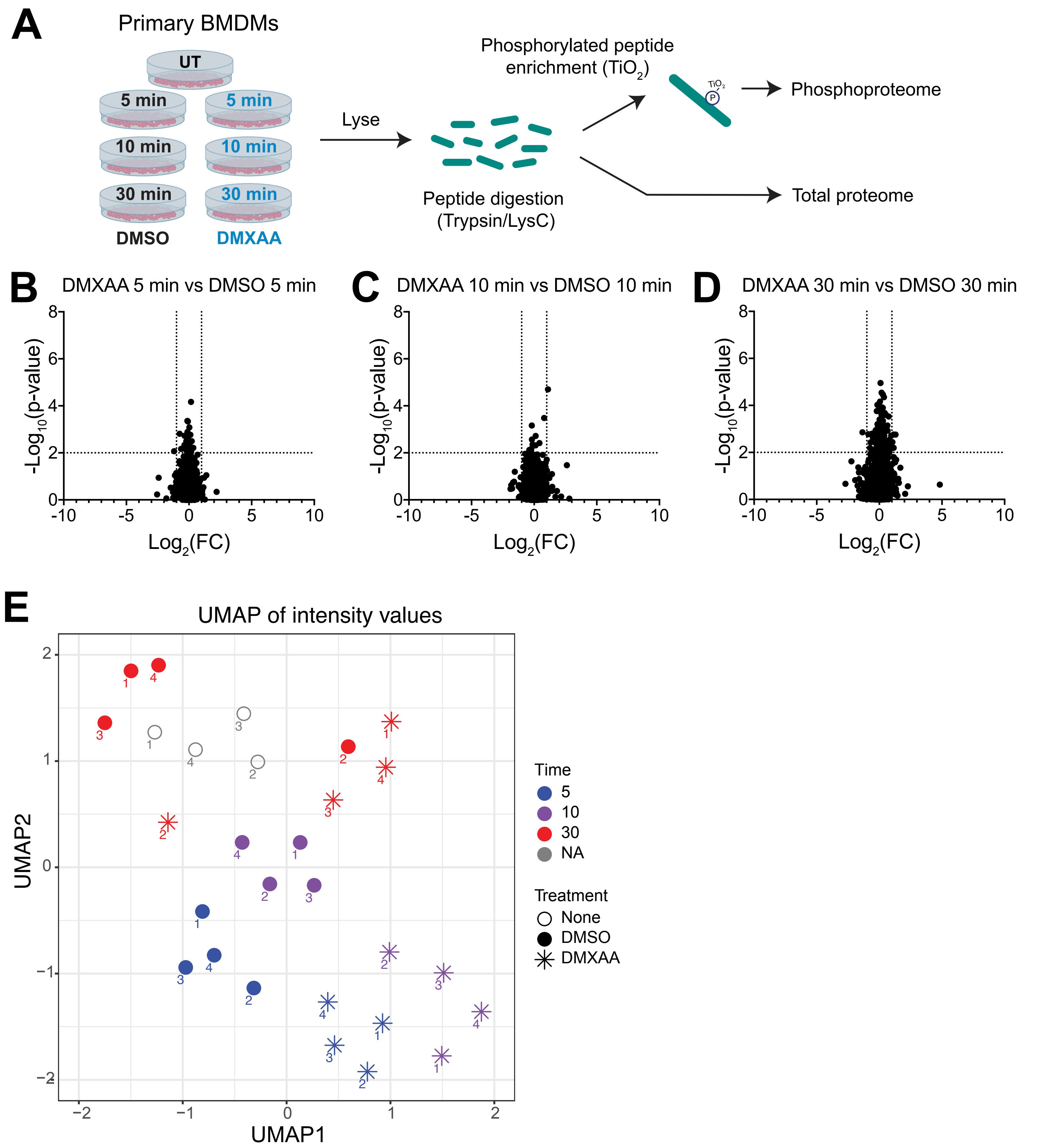

Appendix Figure S1: Phosphoproteomic analysis of primary BMDMs following STING activation

A Schematic showing phosphoproteomic experimental design by primary BMDMs were left untreated (UT) or treated with DMSO (vehicle control) or 50 µg/mL DMXAA for 5, 10 or 30 min. A portion of digest peptides were used for total proteome analysis, while the rest underwent enrichment with titanium oxide beads for phosphorylated peptides, and analysis of the phosphoproteome.

B-D Volcano plots showing changes in total proteome levels for DMXAA vs DMSO groups for 5 min (**B**), 10 min (**C**) and 30 min (**D**) timepoints. -Log10(p-value) is plotted on the y-axis vs Log2(Fold change[FC]) on the x-axis.

E Phosphoproteomic samples were visualised via uniform manifold approximation and projection (UMAP).

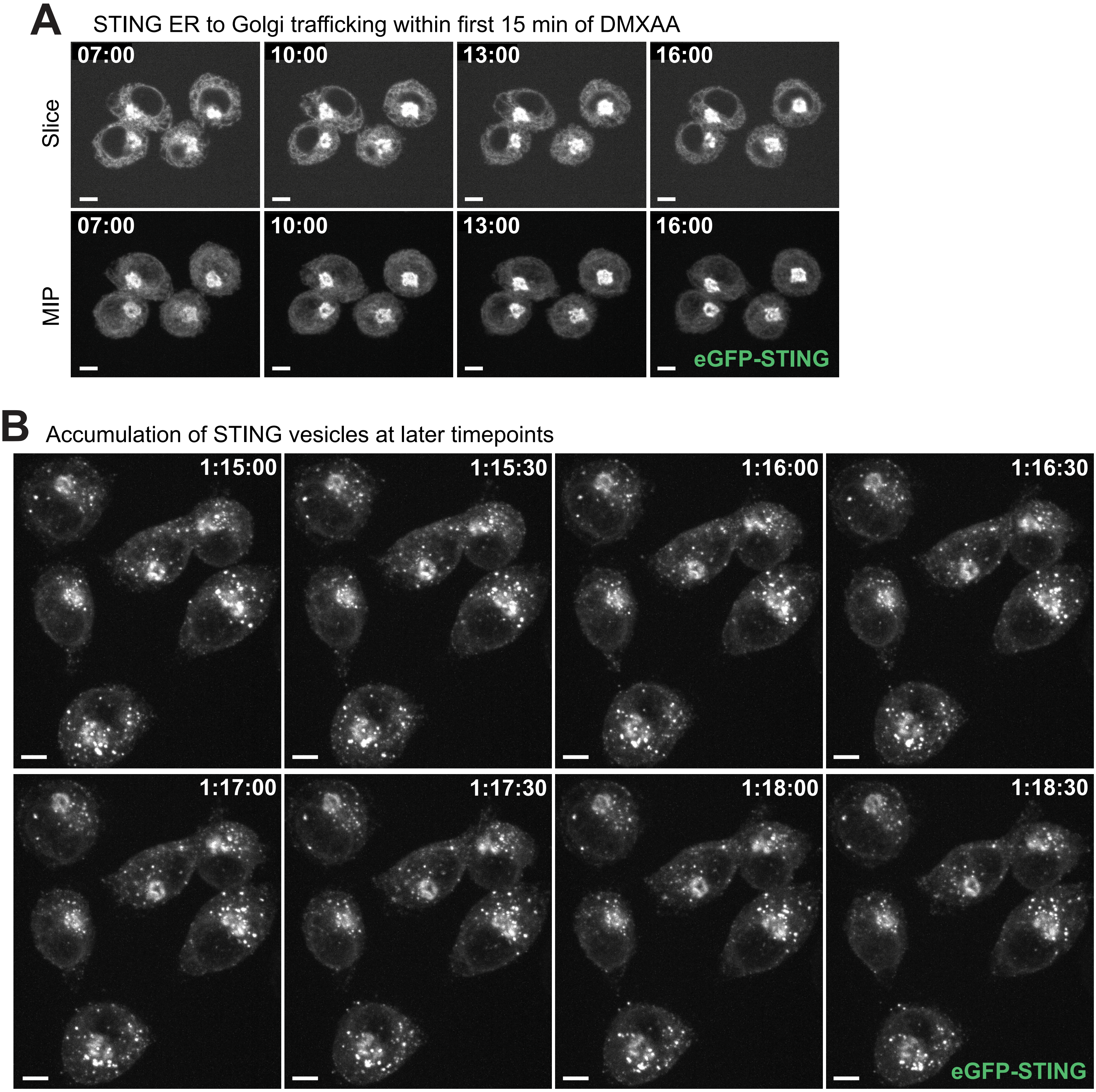

Appendix Figure S2: STING ER-Golgi and vesicular trafficking

A *Sting*$^{-/-}$ iBMDMs expressing eGFP-STING were imaged live on the spinning disk microscope. Images display a time series as indicated, showing STING translocation from ER to Golg after 50 µg/mL DMXAA treatment. Data is shown as a maximum intensity projection (MIP) of Z stack images. Scale bar = 5 µm. Data shown is representative of 3 independent experiments.

B *Sting*$^{-/-}$ iBMDMs expressing eGFP-STING were imaged live on the spinning disk microscope. Images display a time series as indicated, showing transport of STING vesicles for a recording starting 1 h 15 min after 50 µg/mL DMXAA treatment. Data is shown as a maximum intensity projection (MIP) of Z stack images. Scale bar = 5 µm. Data shown is representative of 3 independent experiments.

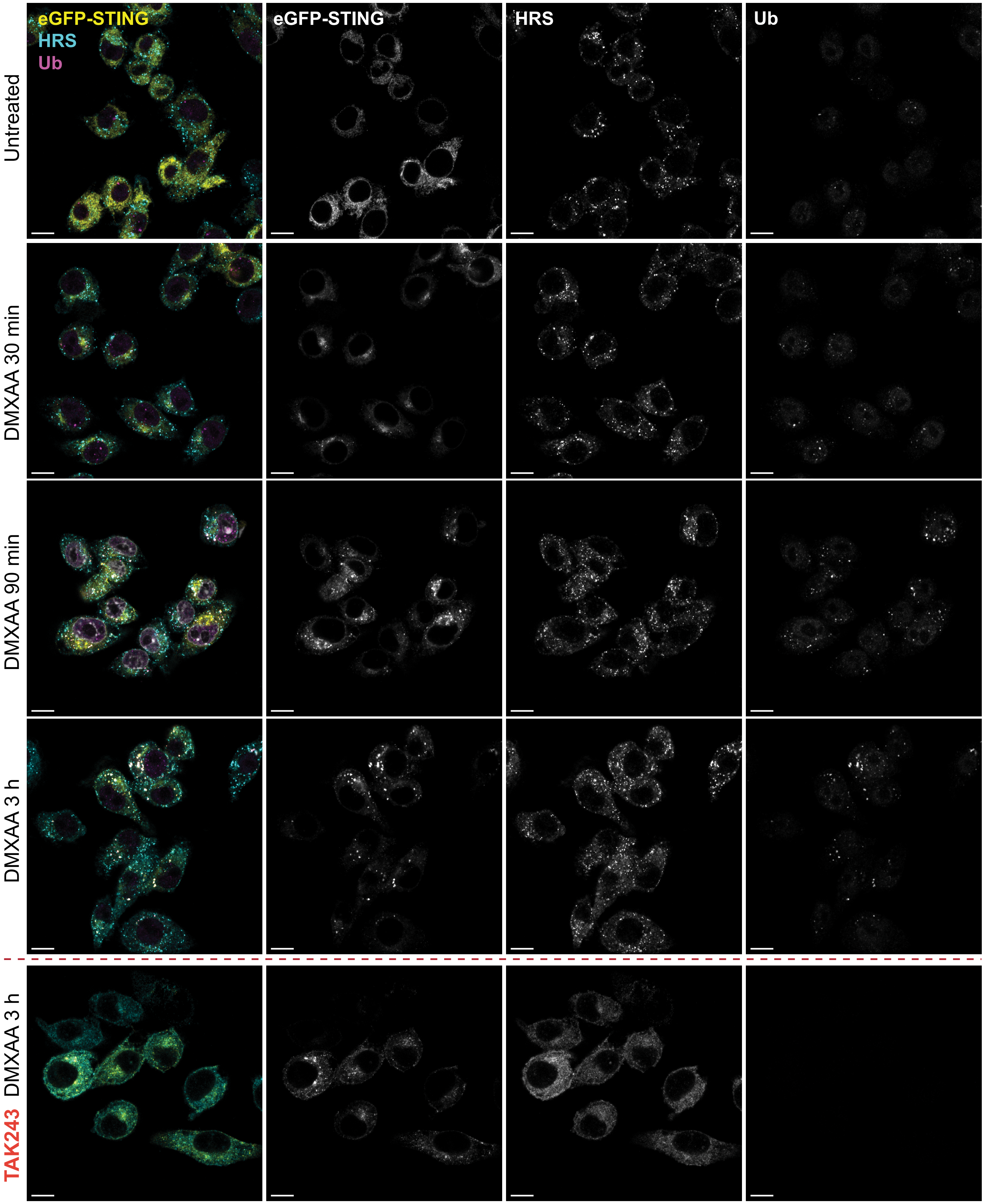

Appendix Figure S3: STING interacts with HRS over time in a ubiquitin-dependent fashion
Sting$^{-/-}$ iBMDMs expressing eGFP-STING (yellow) were left untreated or treated with 50 µg/mL DMXAA for 30 min, 90 min or 3 h. Cells were additionally treated with 2 µM TAK243 (as indicated in red) for 30 min before 50 µg/mL DMXAA for 3 h. Cells were then fixed and underwent immunofluorescence staining for conjugated ubiquitin (Ub; magenta) and HRS (cyan). Z stack images were acquired on the LSM980 confocal microscope. Images are displayed as merged and single colour images for a single Z slice. Scale bar = 10 µm. Data shown is representative of 3 independent experiments. The untreated merged image is also shown in Figure 5C.

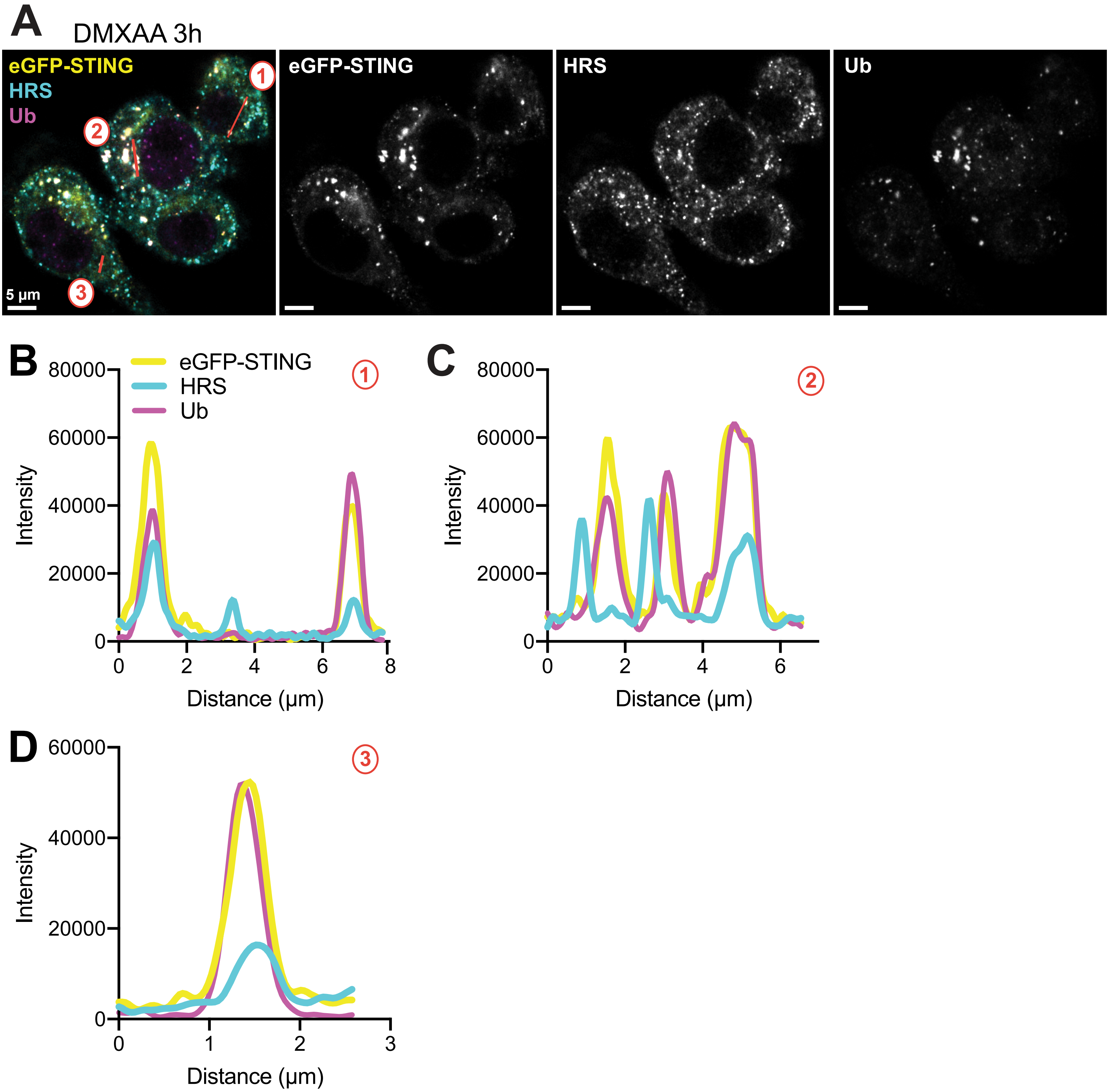

Appendix Figure S4: STING colocalises with HRS and ubiquitin
A *Sting*⁻/⁻ iBMDMs expressing eGFP-STING (yellow) were left untreated or treated with 50 µg/mL DMXAA for 3 h. Cells were fixed and underwent immunofluorescence staining for conjugated ubiquitin (Ub; magenta) and HRS (cyan). Z stack images were acquired on the LSM980 confocal microscope. Images are displayed as merged images for a single Z slice. Scale bar = 5 µm. Data shown is a zoomed in region from **Appendix Figure S3**, displayed as both merged and single colour images.
B-D The intensity profiles were plotted for each channel for three-line regions as indicated in **A**. This provides an additional example to the data shown in **Figure 5D-F**
