# Supplementary material for: Termination of STING responses is mediated via ESCRT‐dependent degradation
Source: EMBO J. 2023 May 4;42(12):e112712. doi: 10.15252/embj.2022112712 (PMC10267698; doi:10.15252/embj.2022112712)
Supplement: Supplementary file 15 — Source Data for Figure 5 [file EMBJ-42-e112712-s014.zip › Figure 5/Figure 5A.pdf]

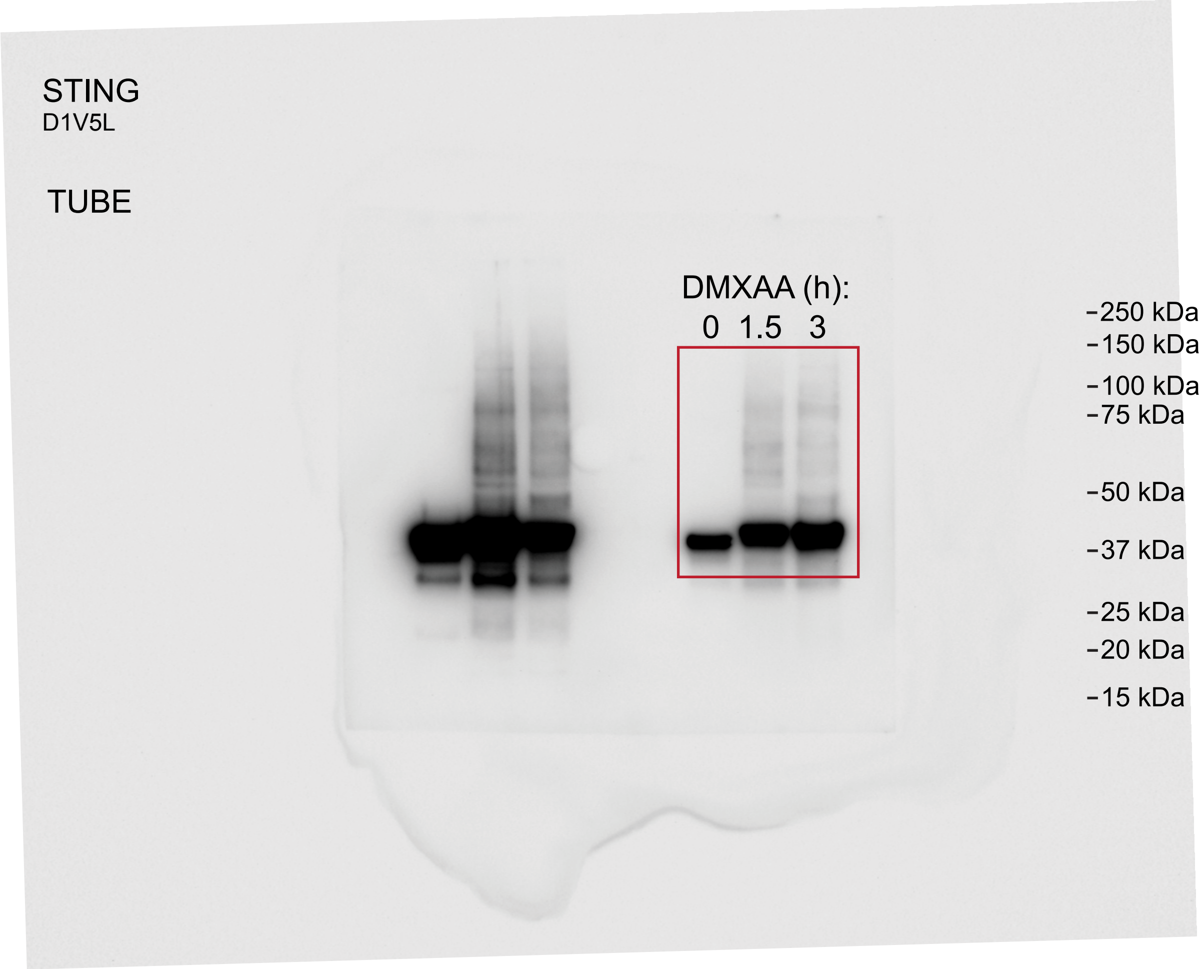
STING
D1V5L
TUBE
DMXAA (h):
0 1.5 3
-250 kDa
-150 kDa
-100 kDa
-75 kDa
-50 kDa
-37 kDa
-25 kDa
-20 kDa
-15 kDa

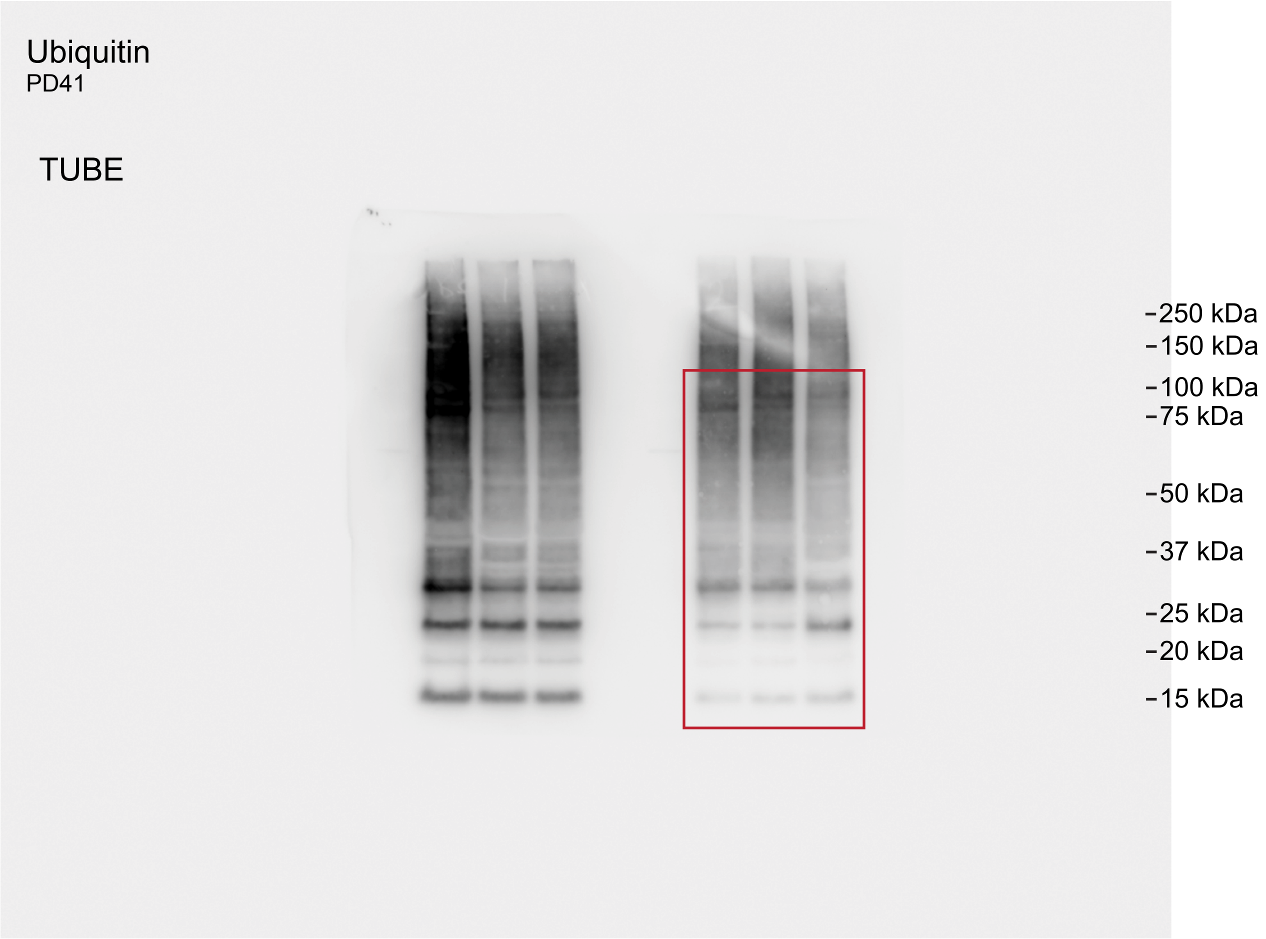
Ubiquitin
PD41
TUBE
-250 kDa
-150 kDa
-100 kDa
-75 kDa
-50 kDa
-37 kDa
-25 kDa
-20 kDa
-15 kDa

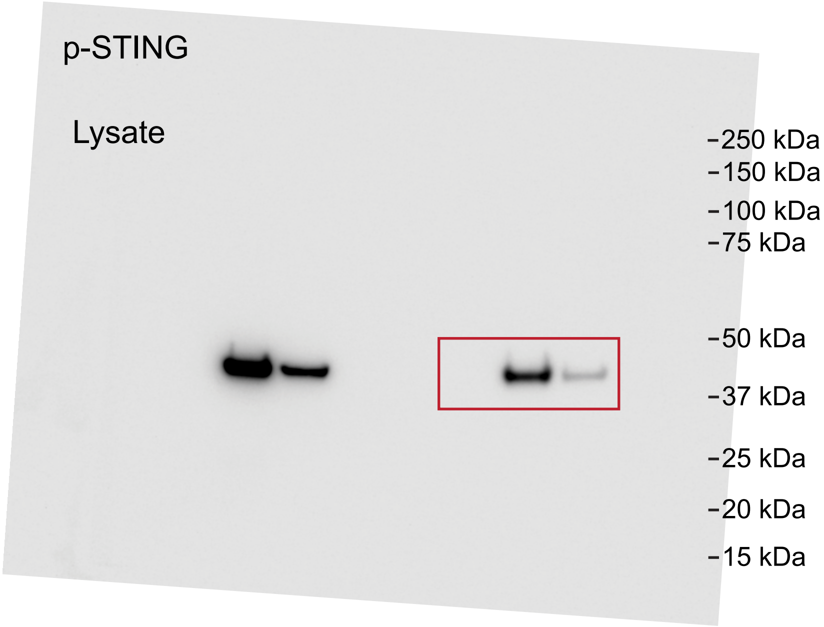
p-STING
Lysate
-250 kDa
-150 kDa
-100 kDa
-75 kDa
-50 kDa
-37 kDa
-25 kDa
-20 kDa
-15 kDa

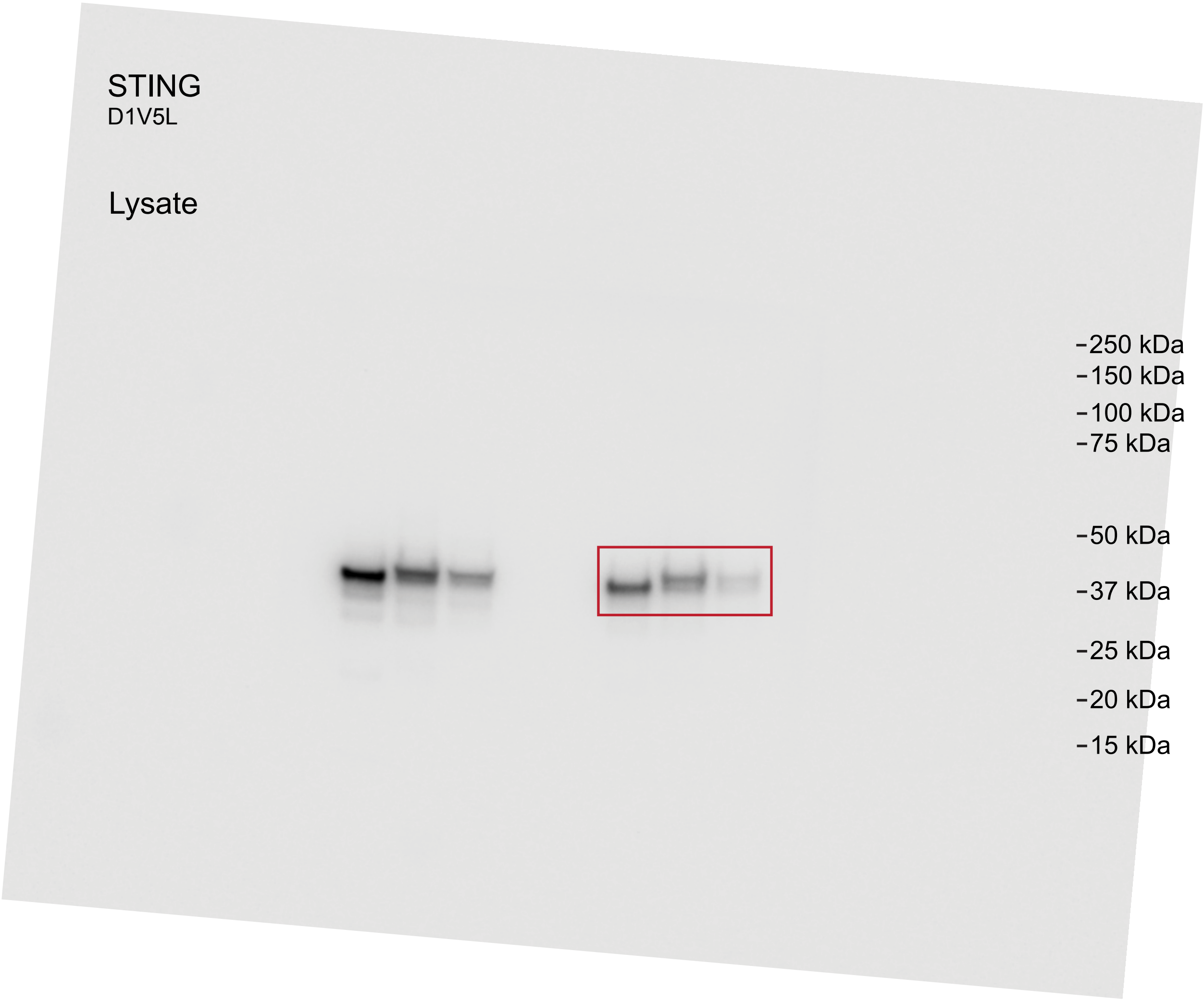
STING
D1V5L
Lysate
-250 kDa
-150 kDa
-100 kDa
-75 kDa
-50 kDa
-37 kDa
-25 kDa
-20 kDa
-15 kDa

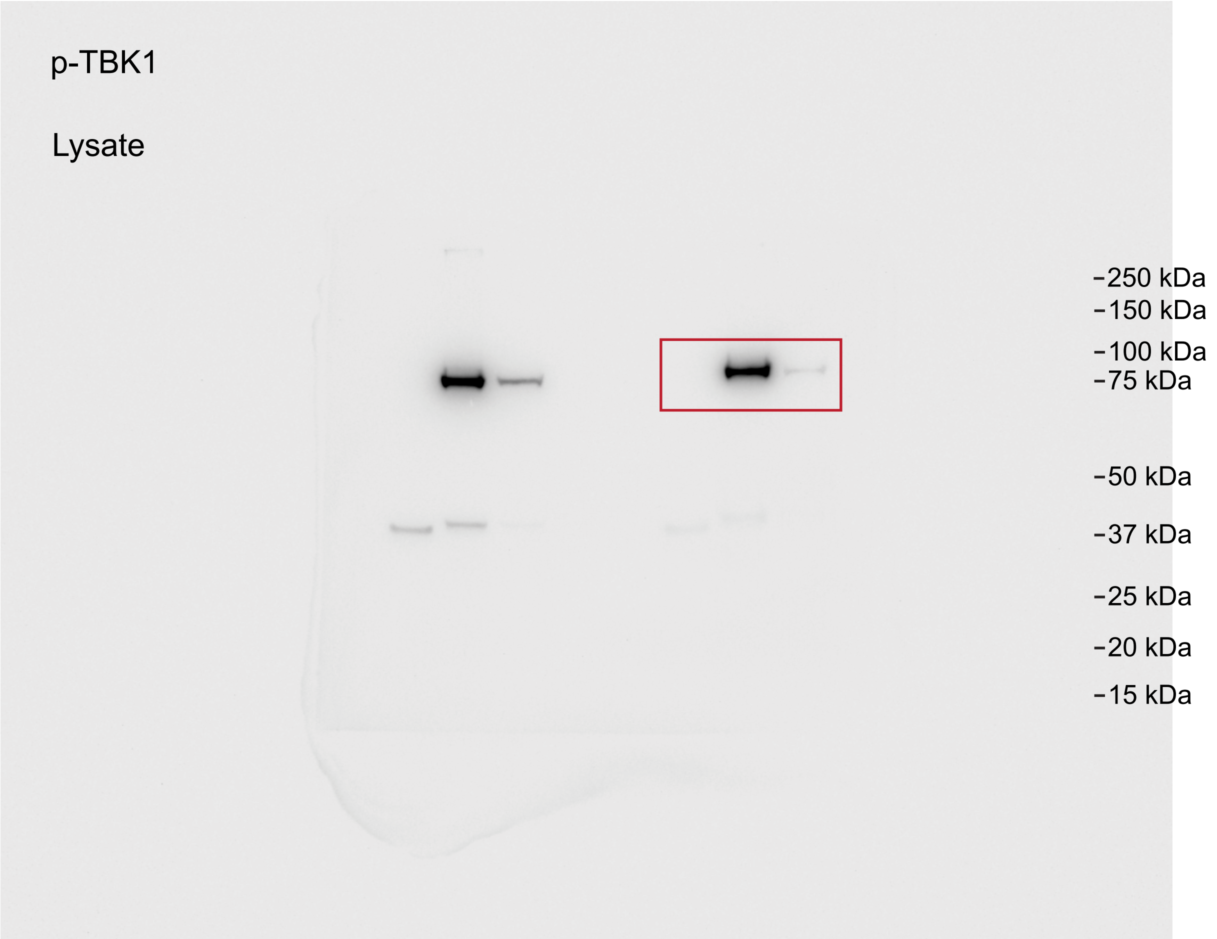
p-TBK1
Lysate
-250 kDa
-150 kDa
-100 kDa
-75 kDa
-50 kDa
-37 kDa
-25 kDa
-20 kDa
-15 kDa

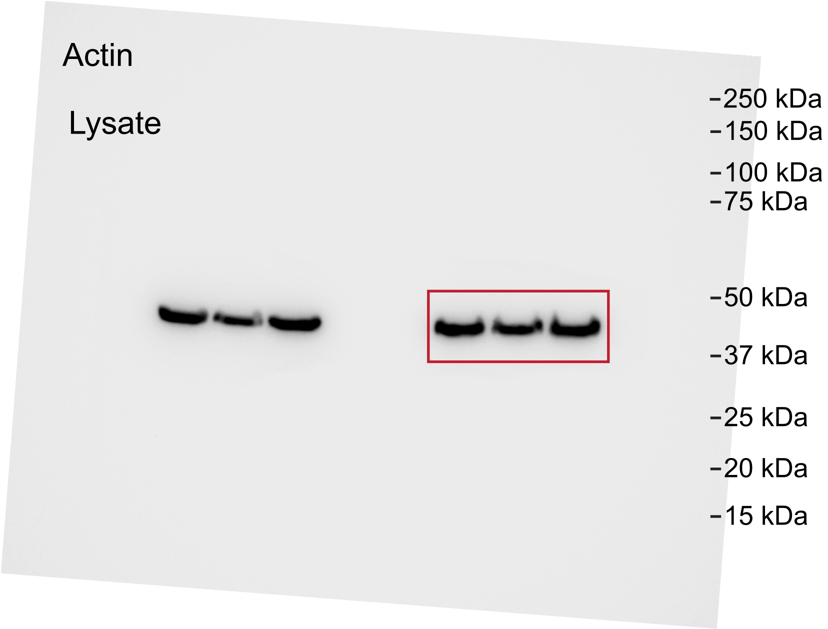
Actin
Lysate
-250 kDa
-150 kDa
-100 kDa
-75 kDa
-50 kDa
-37 kDa
-25 kDa
-20 kDa
-15 kDa
