# Supplementary material for: Termination of STING responses is mediated via ESCRT‐dependent degradation
Source: EMBO J. 2023 May 4;42(12):e112712. doi: 10.15252/embj.2022112712 (PMC10267698; doi:10.15252/embj.2022112712)
Supplement: Supplementary file 15 — Source Data for Figure 5 [file EMBJ-42-e112712-s014.zip › Figure 5/Figure 5B.pdf]

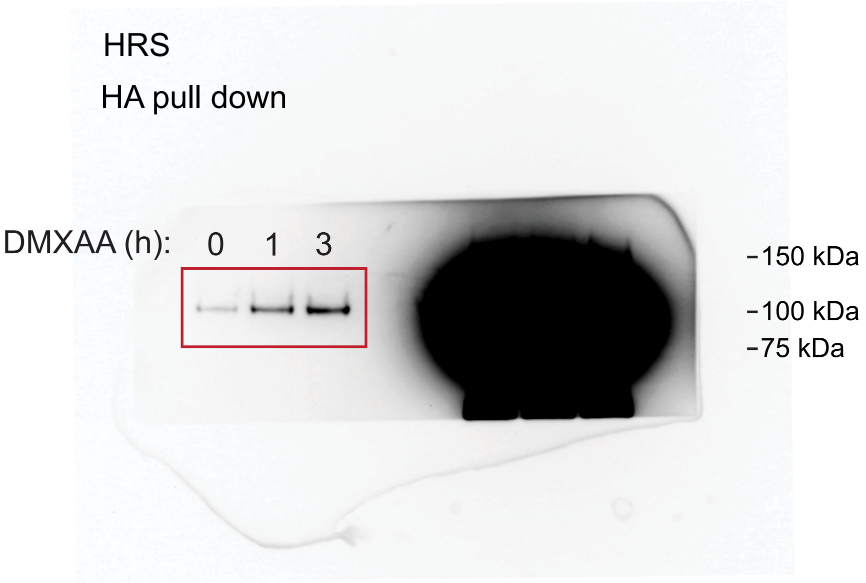
HRS
HA pull down
DMXAA (h): 0 1 3
-150 kDa
-100 kDa
-75 kDa

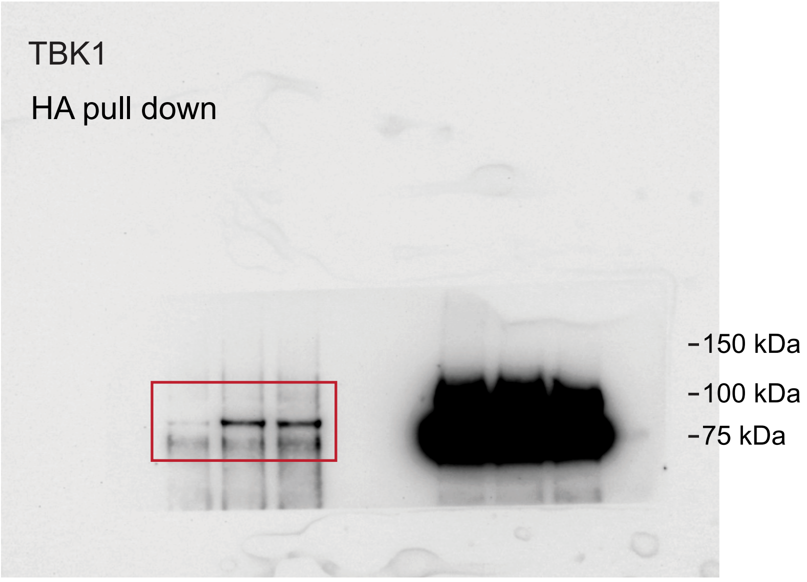
TBK1
HA pull down
-150 kDa
-100 kDa
-75 kDa

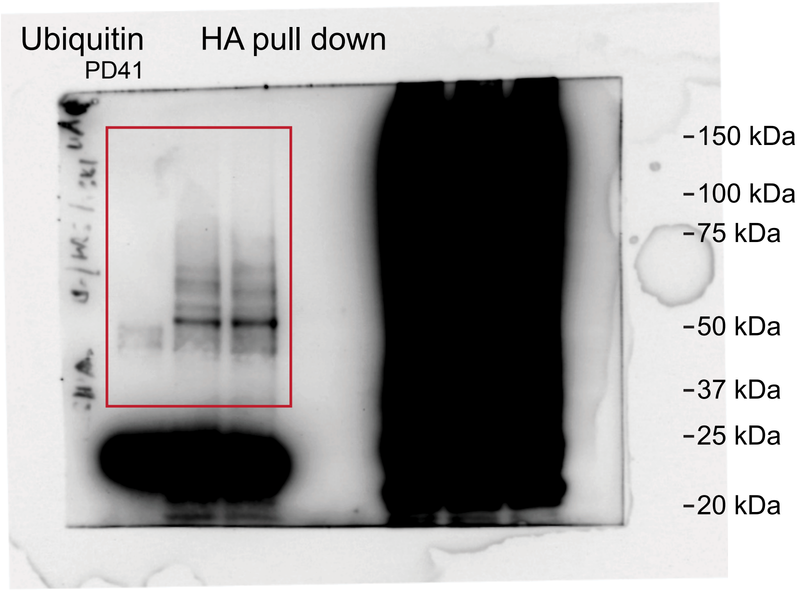
Ubiquitin
PD41
HA pull down
-150 kDa
-100 kDa
-75 kDa
-50 kDa
-37 kDa
-25 kDa
-20 kDa

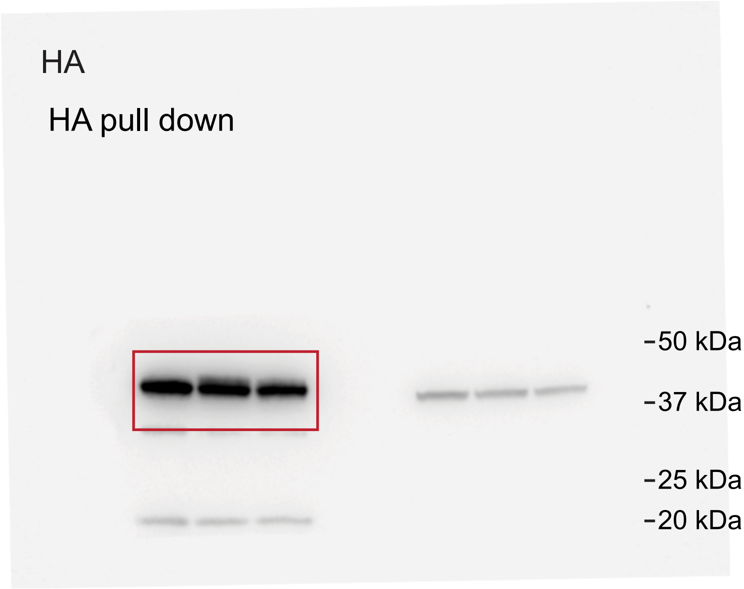
HA
HA pull down
-50 kDa
-37 kDa
-25 kDa
-20 kDa

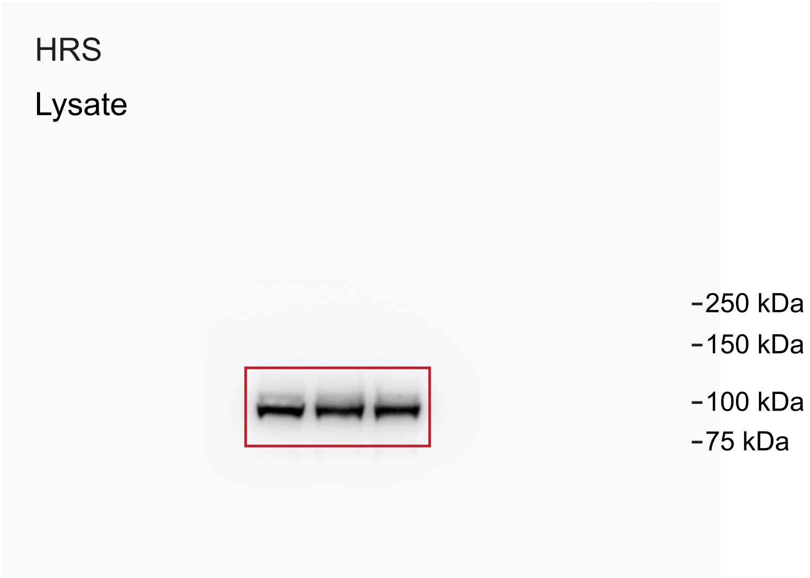
HRS
Lysate
-250 kDa
-150 kDa
-100 kDa
-75 kDa

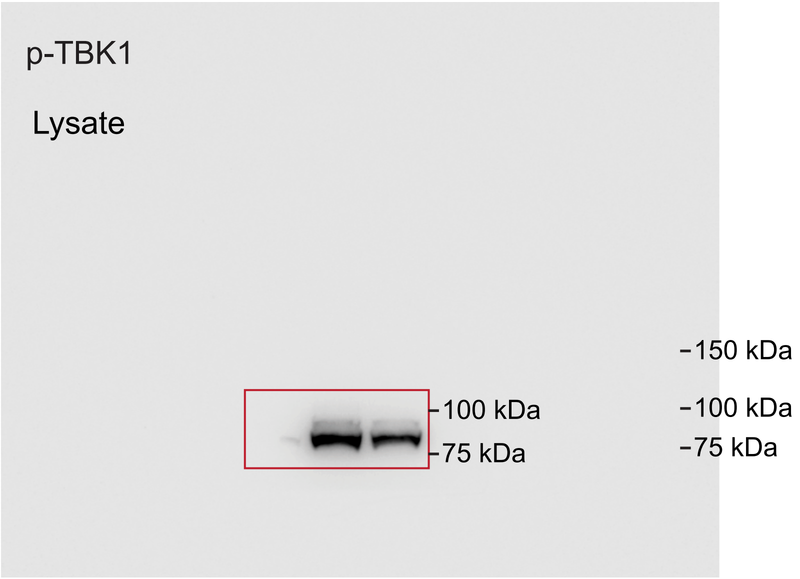
p-TBK1
Lysate
-100 kDa
-75 kDa
-150 kDa
-100 kDa
-75 kDa

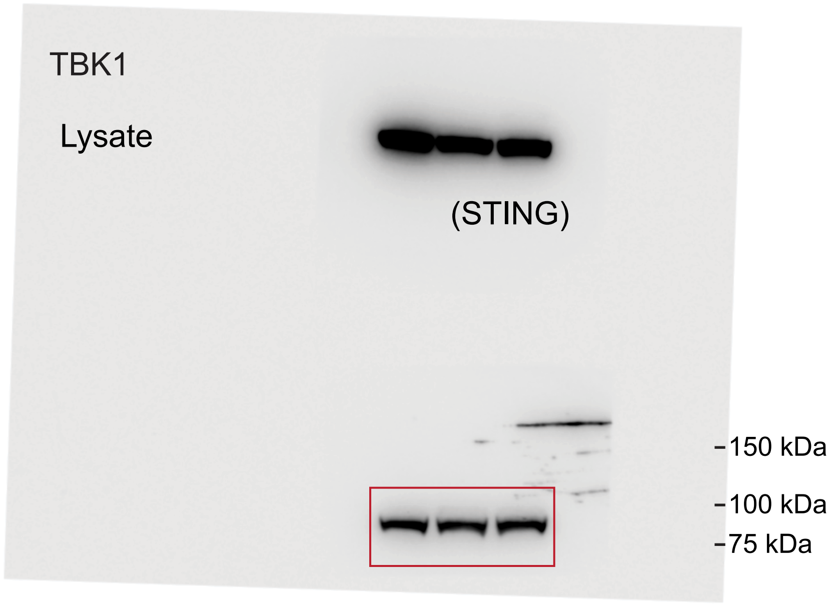
TBK1
Lysate
(STING)
150 kDa
100 kDa
75 kDa

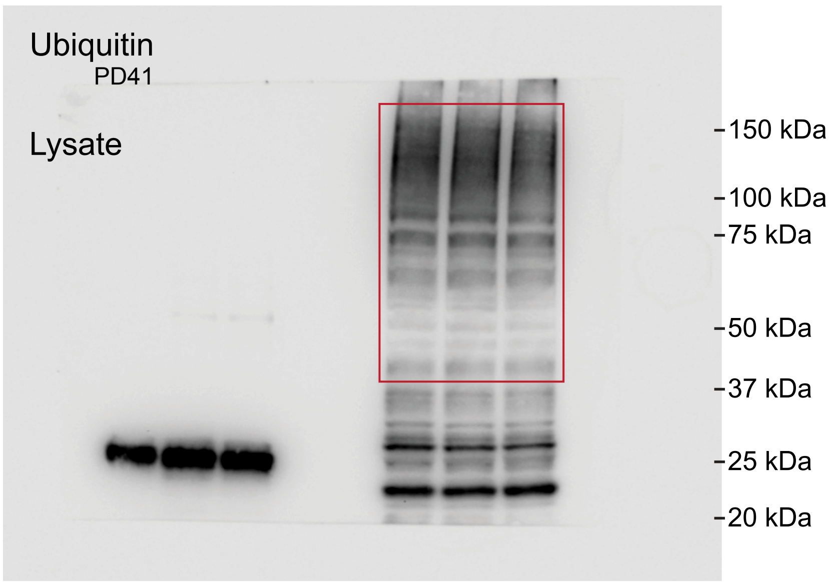
Ubiquitin
PD41
Lysate
150 kDa
100 kDa
75 kDa
50 kDa
37 kDa
25 kDa
20 kDa

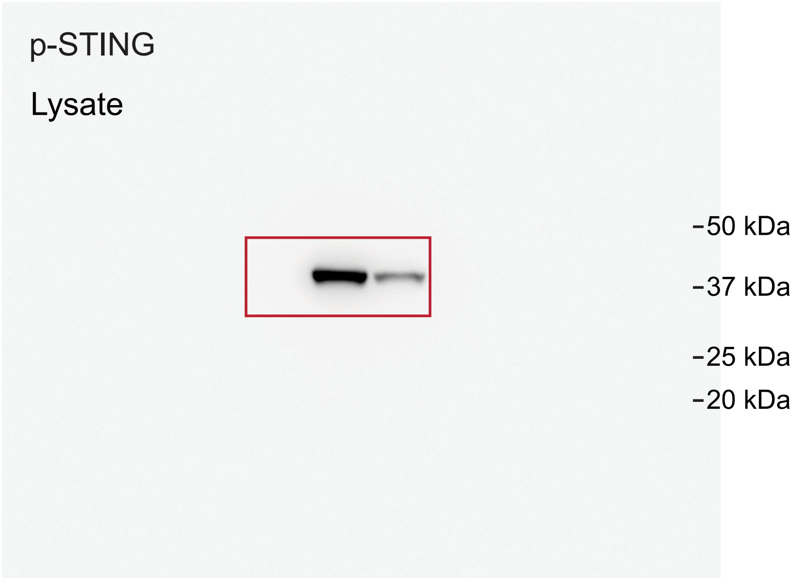
p-STING
Lysate
50 kDa
37 kDa
25 kDa
20 kDa

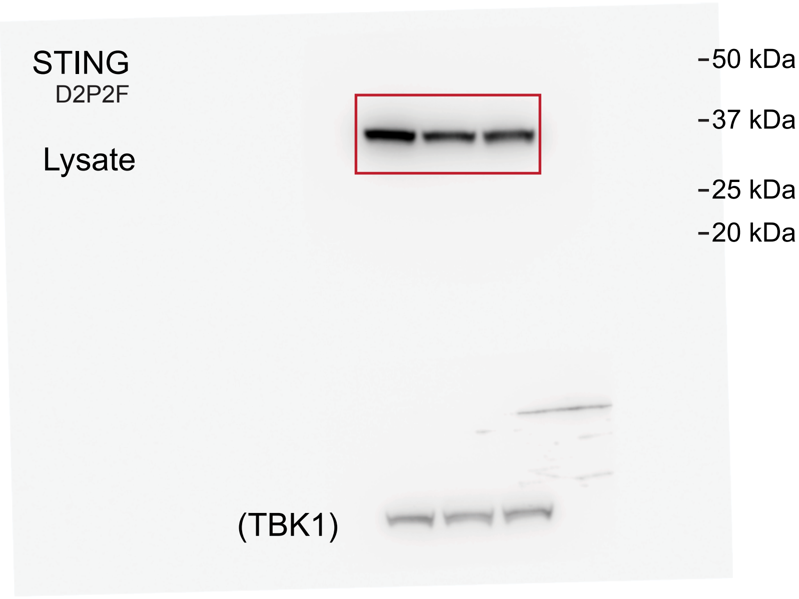
STING
D2P2F
Lysate
(TBK1)
-50 kDa
-37 kDa
-25 kDa
-20 kDa

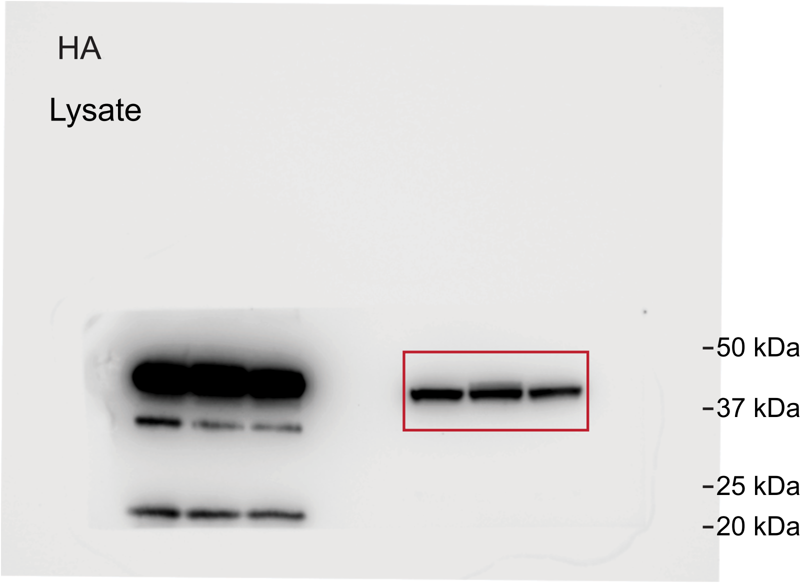
HA
Lysate
-50 kDa
-37 kDa
-25 kDa
-20 kDa

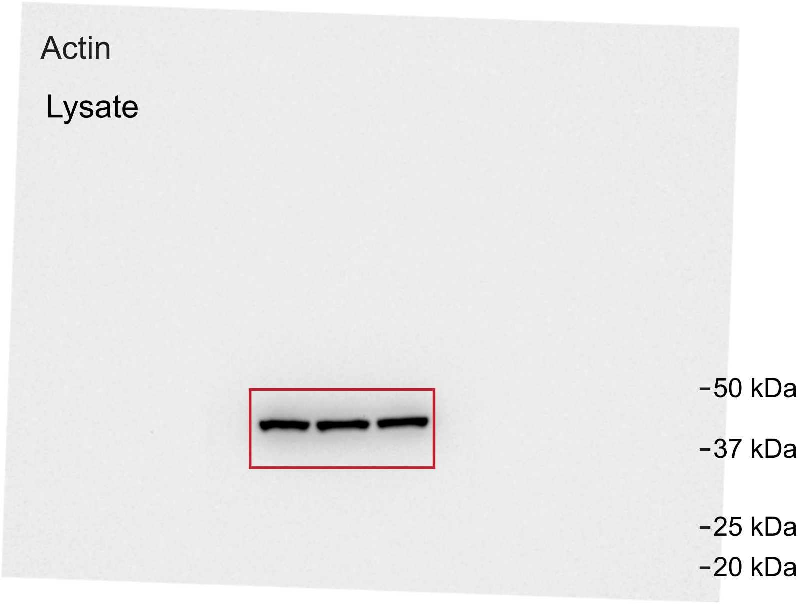
Actin
Lysate
-50 kDa
-37 kDa
-25 kDa
-20 kDa
