# Supplementary material for: Termination of STING responses is mediated via ESCRT‐dependent degradation
Source: EMBO J. 2023 May 4;42(12):e112712. doi: 10.15252/embj.2022112712 (PMC10267698; doi:10.15252/embj.2022112712)
Supplement: Supplementary file 16 — Source Data for Figure 6 [file EMBJ-42-e112712-s002.zip › Figure 6/Figure 6A.pdf]

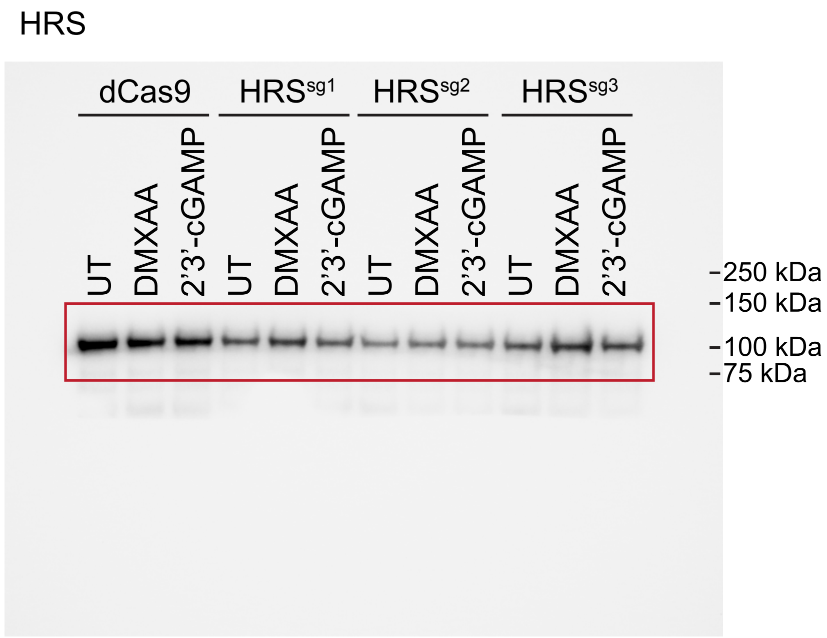
HRS
dCas9
HRSsg1
HRSsg2
HRSsg3
UT
DMXAA
2'3'-cGAMP
UT
DMXAA
2'3'-cGAMP
UT
DMXAA
2'3'-cGAMP
UT
DMXAA
2'3'-cGAMP
250 kDa
150 kDa
100 kDa
75 kDa

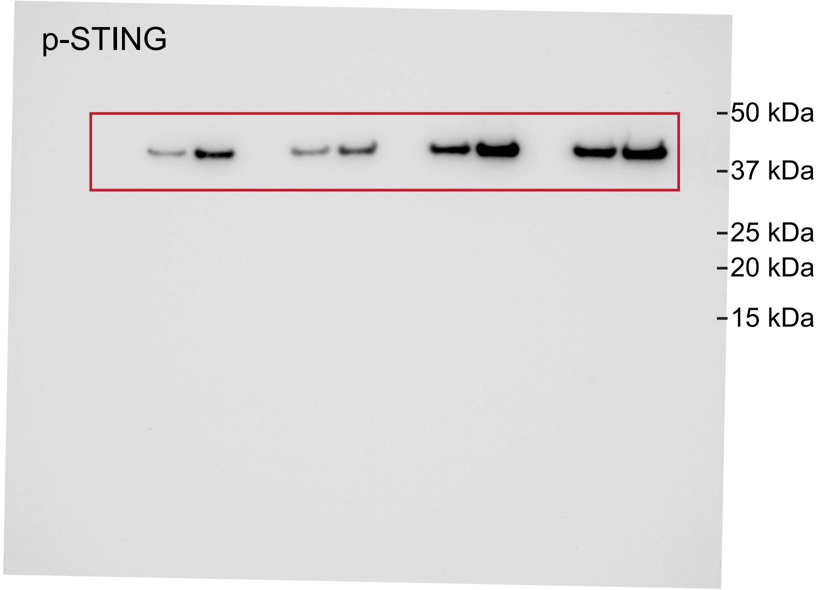
p-STING
50 kDa
37 kDa
25 kDa
20 kDa
15 kDa

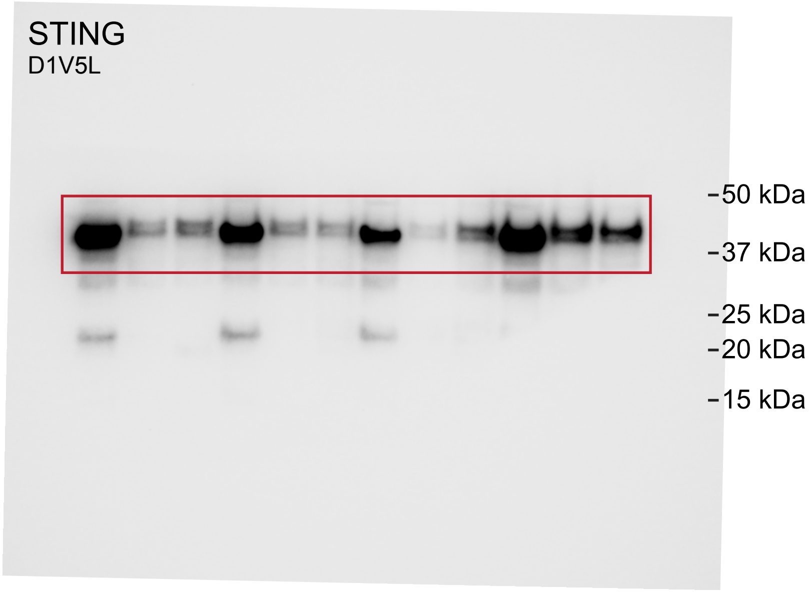
STING
D1V5L
50 kDa
37 kDa
25 kDa
20 kDa
15 kDa

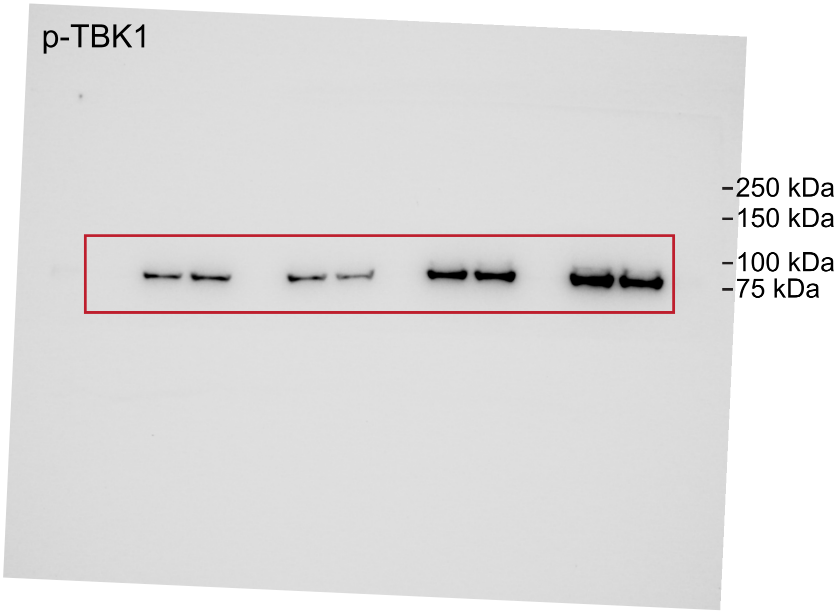
p-TBK1
250 kDa
150 kDa
100 kDa
75 kDa

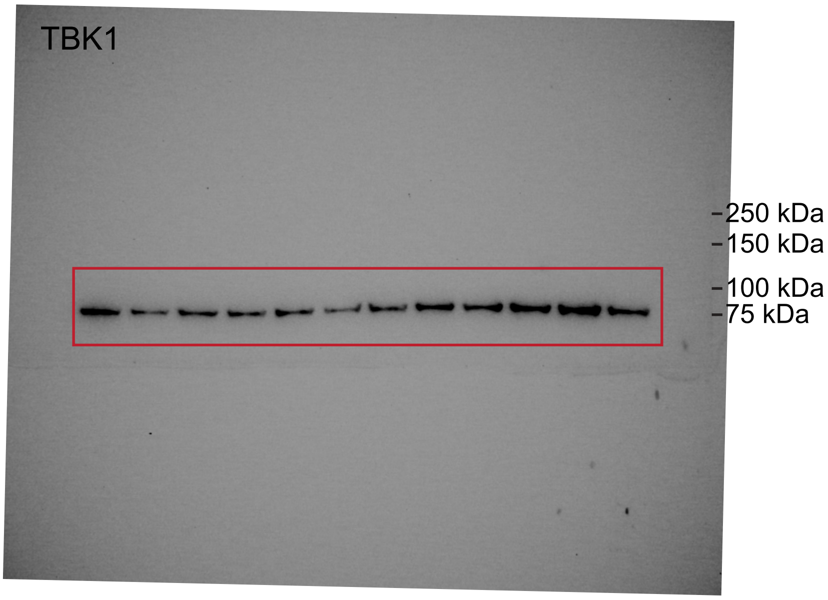
TBK1
250 kDa
150 kDa
100 kDa
75 kDa

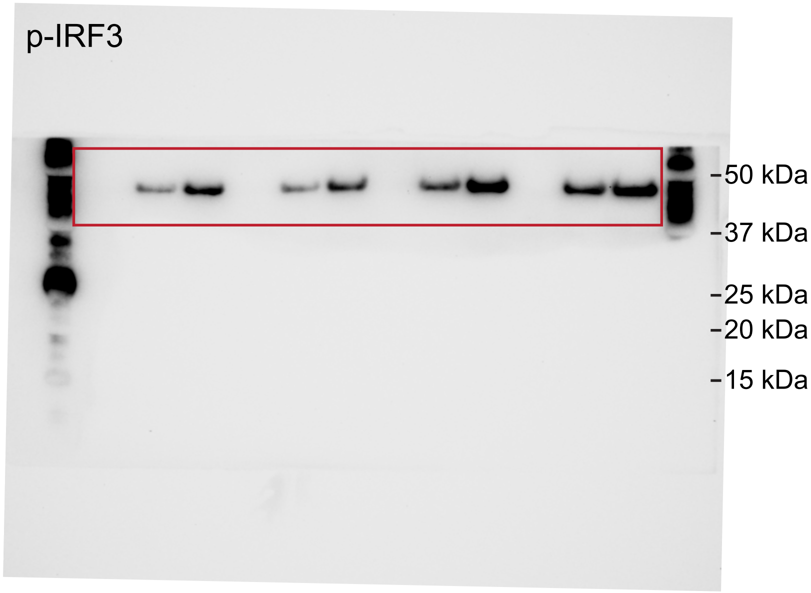
p-IRF3
50 kDa
37 kDa
25 kDa
20 kDa
15 kDa

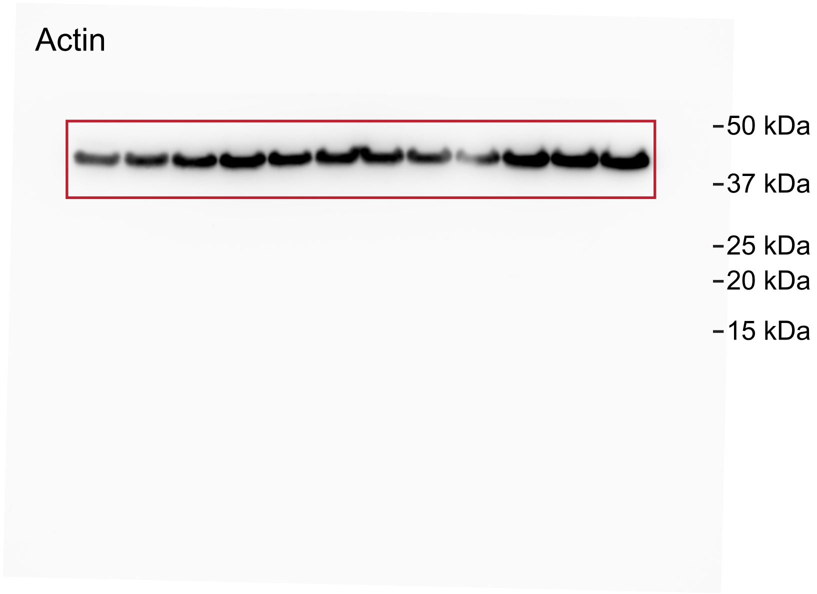
Actin
50 kDa
37 kDa
25 kDa
20 kDa
15 kDa
