# Supplementary material for: Termination of STING responses is mediated via ESCRT‐dependent degradation
Source: EMBO J. 2023 May 4;42(12):e112712. doi: 10.15252/embj.2022112712 (PMC10267698; doi:10.15252/embj.2022112712)
Supplement: Supplementary file 17 — Source Data for Figure 7 [file EMBJ-42-e112712-s017.zip › Figure 7/Figure 7B.pdf]

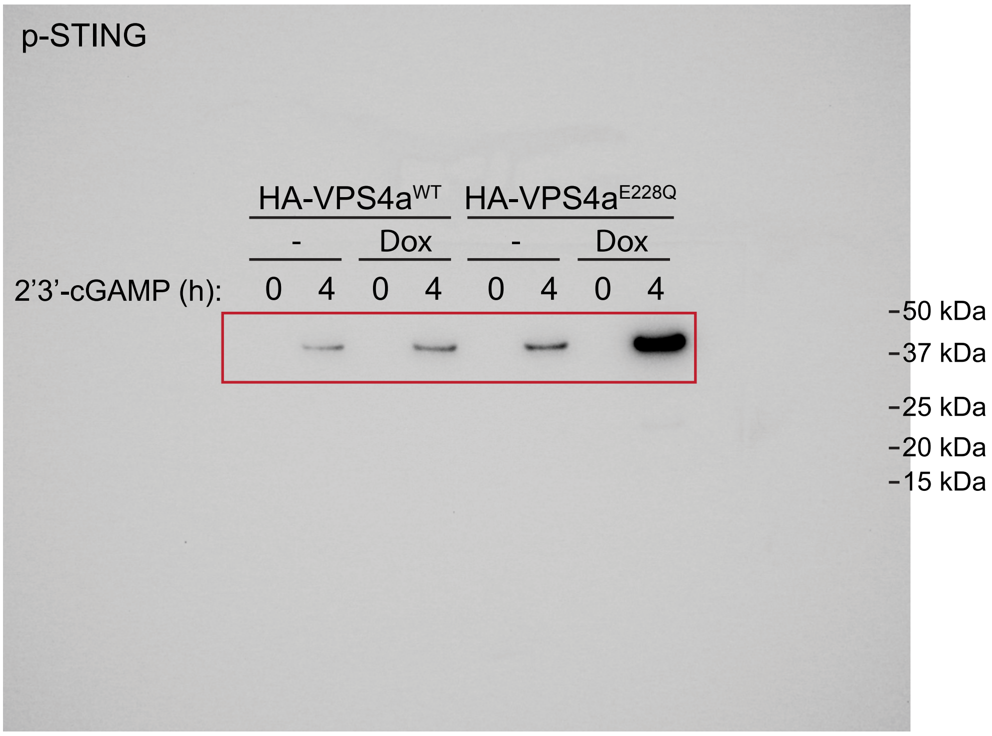
p-STING
HA-VPS4a^WT
HA-VPS4a^E228Q
-
Dox
-
Dox
2'3'-cGAMP (h):
0
4
0
4
0
4
0
4
-50 kDa
-37 kDa
-25 kDa
-20 kDa
-15 kDa

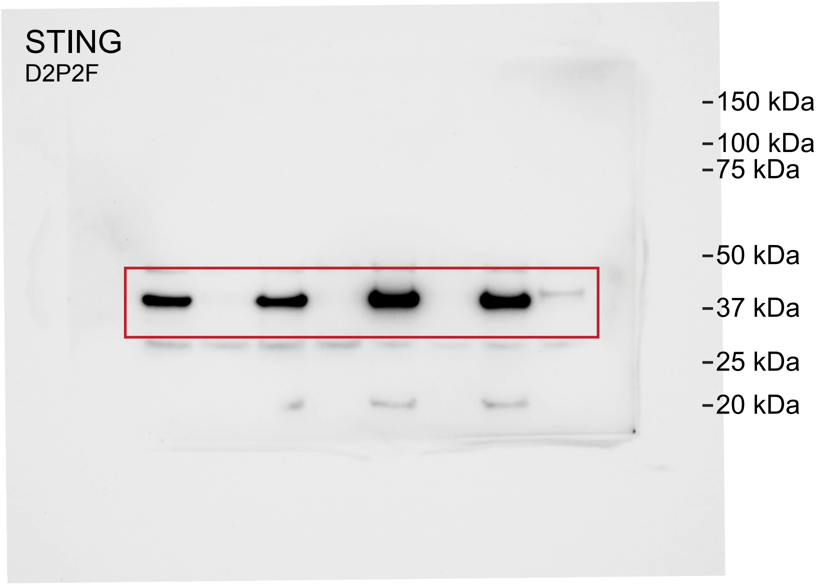
STING
D2P2F
-150 kDa
-100 kDa
-75 kDa
-50 kDa
-37 kDa
-25 kDa
-20 kDa

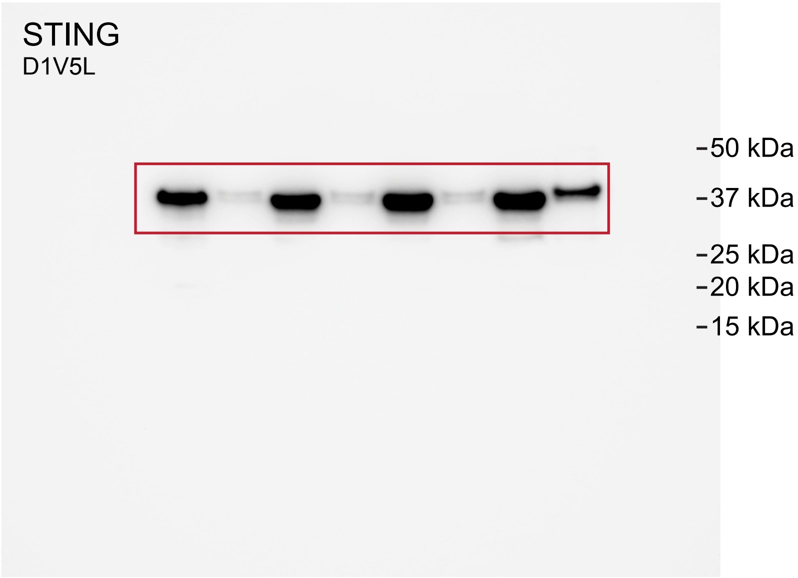
STING
D1V5L
-50 kDa
-37 kDa
-25 kDa
-20 kDa
-15 kDa

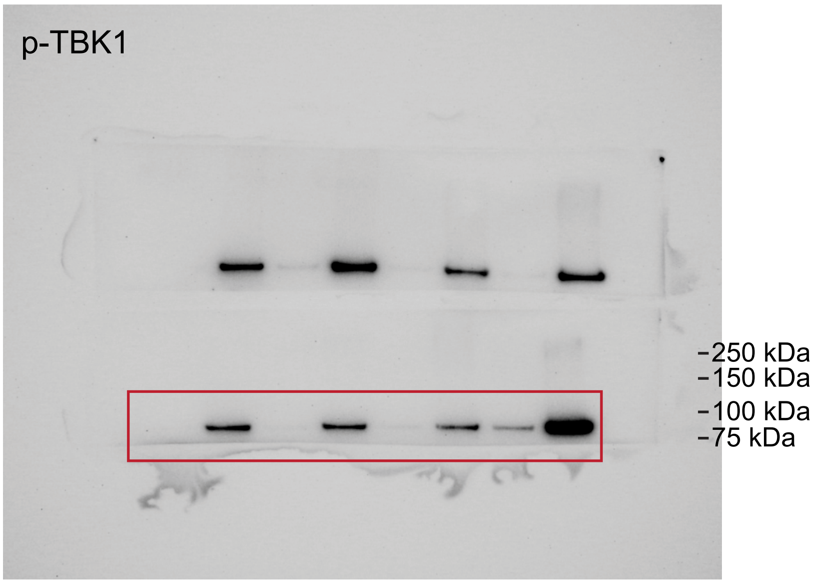
p-TBK1
-250 kDa
-150 kDa
-100 kDa
-75 kDa

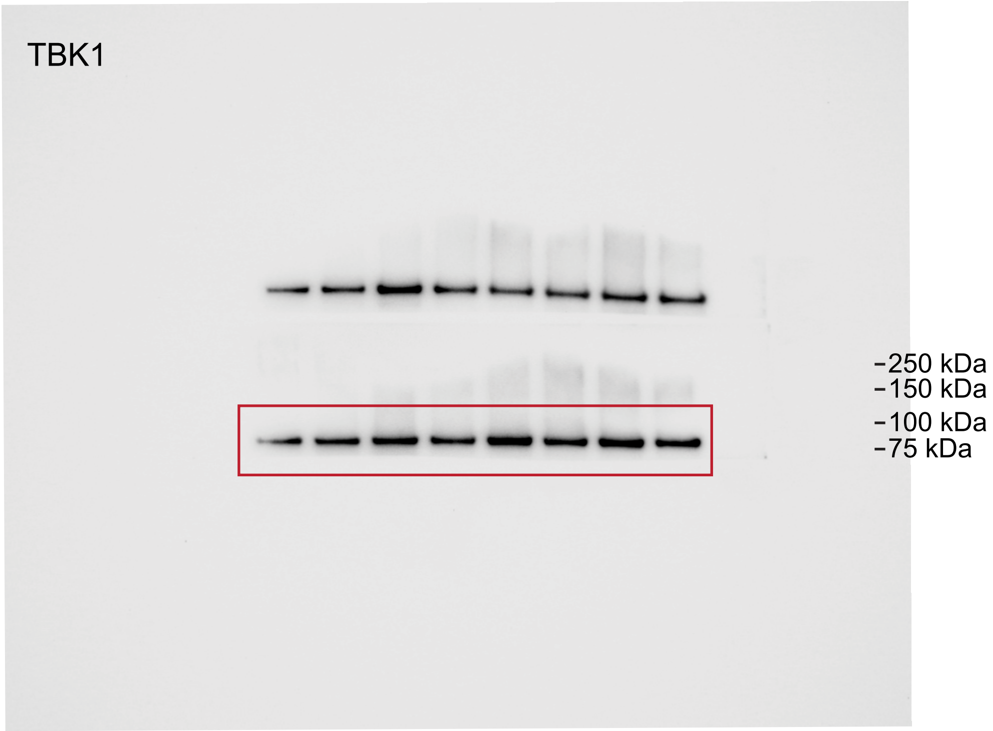
TBK1
-250 kDa
-150 kDa
-100 kDa
-75 kDa

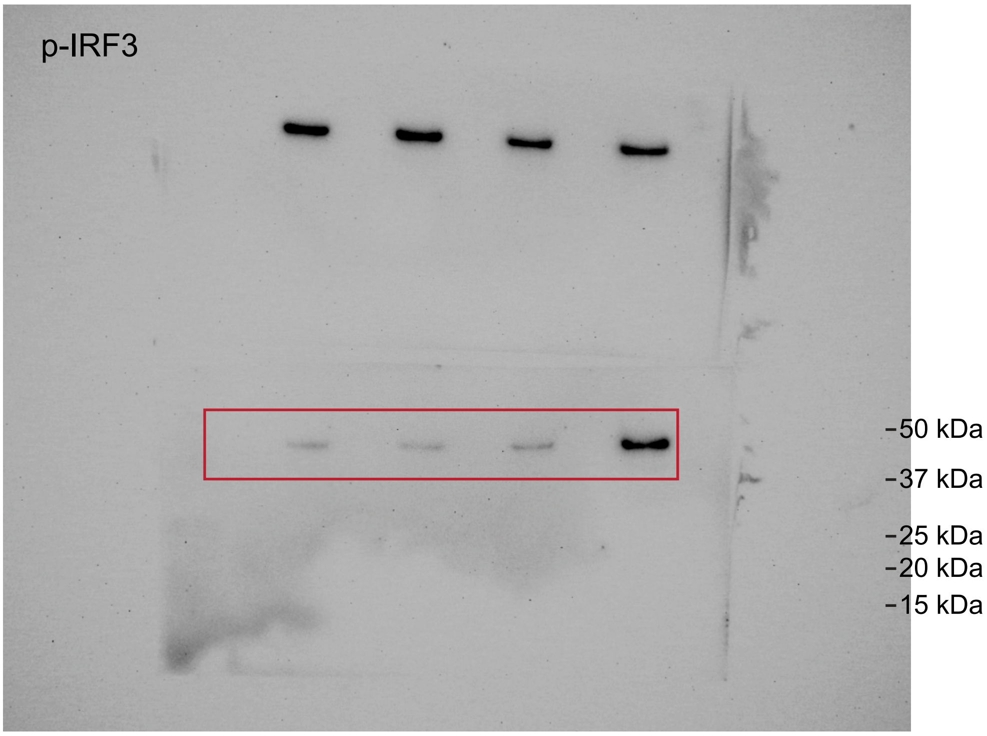
p-IRF3
-50 kDa
-37 kDa
-25 kDa
-20 kDa
-15 kDa

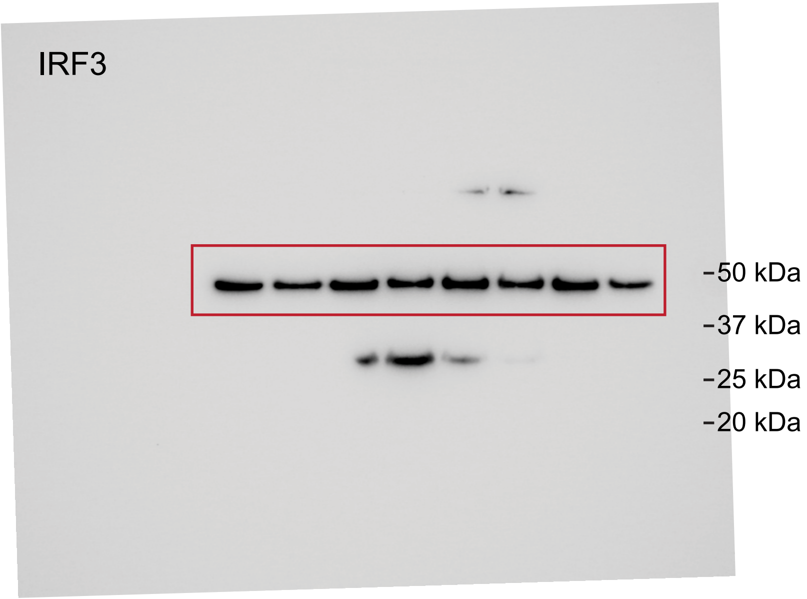
IRF3
-50 kDa
-37 kDa
-25 kDa
-20 kDa

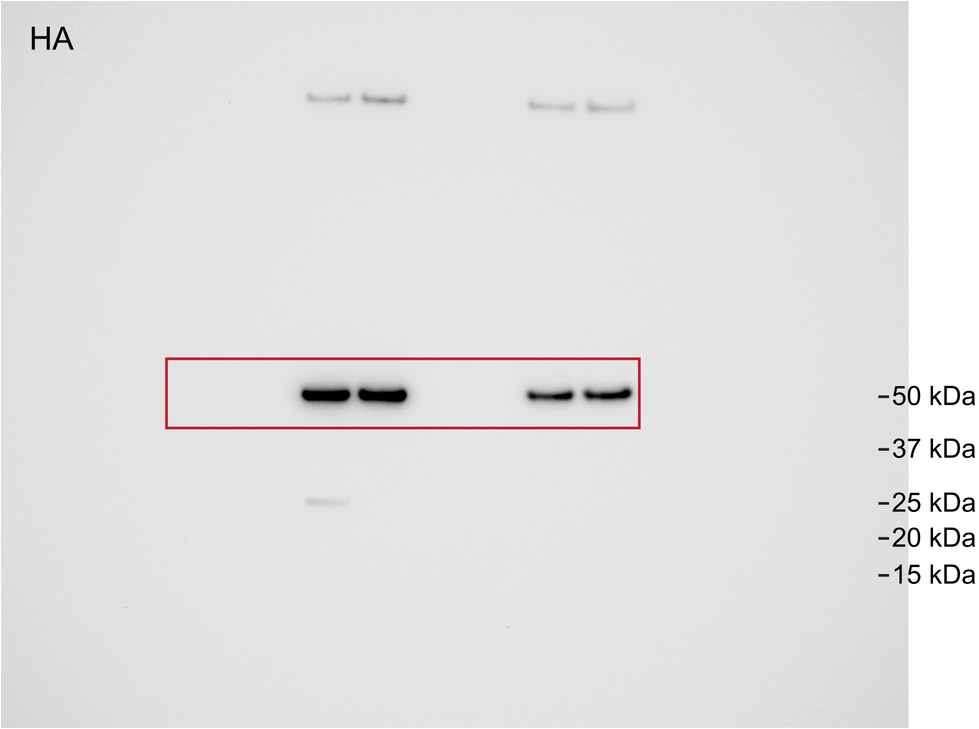
HA
-50 kDa
-37 kDa
-25 kDa
-20 kDa
-15 kDa

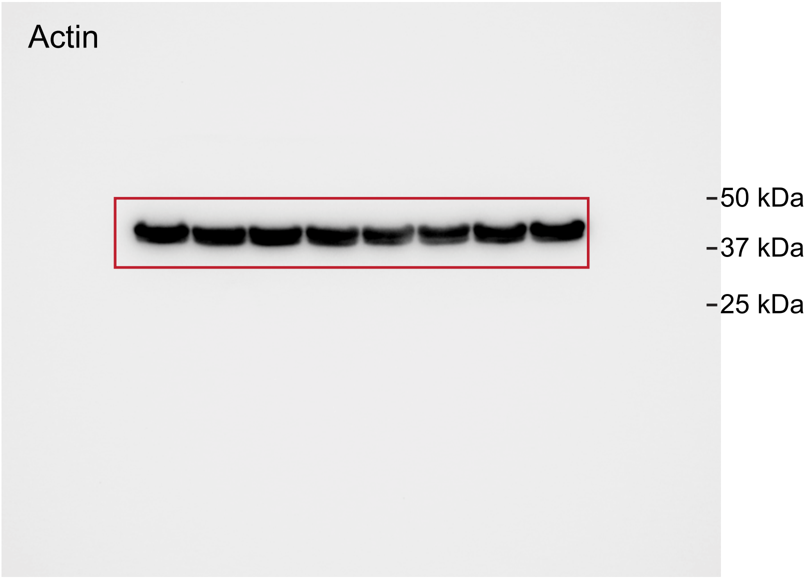
Actin
-50 kDa
-37 kDa
-25 kDa
