# Supplementary material for: Termination of STING responses is mediated via ESCRT‐dependent degradation
Source: EMBO J. 2023 May 4;42(12):e112712. doi: 10.15252/embj.2022112712 (PMC10267698; doi:10.15252/embj.2022112712)
Supplement: Supplementary file 18 [file EMBJ-42-e112712-s016.pdf]

THE EMBO JOURNAL

Termination of STING responses is mediated via ESCRT-dependent degradation

Katherine Balka, Rajan Venkatraman, Tahnee Saunders, Angus Schoppee, Ee Shan Pang, Zoe Magill, Jihane Homman-Ludiye, Cheng Huang, Rachael Lane, Harrison York, Peck Tan, Ralf Schittenhelm, Senthil Arumugam, Benjamin Kile, Meredith O'Keeffe, and Dominic De Nardo
DOI: 10.15252/embj.2022112712

Corresponding author(s): Dominic De Nardo (dominic.denardo@monash.edu) , Katherine Balka (Katherine.Balka@monash.edu)

Review Timeline:

Submission Date:	29th Sep 22
Editorial Decision:	10th Nov 22
Revision Received:	9th Mar 23
Editorial Decision:	11th Apr 23
Revision Received:	14th Apr 23
Accepted:	17th Apr 23

Editor: Karin Dumstrei

Transaction Report:

(Note: With the exception of the correction of typographical or spelling errors that could be a source of ambiguity, letters and reports are not edited. Depending on transfer agreements, referee reports obtained elsewhere may or may not be included in this compilation. Referee reports are anonymous unless the Referee chooses to sign their reports.)

Dear Dom,

Thank you for submitting your manuscript to The EMBO Journal. Your study has now been seen by three referees and their comments are provided below.

As you can see from the comments, the referees find the study interesting and suitable for publication here. They raise a number of constructive points that I would like to ask you to incorporate in a revised version.

It would be good to discuss the needed revisions further and I am available to do so via email or a video call. Let me know what works best for you.

When preparing your letter of response to the referees' comments, please bear in mind that this will form part of the Review Process File, and will therefore be available online to the community. For more details on our Transparent Editorial Process, please visit our website: https://www.embopress.org/page/journal/14602075/authorguide#transparentprocess

I thank you for the opportunity to consider your work for publication. I look forward to your revision.

Yours sincerely,

Karin

Karin Dumstrei, PhD
Senior Editor
The EMBO Journal

Instructions for preparing your revised manuscript:

Please make sure you upload a letter of response to the referees' comments together with the revised manuscript.

Please also check that the title and abstract of the manuscript are brief, yet explicit, even to non-specialists.

When assembling figures, please refer to our figure preparation guideline in order to ensure proper formatting and readability in print as well as on screen:
https://bit.ly/EMBOPressFigurePreparationGuideline
See also guidelines for figure legends: https://www.embopress.org/page/journal/14602075/authorguide#figureformat

At EMBO Press we ask authors to provide source data for the main manuscript figures. Our source data coordinator will contact you to discuss which figure panels we would need source data for and will also provide you with helpful tips on how to upload and organize the files.

IMPORTANT: When you send the revision we will require
- a point-by-point response to the referees' comments, with a detailed description of the changes made (as a word file).
- a word file of the manuscript text.
- individual production quality figure files (one file per figure)
- a complete author checklist, which you can download from our author guidelines (https://www.embopress.org/page/journal/14602075/authorguide).
- Expanded View files (replacing Supplementary Information)
Please see out instructions to authors
https://www.embopress.org/page/journal/14602075/authorguide#expandedview

Please remember: Digital image enhancement is acceptable practice, as long as it accurately represents the original data and conforms to community standards. If a figure has been subjected to significant electronic manipulation, this must be noted in the figure legend or in the 'Materials and Methods' section. The editors reserve the right to request original versions of figures and the original images that were used to assemble the figure.

Guide For Authors: https://www.embopress.org/page/journal/14602075/authorguide

We realize that it is difficult to revise to a specific deadline. In the interest of protecting the conceptual advance provided by the work, we recommend a revision within 3 months (8th Feb 2023). Let me know if you need more time as I can extend the revision time.

As a matter of policy, competing manuscripts published during this period will not negatively impact on our assessment of the conceptual advance presented by your study.

Referee #1:

The ER-resident protein STING is a crucial component of the innate immune response mediated by pattern recognition receptors (PRR). STING is essential to induce the expression of pro-inflammatory cytokines and type I interferons (IFN) via the transcription factors NF-kB and IRF3 upon recognition of viral infection by the DNA-sensing PRR cGAS. STING is localized to the ER in resting cells, and traffics from the ER to the ERGIC and the Golgi upon binding its ligand 2'3'cGAMP, which is produced by cGAS. This relocalisation of STING is crucial to activate the type I IFN response, whereas the NF-kB transcription factor can already be activated from the ER prior trafficking. While the regulation of STING trafficking from the ER to the Golgi has been described in quite some detail, its fate after signaling remains relatively poorly defined. It is known that STING is rapidly degraded after initiation of signaling and relocalises from the Golgi to lysosomes. Further, it was shown that blocking lysosomal acidification prevents STING degradation and prolongs STING-mediated signaling.
However, the precise factors that contribute to Golgi-lysosome trafficking of STING and are therefore crucial to terminate STING signaling have not been described.
The study by Balka et al. performed an unbiased phospho-proteomics screen in primary murine macrophages and compared unstimulated cells with cells being stimulated with the STING agonist DMXAA for 5, 10 and 30 min. They find an enrichment of phosphorylation events of proteins being involved in membrane trafficking, and also identify proteins known to undergo phosphorylation upon STING activation such as iRHOM2, IKKbeta and STING itself. The authors then show live cell imaging data with tagged STING versions and identify a component of the ESCRT complex, HRS, to recognize ubiquitinated STING and interact and colocalize with it. They further show that disrupting components of the ESCRT complex leads to prolonged STING signaling, concluding that the ESCRT complex plays an important role to terminate STING signaling and mediate its degradation.
The finding that the ESCRT complex contributes to terminate STING signaling is very interesting and important for the field, but the presented data needs additional experiments and controls to substantiate this finding.

Major comments:

1. Figure 1: The phosphor-proteomics experiment is a strong dataset and I am wondering whether it would be possible to visualize the time resolution graphically (as a scheme) when focusing on proteins involved in membrane trafficking. Since different time points of stimulation were chosen, it should be possible to follow the kinetics of STING trafficking path by analyzing the intracellular localization of the proteins and the time of their phosphorylation - a graphic similar to Figure 6F, but adding the details of the phospho-proteomics results.
I have one comment on this experiment though: to unequivocally show that the phosphorylation events are attributed only to STING, it would be ideal to perform this experiment in STING KO cells treated with DMXAA - this would control for other pathways DMXXA may possibly activate.
2. Figure 2: The authors use two different versions of fluorescently tagged STING, Ruby-STING and eGFP-STING, and analyse its localization upon different time points post stimulation. First, it would be crucial to show that both of these proteins are signaling competent by expressing them in STING KO cells and measuring type I IFN secretion/transcriptionupon stimulation. Second, in Figure 2E the authors used eGFP-STING and claim that the structures that eGFP-STING localizes to are endosomes. However, since markers for endosomes where not used, this claim cannot be made.
3. Figure 3: What is the purpose of the recovery experiment shown in Figure 3C, which shows STING levels in cells recovering for different lengths of time after DMXXA stimulation? This experiment is not really linked to the major finding of this manuscript, which is the identification of the ESCRT pathway being crucial for terminating STING signaling.
Regarding the second stimulation after 24h of recovery: while STING signaling can be activated a second time, shown by phosphorylation=activation of TBK1 and IRF3 (albeit less efficiently), secretion of type I IFN is completely blunted. In the discussion, the authors speculate that epigenetic changes/transcription regulation may explain this finding. However, another explanation may be that proteins important for type I IFN secretion are altered (regarding their trafficking). To distinguish between these possibilities, the type I IFN response should be analyzed by RT-PCR. Alternatively, since IFNbeta is sometimes difficult to analyze by RT-PCR, transcription of ISGs can be analyzed.
4. Figure 4: This figure nicely shows that STING signaling is prolonged when ubiquitination is inhibited (as measured by detecting phosphorylated STING/TBK1/IRF3). What needs to be added is also a functional readout such as type I IFN transcription or secretion, which should be enhanced in the presence of TAK243.
5. Figure 5: This figure shows that STING is ubiquitinated and interacts and colocalizes with the ESCRT-0 component HRS. What is missing in the STING-HRS Immunoprecipitation is a control with cells not expressing HA-tagged STING to control for unspecific binding of HRS to the beads (Figure 5B). Regarding the colocalization of STING and HRS (Figure 5 C+D): please also show the following overlays: eGFP-STING with HRS, eGFP-STING with Ub, HRS with Ub. I am amazed how clean the IF

with the Ub antibody is - I would have expected a much more diffuse pattern, but it seems that STING and its "surrounding" are the major sites of ubiquitination at these time points?
6. Figure 5A suggests that STING is ubiquitinated already in unstimulated conditions. Is this ER localized STING or Golgi-localized STING (since STING cycles also in unstimulated cells)? To unequivocally show that it is ER resident STING, the STING K288R mutant could be included in this assay. Further, I am puzzled by this sentence, line 296: "We observed an enrichment in STING and the appearance of ubiquitin smears, suggesting that STING is directly ubiquitinated following activation (Fig 5A)." However, STING is already ubiquitinated in unstimulated cells (upper panel lane 1 in Figure 5A) and I do not agree that ubiquitin smears appear with activation since they are already present in lane 1 (0 h of DMXXA).
7. Figure 6 A+B analyses STING signaling in HRS knockdown cells and provides some evidence that STING signaling may be prolonged in the absence of HRS, and IL6 secretion is enhanced. The issue with this figure is that the knockdown is not very efficient. I see the efforts made by the authors by trying to knockdown HRS with three independent guide RNAs. However, due to the weak knockdown the data is not very convincing - would it make sense to design another set of guide RNAs that target the first exons of HRS instead of the promoter? What should be added is, to underpin the IL6 data, a type I IFN readout (ELISA or RT-PCR).
The data with the dominant negative VPS4A E228Q mutant (Figure 6 C+D) is more convincing than the HRS data, and also includes functional readouts with IL6 and IFNbeta.

What could strengthen the finding that ubiquitinated STING is recognized by HRS would be to inhibit STING ubiquitination which should result in a loss of STING-HRS interaction. Ideally, the site that is ubiquitinated in STING would be identified and mutated (although this may be difficult since STING may then be ubiquitinated at another site). However, an easier way could be to repeat the IP in cells treated with TAK243. A nice control would also be the inclusion of the K288R mutant - HRS should not interact with this ER resident mutant, and it would be interesting to see if this mutant can get ubiquitinated.

Minor comments:
1. This study focuses on the regulation of Golgi to Endo/Lysosome trafficking of murine STING. Since differences between human and murine STING exist, it should be clarified already in the abstract that this work was done with murine STING in macrophages derived from mice.
2. Figure 6F (scheme): STING is known to activate NF-kB also from the ER, prior trafficking (Stempel et al., 2019). While the authors show this in their 2020 review (Balka and De Nardo, FEBS Journal), the according arrow is missing in this scheme.
3. Line 69-71: Would be nice to elaborate a bit for the non-STING expert reader about the non-IFN responses mediated by STING (one sentence may already suffice).
4. Line 259: Does termination of STING signaling necessarily/exclusively require internalization into larger structures? Signaling may also be terminated due to conformational changes/changes in binding partners.

Referee #2:

In the study, the authors identify ESCRT-mediated degradation of STING as a crucial checkpoint to terminate innate immune signaling. The study is well-written and clear, and the data is of high quality. The finding is timely and addresses an important question. I have several comments:

1. STING is activated with DMXAA in the study, and diAbzi in some experiments. These are artificial STING agonists, and we know that STING does not adopt the same conformation when it is activated with the natural agonist 2'3'-cGAMP (PMID 23910378). It is important to establish the key finding of the study with 2'3'-cGAMP. Is STING degradation induced by 2'3'-cGAMP, and its negative effect on signaling & cytokine production, dependent on the ESCRT machinery?
2. The refractory response to secondary stimulation appears to correlate with the restored level of STING (Fig 3C, 3E). Can secondary STING stimulation be rescued by STING over-expression after the 1st stimulation?
3. They clearly show that inhibiting STING degradation using TAK243 or VPS4a-E228Q enhances the response to DMXAA, but surprisingly, this does not lead to constitutive STING signaling. This is rather contradictory with the current "prime-boost" model of STING signaling (PMID 34290407) (l.417-419, equilibrium and STING turnover). This is also seemingly in contradiction with COPA mutations that lead to constitutive STING trafficking and activation in the absence of agonist increase. This may suggest that the ESCRT pathway of STING degradation is selectively wired to degrade STING activated by the ligand, and not resting STING even if the latter trafficks to the Golgi at baseline in the absence of ligand. However, the degradation of activated STING appears to be independent of TBK1 and IKKe. This is a weak point in the study that should be discussed and addressed with experiments:
a. Is STING trafficking to the Golgi after DMXXA treatment required for ubiquitination (as in Fig. 5B)? This can be tested using BFA.
b. Is STING with a SAVI mutation (V154M or N153S in mice) ubiquitinated and sensitive to ESCRT-mediated degradation?
c. Is STING itself ubiquitinated, or is it an indirect ubiquitination of other proteins? This is not clear from Fig 5B, the anti-STING HA in the pull-down does not show a smear, while the anti-Ub does. Evidently, it is difficult to introduce large deletions in STING without impacting ligand binding or trafficking. However, the authors should be able to test the role of lysine residues with point mutations, and also to test the role of key residues in the CTT (such as the serine/threonine residues identified in Figure EV2).
d. During nuclear envelope rupture, as in many other scenarios, the ESCRT machinery is recruited to close membrane holes or

damages. Can the authors envisage a scenario in which activated STING would lead to membrane damage at the Golgi, resulting in ESCRT recruitment?
e. Contrary to claims in the text (l.46, l.121, l.290, l.300, l.317-318, l.1087), they do not show that STING is ubiquitinated. They can however detect ubiquitinated proteins in a STING IP (with a major protein at ~52 kDa, Fig 5B). They should carefully revise the text.
4. The identification of an essential factor of the ESCRT machinery using convincing genetic depletion is crucial. The current data with HRS is encouraging but not convincing due to the low level of depletion. I understand that HRS is essential for viability and that they cannot make full KO cells. However, their inducible Crispr/Cas9 silencing system is just not working well. They can probably obtain much better results with siRNAs or inducible shRNA lentivectors. They should also test the specificity of HRS for STING signaling using control ligands (e.g. RIG-I/MDA5).
5. The analysis of phosphorylation of STING and cellular proteins shown in Fig 1 is impressive but only serves to report that many trafficking proteins are detected. This data seems under-exploited and is superfluous to rationalize an analysis of STING trafficking. Can the authors make any link with the ESCRT machinery specifically? Are there phospho-proteins of interest that can be tested? And what is the role of phosphorylated residues in STING in its degradation? If they cannot better link this dataset, the authors should consider removing it.

Other comments:
6. Abstract l.51: "a" rather than "the" mechanisms
7. L.147: Avoid claims of novelty
8. L.199 Fig 2E indicates "endosomes" but these are rather undefined "vesicles"?
9. L.219-221, l.233-234: Masking of STING detection by monoclonals due to PTMs can be easily addressed using polyclonal antibodies, this should be tested instead of leaving hanging such speculation. Furthermore, the two antibodies in Fig 3C show the same pattern, there are just different intensities through all time points (even without ligand at t=0). Therefore, the differences are not related to PTM changes during activation, but rather to differences in sensitivity between the two antibodies and the ECL assay. Again, this should be addressed with a polyclonal antibody.
10. Statistics: a t-test cannot be used if the number of groups shown on the graph is > 2. They need to use an ANOVA instead (Fig 3F, 3G).
11. Claims for Fig 4D/E are not supported by statistical analysis, this needs to be performed. Consider normalizing the data with a log transformation.

Referee #3:

Conducting a phosphoproteomic screen following STING activation, the authors identify various proteins associated with endosomal and lysosomal function to be phosphorylated. Following up on STING trafficking using live cell microscopy, the authors first corroborate previous work on STING translocation to the Golgi apparatus. Moreover, they find that STING levels decrease upon stimulation and that previously stimulated cells displayed a blunted STING response upon re-stimulation. This phenomenon was partly related to reduced STING levels, which were slow to recover, but also to additional, unspecified mechanisms that led to refractory STING signaling. Focusing on how STING is degraded in the lysosome, the authors then turn their attention to the ESCRT machinery. Since ubiquitination precedes ESCRT dependent cargo degradation, the authors test whether blocking ubiquitination impacts on the abundance of STING following stimulation. Here, they find that blocking ubiquitination not only allows STING phosphorylation and subsequent IRF3 phosphorylation to proceed normally (in fact these two hallmarks of STING activation are indeed enhanced), but that it also rescues STING from degradation. These results indirectly imply the ESCRT pathway in STING degradation. To corroborate this notion, they go on to block the ESCRT-0 protein Hrs and they also overexpress a dominant negative version of the ESCRT-component VPS4a. Both of these perturbations lead to increased STING levels and increased activity of the STING signaling cascade, confirming the original hypothesis.

All in all, this is an interesting manuscript that describes a novel aspect of STING biology, namely how STING is degraded via an ESCRT-dependent mechanism. All in all, the experiments are performed at a high level and the claims are well supported by the data. A particularly interesting side aspect of these data is that the inhibition of ubiquitination has no negative impact on proximal events of STING activation. This sets aside several studies in the field that have proposed sophisticated models for STING ubiquitination upstream of TBK1 recruitment or activation. Some parts of the manuscript, on the other hand, are less developed. E.g. it is unclear how STING stimulation results in this pronounced tachyphylaxis as reported in Fig. 3F/G. This is clearly not due to reduced STING levels and therefore diverts the narrative of the manuscript in another direction that has not yet been explored.

Nevertheless, the manuscript is already quite mature and its results certainly warrant publication without additional mechanistic or functional studies.

***Balka et al.,* (Manuscript EMBOJ-2022-112712R) Point-by Point response to reviewers.**

Reviewer comments are shown in *italics* and our responses are shown in blue.

Referee #1

The ER-resident protein STING is a crucial component of the innate immune response mediated by pattern recognition receptors (PRR). STING is essential to induce the expression of pro-inflammatory cytokines and type I interferons (IFN) via the transcription factors NF-kB and IRF3 upon recognition of viral infection by the DNA-sensing PRR cGAS. STING is localized to the ER in resting cells, and traffics from the ER to the ERGIC and the Golgi upon binding its ligand 2'3'cGAMP, which is produced by cGAS. This relocalisation of STING is crucial to activate the type I IFN response, whereas the NF-kB transcription factor can already be activated from the ER prior trafficking. While the regulation of STING trafficking from the ER to the Golgi has been described in quite some detail, its fate after signaling remains relatively poorly defined. It is known that STING is rapidly degraded after initiation of signaling and relocalises from the Golgi to lysosomes. Further, it was shown that blocking lysosomal acidification prevents STING degradation and prolongs STING-mediated signaling.
However, the precise factors that contribute to Golgi-lysosome trafficking of STING and are therefore crucial to terminate STING signaling have not been described.

The study by Balka et al. performed an unbiased phospho-proteomics screen in primary murine macrophages and compared unstimulated cells with cells being stimulated with the STING agonist DMXAA for 5, 10 and 30 min. They find an enrichment of phosphorylation events of proteins being involved in membrane trafficking, and also identify proteins known to undergo phosphorylation upon STING activation such as iRHOM2, IKKbeta and STING itself. The authors then show live cell imaging data with tagged STING versions and identify a component of the ESCRT complex, HRS, to recognize ubiquitinated STING and interact and colocalize with it. They further show that disrupting components of the ESCRT complex leads to prolonged STING signaling, concluding that the ESCRT complex plays an important role to terminate STING signaling and mediate its degradation.
The finding that the ESCRT complex contributes to terminate STING signaling is very interesting and important for the field, but the presented data needs additional experiments and controls to substantiate this finding.

Major comments:
1. Figure 1: The phosphor-proteomics experiment is a strong dataset and I am wondering whether it would be possible to visualize the time resolution graphically (as a scheme) when focusing on proteins involved in membrane trafficking. Since different time points of stimulation were chosen, it should be possible to follow the kinetics of STING trafficking path by analyzing the intracellular localization of the proteins and the time of their phosphorylation - a graphic similar to Figure 6F, but adding the details of the phospho-proteomics results.

We thank the reviewer for their interesting suggestion. We utilised the STRING online database (https://string-db.org) to unbiasedly assign detected phosphorylated/dephosphorylated proteins into different "cellular components" according to gene ontology analysis. We have generated a new schematic of STING trafficking depicting these proteins presented as **Fig EV2G**.

I have one comment on this experiment though: to unequivocally show that the phosphorylation events are attributed only to STING, it would be ideal to perform this experiment in STING KO cells treated with DMXAA - this would control for other pathways DMXXA may possibly activate.

We appreciate the reviewer's suggestion and do agree that in hindsight utilising *Sting*$^{-/-}$ BMDMs would have provided a better negative control. However, as the reviewer notes this

is already a "strong dataset" (e.g. detection of STING phosphorylation events and known proteins involved in the STING pathway, as well as several novel trafficking proteins) we do not believe it is necessary to repeat the screen with *Sting*$^{-/-}$ controls.

2. Figure 2: The authors use two different versions of fluorescently tagged STING, Ruby-STING and eGFP-STING, and analyse its localization upon different time points post stimulation.
First, it would be crucial to show that both of these proteins are signaling competent by expressing them in STING KO cells and measuring type I IFN secretion/transcription upon stimulation.

We thank the reviewer for this suggestion. We now provide signalling data showing that cells expressing eGFP-STING and mRuby3-STING are responsive to STING activation. These new data are presented as **Fig EV3A**.

Second, in Figure 2E the authors used eGFP-STING and claim that the structures that eGFP-STING localizes to are endosomes. However, since markers for endosomes where not used, this claim cannot be made.

We thank the reviewer for this astute observation. We concede that, although STING vesicles have been previously characterised as endosomes by others (e.g., PMID: 29241549), we did not do that in our study. We have therefore amended the text and figure to remove specific reference to endosomes.

3. Figure 3: What is the purpose of the recovery experiment shown in Figure 3C, which shows STING levels in cells recovering for different lengths of time after DMXXA stimulation? This experiment is not really linked to the major finding of this manuscript, which is the identification of the ESCRT pathway being crucial for terminating STING signaling. Regarding the second stimulation after 24h of recovery: while STING signaling can be activated a second time, shown by phosphorylation=activation of TBK1 and IRF3 (albeit less efficiently), secretion of type I IFN is completely blunted. In the discussion, the authors speculate that epigenetic changes/transcription regulation may explain this finding. However, another explanation may be that proteins important for type I IFN secretion are altered (regarding their trafficking). To distinguish between these possibilities, the type I IFN response should be analyzed by RT-PCR. Alternatively, since IFNbeta is sometimes difficult to analyze by RT-PCR, transcription of ISGs can be analyzed.

We thank the reviewer for their helpful comment and agree that the recovery data presented in Figure 3 of the original study requires much more work and is ultimately distracting to the narrative of the rest of the study. This notion was shared by all three reviewers. As such we have decided to remove the recovery and re-stimulation data from the manuscript.

4. Figure 4: This figure nicely shows that STING signaling is prolonged when ubiquitination is inhibited (as measured by detecting phosphorylated STING/TBK1/IRF3). What needs to be added is also a functional readout such as type I IFN transcription or secretion, which should be enhanced in the presence of TAK243.

To address this point we have performed qPCR experiments to assess *Ifnb1*, *Isg15* and *Il6* gene expression following DMXAA and 2'3'-cGAM(PS)2 stimulation in the absence or presence of TAK243. This new data, provided as **Fig 4G-I,** demonstrates that TAK243 increased expression of all three genes following STING activation. In addition, we also provide new western blot data (similar to **Fig 4B** with DMXAA) demonstrating the same effect of TAK243 on STING signalling (i.e., increased p-STING, p-TBK1, p-IRF3) in response to another STING ligand – 2'3'-cGAM(PS)2 (presented as new **Fig 4C**).

5. Figure 5: This figure shows that STING is ubiquitinated and interacts and colocalizes with the ESCRT-0 component HRS. What is missing in the STING-HRS Immunoprecipitation is a control with cells not expressing HA-tagged STING to control for unspecific binding of HRS to the beads (Figure 5B).

We thank the reviewer for suggesting this important control experiment. We repeated the HA immunoprecipitation (IP) experiments including the *Sting*$^{-/-}$ controls. This new data, shown in **Fig EV4B,** demonstrates that while we observe low level non-specific binding of HRS to the magnetic beads used for IP in *Sting*$^{-/-}$ macrophages, we still see a clear enrichment of HRS binding in HA-STING cells in response to activation.

Regarding the colocalization of STING and HRS (Figure 5 C+D): please also show the following overlays: eGFP-STING with HRS, eGFP-STING with Ub, HRS with Ub. I am amazed how clean the IF with the Ub antibody is - I would have expected a much more diffuse pattern, but it seems that STING and its "surrounding" are the major sites of ubiquitination at these time points?

We thank the reviewer for this suggestion and have now added the requested pairwise overlays in **Fig EV4D**. We were also surprised how clear the staining for conjugated ubiquitin was, however was consistent with previous studies (e.g., PMIDs: 30482832, 32651614, 31951200). In our study, as the correlation of ubiquitin intensity with STING localisation is strong, the images have been thresholded to avoid oversaturated pixels. Furthermore, these images were captured using a confocal microscope equipped with Airyscan detector, providing improved resolution and greatly enhanced signal to noise over standard confocal microscopy, which could have further contributed to improve the clarity of ubiquitin intensity nearby STING (reducing background/basal diffuse ubiquitin signal).

6. Figure 5A suggests that STING is ubiquitinated already in unstimulated conditions. Is this ER localized STING or Golgi-localized STING (since STING cycles also in unstimulated cells)? To unequivocally show that it is ER resident STING, the STING K288R mutant could be included in this assay. Further, I am puzzled by this sentence, line 296: "We observed an enrichment in STING and the appearance of ubiquitin smears, suggesting that STING is directly ubiquitinated following activation (Fig 5A)." However, STING is already ubiquitinated in unstimulated cells (upper panel lane 1 in Figure 5A) and I do not agree that ubiquitin smears appear with activation since they are already present in lane 1 (0 h of DMXXA).

We apologise for the confusion regarding the data displayed in **Fig 5A** regarding the ubiquitination of STING. To clarify, in **Fig 5A** we have performed a Tandem Ubiquitin Binding Entities (TUBE) assay, which isolates all poly-ubiquitinated proteins from total cell lysates – hence, the appearance of STING strongly suggests its ubiquitination. In the STING panel from the TUBE assay (**Fig 5A**, upper panel), we can observe STING at its native molecular weight (i.e. ~42kDa) in untreated conditions, which following activation is greatly enriched. Upon activation we further observe the appearance of a distinctive ubiquitin smearing pattern when probing with the STING antibody. These higher molecular weight versions of STING are due to covalent modification of STING with polyubiquitin chains – further evidence STING is directly ubiquitinated. We have improved our explanation of this data in the text (lines 289-297) and annotated the ubiquitinated STING smearing pattern (i.e., “Ub-STING”) on **Fig 5A** for clarity. In comparison to probing for STING (**Fig 5A**, upper panel of TUBE), using an anti-ubiquitin antibody (**Fig 5A**, lower panel of TUBE) we can observe ubiquitin smearing across all three lanes – i.e. we have isolated comparable levels of ubiquitinated proteins in each sample.

7. Figure 6 A+B analyses STING signaling in HRS knockdown cells and provides some evidence that STING signaling may be prolonged in the absence of HRS, and IL6 secretion is

enhanced. The issue with this figure is that the knockdown is not very efficient. I see the efforts made by the authors by trying to knockdown HRS with three independent guide RNAs. However, due to the weak knockdown the data is not very convincing - would it make sense to design another set of guide RNAs that target the first exons of HRS instead of the promoter? What should be added is, to underpin the IL6 data, a type I IFN readout (ELISA or RT-PCR). The data with the dominant negative VPS4A E228Q mutant (Figure 6 C+D) is more convincing than the HRS data, and also includes functional readouts with IL6 and IFNbeta.

We appreciate the reviewer's comment and also agree that the knockdown of HRS shown in Figure 6 of the original manuscript was minor. We re-selected the cells for dCas9 and the HRS-targeting sgRNAs and found that 48 h doxycycline treatment (rather than the original overnight i.e., 18 h) produced a slightly improved HRS knockdown (without impeding cell viability). We also included 2'3'-cGAM(PS)2 in our repeat experiments (**Fig 6A-C**). Utilising this knockdown approach, we can observe a more appreciable increase in signalling responses and cytokine production (**Fig 6A-C**). Interestingly, during the undertaking of our revision experiments, a complementary manuscript (PMID: 36739287) has very recently been published (February 2023) from an independent group. Gentili et al., similarly knocked down HRS in human fibroblasts using CRISPR. Although they similarly induced a mild knockdown of HRS protein, they also observed the characteristic increase in STING signalling and cytokine expression. These observations strongly support our presented findings.

What could strengthen the finding that ubiquitinated STING is recognized by HRS would be to inhibit STING ubiquitination which should result in a loss of STING-HRS interaction. Ideally, the site that is ubiquitinated in STING would be identified and mutated (although this may be difficult since STING may then be ubiquitinated at another site). However, an easier way could be to repeat the IP in cells treated with TAK243. A nice control would also be the inclusion of the K288R mutant - HRS should not interact with this ER resident mutant, and it would be interesting to see if this mutant can get ubiquitinated.

This is an important point and as suggested by the reviewer, to address this we have examined STING-HRS interactions in the presence of TAK243. We performed immunoprecipitation (IP) of endogenous STING in primary BMDMs and confirmed that the observed interaction with HRS appears to be lost following pre-treatment with TAK243 (**Fig EV4C**). The inclusion of the K288R mutant as a control was not possible in primary BMDMs and although interesting is not required to address the specific point in question.

Minor comments:

1. This study focuses on the regulation of Golgi to Endo/Lysosome trafficking of murine STING. Since differences between human and murine STING exist, it should be clarified already in the abstract that this work was done with murine STING in macrophages derived from mice.

We have stated in the abstract that this work was performed in murine macrophages (line 43). Interestingly, the recently published manuscript (PMID: 36739287) made very similar findings in human cells, thus complementing our findings in mouse macrophages. This supports both the validity and timely nature of our study.

2. Figure 6F (scheme): STING is known to activate NF-kB also from the ER, prior trafficking (Stempel et al., 2019). While the authors show this in their 2020 review (Balka and De Nardo, FEBS Journal), the according arrow is missing in this scheme.

The schematic presented as **Fig 7E** in the revision manuscript is meant to summarise the current findings of this manuscript, not more general findings in the field (e.g., as in our previous review article). As we did not examine STING-induced activation of NF-kB from the ER here we don't feel the addition of this arrow is necessary.

3. Line 69-71: Would be nice to elaborate a bit for the non-STING expert reader about the non-IFN responses mediated by STING (one sentence may already suffice).

We thank the reviewer for this suggestion and have included more information regarding STING non-IFN responses on lines 61-66 of the revised manuscript.

4. Line 259: Does termination of STING signaling necessarily/exclusively require internalization into larger structures? Signaling may also be terminated due to conformational changes/changes in binding partners.

We thank the reviewer for raising this important point and agree that there may be other mechanisms that are also involved in the termination of STING signalling. We have therefore elaborated on this point in the discussion, lines 403-405.

Referee #2:

In the study, the authors identify ESCRT-mediated degradation of STING as a crucial checkpoint to terminate innate immune signaling. The study is well-written and clear, and the data is of high quality. The finding is timely and addresses an important question. I have several comments:

1. STING is activated with DMXAA in the study, and diAbzi in some experiments. These are artificial STING agonists, and we know that STING does not adopt the same conformation when it is activated with the natural agonist 2'3'-cGAMP (PMID 23910378). It is important to establish the key finding of the study with 2'3'-cGAMP. Is STING degradation induced by 2'3'-cGAMP, and its negative effect on signaling & cytokine production, dependent on the ESCRT machinery?

We thank the reviewer for this important suggestion. To address this, we have performed several new experiments utilising 2’3’-cGAM(PS)2 (a stabilised form of 2’3’-cGAMP not requiring transfection). We have now shown that STING is degraded in primary BMDMs following 2’3’-cGAM(PS)2 stimulation (**Fig 3B,D**). We further show that TAK243 blocks 2’3’-cGAM(PS)2-mediated STING degradation (**Fig 4C**) and causes increased *Ifnb1* and ISG expression in response to 2’3’-cGAM(PS)2 (**Fig 4G-I**). Finally, we performed new experiments using 2’3’-cGAM(PS)2 to activate STING in our two models of ESCRT perturbation – HRS CRISPRi knockdown and VPS4-DN overexpression (**Fig 6A-C** and **Fig 7B**, **Fig EV5E** respectively). The results of these new experiments paralleled our previous data utilising DMXAA.

2. The refractory response to secondary stimulation appears to correlate with the restored level of STING (Fig 3C, 3E). Can secondary STING stimulation be rescued by STING over-expression after the 1st stimulation?

As discussed in response to **reviewer 1–point 3**, we have taken on board comments from all three reviewers and decided to remove the restimulation and recovery experiments from this manuscript.

3. They clearly show that inhibiting STING degradation using TAK243 or VPS4a-E228Q enhances the response to DMXAA, but surprisingly, this does not lead to constitutive STING signaling. This is rather contradictory with the current "prime-boost" model of STING signaling (PMID 34290407) (l.417-419, equilibrium and STING turnover). This is also seemingly in contradiction with COPA mutations that lead to constitutive STING trafficking and activation in the absence of agonist increase. This may suggest that the ESCRT pathway of STING

degradation is selectively wired to degrade STING activated by the ligand, and not resting STING even if the latter trafficks to the Golgi at baseline in the absence of ligand.

We thank the reviewer for their thoughtful observation. We were not able to detect constitutive signalling or cytokine production in our models. This may in part be due to the acute nature our experiments. In order to maintain cell viability, we only utilised short-term treatment (i.e., 4 h) with TAK243 or VPS4a-E228Q expression. To explore whether ESCRT perturbation could drive constitutive STING signalling in the longer-term we therefore performed new experiments utilising qPCR after 48 h knockdown of ESCRT-0 HRS. This longer HRS knockdown resulted in increased basal *Ifnb1* and ISG expression, which was blocked by pre-treatment with the STING inhibitor, H-151 (**Fig 6D-F**). Hence, our data suggests that HRS knockdown does impact basal STING activity.

However, the degradation of activated STING appears to be independent of TBK1 and IKKe. This is a weak point in the study that should be discussed and addressed with experiments:
a. Is STING trafficking to the Golgi after DMXXA treatment required for ubiquitination (as in Fig. 5B)? This can be tested using BFA.

We thank the reviewer for this suggestion. From our imaging data presented in **Appendix Fig S3** in which eGFP-STING expressing macrophages were activated and stained for conjugated ubiquitin, we observe the strongest ubiquitin intensity and colocalisation of STING and ubiquitin on STING positive vesicles. This is suggestive that STING becomes ubiquitinated post-Golgi. There is supporting evidence for this notion in a number of published studies:
1) Ni et al., previously found that ubiquitin smearing in a STING pull down was blocked with BFA treatment (inhibiting ER-Golgi transport), suggested most ubiquitination events following STING activation occur following ER-Golgi trafficking (PMID: 28763789).
2*) Gentili et al.,* show that use of H-151 which blocks STING clustering at the Golgi also prevents STING ubiquitination (PMID: 36739287).
3) Kuchitsu et al*.,* (BioRvix pre-print, doi: https://doi.org/10.1101/2022.03.20.485012) utilised a temperature assay, whereby trafficking from the Golgi is prevented at 20ºC, and observe that STING ubiquitination was blocked.
We therefore believe there is already sufficient data to suggest exit from the ER is a pre-requisite for STING ubiquitination.

b. Is STING with a SAVI mutation (V154M or N153S in mice) ubiquitinated and sensitive to ESCRT-mediated degradation?

We thank the reviewer for this excellent suggestion. We have found that stable expression of SAVI STING is difficult to maintain in murine macrophage cell lines due to excessive activation and cytokine production leading to cell death. Therefore, we established a doxycycline-inducible system to look at STING SAVI responses. However, as our models of ESCRT disruption also require doxycycline treatment, the combination of the two approaches was not feasible. However, we were able to show that hSTING N154S exhibits lower expression compared to WT hSTING when expressed in iBMDMs, potentially due to increased degradation (**Fig 4J**). Similar to the data presented in **Fig 4**, TAK243 treatment led to increased activation (i.e. p-STING) of hSTING N154S, suggesting like WT STING, SAVI STING also requires ubiquitination for degradation. This new data is presented as **Fig 4J**. In addition, in the recent publication by Gentili et al., (PMID**:** 36739287), which also shows ESCRT functions to degrade STING, the authors performed co-immunoprecipitation studies in HEK293T cells, demonstrating that STING V155M and R284S (i.e., SAVI forms) interacted with HRS and the ESCRT protein VPS37A, further supporting a role for ESCRT in the degradation of SAVI STING.

c. Is STING itself ubiquitinated, or is it an indirect ubiquitination of other proteins? This is not clear from Fig 5B, the anti-STING HA in the pull-down does not show a smear, while the anti-

Ub does. Evidently, it is difficult to introduce large deletions in STING without impacting ligand binding or trafficking. However, the authors should be able to test the role of lysine residues with point mutations, and also to test the role of key residues in the CTT (such as the serine/threonine residues identified in Figure EV2).

We apologise to the reviewer regarding the explanation of STING ubiquitination. We agree that it is not possible to discern that STING itself is ubiquitinated in **Fig 5B** (i.e., pull down of HA-tagged STING). The ubiquitin smearing can only suggest that either STING or proteins in complex with STING are ubiquitinated. However, as we observe STING smearing within the TUBE assay in **Fig 5A**, this strongly suggests STING is ubiquitinated. Please also see our response to **reviewer 1–point 6**. We have improved the explanation of this data in lines 289-297 of the main text of the revised version of the manuscript to make this point clearer.

In regards to the precise lysine or phosphorylated residues of STING which mediate degradation, we believe this is beyond the scope of this manuscript. Of note, the lysine residues regulating STING degradation were interrogated in the recently published study from Gentili et al., (PMID**:** 36739287). Here the authors found that actually mutation of multiple (i.e., at least 5) lysine residues was required to prevent STING degradation and impede HRS interaction. This supports the suggestion by **reviewer 1 (point 7)** that there appears to be redundancy and complexity for the ubiquitination of STING, in the context of its degradation.

d. During nuclear envelope rupture, as in many other scenarios, the ESCRT machinery is recruited to close membrane holes or damages. Can the authors envisage a scenario in which activated STING would lead to membrane damage at the Golgi, resulting in ESCRT recruitment?

This is a very interesting question raised by the reviewer. To our knowledge ESCRT machinery has not been reported to repair Golgi membranes. Similarly, nor has STING activation been shown to directly damage Golgi membrane. We predict ESCRT machinery is more likely to interact with STING at later post-Golgi vesicles, which fits with the data and timepoints utilised in our immunoprecipitation (**Fig 5B, Fig EV4B,C**) and immunofluorescence experiments (**Fig 5C-F, Fig EV4D, Appendix Fig S3, Fig S4**).

e. Contrary to claims in the text (l.46, l.121, l.290, l.300, l.317-318, l.1087), they do not show that STING is ubiquitinated. They can however detect ubiquitinated proteins in a STING IP (with a major protein at ~52 kDa, Fig 5B). They should carefully revise the text.

We believe we have now clarified this point. Please see our response to **point 3c** above and to **reviewer 1–point 6**.

4. The identification of an essential factor of the ESCRT machinery using convincing genetic depletion is crucial. The current data with HRS is encouraging but not convincing due to the low level of depletion. I understand that HRS is essential for viability and that they cannot make full KO cells. However, their inducible Crispr/Cas9 silencing system is just not working well. They can probably obtain much better results with siRNAs or inducible shRNA lentivectors. They should also test the specificity of HRS for STING signaling using control ligands (e.g. RIG-I/MDA5).

We thank the reviewer for their helpful suggestion. As discussed in response to **reviewer 1–point 7**, by adjusting the conditions we were able to improve the knockdown of HRS using CRISPRi targeting (**Fig 6**). Utilising this new set up we also included 2’3’-cGAM(PS)2 stimulation (as well as DMXAA) where we could observe increased STING-dependent signalling (**Fig 6A**) and cytokine production (**Fig 6B,C**) in HRS knockdown macrophages. As requested by the reviewer we also performed additional control experiments utilising non-

STING ligands. As shown in **Fig EV5A-C**, the HRS-depleted cells maintained normal responses to LPS (TLR4) and Pam3CysK4 (TLR1/2) stimulation.

5. The analysis of phosphorylation of STING and cellular proteins shown in Fig 1 is impressive but only serves to report that many trafficking proteins are detected. This data seems under-exploited and is superfluous to rationalize an analysis of STING trafficking. Can the authors make any link with the ESCRT machinery specifically? Are there phospho-proteins of interest that can be tested? And what is the role of phosphorylated residues in STING in its degradation? If they cannot better link this dataset, the authors should consider removing it.

We politely disagree with the reviewer and believe this valuable dataset is an important addition to the manuscript and for the wider scientific community. As discussed in the manuscript we were able to detect phosphorylation of trafficking and lysosomal-targeting proteins (**Fig EV2**). Moreover, we did specifically detect direct phosphorylation of the ESCRT-III protein CHMP4B (**Fig EV2B**), which was an important finding leading to our further investigation of the ESCRT pathway in STING degradation. We also thank the reviewer for their interesting suggestions to undertake experiments examining phospho-proteins that we identified in our screen, however we believe these studies are far beyond the scope of this manuscript, which already contains mechanistic insight into STING degradation via ESCRT.

Other comments:
6. Abstract l.51: "a" rather than "the" mechanisms

This has been altered in the text.

7. L.147: Avoid claims of novelty

We have removed this line to avoid claims of novelty.

8. L.199 Fig 2E indicates "endosomes" but these are rather undefined "vesicles"?

We have removed specific reference to endosomes.

9. L.219-221, l.233-234: Masking of STING detection by monoclonals due to PTMs can be easily addressed using polyclonal antibodies, this should be tested instead of leaving hanging such speculation. Furthermore, the two antibodies in Fig 3C show the same pattern, there are just different intensities through all time points (even without ligand at t=0). Therefore, the differences are not related to PTM changes during activation, but rather to differences in sensitivity between the two antibodies and the ECL assay. Again, this should be addressed with a polyclonal antibody.

We have removed this data from Figure 3 and the corresponding discussion from the manuscript.

10. Statistics: a t-test cannot be used if the number of groups shown on the graph is > 2. They need to use an ANOVA instead (Fig 3F, 3G).

We thank the reviewer for their advice. In addition, this data has since been removed from the manuscript.

11. Claims for Fig 4D/E are not supported by statistical analysis, this needs to be performed. Consider normalizing the data with a log transformation.

We thank the reviewer for this observation. We have performed statistical analysis using two-way ANOVA and observed statistically significant differences at the 4 h time point.

Referee #3

Conducting a phosphoproteomic screen following STING activation, the authors identify various proteins associated with endosomal and lysosomal function to be phosphorylated. Following up on STING trafficking using live cell microscopy, the authors first corroborate previous work on STING translocation to the Golgi apparatus. Moreover, they find that STING levels decrease upon stimulation and that previously stimulated cells displayed a blunted STING response upon re-stimulation. This phenomenon was partly related to reduced STING levels, which were slow to recover, but also to additional, unspecified mechanisms that led to refractory STING signaling. Focusing on how STING is degraded in the lysosome, the authors then turn their attention to the ESCRT machinery. Since ubiquitination precedes ESCRT dependent cargo degradation, the authors test whether blocking ubiquitination impacts on the abundance of STING following stimulation. Here, they find that blocking ubiquitination not only allows STING phosphorylation and subsequent IRF3 phosphorylation to proceed normally (in fact these two hallmarks of STING activation are indeed enhanced), but that it also rescues STING from degradation. These results indirectly imply the ESCRT pathway in STING degradation. To corroborate this notion, they go on to block the ESCRT-0 protein Hrs and they also overexpress a dominant negative version of the ESCRT-component VPS4a. Both of these perturbations lead to increased STING levels and increased activity of the STING signaling cascade, confirming the original hypothesis.

All in all, this is an interesting manuscript that describes a novel aspect of STING biology, namely how STING is degraded via an ESCRT-dependent mechanism. All in all, the experiments are performed at a high level and the claims are well supported by the data. A particularly interesting side aspect of these data is that the inhibition of ubiquitination has no negative impact on proximal events of STING activation. This sets aside several studies in the field that have proposed sophisticated models for STING ubiquitination upstream of TBK1 recruitment or activation.

Some parts of the manuscript, on the other hand, are less developed. E.g. it is unclear how STING stimulation results in this pronounced tachyphylaxis as reported in Fig. 3F/G. This is clearly not due to reduced STING levels and therefore diverts the narrative of the manuscript in another direction that has not yet been explored. Nevertheless, the manuscript is already quite mature and its results certainly warrant publication without additional mechanistic or functional studies.

We are grateful to the reviewer for their positive feedback regarding our manuscript. As discussed in response to **reviewer 1–point 3** and **reviewer 2–point 2** we have decided to remove the re-stimulation data from the original Figure 3 from our manuscript.

Dear Dom,

Thank you for submitting your revised manuscript to The EMBO Journal. Your study has now been seen by referees #1 and 2 and their comments are provided below. As you can see, the referees appreciate the introduced revisions and support publication here. Referee #2 has a few minor comments that I would like to ask you to take into consideration in a final revised version.

When you submit the revision, please also take care of the following editorial points.

- The following funding information is missing in the journal submission system: Monash Silver Jubilee Postgraduate Research scholarship; Monash Graduate Excellence Scholarship; Australian Government Research Training Program Scholarships; Monash University FMNHS Senior Postdoctoral Fellowship;

- Remove the Authors Contributions from the manuscript. The 'Author Contributions' section is replaced by the CRediT contributor roles taxonomy to specify the contributions of each author in the journal submission system. Please use the free text box in the 'author information' section of the manuscript submisssion system to provide more detailed descriptions (e.g., 'X provided intracellular Ca++ measurements in fig Y')

- Check figure callouts for Figure 3G-I (I think it should be Figure 4G-I) & Appendix Figure S4 panels

- Please add page numbers to the ToC; nomenclature should be Appendix Figure S1-S4 (the callouts are correct)

- Source data files need to be reorganized to one file/folder per figure and ZIPing for each main figure. For EV and/or appendix figures, ZIP together all source data.

- The synopsis image is too large should be 550 wide by 400

- Make sure that the deposited proteomics dataset is open and available.

- Regarding the movies - the legends should be removed from MS file and zipped with each movie file.

- Figure issues:

- Figure 4D - Images DMSO / DMXAA 1 H & DMXAA 2H look identical. Please look and clarify.

- Reuse of Figure 5D And EV4D images, which is labelled in the figure legend. Please check the call out in the figure legend is correct. It says Figure 4D but I think should be 5D.

- It looks like the untreated image in Figure 5C is reused in Figure Appendix S3. Please look and if correct state this in the figure legend.

- Our publisher has done their pre-publication check on your manuscript. When you log into the manuscript submission system you will see the file "Data Edited Manuscript file". Take a look at the word file and the comments regarding the figure legends and respond to the issues.

Please send a point-by-point response also to the editorial points when you resubmit

That should be it- Congratulations on a nice study! Let me know if you have any further questions

best Karin

Karin Dumstrei, PhD
Senior Editor
The EMBO Journal

Instructions for preparing your revised manuscript:

Guide For Authors: https://www.embopress.org/page/journal/14602075/authorguide

----

Referee #1:

I went through the point to point response to my comments and I am content with the answers and new experiments added by the authors. They addressed all my concerns adequately. I have no further comments.

Referee #2:

The authors have performed additional experiments and addressed my comments. This is an interesting and convincing study overall.

Minor remaining comments:

1. In the absence of identified lysine mutant, it remains unconvincing that STING is directly ubiquitinated (Fig 5B) in response to ligand stimulation and this has not improved. I also do not see a convincing "appearance of distinctive smearing pattern" as they state in their response to Reviewer 1 for Fig 5A. These limitations should be discussed in the paper.

2. The implication of HRS has improved but this remains the weakest point of the study. Even with the new optimized condition of CRISPRi, a substantial amount of HRS protein remains and the effects on interferon gene and protein are subtle. Of note, there seems to be a stronger effect on IL-6 production. It may be that HRS has a more direct effect on the termination of the NFkB-mediated arm of STING signaling, rather than the IRF3 arm. It is worth discussing this.

3. Fig 5D: HRS label is missing

Point-by Point response to *Balka et al.,* (Manuscript EMBOJ-2022-112712R1).

Editor/Reviewer comments are shown in *italics* and our responses are shown in blue.

Editorial comments:

- The following funding information is missing in the journal submission system: Monash Silver Jubilee Postgraduate Research scholarship; Monash Graduate Excellence Scholarship; Australian Government Research Training Program Scholarships; Monash University FMNHS Senior Postdoctoral Fellowship;

We have added the funding information to the journal submission system. (However, please note the scholarships/fellowships do not have a grant reference number).

- Remove the Authors Contributions from the manuscript. The 'Author Contributions' section is replaced by the CRediT contributor roles taxonomy to specify the contributions of each author in the journal submission system. Please use the free text box in the 'author information' section of the manuscript submission system to provide more detailed descriptions (e.g., 'X provided intracellular Ca++ measurements in fig Y')

This has been removed from the manuscript file and updated in the journal submission system.

- Check figure callouts for Figure 3G-I (I think it should be Figure 4G-I) & Appendix Figure S4 panels

Thank you for your observation. We have checked these and amended as required.

- Please add page numbers to the ToC; nomenclature should be Appendix Figure S1-S4 (the callouts are correct)

We have updated the Appendix file as requested.

- Source data files need to be reorganized to one file/folder per figure and ZIPing for each main figure. For EV and/or appendix figures, ZIP together all source data.

Source data has now been uploaded into the appropriate ZIP files.

- The synopsis image is too large should be 550 wide by 400

Thank you, we have now altered the dimensions of the synopsis image to 550 x 400.

- Make sure that the deposited proteomics dataset is open and available.

The dataset has been deposited under the identifier PXD037108. When we receive a DOI and/or Pubmed ID number the dataset will be made publicly available and linked to the article.

- Regarding the movies - the legends should be removed from MS file and zipped with each movie file.

The figure legends for each movie have been added as a text file and zipped with the appropriate movie.

Figure issues:

- Figure 4D - Images DMSO / DMXAA 1 H & DMXAA 2H look identical. Please look and clarify.

Thank you for noticing this error. The image for DMXAA 1h (and the source data) has been updated in the revised version.

- Reuse of Figure 5D And EV4D images, which is labelled in the figure legend. Please check the call out in the figure legend is correct. It says Figure 4D but I think should be 5D.

Thank you for picking this up – this has been fixed in the figure legend.

- It looks like the untreated image in Figure 5C is reused in Figure Appendix S3. Please look and if correct state this in the figure legend.

This is correct. We have added this into the figure legend for Appendix Figure S3.

- Our publisher has done their pre-publication check on your manuscript. When you log into the manuscript submission system you will see the file "Data Edited Manuscript file". Take a look at the word file and the comments regarding the figure legends and respond to the issues.

We thank the publisher for their check. We have made the requested changes in the revised manuscript.

Referee comments

Referee #1:

I went through the point to point response to my comments and I am content with the answers and new experiments added by the authors. They addressed all my concerns adequately. I have no further comments.

We are very grateful to the reviewer for their positive response to our revised manuscript.

Referee #2:

The authors have performed additional experiments and addressed my comments. This is an interesting and convincing study overall.

We thank the reviewer for their encouraging feedback regarding our manuscript.

Minor remaining comments:
1. In the absence of identified lysine mutant, it remains unconvincing that STING is directly ubiquitinated (Fig 5B) in response to ligand stimulation and this has not improved. I also do not see a convincing "appearance of distinctive smearing pattern" as they state in their response to Reviewer 1 for Fig 5A. These limitations should be discussed in the paper.

We agree that we have not investigated the ubiquitination of STING in detail. We have now mentioned this limitation in the revised discussion and directed the readers to additional studies that have investigated STING lysine residues in the context of STING degradation via ESCRT (can be found in lines 394-400). We politely disagree with the reviewer that the TUBE data presented in Fig 5A does not demonstrate smearing associated with STING ubiquitination.

2. The implication of HRS has improved but this remains the weakest point of the study. Even with the new optimized condition of CRISPRi, a substantial amount of HRS protein remains and the effects on interferon gene and protein are subtle. Of note, there seems to be a stronger effect on IL-6 production. It may be that HRS has a more direct effect on the termination of the NFkB-mediated arm of STING signaling, rather than the IRF3 arm. It is worth discussing this.

This is an interesting point raised by the reviewer. Of note we did see a stronger phenotype when blocking VPS4a activity (Figure 7). In line with our data, two recent studies also implicating ESCRT machinery in STING degradation (PMID36739287, PMID36918692) compared different ESCRT components to HRS and found that knockdown of alternative ESCRT proteins had a stronger effect on STING cytokine production and/or degradation (e.g. VPS37, TSG101, CHMP4B etc) compared to HRS knockdown. Furthermore, we have mentioned this in the manuscript (lines 404-408) and also added a comment on the apparent relative sensitivity of IL6 production induced by HRS knockdown (lines 348-350).

3. Fig 5D: HRS label is missing

We thank the reviewer for this observation. This has been updated on the figure.

2nd Revision - Editorial Decision 17th Apr 2023

Dear Dom,

Thank you for submitting your revised manuscript to The EMBO Journal. I have now looked at everything and all looks good.

I am therefore very pleased to accept the manuscript for publication here.

Congratulations on a nice study

with best wishes

Karin

Karin Dumstrei, PhD
Senior Editor
The EMBO Journal

Please note that it is EMBO Journal policy for the transcript of the editorial process (containing referee reports and your response letter) to be published as an online supplement to each paper. If you do NOT want this, you will need to inform the Editorial Office via email immediately. More information is available here: https://www.embopress.org/page/journal/14602075/authorguide#transparentprocess

Your manuscript will be processed for publication in the journal by EMBO Press. Manuscripts in the PDF and electronic editions of The EMBO Journal will be copy edited, and you will be provided with page proofs prior to publication. Please note that supplementary information is not included in the proofs.

You will be contacted by Wiley Author Services to complete licensing and payment information. The required 'Page Charges Authorization Form' is available here: https://www.embopress.org/pb-assets/embo-site/tej_apc.pdf - please download and complete the form and return to embopressproduction@wiley.com

EMBO Press participates in many Publish and Read agreements that allow authors to publish Open Access with reduced/no publication charges. Check your eligibility: https://authorservices.wiley.com/author-resources/Journal-Authors/open-access/affiliation-policies-payments/index.html

Should you be planning a Press Release on your article, please get in contact with embojournal@wiley.com as early as possible, in order to coordinate publication and release dates.

If you have any questions, please do not hesitate to call or email the Editorial Office. Thank you for your contribution to The EMBO Journal.

EMBO Press Author Checklist

Corresponding Author Name: Dominic De Nardo
Journal Submitted to: The EMBO Journal
Manuscript Number: EMBOJ-2022-112712R

USEFUL LINKS FOR COMPLETING THIS FORM
The EMBO Journal - Author Guidelines
EMBO Reports - Author Guidelines
Molecular Systems Biology - Author Guidelines
EMBO Molecular Medicine - Author Guidelines

Reporting Checklist for Life Science Articles (updated January
This checklist is adapted from Materials Design Analysis Reporting (MDAR) Checklist for Authors. MDAR establishes a minimum set of requirements in transparent reporting in the life sciences (see Statement of Task: 10.31222/osf.io/9sm4x). Please follow the journal's guidelines in preparing your
Please note that a copy of this checklist will be published alongside your article.

Abridged guidelines for figures
1. Data
The data shown in figures should satisfy the following conditions:
- the data were obtained and processed according to the field's best practice and are presented to reflect the results of the experiments in an accurate and unbiased manner.
- ideally, figure panels should include only measurements that are directly comparable to each other and obtained with the same assay.
- plots include clearly labeled error bars for independent experiments and sample sizes. Unless justified, error bars should not be shown for technical
- if n<5, the individual data points from each experiment should be plotted. Any statistical test employed should be justified.
- Source Data should be included to report the data underlying figures according to the guidelines set out in the authorship guidelines on Data

2. Captions
Each figure caption should contain the following information, for each panel where they are relevant:
- a specification of the experimental system investigated (eg cell line, species name).
- the assay(s) and method(s) used to carry out the reported observations and measurements.
- an explicit mention of the biological and chemical entity(ies) that are being measured.
- an explicit mention of the biological and chemical entity(ies) that are altered/varied/perturbed in a controlled manner.
- the exact sample size (n) for each experimental group/condition, given as a number, not a range;
- a description of the sample collection allowing the reader to understand whether the samples represent technical or biological replicates (including how many animals, litters, cultures, etc.).
- a statement of how many times the experiment shown was independently replicated in the laboratory.
- definitions of statistical methods and measures:
  - common tests, such as t-test (please specify whether paired vs. unpaired), simple χ2 tests, Wilcoxon and Mann-Whitney tests, can be unambiguously identified by name only, but more complex techniques should be described in the methods section;
  - are tests one-sided or two-sided?
  - are there adjustments for multiple comparisons?
  - exact statistical test results, e.g., P values = x but not P values < x;
  - definition of 'center values' as median or average;
  - definition of error bars as s.d. or s.e.m.

Please complete ALL of the questions below.
Select "Not Applicable" only when the requested information is not relevant for your study.

Materials

Newly Created Materials	Information included in the manuscript?	In which section is the information available? (Reagents and Tools Table, Materials and Methods, Figures, Data Availability Section)
New materials and reagents need to be available; do any restrictions apply?	Yes	No restrictions - upon request. Materials and Methods

Antibodies	Information included in the manuscript?	In which section is the information available? (Reagents and Tools Table, Materials and Methods, Figures, Data Availability Section)
For **antibodies** provide the following information:<br>- Commercial antibodies: RRID (if possible) or supplier name, catalogue number and or/clone number<br>- Non-commercial: RRID or citation	Yes	Materials and Methods

DNA and RNA sequences	Information included in the manuscript?	In which section is the information available? (Reagents and Tools Table, Materials and Methods, Figures, Data Availability Section)
**Short novel DNA or RNA including primers, probes**: provide the sequences.	Yes	Materials and methods

Cell materials	Information included in the manuscript?	In which section is the information available? (Reagents and Tools Table, Materials and Methods, Figures, Data Availability Section)
**Cell lines:** Provide species information, strain. Provide accession number in repository **OR** supplier name, catalog number, clone number, and/**OR** RRID.	Yes	Materials and Methods
**Primary cultures:** Provide species, strain, sex of origin, genetic modification status.	Yes	Materials and Methods
Report if the cell lines were recently **authenticated** (e.g., by STR profiling) and tested for mycoplasma contamination.	Not Applicable	

Experimental animals	Information included in the manuscript?	In which section is the information available? (Reagents and Tools Table, Materials and Methods, Figures, Data Availability Section)
**Laboratory animals or Model organisms:** Provide species, strain, sex, age, genetic modification status. Provide accession number in repository **OR** supplier name, catalog number, clone number, **OR** RRID.	Yes	Materials and Methods
**Animal observed in or captured from the field:** Provide species, sex, and age where possible.	Not Applicable	
Please detail **housing and husbandry conditions**.	Yes	Materials and Methods

Plants and microbes	Information included in the manuscript?	In which section is the information available? (Reagents and Tools Table, Materials and Methods, Figures, Data Availability Section)
**Plants:** provide species and strain, ecotype and cultivar where relevant, unique accession number if available, and source (including location for collected wild specimens).	Not Applicable	
**Microbes:** provide species and strain, unique accession number if available, and source.	Not Applicable	

Human research participants	Information included in the manuscript?	In which section is the information available? (Reagents and Tools Table, Materials and Methods, Figures, Data Availability Section)
If collected and within the bounds of privacy constraints report on age, sex and gender or ethnicity for all study participants.	Not Applicable	

Core facilities	Information included in the manuscript?	In which section is the information available? (Reagents and Tools Table, Materials and Methods, Figures, Data Availability Section)
If your work benefited from core facilities, was their service mentioned in the acknowledgments section?	Yes	Acknowledgements

Design

Study protocol	Information included in the manuscript?	In which section is the information available? (Reagents and Tools Table, Materials and Methods, Figures, Data Availability Section)

If study protocol has been **pre-registered, provide DOI in the manuscript**. For clinical trials, provide the trial registration number **OR** cite DOI.	Not Applicable	
Report the **clinical trial registration number** (at ClinicalTrials.gov or equivalent), where applicable.	Not Applicable	

Laboratory protocol	Information included in the manuscript?	In which section is the information available? (Reagents and Tools Table, Materials and Methods, Figures, Data Availability Section)
Provide DOI OR other citation details if **external detailed step-by-step protocols** are available.	Not Applicable	

Experimental study design and statistics	Information included in the manuscript?	In which section is the information available? (Reagents and Tools Table, Materials and Methods, Figures, Data Availability Section)
Include a statement about **sample size** estimate even if no statistical methods were used.	Yes	Figure Legends and Materials and Methods
Were any steps taken to minimize the effects of subjective bias when allocating animals/samples to treatment (e.g. **randomization procedure**)? If yes, have they been described?	Not Applicable	
Include a statement about **blinding** even if no blinding was done.	Yes	Materials and Methods
Describe **inclusion/exclusion criteria** if samples or animals were excluded from the analysis. Were the criteria pre-established?<br><br>If sample or data points were omitted from analysis, report if this was due to attrition or intentional exclusion and provide justification.	Not Applicable	
For every figure, are **statistical tests** justified as appropriate? Do the data meet the assumptions of the tests (e.g., normal distribution)? Describe any methods used to assess it. Is there an estimate of variation within each group of data? Is the variance similar between the groups that are being statistically compared?	Yes	Figure legends and Materials and Methods

Sample definition and in-laboratory replication	Information included in the manuscript?	In which section is the information available? (Reagents and Tools Table, Materials and Methods, Figures, Data Availability Section)
In the figure legends: state number of times the experiment was **replicated** in laboratory.	Yes	Figure Legends
In the figure legends: define whether data describe **technical or biological replicates.**	Yes	Figure Legends

Ethics

Ethics	Information included in the manuscript?	In which section is the information available? (Reagents and Tools Table, Materials and Methods, Figures, Data Availability Section)
Studies involving **human participants**: State details of **authority granting ethics approval** (IRB or equivalent committee(s), provide reference number for approval.	Not Applicable	
Studies involving **human participants**: Include a statement confirming that **informed consent** was obtained from all subjects and that the experiments conformed to the principles set out in the WMA Declaration of Helsinki and the Department of Health and Human Services Belmont Report.	Not Applicable	
Studies involving **human participants:** For publication of **patient photos**, include a statement confirming that consent to publish was obtained.	Not Applicable	
Studies involving experimental **animals**: State details of **authority granting ethics approval** (IRB or equivalent committee(s), provide reference number for approval. Include a statement of compliance with ethical regulations.	Yes	Materials and Methods
Studies involving **specimen and field samples:** State if relevant **permits** obtained, provide details of authority approving study; if none were required, explain why.	Not Applicable	

Dual Use Research of Concern (DURC)	Information included in the manuscript?	In which section is the information available? (Reagents and Tools Table, Materials and Methods, Figures, Data Availability Section)
Could your study fall under dual use research restrictions? Please check biosecurity documents and list of **select agents and toxins** (CDC): https://www.selectagents.gov/sat/list.htm	Not Applicable	
If you used a select agent, is the security level of the lab appropriate and reported in the manuscript?	Not Applicable	
If a study is subject to dual use research of concern regulations, is the name of the **authority granting approval and reference number** for the regulatory approval provided in the manuscript?	Not Applicable	

Reporting

The MDAR framework recommends adoption of discipline-specific guidelines, established and endorsed through community initiatives. Journals have their own policy about requiring specific guidelines and recommendations to complement MDAR.

Adherence to community standards	Information included in the manuscript?	In which section is the information available? (Reagents and Tools Table, Materials and Methods, Figures, Data Availability Section)
State if relevant guidelines or checklists (e.g., **ICMJE, MIBBI, ARRIVE, PRISMA**) have been followed or provided.	Not Applicable	
For **tumor marker prognostic studies,** we recommend that you follow the **REMARK** reporting guidelines (see link list at top right). See author guidelines, under 'Reporting Guidelines'. Please confirm you have followed these guidelines.	Not Applicable	
For **phase II and III randomized controlled trials**, please refer to the **CONSORT** flow diagram (see link list at top right) and submit the CONSORT checklist (see link list at top right) with your submission. See author guidelines, under 'Reporting Guidelines'. Please confirm you have submitted this list.	Not Applicable	

Data Availability

Data availability	Information included in the manuscript?	In which section is the information available? (Reagents and Tools Table, Materials and Methods, Figures, Data Availability Section)
Have **primary datasets** been deposited according to the journal's guidelines (see 'Data Deposition' section) and the respective accession numbers provided in the Data Availability Section?	Yes	Data Availability Section
Were **human clinical and genomic datasets** deposited in a public access-controlled repository in accordance to ethical obligations to the patients and to the applicable consent agreement?	Not Applicable	
Are **computational models** that are central and integral to a study available without restrictions in a machine-readable form? Were the relevant accession numbers or links provided?	Not Applicable	
If publicly available data were reused, provide the respective **data citations in the reference list**.	Not Applicable	
